# Lymphocytic infiltration in stage II microsatellite stable colorectal tumors: A retrospective prognosis biomarker analysis

**DOI:** 10.1371/journal.pmed.1003292

**Published:** 2020-09-24

**Authors:** Rebeca Sanz-Pamplona, Marilena Melas, Asaf Maoz, Stephanie L. Schmit, Hedy Rennert, Flavio Lejbkowicz, Joel K. Greenson, Xavier Sanjuan, Maria Lopez-Zambrano, M. Henar Alonso, Chenxu Qu, Kevin J. McDonnell, Gregory E. Idos, Marissa Vignali, Ryan Emerson, Paul Fields, Elisabet Guinó, Cristina Santos, Ramon Salazar, Harlan S. Robins, Gad Rennert, Stephen B. Gruber, Victor Moreno

**Affiliations:** 1 Catalan Institute of Oncology (ICO), Hospitalet de Llobregat, Barcelona, Spain; 2 ONCOBELL Program, Bellvitge Biomedical Research Institute (IDIBELL), Hospitalet de Llobregat, Barcelona, Spain; 3 Consortium for Biomedical Research in Epidemiology and Public Health (CIBERESP), Spain; 4 Nationwide Children’s Hospital, Columbus, Ohio, United States of America; 5 Department of Medicine, Boston University School of Medicine, Boston, Massachusetts, United States of America; 6 H. Lee Moffitt Cancer Center and Research Institute, Tampa, Florida, United States of America; 7 Carmel Medical Center, and Technion, Haifa, Israel; 8 University of Michigan Medical School, Ann Arbor, Michigan, United States of America; 9 University Hospital Bellvitge (HUB-IDIBELL), L'Hospitalet de Llobregat, Barcelona, Spain; 10 Department of Clinical Sciences, Faculty of Medicine, University of Barcelona, Barcelona, Spain; 11 City of Hope National Medical Center, Duarte, California, United States of America; 12 Adaptive Biotechnologies, Seattle, Washington, United States of America; 13 Consortium for Biomedical Research in Oncology (CIBERONC), Spain; 14 Fred Hutchinson Cancer Research Center, Seattle, Washington, United States of America; University of Pittsburgh, UNITED STATES

## Abstract

**Background:**

Identifying stage II patients with colorectal cancer (CRC) at higher risk of progression is a clinical priority in order to optimize the advantages of adjuvant chemotherapy while avoiding unnecessary toxicity. Recently, the intensity and the quality of the host immune response in the tumor microenvironment have been reported to have an important role in tumorigenesis and an inverse association with tumor progression. This association is well established in microsatellite instable CRC. In this work, we aim to assess the usefulness of measures of T-cell infiltration as prognostic biomarkers in 640 stage II, CRC tumors, 582 of them confirmed microsatellite stable.

**Methods and findings:**

We measured both the quantity and clonality index of T cells by means of T-cell receptor (TCR) immunosequencing in a discovery dataset (95 patients with colon cancer diagnosed at stage II and microsatellite stable, median age 67, 30% women) and replicated the results in 3 additional series of stage II patients from 2 countries. Series 1 and 2 were recruited in Barcelona, Spain and included 112 fresh frozen (FF, median age 69, 44% women) and 163 formalin-fixed paraffin-embedded (FFPE, median age 67, 39% women) samples, respectively. Series 3 included 270 FFPE samples from patients recruited in Haifa, Northern Israel, as part of a large case-control study of CRC (median age 73, 46% women). Median follow-up time was 81.1 months. Cox regression models were fitted to evaluate the prognostic value of T-cell abundance and Simpson clonality of TCR variants adjusting by sex, age, tumor location, and stage (IIA and IIB). In the discovery dataset, higher TCR abundance was associated with better prognosis (hazard ratio [HR] for ≥Q1 = 0.25, 95% CI 0.10–0.63, *P* = 0.003). A functional analysis of gene expression on these tumors revealed enrichment in pathways related to immune response. Higher values of clonality index (lower diversity) were not associated with worse disease-free survival, though the HR for ≥Q3 was 2.32 (95% CI 0.90–5.97, *P* = 0.08). These results were replicated in an independent FF dataset (TCR abundance: HR = 0.30, 95% CI 0.12–0.72, *P* = 0.007; clonality: HR = 3.32, 95% CI 1.38–7.94, *P* = 0.007). Also, the association with prognosis was tested in 2 independent FFPE datasets. The same association was observed with TCR abundance (HR = 0.41, 95% CI 0.18–0.93, *P* = 0.03 and HR = 0.56, 95% CI 0.31–1, *P* = 0.042, respectively, for each FFPE dataset). However, the clonality index was associated with prognosis only in the FFPE dataset from Israel (HR = 2.45, 95% CI 1.39–4.32, *P* = 0.002). Finally, a combined analysis combining all microsatellite stable (MSS) samples demonstrated a clear prognosis value both for TCR abundance (HR = 0.39, 95% CI 0.26–0.57, *P* = 1.3e-06) and the clonality index (HR = 2.13, 95% CI 1.44–3.15, *P* = 0.0002). These associations were also observed when variables were considered continuous in the models (HR per log2 of TCR abundance = 0.85, 95% CI 0.78–0.93, *P* = 0.0002; HR per log2 or clonality index = 1.16, 95% CI 1.03–1.31, *P* = 0.016).

**Limitations:**

This is a retrospective study, and samples had been preserved with different methods. Validation series lack complete information about microsatellite instability (MSI) status and pathology assessment. The Molecular Epidemiology of Colorectal Cancer (MECC) study had information about overall survival instead of progression-free survival.

**Conclusion:**

Results from this study demonstrate that tumor lymphocytes, assessed by TCR repertoire quantification based on a sequencing method, are an independent prognostic factor in microsatellite stable stage II CRC.

## Author summary

**Why was this study done?:**

- About 20% of stage II colorectal cancer (CRC) patients experienced relapse after surgery. Thus, it is important to identify prognostic biomarkers in this specific setting.
- Lymphocytic infiltration has been associated with better prognosis in CRC patients, specifically in patients with microsatellite instable (MSI) tumors. However, this is a less studied issue in microsatellite stable (MSS) tumors.
- Novel techniques based on sequencing the T-cell receptor (TCR) gene to assess lymphocytic infiltration provide clonality information in addition to abundance. High clonality indicates that only a few abundant clones exist, whereas lower clonality indicates that multiple clones exist with similar abundance.

**What did the researchers do and find?:**

- The prognostic value of TCR abundance and clonality discovered in a retrospective study of 95 stage II colon patients. These results were replicated in 2 additional retrospective studies from Spain (*N* = 112 and 163), and other from Israel (*N* = 270).
- Results were confirmed both when fresh frozen and paraffin-embedded tissue were analyzed by immune-sequencing.
- High TCR abundance and low clonality index (more diverse repertoire) were associated with better prognosis. Though partially correlated, both provide complementary prognostic information.

**What do these findings mean?:**

- Information about lymphocytic abundance and clonality in stage II MSS CRC tumors informs about prognosis.
- These results should be validated in additional prospective studies to prove their clinical value.

## Introduction

Colorectal cancer (CRC) is the third most common cancer worldwide, with more than 1.4 million new cases diagnosed annually [1]. A remarkable feature of CRC is the difference in prognosis of patients diagnosed at early versus late stages of the disease: Stage I and II have low to moderate risk of progression after surgical resection (about 5% and 20%, respectively), whereas patients with stage III have a higher chance of progression [2,3]. Postsurgical adjuvant chemotherapy is the standard of care for stage III patients, but guidelines differ with respect to recommendations for adjuvant therapy for patients with stage II disease. Recognized clinical risk factors for progression (emergency presentation, poorly differentiated tumor, depth of tumor invasion, and adjacent organ involvement) are insufficient to identify those patients with stage II CRC at higher risk of disease progression [4,5]. Recently, as in other cancer types, an effort has been made to develop gene expression signatures useful to identify CRC patients at higher risk of relapse like Oncotype [6]. However, none of these signatures have translated into routine clinical practice. Indeed, a meta-analysis aimed to assess the predictive ability of these signatures revealed that although gene expression signatures may be associated with prognosis, their ability to accurately predict patients’ risk of progression was limited, probably due to the molecular heterogeneity of tumors [7]. Therefore, the identification of new biomarkers to inform clinical decision-making for adjuvant chemotherapy is needed [8].

Immune cells clearly play an important role in tumorigenesis, because evasion of immune surveillance and/or suppression of immune system has been described as a hallmark of cancer cells [9]. In addition, it is well-known that tumor-immune interactions offer important prognostic information for some cancer patients [10]. A proposed clinical translation of these observations is the introduction of a scoring system designated Immunoscore measured by immunohistochemistry techniques and based on the enumeration of 2 lymphocyte populations (CD3/CD8) in the core of the tumor and in the invasive margin [11]. In CRC, Immunoscore has been reported as a clinically useful prognostic marker independent of traditional staging [12].

One of the mechanisms by which the immune system recognizes cancer cells is through the tumor cell presentation of neoantigens from mutated proteins on the cell surface by the HLA system (codified by major histocompatibility complex [MHC] genes) and their subsequent recognition by the T cells of the immune system [13]. The cellular adaptive immune system, in order to recognize a diverse and unpredictable broad spectrum of antigens, generates a remarkable breadth of diversity in antigen-specific T-cell receptors (TCRs) by a combinatoric shuffling of gene segments. The primary hallmark of a tumor-specific immune response is a large oligoclonal expansion of T cells within a tumor, a feature that pathology-based methods cannot assess [14]. This context of T cells within the tumor, tumor-infiltrating lymphocytes (TILs), as well as immune cells in the surrounding stroma, which can include a pathologic feature called “Crohn’s-like Lymphoid Reaction” (CLR), include a mix of T cells and B cells. Recently, we have published that both TILs and CLR are important, independent prognostic factors of survival in CRC [15]. We and others have observed an increased number of tumor-infiltrating cytotoxic T-lymphocytes in microsatellite instability (MSI) compared with microsatellite stable (MSS) tumors [16]. We have also reported the utility of *CD8A* gene expression (a surrogate of TIL infiltration) as a prognosis biomarker in a series of 100 MSS tumors [17]. Here, we aim to quantify and characterize T cells within and at the leading edge of colorectal cancers as a prognostic biomarker in a large set of stage II MSS CRCs. We utilize a quantitative technique based on DNA sequencing that measures both the quantity of T cells and their clonality. We hypothesize that PCR-based measures of T-cell infiltration offer a new prognostic biomarker for early stage, MSS colorectal cancers.

## Methods

### Patients and samples

The discovery dataset (named ICO/CLX) included a previously described set of 100 patients with colon cancer diagnosed at stage II and MSS paired normal-tumor samples (Colonomics study, “CLX”: www.colonomics.org; NCBI BioProject PRJNA188510). MSS status was determined by DNA-based microsatellite testing. None of the patients in CLX received adjuvant chemotherapy. All patients had been recruited at the Catalan Institute of Oncology (ICO) and the Bellvitge University Hospital (Barcelona, Spain). Gene expression profiling for 98 of these tumors was available [18] (GEO repository with accession GSE44076). All fresh frozen (FF) tumors and paired normal mucosa with available DNA (*n* = 95) were analyzed by means of immunosequencing.

Three independent datasets were used to replicate the consistency of the findings: (1) FF samples of tumors from a series of 112 stage II diagnosed at the same hospital as the discovery dataset. This series was unselected regarding treatment or microsatellite instability status (ICO/FF dataset). (2) FFPE samples from 170 patients from the same hospital with stage II colon cancer, none of them receiving chemotherapy (most similar replication series with respect to clinical features). After excluding 7 samples because of technical issues, 163 samples were included in the analysis (ICO/FFPE dataset). (3) FFPE samples from 270 stage II patients recruited in Haifa, Northern Israel as part of a large case-control study of CRC, the Molecular Epidemiology of Colorectal Cancer Study (MECC dataset). These patients were unselected regarding adjuvant therapy. Baseline information about the patients is shown in Table 1.

**Table 1:** Baseline characteristics.

	ICO/CLX *(n* = 95)	ICO/FF (*n* = 112)	ICO/FFPE (*n* = 163)	MECC (*n* = 270)
Gender				
Male	67 (70.5%)	63 (56.3%)	103 (63.2%)	145 (53.7%)
Female	28 (29.5%)	49 (43.7%)	60 (36.8%)	125 (46.3%)
Age				
Median (range) years	72 (43–87)	69 (32–92)	67 (37–87)	73 (24–93)
Site				
Right	38 (40%)	49 (43.7%)	64 (39.3%)	96 (35.6%)
Left	57 (60%)	63 (56.3%)	99 (60.7%)	172 (63.7%)
Unknown				2 (0.8%)
Stage				
IIA	87 (91.6%)	100 (89.3%)	133 (81.6%)	261 (97%)
IIB	8 (8.4%)	12 (10.7%)	30 (18.4%)	9 (3%)
Lymph node assessed				
Median (range)	18 (2–49)	23 (3–73)	18 (2–83)	
Microsatellite instability				
MSI	0	---	20 (12.3%)	22 (8.1%)
MSS	95 (100%)	---	120 (73.6%)	248 (91.9%)
Unknown		112 (100%)	23 (14.1%)	
Recurrence^a^				
Event	22 (23.2%)	21 (18.75%)	24 (14.7%)	56 (20.7%)
No event	73 (76.8%)	91 (81.25%)	139 (85.3%)	214 (79.3%)
Recurrence-free time Median (range) months	63 (7–137)	65 (0.2–234)	66 (0.4–210)	80 (0.9–222)
Pathology assessment mean (range)				
TILs	1.2 (0–8.8)	---	---	1.6 (0–46)
STLs (%)	17.6 (5–88)	---	---	---
Stroma/Tumor %	44.4 (10–90)	---	---	---

^a^Recurrence for ICO and death related to CRC for MECC.

CLX, Colonomics Study; CRC, colorectal cancer; FF, fresh frozen; FFPE, formalin-fixed paraffin-embedded; ICO, Catalan Institute of Oncology; MECC, Molecular Epidemiology of Colorectal Cancer Study, STL, stromal lymphocyte; TIL, tumor-infiltrating lymphocyte.

### Ethics statement

All procedures performed were in accordance with the ethical standards of studies involving human participants. Written informed consent was obtained from patients. The Bellvitge University Hospital Ethics Committee approved the study protocol (PR112/15). Also, the Institutional Review Board at the University of Southern California approved this study (HS-12-00324).

### T-cell infiltration measurement by DNA sequencing

All samples in the 4 datasets were analyzed by immunosequencing. A multiplex PCR system was used to amplify the variable CDR3β sequences of the TCR from DNA segments in 7 gene families, 10 orphan segments in 10 gene families, both D genes and the 13 functional J segments. This approach generated an 87 base-pair fragment capable of identifying the VDJ region spanning each unique CDR3β. Then, amplicons were sequenced using the Illumina HiSeq platform. Both TCR abundance and clonality metrics were calculated. Using a baseline developed from a suite of synthetic templates, primer concentrations and computational corrections were used to correct for the primer bias common to multiplex PCR reactions. Raw sequence data were filtered based on the TCRβ V, D, and J gene definitions provided by the international ImMunoGeneTics information system (IMGT) database and binned using a modified nearest-neighbor algorithm to merge closely related sequences and remove both PCR and sequencing errors. The fraction of T cells in FFPE tissue samples was calculated by normalizing TCR-β template counts to the total amount of DNA usable for TCR sequencing, where the amount of usable DNA was determined by PCR-amplification and sequencing of several housekeeping genes that are expected to be present in all nucleated cells with the same length amplicons. In that way, TIL fraction in a sample could be measured independent of the extent of the degradation. The approach was capable of detecting 1 cell in 200,000 [19]. Two metrics were derived from the raw sequences, TCR abundance (estimated as the normalized number of TCR reads over the estimate of the total number of cells) and TCR clonality. In order to quantify the clonality and diversity of the sequences of the TCRs observed within different components of colorectal cancers, a modification of the Simpson diversity index was applied to immunoSEQ data. As demonstrated by Parameswaran and colleagues [20], sequence-based immune monitoring of the antibody response can be quantified on a scale ranging from 0–1 using the Simpson diversity index = $\sum p_{i}^{2}$, where *p_i_* is the frequency of each productive rearrangement. Here we used a variation of the Simpson diversity index, which we term “Simpson clonality,” calculated as the square root of the Simpson diversity index $SC=\sqrt{\sum p_{i}^{2}}$. We have found this metric to be more robust to a range of template counts and T-cell fraction than the classic clonality metric while conveying the same information and within the same range of 0–1. High Simpson clonality indicates that one or a few clones are very abundant compared with other clones, whereas low values indicate an even distribution of multiple clones and a more diverse repertoire. In this paper, we will use clonality index as indicative of the Simpson diversity index, and high clonality should be interpreted as indicator of the existence of few abundant clones.

### TIL measurement by a pathologist

In addition to immunosequencing, lymphocytic infiltration for the series ICO/CLX and MECC were analyzed by pathologist in hematoxylin and eosin (HE) stained histological slides used for diagnosis. The tumor samples from the ICO/CLX discovery series were examined by 2 pathologists (XS, MLZ) and scored for stroma and lymphocyte abundance. Three histological variables were studied: stromal lymphocytes (STLs), tumor-infiltrating lymphocytes (TILs), and the proportion of stroma/tumor. To analyze STLs, the pathologist evaluated 5 histological regions at a high-powered field (HPF, 400×) measuring the percentage of lymphocytes and plasmatic cells (excluding polymorphonuclear neutrophils) in relation to the surrounding stroma of the tumor. Hotspot, necrotic, hyalinization, and out-of-the-tumor growth areas were avoided. The mean from the 5 evaluated fields was calculated to obtain the percentage of STLs. The proportion of lymphocytes and plasmatic cells relative to total number of cells in the field was used instead of counts, because lymphocytes are usually very abundant in the stroma surrounding colon tumors, and accurate counting is not feasible [21]. To analyze TILs, 5 tumor hotspot areas were evaluated at high power. The number of lymphocytes within tumor cells were counted, avoiding apoptotic and mitotic cells. In each tumor, the average number of TIL per HPF was calculated. Finally, the proportion of stroma to tumor was evaluated at a 100× field of the tumor growth edge. Mucinous and necrotic areas were excluded. A field in which the tumor was visualized in all field edges was chosen. In heterogeneous tumors, the proportion stroma was measured in the field with the higher stroma component and an estimated percentage of stroma in relation with the tumor was calculated.

The MECC study took advantage of uniform histopathologic review by a single pathologist (JKG). Methods and procedures for pathologic evaluation have been previously described [15], and in brief, HE stained slides were scored for histology, grade, TIL per HPF, and Crohn’s like reaction (CLR), among other features. The proportion of lymphocytes in the stroma and the proportion of stroma to tumor was not available for these tumors. Analyses were restricted to cases with pathologically confirmed adenocarcinoma.

### Statistical analysis plan

The study did not have a formal analysis plan. The discovery series ICO/CLX was established in 2011 and was immunosequencing in 2015. The prognosis value of TCR abundance and clonality was initially assessed using a binary cutoff based on the median. Then optimal cutoffs were searched to improve the discriminant value of the variables. For TCR abundance, the first quartile was selected, and for clonality, the third quartile. Then, independent studies were obtained to replicate the findings. First and second replication studies, performed at the same institution in 2016, requested respectively available FF samples of CRC in the tumor bank and DNA extracted from FFPE from stage II CRC samples. The TCR analysis of the FFPE samples used a different calibration method, which had strong batch effect and rendered the results in a very different distribution of values. We had to calculate new cutoffs for the FFPE series and used the same quartiles as those of the discovery series (Q1 for TCR abundance and Q3 for clonality). Finally, existing samples from stage II CRC from a different country (MECC study, 2018) were also analyzed. Again, the distribution of values was different, and we calculated new study-specific cutoffs based on the same quartiles (see in Table 2 the distribution values and cutoffs used). Cutoffs were required to plot survival curves and try to define a threshold with clinical utility, but that aim could not be accomplished in this study because of the evolving immunosequencing technique that required changing the cutoffs. To overcome the problem of cutoffs, and at the request of a reviewer, the analysis of the variables as continuous were also performed. Because the quantitative values were skewed, log2 transformation of the TCR abundance and clonality values were used in survival models, and hazard ratios (HRs) can be interpreted as the relative hazard associated with doubling the value of TCR abundance or clonality. A few missing values existed for clonality in some studies (Table 2), and these cases have been excluded from the analyses. Missing values for MSI status in the ICO/FFPE study (*n* = 23) were imputed using the prediction of a logistic model with clinical variables in complete cases.

**Table 2:** TCR abundance and clonality prognostic value.

	ICO/CLX (*n* = 95)	ICO/FF (*n* = 112)	ICO/FFPE (*n* = 163)	MECC (*n* = 270)	Combined MSS (*n* = 575)^a^
TCR abundance					
Mean (range)	0.05 (0–0.18)	0.05 (0–0.16)	0.17 (0.01–0.66)	0.12 (0.003–0.63)	0.10 (0–0.66)
IQR (Q1-Q3)	0.04 (0.03–0.07)	0.05 (0.022–0.07)	0.11 (0.10–0.21)	0.09 (0.06–0.15)	0.10 (0.04–0.14)
< Q1	< 0.03 = 22	< 0.022 = 28	< 0.10 = 56	< 0.06 = 62	149
≥ Q1 HR [95% CI]^b^	≥ 0.03 = 73 0.25 [0.10–0.63]	≥ 0.022 = 84 0.30 [0.12–0.72]	≥ 0.10 = 107 0.41 [0.18–0.93]	≥ 0.06 = 208 0.56 [0.31–1.00]	426 0.39 [0.26–0.57]
*P* value	0.003	0.007	0.034	0.042	1.31e-06
Clonality index					
Mean (range)	0.06 (0.02–0.18)	0.06 (0.02–0.22)	0.11 (0.04–0.52)	0.18 (0.04–0.74)	0.12 (0.02–0.74)
IQR (Q1–Q3)	0.03 (0.04–0.069)	0.04 (0.04–0.08)	0.06 (0.07–0.13)	0.11 (0.09–0.20)	0.07 (0.06–0.13)
< Q3	< 0.069 = 68	< 0.08 = 84	< 0.13 = 103	< 0.20 = 187	404
≥ Q3 HR [95% CI]^b^	≥ 0.069 = 26 2.32 [0.90–5.97]	≥ 0.08 = 28 3.32 [1.38–7.94]	≥ 0.13 = 59 0.71 [0.30–1.68]	≥ 0.20 = 68 2.45 [1.39–4.32]	155 2.13 [1.44–3.15]
*P* value	0.08	0.007	0.43	0.002	0.0002
Undetermined (*N*, %)	1 (1%)	0	1 (0.6%)	15 (5.6%)	0.0002
TCR continuous (log2)					
HR [95% CI]	0.71 [0.57–0.89]	0.68 [0.51–0.91]	0.78 [0.49–1.23]	0.87 [0.69–1.10]	0.85 [0.78–0.93]
*P* value	0.003	0.010	0.29	0.24	0.002
Clonality continuous (log2)					
HR [95% CI]	1.26 [0.67–2.35]	1.56 [0.87–2.80]	1.06 [0.57–1.94]	1.28 [0.95–1.72]	1.16 [1.03–1.31]
*P* value	0.47	0.13	0.86	0.10	0.016

^a^Patients with MSI tumors excluded. Each study uses different cutoffs.

^b^HR and 95% CI, adjusted by sex, age, tumor location and stage (Stage IIA, IIB) and MSI status.

CI, confidence interval; CLX, Colonomics study; CRC, colorectal cancer; FF, fresh frozen; FFPE, formalin-fixed paraffin-embedded; HR, hazard ratio; ICO, Catalan Institute of Oncology; IQR, interquartile range; MECC, Molecular Epidemiology of Colorectal Cancer study, MSI, microsatellite instable; Q, quartile; TCR, T-cell receptor.

Disease-free survival (DFS) was used as the primary time-to-event outcome for the ICO studies (discovery and replications). Tumor recurrence, metastasis, or death were considered relevant endpoints. For the MECC dataset, disease-specific survival was used. Follow-up time was truncated at 96 months, although it was available for a longer period for most cases, but without additional observed events. Cox regression models were fitted to evaluate the prognostic value of TCR abundance and the clonality index. All models were adjusted by sex, age, tumor location, and stage (Stage IIA, IIB) and MSI status (when available). *P* values were derived from likelihood ratio tests. HRs and 95% CIs were calculated from the models. The proportionality hazards assumption was tested and was reasonable for all variables. Kaplan–Meier curves were plotted to visually represent the results.

A combined analysis of the 4 series was also performed, fitting a Cox model with the data of all the available patients and using a stratified likelihood, in addition to adjusting by potential confounders. Patients with known MSI tumors were excluded in this analysis to show that the estimate was not driven by MSI tumors that are known to have higher lymphocytic infiltrate. Also, in these models, the net effect of TCR abundance and clonality were assessed, combining them in the same model.

### Functional analysis

The Gene Set Enrichment Analysis (GSEA) algorithm (Broad Institute, https://www.gsea-msigdb.org) [22] was used to identify enrichment of specific functions in the list of genes preranked according to their level of correlation (Spearman method) with infiltration cell abundance. The statistical significance of the enrichment score was calculated by permuting the genes 1,000 times as implemented in the GSEA software.

This study is reported as per the Strengthening the Reporting of Observational Studies in Epidemiology (STROBE) guideline (S1 STROBE Checklist).

## Results

### TCR abundance and clonality index associations with prognosis in stage II CRC tumors

#### TCR measurement by immunosequencing in FF discovery dataset

TCR repertoires from a total of 95 paired adjacent normal-tumor samples achieving enough DNA quality were amplified and sequenced to quantify the number of T cells within the tumor specimen and their clonal diversity. When adjacent normal and tumor were compared, paired normal mucosa had more T cells than tumors (Mann–Whitney *P* < 0.001) and a lower clonality index (Mann–Whitney *P* = 0.02), but no meaningful differences were evident in the most frequent single clone between tumor and adjacent normal (S1 Fig).

**Fig 1:**
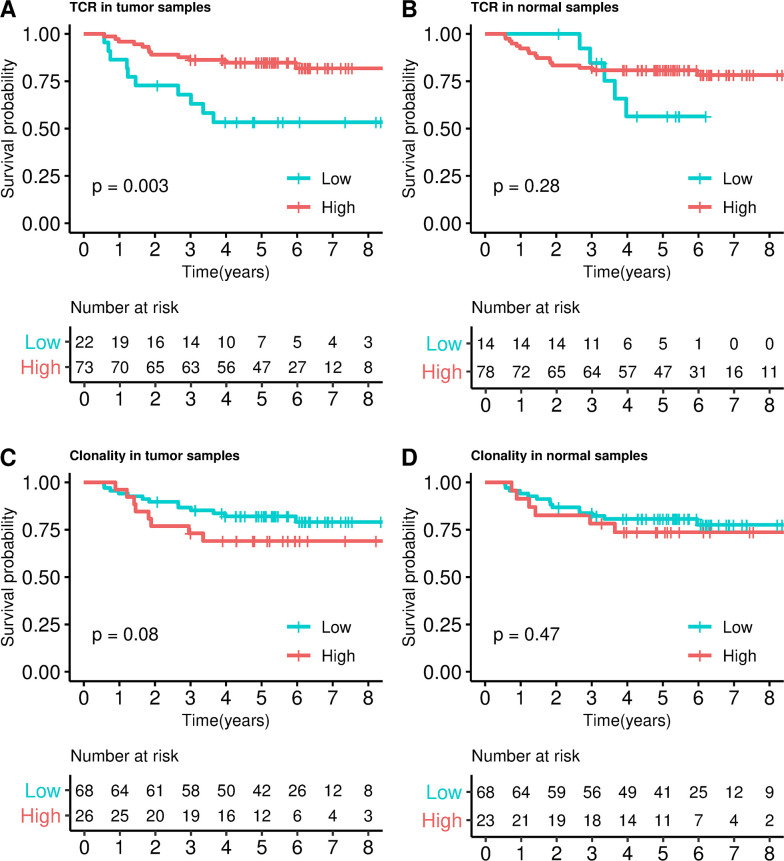
Survival analysis in ICO/CLX discovery dataset. Kaplan–Meier curves dividing into high and low TCRs categories in tumor (**A**) and adjacent normal (**B**); and into high and low clonality in tumor (**C**) and adjacent normal (**D**). *P* values were calculated by fitting a Cox regression model adjusted by sex, age, tumor location, and stage (IIA, IIB). CLX, Colonomics study; ICO, Catalan Institute of Oncology; TCR, T-cell receptor.

Regarding the association with prognosis, when patients were categorized into 2 groups, those with higher versus lower TCR abundance (Q1 cutoff = 0.03) in tumor exhibited better DFS (HR = 0.25, 95% CI 0.10–0.63, *P* = 0.003, Fig 1A). This association was also significant when TCR abundance was assessed as a continuous variable (HR = 0.71, 95% CI 0.57–0.89, *P* = 0.003). The association between TCR abundance and survival in adjacent normal tissue was not statistically significant (HR = 0.57, 95% CI 0.20–1.60, *P* = 0.28, Fig 1B).

In tumors, higher degrees of clonality index were not associated with worse DFS, though the HR for ≥Q3 was 2.32 (95% CI 0.90–5.97, *P* = 0.08, Fig 1C). When clonality index was evaluated as continuous variable, no association was found (HR = 1.26, 95% CI 0.67–2.35, *P* = 0.47). Similarly, no association with clonality index was observed when adjacent normal tissue was analyzed (HR = 1.46, 95% CI 0.52–4.08, *P* = 0.42, Fig 1D).

#### Lymphocytosis measurement by a pathologist

**Fig 2:**
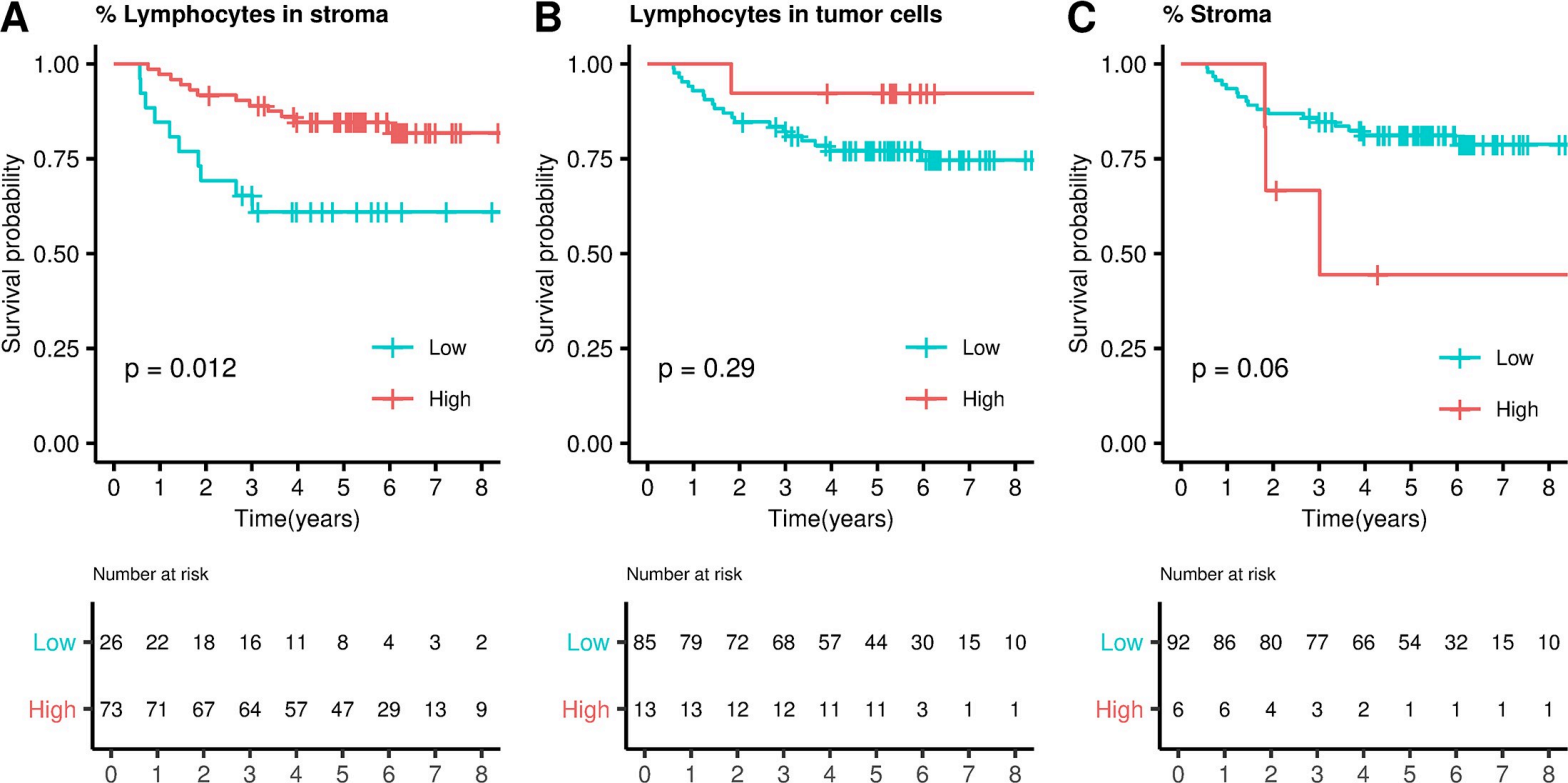
Lymphocytosis measured by a pathologist: survival analysis. Kaplan–Meier curves dividing samples into high and low lymphocytes categories in stroma (**A**) and epithelial cells (**B**). **C**. Kaplan–Meier curve dividing samples into high and low stromal infiltration. *P* values were calculated by fitting a Cox regression model adjusted by sex, age, tumor location and stage (IIA, IIB).

**Fig 3:**
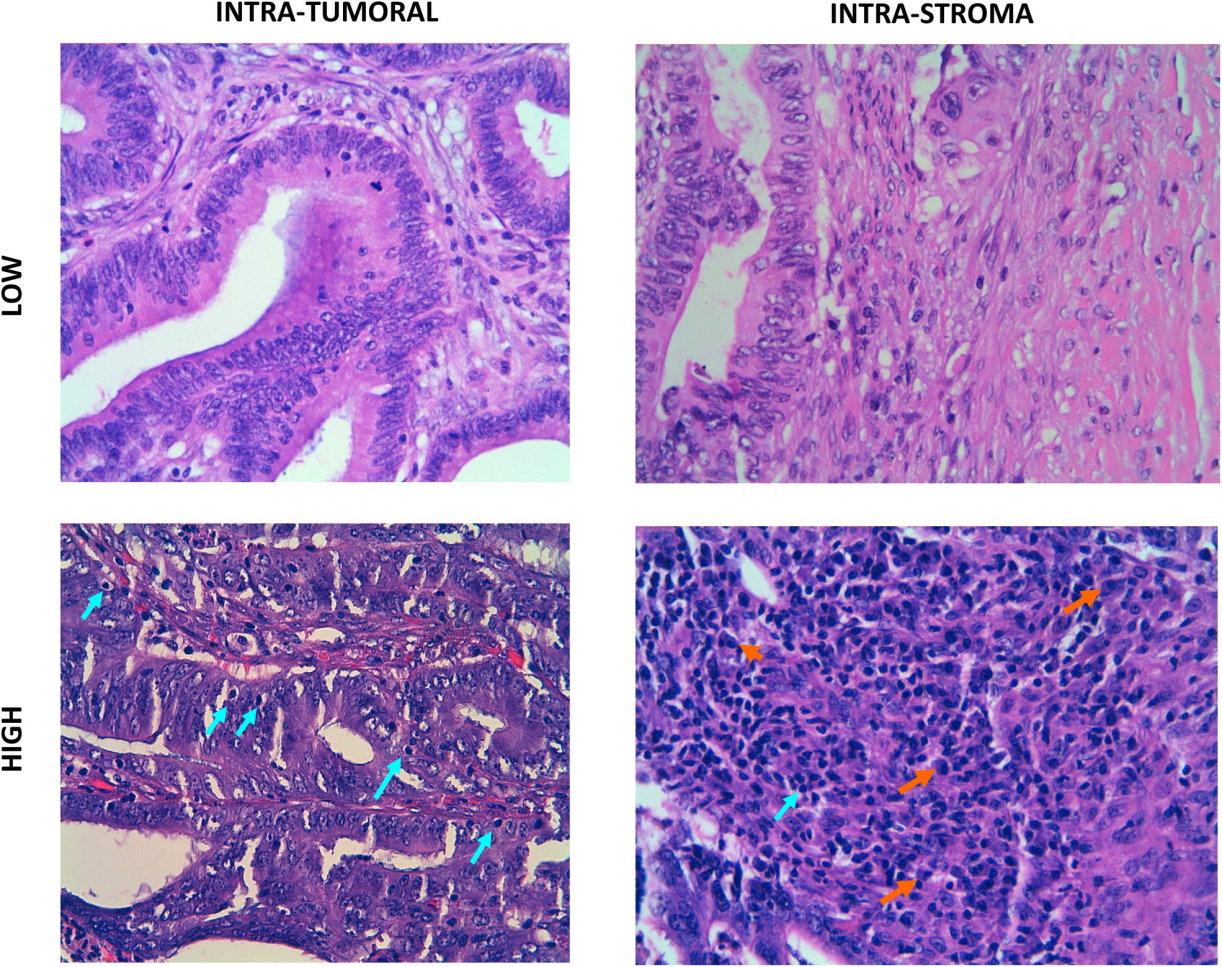
Examples of pathologist-based quantification of lymphocytes in HE slides. **A.** Low intraepithelial TILs. HE staining 400×. **B.** Low STL. HE staining 400×. **C.** High intraepithelial TILs. HE staining 400x. Intraepithelial lymphocytes (arrow). **D.** High STLs. HE staining 400×. Lymphocytes (green arrow). Plasma cells (orange arrow). HE, hematoxylin and eosin; STL, stromal lymphocyte; TIL, tumor-infiltrating lymphocyte.

A measurement of lymphocytic abundance in HE fixed slides was also performed in the ICO/CLX discovery dataset comprising 95 tumors (see Methods). The percentage of lymphocytes in the stroma was a prognosis biomarker in those tumors (optimal cutoff = 8, HR = 0.33, 95% CI 0.14–0.78, *P* = 0.012, Fig 2A) whereas the average number of lymphocytes/HPF in the tumor was not (optimal cutoff = 3, HR = 0.33, 95% CI 0.04–2.64, *P* = 0.29, Fig 2B). The percentage of tumor/stroma per sample was also explored as a prognosis biomarker, but no significant association was found (optimal cutoff = 85, HR = 3.3, 95% CI 0.91–11.95, *P* = 0.068, Fig 2C), suggesting that the most informative parameter is not the quantity but the composition of the stroma (lymphocytosis). An example of lymphocytes staining in both stromal and epithelial compartment is shown in Fig 3.

**Fig 4:**
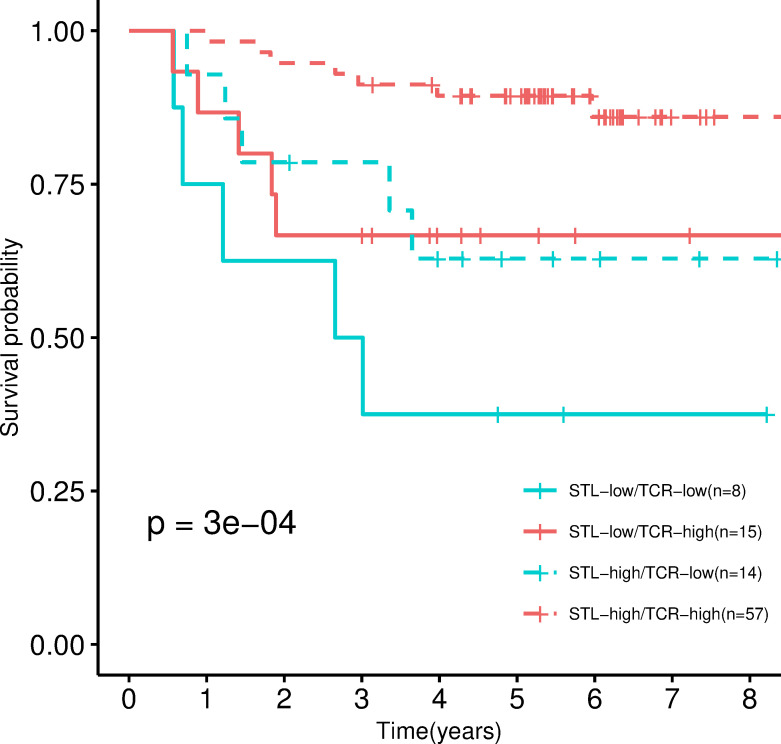
Combination of TCRs and lymphocytosis measures by immunosequencing and by a pathologist. Kaplan–Meier curve stratified into 4 categories resulting from the combinations of high/low TCR measured by immunoSEQ and high/low STL measured by the pathologist. *P* values were calculated by fitting a Cox regression model adjusted by sex, age, tumor location and stage (IIA, IIB). STL, stromal lymphocyte; TCR, T-cell receptor.

Regarding the correlation between the pathologist measurements and the immunosequencing of TCR, no significant correlations were found (S2 Fig). This may be related to the relatively small sample size but also to the fact that lymphocytes measured in HE fixed slides included T cells (both CD4 and CD8 staining) but also B cells (CD20 staining) and plasma cells (CD138 staining). Also, immuneSEQ captured stroma and tumor combined TCRs. Therefore, we evaluated the hypothesis that the combination of these 2 independent measures, TCR sequencing and pathologist assessment of STL proportion, may confer better prognostic information, and we stratified samples into 4 categories (TCR-high/STL-high, TCR-high/STL-low, TCR-low/STL-high, and TCR-low/STL-low). The group in TCR-low/STL-low showed a worse prognosis in comparison with the TCR-high/STL-high group (HR = 0.1, 95% CI 0.03–0.36, *P* = 0.0003, Fig 4).

#### Functional enrichment

The ICO/CLX discovery series had available gene expression data, which were used to perform a functional study in order to decipher biological insights underlying T-cell infiltration. Genes were ranked according to the correlation between their level of expression in the tumor and number of T cells measured by immunosequencing. As expected, functions related with immune response appeared as the most statistically significantly enriched among tumors with high T-cell abundance by immunoSEQ, such as *T cell activation*, *immunoregulatory interactions between lymphoid and nonlymphoid cells*, *PD1 signaling*, or *intestinal immune network*, among others (S1 Table and S3 Fig). Interestingly, a significant correlation was found between T-cell abundance and *CD8A* expression levels, supporting the utility of this gene as a surrogate of lymphocyte infiltration (S4 Fig).

### Assessment of TCR abundance and clonality index prognostic value in independent datasets

#### Replication in FF samples

**Fig 5:**
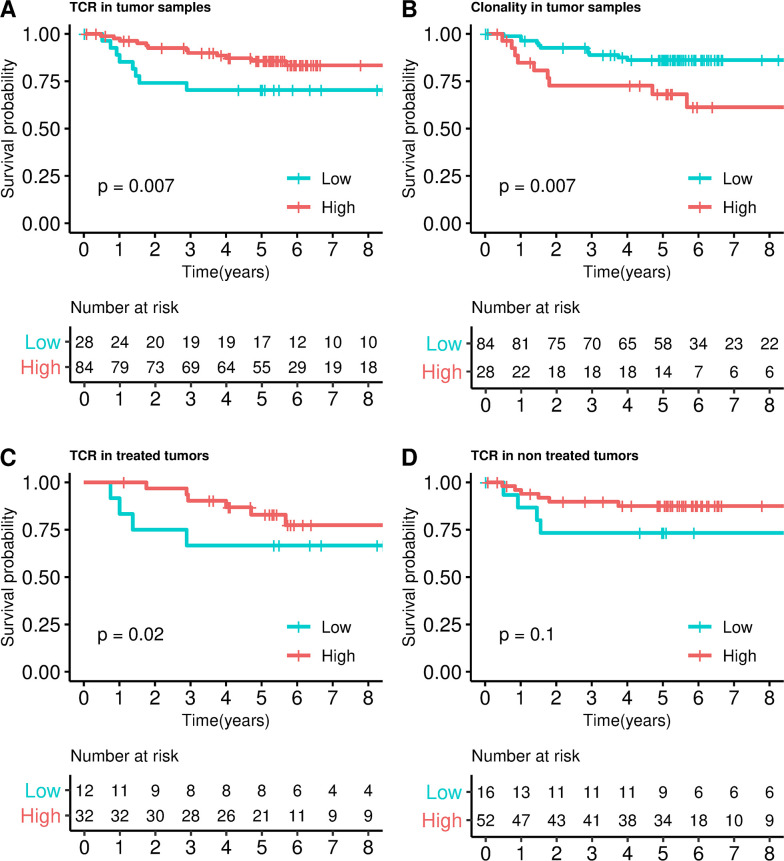
Survival analysis in ICO/FF dataset. Kaplan–Meier curves dividing into high and low TCRs categories in tumor samples (**A**), into high and low clonality categories (**B**); and into high and low TCRs in treated (**C**) and nontreated samples (**D**). *P* values were calculated by fitting a Cox regression model adjusted by sex, age, and stage (IIA, IIB). The same trend was observed in TCR (HR = 0.68, 95% CI 0.51–0.91, *P* = 0.01) and clonality index (HR = 1.56, 95% CI 0.87–2.80, *P* = 0.13) when continuous variables were used. CI, confidence interval; FF, fresh frozen; HR, hazard ratio; ICO, Catalan Institute of Oncology; TCR, T-cell receptor.

The prognostic value of TCRs in tumors using the same immunosequencing technique was performed in several independent series. First, an extended dataset of 112 FF stage II samples comprising both treated and nontreated patients was analyzed (ICO/FF). The cutoff used was the first quartile (almost the same value that the one in the ICO CLX discovery dataset, see Table 1). As a result, a clear association with prognosis was found (Fig 5A), in which those tumors with higher T-cell abundance demonstrates better survival (HR = 0.30, 95% CI 0.12–0.72, *P* = 0.007). Clonality index was associated with prognosis in this series (HR = 3.32, 95% CI 1.38–7.94, *P* = 0.007, Fig 5B). When patients were analyzed separately according to adjuvant chemotherapy (44 treated and 68 nontreated), treated patients had better prognosis for high T-cell abundance (HR = 0.22, 95% CI 0.06–0.79, *P* = 0.02, Fig 5C). However, although nontreated patients showed a tendency to have better prognosis for high T-cell abundance, this was nonsignificant (HR = 0.34, 95% CI 0.09–1.27, *P* = 0.1, Fig 5D).

#### Replication in formalin-fixed paraffin-embedded (FFPE) samples

**Fig 6:**
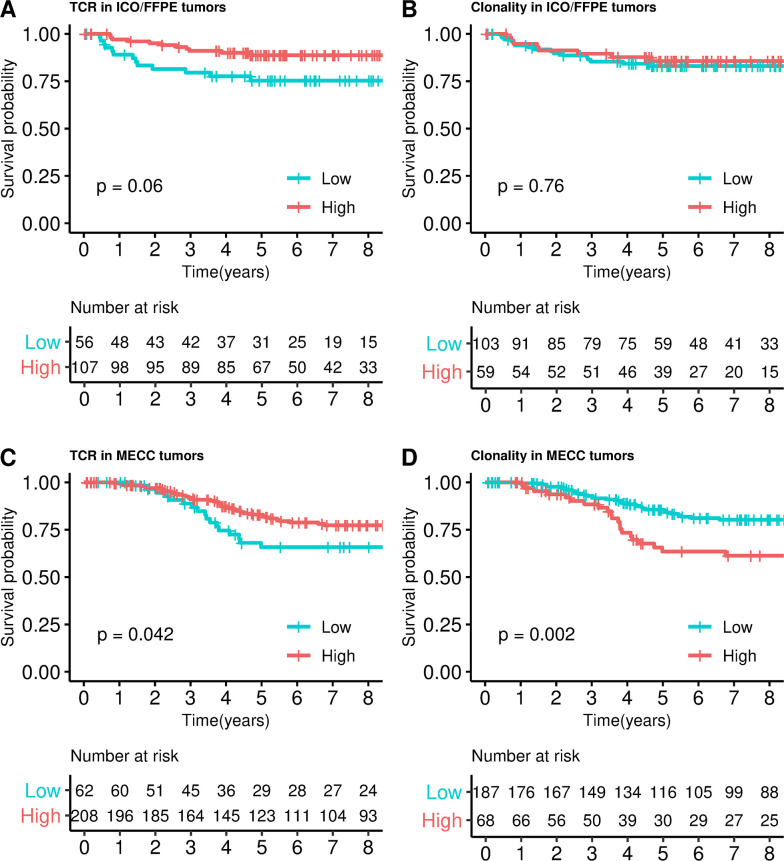
Survival analysis in ICO/FFPE and MECC datasets. Kaplan–Meier curves dividing into high and low TCRs categories in ICO/FFPE dataset (**A**), MECC dataset (**B**). Kaplan–Meier curves dividing into high and low clonality categories in ICO/FFPE dataset (**C**) and MECC dataset (**D**). *P* values were calculated by fitting a Cox regression model adjusted by sex, age, tumor location, stage (IIA, IIB), and MSI status. FFPE, formalin-fixed paraffin-embedded; ICO, Catalan Institute of Oncology; MECC, Molecular Epidemiology of Colorectal Cancer study; TCR, T-cell receptor.

Next, 163 FFPE samples were analyzed (ICO/FFPE). This dataset was similar to the discovery because all tumors were obtained from patients diagnosed in stage II that had not been treated with adjuvant chemotherapy. This series initially included 20 (12%) MSI tumors. The same trend of association with prognosis was observed (HR = 0.41, 95% CI 0.18–0.93, *P* = 0.03, Fig 6A) when TCRs were stratified in high and low categories. The first quartile was used to calculate a new cutoff for this dataset (TCR > 0.10) because TCRs measured in FFPE used a different standardization technique that relied on housekeeping genes to provide a result independent of DNA degradation, but the distribution of TCR values was different from that measured in FF samples. The prognostic association was retained when MSI tumors were excluded from the analysis (HR = 0.40, 95% CI 0.16–0.98, *P* = 0.04). Clonality index was not associated with prognosis in this series (HR = 0.71, 95% CI 0.30–1.68, *P* = 0.44, Fig 6B). However, when association with prognosis was assessed in a continuous manner, TCR was not significant (HR = 0.78, 95% CI 0.49–1.23, *P* = 0.28) neither clonality index (HR = 1.06, 95% CI 0.57–1.94, *P* = 0.86).

Finally, TCR immunosequencing was analyzed in a larger FFPE dataset from a different country (MECC dataset in Israel) comprising 270 stage II tumors (see Table 2). The cutoff was calculated as the first quartile (T-cell abundance > 0.06). TCR immunosequencing confirmed a significant prognostic association among the MSS, stage II (HR = 0.52, 95% CI 0.28–0.95, *P* = 0.03). A borderline significant prognostic association was confirmed in this dataset among all tumors, including those that were MSI (HR = 0.56, 95% CI 0.31–1.00, *P* = 0.042, Fig 6C). Clonality index was also associated with prognosis (HR = 2.45, 95% CI 1.39–4.32, *P* = 0.002, Fig 6D) even when MSI tumors were excluded from the analysis (HR = 2.58, 95% CI 1.41–4.71, *P* = 0.002). Not significant association was retained with the continuous variables TCR (HR = 0.87, 95% CI 0.69–1.10, *P* = 0.24) and clonality index (HR = 1.28, 95% CI 0.95–1.72, *P* = 0.10).

Details for all these adjusted models are shown in S2 Table.

### Combined analysis of TCR abundance and clonality index in MSS stage II tumors

**Fig 7:**
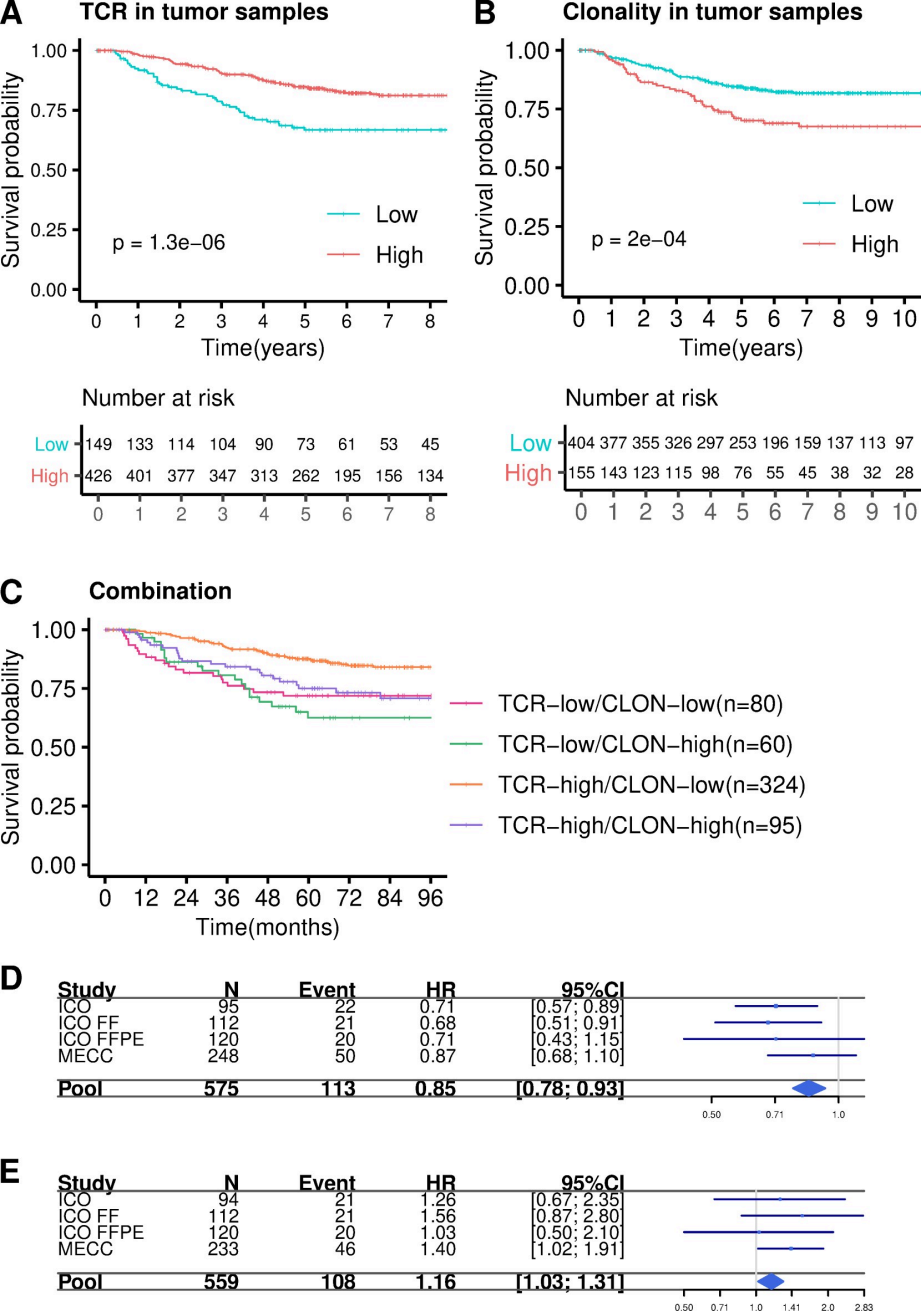
Cox model stratified by studies. Kaplan–Meier curves dividing into high and low TCRs (**A**) and high and low clonality (**B**) categories in all datasets. **C.** Kaplan–Meier curve categorizing into four categories high TCRs -high clonality, high TCRs -low clonality, low TCRs -high clonality, low TCRs -low clonality. *P* values were calculated by fitting a Cox regression model adjusted by sex, age, tumor location and stage (IIA, IIB). **D**. Forest plot for the TCR abundance combined analysis **E**. Forest plot for the clonality index combined analysis. CI, confidence interval; CLON, clonality index; FF, fresh frozen; FFPE, formalin-fixed paraffin-embedded; HR, hazard ratio; ICO, Catalan Institute of Oncology; MECC, Molecular Epidemiology of Colorectal Cancer study; TCR, T-cell receptor.

Finally, a stratified Cox model was adjusted looking for prognostic value of TCR in all datasets combined (*n* = 575 after excluding MSI tumors in datasets in which this information was available). As a result, a clear association between higher levels of relative T-cell abundance and better prognosis in MSS stage II tumors was found (HR = 0.39, 95% CI 0.26–0.57, *P* = 3e-06, Fig 7A). Also, a significant association was observed with clonality index (HR = 2.13, 95% CI 1.44–3.15, *P* = 0.0002, Fig 7B). Moreover, the relationship between T-cell abundance and clonality index was explored. The models that included both variables as quantitative showed that the association with prognosis was stronger for TCR abundance than clonality index, which was no longer significant in the fully adjusted model. This probably was related to the correlation between the variables (partial correlation = −0.13). The model with categorical variables showed, however, a strong association for both metrics: (HR = 0.47, 95% CI 0.32–0.71, *P* = 0.0003 for high TCR abundance and HR = 1.79, 95% CI 1.20–2.69, *P* = 0.004 for high clonality index). Details for these adjusted models are shown in S2 Table. The stratified analysis of all stage II patients by the combination of TCR abundance and clonality in the tumor showed that patients in the “high abundance/low clonality index” group had the better prognosis when comparing with the other groups (HR = 0.38, 95% CI 0.22–0.65, *P* = 0.0004, Fig 7C).

Interestingly, when only stage II-B tumors (*n* = 49) were taken into account, TCR abundance was also significantly associated with prognosis (HR = 0.11, 95% CI 0.02–0.59, *P* = 0.01, S5A Fig), whereas clonality was not (HR = 1.66, 95% CI 0.45–6.10, *P* = 0.44, S5B Fig). However, the combined analysis in the II-B setting also retained the “high abundance/low clonality” group as the one showing the better prognosis (HR = 0.13, 95% CI 0.03–0.34, *P* = 0.0038, S5C Fig).

The combined analysis of MSS tumors interrogating TCR abundance and clonality index as continuous variables also confirmed the results (HR = 0.85, 95% CI 0.78–0.93, *P* = 0.002 for TCR abundance, Fig 7D; and HR = 1.16, 95% CI 1.03–1.31, *P* = 0.016 for clonality, Fig 7E).

## Discussion

Results from this study demonstrate that tumor lymphocytes, assessed by TCR repertoire quantification based on a sequencing method, are an independent prognostic factor in microsatellite stable stage II CRC. Patients with tumors exhibiting a higher abundance of T cells in the tumor microenvironment had a better prognosis. This result has been replicated in more than 600 tumor samples from 2 countries. A similar association has been previously reported in tumors other than CRC, including breast [23], ovarian [24], esophageal squamous cell carcinoma [25], head and neck squamous cell carcinoma [26], lung [27] or gastric cancer [28], among others.

Immunosequencing also provides information about T-cell clonality [29]. Although not as robust as data on abundance, our results demonstrated that patients with lower clonality showed overall better prognosis. This suggests that the more polyclonal the T-cell population, the better the outcome. The higher the clonality the better the prognosis, because tumors harboring a smaller number of clones are usually less aggressive. However, in agreement with our results, a narrow TCR repertoire has been associated with and adverse outcome in lymphoma [30]. Also, a diverse TCR repertoire in blood is associated with a better prognosis in CRC [31]. Moreover, combined analysis of all datasets showed better prognosis in patients with both highly infiltrative and diverse intratumoral T-cell populations. Thus, immunosequencing provides us with 2 metrics allowing to detect patients at more risk of recurrence in a more accurate way.

Adjuvant chemotherapy offers a limited improvement in survival within stage II CRC, although clinical and molecular features can guide the appropriate application of this clinical approach. Several molecular factors have been investigated as prognostic biomarkers in stage II CRC, and only MSI phenotype has been adopted in routine clinical practice [32]. Our study has identified that low levels of T-cell abundance defines a group of patients with poor prognosis, even though they were diagnosed at early stage microsatellite stable CRC. Of note, TCR abundance is also useful to identify stage II-B tumors exhibiting good prognosis. Because stage II-B patients are routinely treated with chemotherapy, TCR abundance may be useful identifying a group that need not be treated. It is worthwhile to note that there are also patients who relapse despite exhibiting a high abundance of TCR, possibly by acquisition of aberrant immune-phenotypic traits [33].

The lymphocytic reaction and its implication in prognosis have been largely studied in MSI tumors [34]. MSI phenotype is strongly associated with a defective mismatch repair (MMR) system, and as a consequence, these tumors accumulate an elevated number of point mutations [35]. Thus, it has been proposed that the higher level of neoantigens and TILs in MSI tumors may contribute to better patient survival [36]. Interestingly, here we have shown that microsatellite stable tumors with high TCR abundance also have a better prognosis, probably as a consequence of their ability to generate an immune response.

Taking advantage of gene expression data, a functional analysis ranked genes according to their correlation with TCRs abundance was performed. As expected, high enrichment in pathways and functions related to immune response (and specifically with T-cell antitumor activity) emerged, such as lymphocyte activation or TCR signaling. Functions like immunoregulatory interactions between a lymphoid and a nonlymphoid cell suggested active crosstalk between the tumor cell and their surrounding microenvironment. Interestingly, the well-described PD1 pathway that is a known immunotherapy target in advanced disease appears as highly significant in our early stage dataset, too. Furthermore, we observed that the interferon alpha and gamma pathway was one of the most enriched. It is likely that interferon regulates the adaptive immune response in this setting because it has been reported that interferon-inducible chemokines are significantly correlated with the presence of T cells in colon tumors and have a protective and antimetastatic role in CRC [37].

Because we extracted DNA from an enriched tumor area but not microdissected, individually captured TILs, TCR measurement by ultra-sequencing technology did not discriminate between epithelial and STLs. To explore this issue, an independent measure of both cell populations was done by a pathologist in the same cancer samples. Consistent with prior results showing a significant prognostic advantage for TILS independent of stage and MSI [15], we found that intraepithelial TILs, as measured by pathologists in stage II, MSS colorectal cancers are weakly associated with improved prognosis. We postulate that differences in the briskness of the host response in MSS and MSI tumors may partially explain the differences in the strength of the association, although differences in sample size may also contribute. STLs were identified in our study as a prognostic biomarker. This interesting result reinforces the importance of the microenvironment in CRC carcinogenesis. Of note, lymphocytosis measured by TCR immunosequencing performed as well or better as a prognostic biomarker than lymphocytosis measured by a pathologist. In our data, both intraepithelial and intrastroma cells conferred some prognostic information that was simultaneously captured by TCR immunosequencing. In the same vein, we have not found a relationship between the proportion of stroma in a sample and prognosis, suggesting that is the composition and level of activation of the host response that informs prognosis [38]. Moreover, STLs and TCRs measurements were only modestly correlated in our data. Because both measures showed a clear association with prognosis, in our data, this suggests the possibility of an independent but cooperative role against tumor growth. Consistent with this hypothesis, it has been reported that CD20+ and CD8+ tumor-infiltrating lymphocytes work together to mediate antitumor immunity in ovarian cancer. Indeed, CD20+ TILs might act as antigen-presenting cells, as lymphoid organizers, and as polarizing cells, thus promoting potent T-cell responses [39,40].

This study has several limitations. Though 4 independent studies have been analyzed, all were based on retrospective samples already collected with long follow-up. Most of the samples were very old, and some from the studies using material preserved in FFPE had to be excluded because sequencing quality was insufficient. Also, the sequencing technique used different calibration standards for FF and FFPE, which required to use different cutoffs for each preservation method. The retrospective design also precluded obtaining information on treatments or DFS in the MECC study. The studies ICO/FF and ICO/FFPE had no information on the pathology assessment, and the MECC study only had assessed TILs. That prevented to replicate the finding of the discovery series regarding the stronger prognosis value of STLs infiltration than TILs. Finally, the ICO/FF study had no data on microsatellite status, and 10% of the cases are expected to be MSI.

In summary, we propose that tumor lymphocyte assessment by TCR immunosequencing technique, which combines information about abundance and clonality, is an independent prognostic biomarker in stage II MSS tumors. These results should be validated in prospective studies to prove their clinical utility.

## Supporting information

S1 STROBE checklist STROBE, Strengthening the Reporting of Observational Studies in Epidemiology. (DOC)

S1 Table GSEA results. GSEA, Gene Set Enrichment Analysis.
(PDF)

S2 Table Adjusted models. (DOCX)

S1 Fig Boxplots showing differences between 96 adjacent normal and 96 tumor samples in: percentage of TCR (**A**). TCR repertoire clonality **(B)**. Percentage of most frequent single clone (**C**); in the discovery ICO/CLX dataset. CLX, Colonomics study; ICO, Catalan Institute of Oncology; TCR, T-cell receptor.
(TIF)

S2 Fig Correlation of TCRs immunosequencing-measured with stromal and TIL infiltration measured by a pathologist. TCR, T-cell receptor; TIL, tumor-infiltrating lymphocyte.
(TIF)

S3 Fig GSEA plots showing examples of significant pathways and functions in which tumors with high TCR are enriched. GSEA, Gene Set Enrichment Analysis; TCR, T-cell receptor.
(TIF)

S4 Fig Correlation between TCRs immunosequencing-measured and *CD8A* gene expression. TCR, T-cell receptor.
(TIF)

S5 Fig Kaplan–Meier curves dividing into high and low TCRs (**A**) and high and low clonality (**B**) categories in stage II-B tumors. **C.** Kaplan–Meier curve categorizing into 3 categories: high TCRs–high clonality, high TCRs–low clonality, low TCRs–high clonality, and low TCRs–low clonality. TCR, T-cell receptor.
(TIF)

## Abbreviations

CI: confidence interval
CLR: Crohn’s-like Lymphoid Reaction
CLX: Colonomics study
CRC: colorectal cancer
DFS: disease-free survival
FF: fresh frozen
HE: hematoxylin and eosin
HR: hazard ratio
ICO: Catalan Institute of Oncology
MECC: Molecular Epidemiology of Colorectal Cancer study
MHC: major histocompatibility complex
MMR: mismatch repair
MSI: microsatellite instability
MSS: microsatellite stable
STL: stromal lymphocyte
TCR: T-cell receptor
TIL: tumor-infiltrating lymphocyte
