# Supplementary material for: Lymphocytic infiltration in stage II microsatellite stable colorectal tumors: A retrospective prognosis biomarker analysis
Source: PLoS Med. 2020 Sep 24;17(9):e1003292. doi: 10.1371/journal.pmed.1003292 (PMC7514069; doi:10.1371/journal.pmed.1003292)
Supplement: S1 Table — GSEA, Gene Set Enrichment Analysis. (PDF) [file pmed.1003292.s002.pdf]

NAME	SIZE	ES	NES	NOM p-val	FDR q-val	FWER p-val
**HALLMARK_ALLOGRAFT_REJECTION**	200	0.65954506	30.604.916	0.0	0.0	0.0
**HALLMARK_INTERFERON_GAMMA_RESPONSE**	198	0.6578051	30.451.725	0.0	0.0	0.0
**HALLMARK_EPITHELIAL_MESENCHYMAL_TRANSITION**	198	0.6402159	29.477.346	0.0	0.0	0.0
**HALLMARK_INTERFERON_ALPHA_RESPONSE**	94	0.6188131	26.063.433	0.0	0.0	0.0
**HALLMARK_INFLAMMATORY_RESPONSE**	199	0.55809546	25.854.888	0.0	0.0	0.0
**HALLMARK_IL6_JAK_STAT3_SIGNALING**	87	0.5835411	23.987.734	0.0	0.0	0.0
**HALLMARK_COMPLEMENT**	196	0.51860523	2.398.198	0.0	0.0	0.0
**HALLMARK_TNFA_SIGNALING_VIA_NFKB**	199	0.48753825	22.684.073	0.0	0.0	0.0
**HALLMARK_IL2_STAT5_SIGNALING**	199	0.47705644	22.233.922	0.0	0.0	0.0
**HALLMARK_MYOGENESIS**	200	0.4687525	21.808.493	0.0	0.0	0.0
**HALLMARK_KRAS_SIGNALING_UP**	199	0.4688729	21.723.757	0.0	0.0	0.0
HALLMARK_COAGULATION	136	0.44935197	19.731.516	0.0	5,70E+02	0.001
HALLMARK_UV_RESPONSE_DN	144	0.40001053	17.808.143	0.0	7,61E+02	0.014
HALLMARK_APICAL_JUNCTION	200	0.3812462	1.766.853	0.0	8,09E+02	0.016
HALLMARK_ANGIOGENESIS	36	0.47336802	16.311.126	0.015673982	0.0042660446	0.082
HALLMARK_APICAL_SURFACE	44	0.39148498	1.447.745	0.038772214	0.025452888	0.429
HALLMARK_APOPTOSIS	160	0.31525907	14.212.136	0.017711172	0.031019775	0.51

KEGG NAME	SIZE	ES	NES	NOM p-val	FDR q-val	FWER p-val
**KEGG_HEMATOPOIETIC_CELL_LINEAGE**	86	0.66786736	27.774	0.0	0.0	0.0
**KEGG_INTESTINAL_IMMUNE_NETWORK_FOR_IGA_PRODUCTION**	47	0.71539855	265.024	0.0	0.0	0.0
**KEGG_CYTOKINE_CYTOKINE_RECEPTOR_INTERACTION**	259	0.5432273	2.598.793	0.0	0.0	0.0
**KEGG_CELL_ADHESION_MOLECULES_CAMS**	132	0.5829304	25.668.385	0.0	0.0	0.0
**KEGG_PRIMARY_IMMUNODEFICIENCY**	35	0.7429938	25.488.887	0.0	0.0	0.0
**KEGG_CHEMOKINE_SIGNALING_PATHWAY**	187	0.53537685	24.813.821	0.0	0.0	0.0
**KEGG_ALLOGRAFT_REJECTION**	36	0.72263795	24.768.922	0.0	0.0	0.0
**KEGG_T_CELL_RECEPTOR_SIGNALING_PATHWAY**	107	0.56864935	24.349.632	0.0	0.0	0.0
**KEGG_AUTOIMMUNE_THYROID_DISEASE**	51	0.64746433	24.240.677	0.0	0.0	0.0
**KEGG_GRAFT_VERSUS_HOST_DISEASE**	38	0.68133885	24.037.645	0.0	0.0	0.0
**KEGG_B_CELL_RECEPTOR_SIGNALING_PATHWAY**	74	0.576736	23.310.935	0.0	0.0	0.0
**KEGG_ANTIGEN_PROCESSING_AND_PRESENTATION**	81	0.56828386	2.323.671	0.0	0.0	0.0
**KEGG_LEISHMANIA_INFECTION**	71	0.59025836	23.190.527	0.0	0.0	0.0
**KEGG_NATURAL_KILLER_CELL_MEDIATED_CYTOTOXICITY**	131	0.52912164	23.166.835	0.0	0.0	0.0
**KEGG_ASTHMA**	29	0.69339174	22.889.016	0.0	0.0	0.0
**KEGG_SYSTEMIC_LUPUS_ERYTHEMATOSUS**	134	0.52095383	228.618	0.0	0.0	0.0
**KEGG_VIRAL_MYOCARDITIS**	69	0.5742115	22.826.939	0.0	0.0	0.0
**KEGG_ECM_RECEPTOR_INTERACTION**	84	0.5282363	2.139.758	0.0	0.0	0.0
KEGG_FOCAL_ADHESION	199	0.44403663	20.619.285	0.0	9,69E+01	0.002
KEGG_LEUKOCYTE_TRANSENDOTHELIAL_MIGRATION	116	0.47120455	20.403.166	0.0	9,21E+02	0.002
KEGG_COMPLEMENT_AND_COAGULATION_CASCADES	69	0.51670015	20.353.947	0.0	8,77E+01	0.002
KEGG_JAK_STAT_SIGNALING_PATHWAY	151	0.4485636	20.241.401	0.0	1,70E+03	0.004
KEGG_VASCULAR_SMOOTH_MUSCLE_CONTRACTION	115	0.46458596	20.040.631	0.0	1,62E+03	0.004
KEGG_TYPE_I_DIABETES_MELLITUS	42	0.554291	19.636.563	0.0	4,34E+03	0.011
KEGG_TOLL_LIKE_RECEPTOR_SIGNALING_PATHWAY	102	0.44321477	18.584.768	0.0	0.002220317	0.058
KEGG_CALCIUM_SIGNALING_PATHWAY	176	0.4015889	18.292.779	0.0	0.0034022294	0.091
KEGG_FC_EPSILON_RI_SIGNALING_PATHWAY	79	0.4564137	18.170.536	0.0	0.0040173694	0.111
KEGG_GLYCOSAMINOGLYCAN_BIOSYNTHESIS_CHONDROITIN_SULFATE	22	0.5732019	17.831.968	0.0	0.0059759417	0.163
KEGG_LYSOSOME	119	0.4066158	17.654.738	0.0	0.0071565984	0.197
KEGG_NEUROACTIVE_LIGAND_RECEPTOR_INTERACTION	270	0.36544958	17.496.089	0.0	0.008358413	0.235
KEGG_DILATED_CARDIOMYOPATHY	90	0.41267177	17.196.529	0.0014347202	0.01171304	0.323
KEGG_FC_GAMMA_R_MEDIATED_PHAGOCYTOSIS	96	0.40951335	17.078.953	0.0	0.01274488	0.35
KEGG_NOD_LIKE_RECEPTOR_SIGNALING_PATHWAY	62	0.4301662	16.796.834	0.012539185	0.017158898	0.455
KEGG_CYTOSOLIC_DNA_SENSING_PATHWAY	55	0.4406829	16.688.124	0.00625	0.018578207	0.491
KEGG_REGULATION_OF_ACTIN_CYTOSKELETON	212	0.34762174	16.237.423	0.0	0.027409848	0.644
KEGG_GAP_JUNCTION	90	0.38184446	15.741.663	0.011594203	0.040126983	0.786
KEGG_ALDOSTERONE_REGULATED_SODIUM_REABSORPTION	42	0.433548	15.723.605	0.018897638	0.039587628	0.788
KEGG_APOPTOSIS	86	0.37838218	156.782	0.007173601	0.040151045	0.803

REACTOME NAME	SIZE	ES	NES	NOM p-val	FDR q-val	FWER p-val
**REACTOME_IMMUNOREGULATORY_INTERACTIONS_BETWEEN_A_LYMPHOID_AND_A_NON_LYMPHOID_CELL**	61	0.718359	28.367.887	0.0	0.0	0.0
**REACTOME_INTERFERON_GAMMA_SIGNALING**	59	0.708568	2.725.301	0.0	0.0	0.0
**REACTOME_GENERATION_OF_SECOND_MESSENGER_MOLECULES**	26	0.81348026	2.609.139	0.0	0.0	0.0
**REACTOME_CHEMOKINE_RECEPTORS_BIND_CHEMOKINES**	51	0.70387423	26.048.827	0.0	0.0	0.0
**REACTOME_TCR_SIGNALING**	51	0.6732419	25.200.708	0.0	0.0	0.0
**REACTOME_INTERFERON_ALPHA_BETA_SIGNALING**	62	0.60343117	23.593.645	0.0	0.0	0.0
**REACTOME_G_ALPHA_I_SIGNALLING_EVENTS**	189	0.5083344	23.456.109	0.0	0.0	0.0
**REACTOME_PD1_SIGNALING**	17	0.81113756	2.341.908	0.0	0.0	0.0
**REACTOME_PHOSPHORYLATION_OF_CD3_AND_TCR_ZETA_CHAINS**	15	0.8345406	23.310.616	0.0	0.0	0.0
**REACTOME_COLLAGEN_FORMATION**	56	0.60145164	23.046.818	0.0	0.0	0.0
**REACTOME_CLASS_A1_RHODOPSIN_LIKE_RECEPTORS**	288	0.47811848	23.013.976	0.0	0.0	0.0
**REACTOME_EXTRACELLULAR_MATRIX_ORGANIZATION**	84	0.55225116	22.808.762	0.0	0.0	0.0
REACTOME_ANTIGEN_ACTIVATES_B_CELL_RECEPTOR_LEADING_TO_GENERATION_OF_SECOND_MESSENGERS	29	0.61899626	2.067.539	0.0	7,31E+01	0.019
REACTOME_COMPLEMENT_CASCADE	29	0.6228899	2.089.955	0.0	5,28E+02	0.013
REACTOME_INTEGRIN_CELL_SURFACE_INTERACTIONS	79	0.509152	20.591.643	0.0	7,31E+02	0.02
REACTOME_PEPTIDE_LIGAND_BINDING_RECEPTORS	176	0.4703581	21.652.117	0.0	2,32E+03	0.004
REACTOME_GPCR_LIGAND_BINDING	389	0.43431646	21.509.857	0.0	2,64E+03	0.005
REACTOME_DOWNSTREAM_TCR_SIGNALING	34	0.6212139	21.192.684	0.0	3,64E+03	0.008
REACTOME_COSTIMULATION_BY_THE_CD28_FAMILY	61	0.5445732	21.074.307	0.0	3,86E+03	0.009
REACTOME_SMOOTH_MUSCLE_CONTRACTION	23	0.6697147	21.297.133	0.0	3,88E+03	0.008
REACTOME_INTERFERON_SIGNALING	153	0.4565251	20.451.531	0.0	8,01E+03	0.023
REACTOME_CELL_SURFACE_INTERACTIONS_AT_THE_VASCULAR_WALL	84	0.50357604	20.338.137	0.0	0.00102909	0.031
REACTOME_PLATELET_ACTIVATION_SIGNALING_AND_AGGREGATION	198	0.43668813	20.184.703	0.0	0.0012982601	0.041
REACTOME_THE_ROLE_OF_NEF_IN_HIV1_REPLICATION_AND_DISEASE_PATHOGENESIS	27	0.6156036	20.063.546	0.0	0.0015156454	0.05
REACTOME_CYTOKINE_SIGNALING_IN_IMMUNE_SYSTEM	263	0.41851756	198.909	0.0	0.0019180672	0.065
REACTOME_SIGNALING_BY_RHO_GTPASES	111	0.4626563	19.772.524	0.0	0.0019777652	0.075
REACTOME_CHONDROITIN_SULFATE_BIOSYNTHESIS	19	0.68106335	19.845.308	0.0017006802	0.0019855907	0.07
REACTOME_NITRIC_OXIDE_STIMULATES_GUANYLATE_CYCLASE	25	0.6172129	19.800.271	0.0016750419	0.0020222228	0.074
REACTOME_RNA_POL_I_PROMOTER_OPENING	59	0.5135674	19.665.416	0.0	0.0021866225	0.085
REACTOME_OTHER_SEMAPHORIN_INTERACTIONS	15	0.6994511	19.605.584	0.0	0.0023295432	0.094
REACTOME_NUCLEOTIDE_LIKE_PURINERGIC_RECEPTORS	16	0.6851306	19.570.564	0.0	0.0023715028	0.098
REACTOME_GPVI_MEDIATED_ACTIVATION_CASCADE	31	0.5762875	19.518.225	0.001633987	0.0025471437	0.108
REACTOME_CHONDROITIN_SULFATE_DERMATAN_SULFATE_METABOLISM	47	0.5238685	19.327.179	0.0	0.0031272268	0.14
REACTOME_MUSCLE_CONTRACTION	46	0.5319804	19.334.052	0.0	0.0032003727	0.139
REACTOME_HEMOSTASIS	451	0.38376668	1.924.031	0.0	0.0033513163	0.152
REACTOME_RESPONSE_TO_ELEVATED_PLATELET_CYTOSOLIC_CA2_	81	0.46887857	19.206.111	0.0	0.003377139	0.158
REACTOME_INNATE_IMMUNE_SYSTEM	267	0.39893392	1.911.258	0.0	0.003736822	0.179
REACTOME_G_BETA_GAMMA_SIGNALLING_THROUGH_PI3KGAMMA	25	0.57070225	18.573.465	0.0	0.006912913	0.321
REACTOME_IL_3_5_AND_GM_CSF_SIGNALING	43	0.51435065	18.584.383	0.0031948881	0.0070198025	0.318
REACTOME_HDL_MEDIATED_LIPID_TRANSPORT	15	0.6648322	18.438.634	0.005357143	0.007846906	0.36
REACTOME_G_ALPHA_Z_SIGNALLING_EVENTS	44	0.5020078	1.834.757	0.0	0.008024695	0.379
REACTOME_G_ALPHA_S_SIGNALLING_EVENTS	120	0.42275086	18.364.906	0.0	0.008094908	0.377
REACTOME_CGMP_EFFECTS	19	0.61293036	18.061.836	0.0034843206	0.0105980355	0.479
REACTOME_G_PROTEIN_BETA_GAMMA_SIGNALLING	28	0.5526722	1.800.019	0.0	0.010938269	0.502
REACTOME_TOLL_RECEPTOR_CASCADES	113	0.41924983	17.954.698	0.0	0.011135153	0.518
REACTOME_PLATELET_HOMEOSTASIS	76	0.43823305	17.725.958	0.0	0.013720349	0.609
REACTOME_IL_RECEPTOR_SHC_SIGNALING	27	0.543565	17.737.088	0.0048	0.0138921635	0.604
REACTOME_LATENT_INFECTION_OF_HOMO_SAPIENS_WITH_MYCOBACTERIUM_TUBERCULOSIS	31	0.5260831	17.698.407	0.0050847456	0.013920494	0.62
REACTOME_SIGNALING_BY_ILS	106	0.41135067	17.592.843	0.0	0.0152868945	0.66
REACTOME_G_ALPHA_Q_SIGNALLING_EVENTS	179	0.38146967	17.374.412	0.0	0.018782137	0.745
REACTOME_SEMAPHORIN_INTERACTIONS	65	0.44010365	17.181.596	0.002994012	0.022126712	0.819
REACTOME_GLYCOSAMINOGLYCAN_METABOLISM	108	0.4023834	17.130.022	0.0	0.022734208	0.833
REACTOME_REGULATION_OF_IFNA_SIGNALING	24	0.5337077	17.080.265	0.010291595	0.023469387	0.845
REACTOME_DEGRADATION_OF_THE_EXTRACELLULAR_MATRIX	28	0.51815176	17.039.981	0.011784512	0.0239918	0.853
REACTOME_PACKAGING_OF_TELOMERE_ENDS	48	0.454819	16.880.409	0.0047095763	0.026797634	0.882
REACTOME_REGULATION_OF_WATER_BALANCE_BY_RENAL_AQUAPORINS	43	0.46910393	16.896.348	0.0	0.026918879	0.876
REACTOME_IL_2_SIGNALING	41	0.46615377	16.824.304	0.0032051282	0.027793564	0.898
REACTOME_INFLAMMASOMES	16	0.5771901	16.419.561	0.020905923	0.03923497	0.963
REACTOME_GASTRIN_CREB_SIGNALLING_PATHWAY_VIA_PKC_AND_MAPK	200	0.35438177	16.379.312	0.0	0.039970912	0.965
REACTOME_GROWTH_HORMONE_RECEPTOR_SIGNALING	24	0.52339494	16.145.476	0.026622295	0.0481014	0.983
REACTOME_A_TETRASACCHARIDE_LINKER_SEQUENCE_IS_REQUIRED_FOR_GAG_SYNTHESIS	25	0.5137633	16.119.252	0.024590164	0.04835096	0.985
REACTOME_AMYLOIDS	79	0.39912745	16.091.101	0.0043923864	0.048572548	0.985

BIOCARTA NAME	SIZE	ES	NES	NOM p-val	FDR q-val	FWER p-val
**BIOCARTA_CTLA4_PATHWAY**	19	0.85563767	25.617.323	0.0	0.0	0.0
**BIOCARTA_NO2IL12_PATHWAY**	17	0.84340125	24.515.026	0.0	0.0	0.0
**BIOCARTA_IL17_PATHWAY**	15	0.8579667	23.623.898	0.0	0.0	0.0
**BIOCARTA_TCR_PATHWAY**	44	0.6571596	23.576.927	0.0	0.0	0.0
**BIOCARTA_CSK_PATHWAY**	22	0.7381113	2.316.923	0.0	0.0	0.0
**BIOCARTA_NKT_PATHWAY**	29	0.70380306	23.032.966	0.0	0.0	0.0
**BIOCARTA_IL12_PATHWAY**	21	0.7348455	2.292.049	0.0	0.0	0.0
**BIOCARTA_COMP_PATHWAY**	19	0.74640065	22.537.692	0.0	0.0	0.0
BIOCARTA_LAIR_PATHWAY	17	0.72845685	2.081.567	0.0	9,35E+02	0.009
BIOCARTA_TH1TH2_PATHWAY	19	0.7516521	22.255.118	0.0	1,30E+01	0.001
BIOCARTA_TNFR2_PATHWAY	18	0.69820565	20.890.646	0.0017064846	0.0010197469	0.009
BIOCARTA_TOB1_PATHWAY	19	0.7046013	20.896.668	0.0	0.0011217215	0.009
BIOCARTA_IL7_PATHWAY	17	0.6927927	2.000.849	0.0	0.0019638815	0.024
BIOCARTA_IL22BP_PATHWAY	16	0.70446384	20.142.424	0.0	0.0019850458	0.021
BIOCARTA_CD40_PATHWAY	15	0.71567136	20.041.025	0.0	0.002024713	0.023
BIOCARTA_FCER1_PATHWAY	38	0.5478516	191.945	0.0	0.0054076253	0.07
BIOCARTA_DC_PATHWAY	22	0.61220187	19.038.188	0.0	0.005907146	0.081
BIOCARTA_BCR_PATHWAY	34	0.53356117	18.486.905	0.0017271157	0.0115060285	0.161
BIOCARTA_IL3_PATHWAY	15	0.641326	18.432.877	0.0070422534	0.01163277	0.177
BIOCARTA_IL2RB_PATHWAY	38	0.5240392	1.844.801	0.0	0.011848091	0.171
BIOCARTA_STATHMIN_PATHWAY	19	0.61326396	1.817.781	0.0034246575	0.014420791	0.223
BIOCARTA_NKCELLS_PATHWAY	20	0.59149075	1.782.945	0.0050847456	0.020287093	0.306
BIOCARTA_PDGF_PATHWAY	32	0.5164165	17.646.595	0.005	0.023555893	0.366
BIOCARTA_HDAC_PATHWAY	29	0.5287556	17.539.786	0.0082236845	0.024845824	0.394
BIOCARTA_TPO_PATHWAY	24	0.5426781	17.360.256	0.006779661	0.028799817	0.456
BIOCARTA_PAR1_PATHWAY	37	0.48905346	17.139.522	0.0080256825	0.033256434	0.517
BIOCARTA_IL10_PATHWAY	17	0.5967887	1.709.822	0.005586592	0.03353707	0.533
BIOCARTA_TALL1_PATHWAY	15	0.60002726	16.903.695	0.020373514	0.039202284	0.602
BIOCARTA_CCR5_PATHWAY	17	0.58936876	16.832.545	0.012635379	0.04040812	0.625
BIOCARTA_IL2_PATHWAY	22	0.5345735	16.701.863	0.013722127	0.044270087	0.665

GO Biological Process NAME	SIZE	ES	NES	NOM p-val	FDR q-val	FWER p-val
**IMMUNE_RESPONSE**	227	0.63228106	29.895.208	0.0	0.0	0.0
**IMMUNE_SYSTEM_PROCESS**	322	0.5936385	29.025.872	0.0	0.0	0.0
**DEFENSE_RESPONSE**	266	0.5660546	27.186.503	0.0	0.0	0.0
**LEUKOCYTE_ACTIVATION**	69	0.66658115	26.457.381	0.0	0.0	0.0
**CELL_ACTIVATION**	76	0.654515	263.988	0.0	0.0	0.0
**CELLULAR_DEFENSE_RESPONSE**	56	0.6912161	26.322.377	0.0	0.0	0.0
**T_CELL_ACTIVATION**	44	0.7188982	26.243.777	0.0	0.0	0.0
**LYMPHOCYTE_ACTIVATION**	61	0.6700502	25.836.303	0.0	0.0	0.0
**REGULATION_OF_IMMUNE_SYSTEM_PROCESS**	65	0.65376556	25.631.182	0.0	0.0	0.0
**POSITIVE_REGULATION_OF_IMMUNE_SYSTEM_PROCESS**	50	0.643112	2.420.448	0.0	0.0	0.0
**REGULATION_OF_IMMUNE_RESPONSE**	32	0.69299644	23.631.847	0.0	0.0	0.0
**ADAPTIVE_IMMUNE_RESPONSE**	25	0.7302427	23.234.255	0.0	0.0	0.0
**POSITIVE_REGULATION_OF_MULTICELLULAR_ORGANISMAL_PROCESS**	65	0.591635	23.187.006	0.0	0.0	0.0
**REGULATION_OF_MULTICELLULAR_ORGANISMAL_PROCESS**	148	0.5163901	2.318.663	0.0	0.0	0.0
**IMMUNE_EFFECTOR_PROCESS**	36	0.658192	22.951.581	0.0	0.0	0.0
**LOCOMOTORY_BEHAVIOR**	93	0.5487351	2.286.494	0.0	0.0	0.0
**INFLAMMATORY_RESPONSE**	129	0.5229412	2.279.535	0.0	0.0	0.0
**REGULATION_OF_LYMPHOCYTE_ACTIVATION**	35	0.6717722	22.696.078	0.0	0.0	0.0
**ADAPTIVE_IMMUNE_RESPONSE_GO_0002460**	24	0.7359212	22.598.512	0.0	0.0	0.0
REGULATION_OF_T_CELL_ACTIVATION	28	0.6672026	2.249.235	0.0	4,69E-01	0.001
RESPONSE_TO_WOUNDING	189	0.48526046	22.168.813	0.0	8,61E+01	0.002
POSITIVE_REGULATION_OF_LYMPHOCYTE_ACTIVATION	24	0.6564124	20.620.751	0.0	9,78E+01	0.034
POSITIVE_REGULATION_OF_IMMUNE_RESPONSE	28	0.68912363	22.431.366	0.0	4,46E+02	0.001
T_CELL_DIFFERENTIATION	15	0.7949263	2.196.726	0.0	8,24E+02	0.002
CELL_RECOGNITION	19	0.6838339	20.595.014	0.0016556291	9,49E+02	0.035
ACTIVATION_OF_IMMUNE_RESPONSE	17	0.7233281	20.598.605	0.0	9,77E+02	0.035
CATION_HOMEOSTASIS	109	0.5024654	21.780.257	0.0	1,15E+03	0.003
RESPONSE_TO_OTHER_ORGANISM	79	0.5387688	21.734.948	0.0	1,45E+03	0.004
RESPONSE_TO_EXTERNAL_STIMULUS	309	0.4385982	21.423.945	0.0	2,01E+03	0.006
RESPONSE_TO_VIRUS	46	0.5854942	21.512.542	0.0	2,09E+03	0.006
RESPONSE_TO_BIOTIC_STIMULUS	115	0.4880977	21.218.016	0.0	4,55E+03	0.014
POSITIVE_REGULATION_OF_CYTOKINE_BIOSYNTHETIC_PROCESS	25	0.6624339	21.162.274	0.0016638935	4,71E+03	0.015
CYTOKINE_PRODUCTION	73	0.525356	21.035.068	0.0	5,00E+03	0.017
BEHAVIOR	150	0.47117147	21.079.905	0.0	5,17E+03	0.017
MULTI_ORGANISM_PROCESS	160	0.45849475	20.581.217	0.0	9,22E+03	0.035
HUMORAL_IMMUNE_RESPONSE	32	0.6017262	20.504.506	0.0	9,49E+03	0.037
CELLULAR_CATION_HOMEOSTASIS	106	0.5064053	21.843.467	0.0	1,20E+04	0.003
POSITIVE_REGULATION_OF_RESPONSE_TO_STIMULUS	40	0.57164496	20.310.771	0.0	0.0011214707	0.046
REGULATION_OF_RESPONSE_TO_STIMULUS	57	0.53111786	20.327.895	0.0	0.0011269992	0.045
HEMOPOIETIC_OR_LYMPHOID_ORGAN_DEVELOPMENT	75	0.4953065	20.119.607	0.0	0.0013229639	0.057
POSITIVE_REGULATION_OF_T_CELL_ACTIVATION	21	0.6556149	20.147.245	0.005376344	0.0013323537	0.056
HEMOPOIESIS	73	0.5006063	20.033.736	0.0	0.0014275302	0.062
MESODERM_DEVELOPMENT	22	0.64327556	19.928.257	0.0017574693	0.001512717	0.068
IMMUNE_SYSTEM_DEVELOPMENT	79	0.49045104	1.997.399	0.0	0.0015478964	0.068
SECOND_MESSENGER_MEDIATED_SIGNALING	153	0.44405448	19.834.019	0.0	0.0015629396	0.072
ION_HOMEOSTASIS	129	0.457172	19.667.604	0.0	0.001817955	0.085
CHEMICAL_HOMEOSTASIS	155	0.43853208	19.635.936	0.0	0.001961744	0.094
REGULATION_OF_CYTOKINE_BIOSYNTHETIC_PROCESS	38	0.56306064	19.579.924	0.0	0.0021595655	0.102
LYMPHOCYTE_DIFFERENTIATION	26	0.60034925	19.129.399	0.0	0.0036242658	0.167
SKELETAL_DEVELOPMENT	102	0.45102438	18.995.289	0.0	0.00417938	0.191
RAS_PROTEIN_SIGNAL_TRANSDUCTION	66	0.48192683	1.888.891	0.0	0.0045265057	0.209
HOMOPHILIC_CELL_ADHESION	16	0.67458194	18.819.011	0.0017574693	0.004837112	0.228
G_PROTEIN_COUPLED_RECEPTOR_PROTEIN_SIGNALING_PATHWAY	335	0.38579676	18.765.795	0.0	0.004975934	0.241
POSITIVE_REGULATION_OF_TRANSLATION	34	0.54947275	18.781.819	0.0	0.0049969843	0.238
B_CELL_ACTIVATION	20	0.62289953	18.725.072	0.0017421603	0.0051109516	0.251
PHAGOCYTOSIS	17	0.65807605	18.663.064	0.0017391305	0.0053906473	0.265
MUSCLE_DEVELOPMENT	93	0.44659063	18.556.085	0.0	0.0059711765	0.299
CYTOKINE_BIOSYNTHETIC_PROCESS	41	0.5201756	18.572.593	0.0031152647	0.0059751067	0.295
CELL_SUBSTRATE_ADHESION	38	0.5298969	18.515.269	0.0	0.0061264574	0.313
CYTOKINE_METABOLIC_PROCESS	42	0.5050512	18.420.597	0.0	0.0065954505	0.336
CELLULAR_HOMEOSTASIS	146	0.4099782	18.424.319	0.0	0.0067053745	0.336
JAK_STAT_CASCADE	30	0.54946035	18.174.378	0.0	0.008762991	0.442
LEUKOCYTE_DIFFERENTIATION	37	0.5198093	18.179.035	0.0032626428	0.008843429	0.439
CYTOKINE_SECRETION	18	0.6214166	18.110.441	0.0050847456	0.009238741	0.465
CELL_MATRIX_ADHESION	37	0.5139995	18.081.076	0.0015923567	0.009315713	0.473
NEGATIVE_REGULATION_OF_MULTICELLULAR_ORGANISMAL_PROCESS	31	0.5361956	1.803.915	0.0016393443	0.009564479	0.488
T_CELL_PROLIFERATION	19	0.6020914	17.927.402	0.0017513135	0.010702862	0.529
REGULATION_OF_DEFENSE_RESPONSE	19	0.6059867	17.878.616	0.007029877	0.010915992	0.545
G_PROTEIN_SIGNALING_COUPLED_TO_CYCLIC_NUCLEOTIDE_SECOND_MESSENGER	100	0.42270386	17.882.652	0.0014388489	0.011034678	0.543
CYTOKINE_AND_CHEMOKINE_MEDIATED_SIGNALING_PATHWAY	22	0.57035005	17.753.948	0.0102739725	0.012302167	0.592
CALCIUM_MEDIATED_SIGNALING	16	0.61996645	17.716.311	0.012658228	0.0127064	0.607
CYCLIC_NUCLEOTIDE_MEDIATED_SIGNALING	102	0.41636667	17.554.556	0.0029282577	0.014808057	0.664
POSITIVE_REGULATION_OF_CELL_PROLIFERATION	149	0.3922275	1.747.775	0.0	0.015952801	0.698
HOMEOSTATIC_PROCESS	206	0.377045	17.458.205	0.0	0.016100932	0.703
CELL_CELL_ADHESION	86	0.4173379	17.370.979	0.0014662757	0.017285122	0.738
REGULATION_OF_SIGNAL_TRANSDUCTION	221	0.36412936	17.140.387	0.0	0.021289956	0.82
INNATE_IMMUNE_RESPONSE	23	0.54799205	16.893.071	0.016694492	0.026632505	0.888
REGULATION_OF_CELL_ADHESION	35	0.4793566	16.861.442	0.0051457975	0.027056757	0.896
SMALL_GTPASE_MEDIATED_SIGNAL_TRANSDUCTION	88	0.40673906	16.780.684	0.0	0.028481038	0.918
LEUKOCYTE_MIGRATION	16	0.5983012	16.789.377	0.017857144	0.028579043	0.915
REGULATION_OF_BIOLOGICAL_QUALITY	416	0.33605337	16.762.364	0.0	0.028669242	0.926
REGULATION_OF_CELL_PROLIFERATION	306	0.34659418	1.670.259	0.0	0.029873751	0.938
CELL_MIGRATION	96	0.3887779	16.564.822	0.0	0.033443835	0.956
NEGATIVE_REGULATION_OF_SIGNAL_TRANSDUCTION	37	0.4727009	16.477.175	0.014469453	0.03556887	0.963
G_PROTEIN_SIGNALING_COUPLED_TO_CAMP_NUCLEOTIDE_SECOND_MESSENGER	64	0.41933173	16.389.685	0.00304414	0.038088784	0.969
REGULATION_OF_CYTOKINE_SECRETION	16	0.58829826	163.405	0.013961606	0.039356817	0.975
G_PROTEIN_SIGNALING_COUPLED_TO_IP3_SECOND_MESSENGERPHOSPHOLIPASE_C_ACTIVATING	45	0.44321364	1.628.245	0.012618297	0.040879335	0.978
NEURON_DIFFERENTIATION	76	0.40028793	16.217.014	0.004524887	0.04294803	0.982
CAMP_MEDIATED_SIGNALING	65	0.41522512	16.193.737	0.001457726	0.043339346	0.983
PROTEIN_SECRETION	32	0.47318235	16.162.884	0.017432647	0.04414901	0.985
POSITIVE_REGULATION_OF_SIGNAL_TRANSDUCTION	125	0.37121367	16.115.342	0.0013568521	0.045548096	0.986
REGULATION_OF_T_CELL_PROLIFERATION	16	0.5608603	16.042.469	0.031088082	0.047812782	0.991
COAGULATION	43	0.4452324	16.002.772	0.013888889	0.04870132	0.992

GO Molecular Function NAME	SIZE	ES	NES	NOM p-val	FDR q-val	FWER p-val
**CYTOKINE_BINDING**	48	0.6331821	22.841.039	0.0	0.0	0.0
**CHEMOKINE_RECEPTOR_BINDING**	43	0.62106246	22.627.044	0.0	0.0	0.0
**INTERLEUKIN_BINDING**	25	0.683845	2.202.532	0.0	0.0	0.0
**CHEMOKINE_ACTIVITY**	42	0.6084665	21.933.975	0.0	0.0	0.0
G_PROTEIN_COUPLED_RECEPTOR_BINDING	54	0.5614492	21.100.106	0.0	6,73E+02	0.005
EXTRACELLULAR_MATRIX_STRUCTURAL_CONSTITUENT	27	0.65362287	2.143.306	0.0	4,65E+03	0.003
HEMATOPOIETIN_INTERFERON_CLASSD200_DOMAIN_CYTOKINE_RECEPTOR_ACTIVITY	33	0.6205426	21.452.909	0.0	5,57E+03	0.003
PHOSPHORIC_DIESTER_HYDROLASE_ACTIVITY	40	0.56964993	20.315.628	0.0	0.0010478327	0.01
INTERLEUKIN_RECEPTOR_ACTIVITY	20	0.67784286	20.464.528	0.0	0.0010673426	0.009
ANTIGEN_BINDING	27	0.61023635	19.850.389	0.0	0.002226636	0.026
TRANSMEMBRANE_RECEPTOR_ACTIVITY	416	0.4012774	19.976.193	0.0	0.0022606754	0.024
RHO_GTPASE_ACTIVATOR_ACTIVITY	19	0.6493542	19.471.574	0.0	0.0040423647	0.048
PHOSPHOLIPASE_C_ACTIVITY	15	0.66761976	18.661.746	0.0	0.010697284	0.137
PROTEIN_TYROSINE_KINASE_ACTIVITY	63	0.47056752	18.475.307	0.0	0.012744042	0.171
PHOSPHOLIPASE_ACTIVITY	42	0.50729364	18.362.352	0.0	0.013561005	0.189
RECEPTOR_SIGNALING_PROTEIN_ACTIVITY	82	0.43047044	17.460.672	0.002994012	0.03433713	0.427
CALMODULIN_BINDING	25	0.541565	17.239.544	0.013266998	0.040058415	0.497
RAS_GTPASE_ACTIVATOR_ACTIVITY	29	0.5266693	17.155.962	0.0034423408	0.040286925	0.516
INTEGRIN_BINDING	30	0.5102038	17.061.732	0.0065252855	0.041996773	0.549
LIPID_BINDING	87	0.4104348	16.802.553	0.0014705883	0.047515728	0.622
CARBOHYDRATE_BINDING	71	0.4218184	16.811.181	0.0015243902	0.04971318	0.621

GO Cellular Component NAME	SIZE	ES	NES	NOM p-val	FDR q-val	FWER p-val
**EXTRACELLULAR_MATRIX**	99	0.60991263	26.049.685	0.0	0.0	0.0
**PROTEINACEOUS_EXTRACELLULAR_MATRIX**	98	0.6120536	25.567.513	0.0	0.0	0.0
**EXTRACELLULAR_MATRIX_PART**	57	0.5674156	2.189.806	0.0	0.0	0.0
**EXTRACELLULAR_REGION_PART**	335	0.44603083	21.701.753	0.0	0.0	0.0
**LIPID_RAFT**	29	0.653096	21.603.768	0.0	0.0	0.0
**COLLAGEN**	23	0.682327	21.477.323	0.0	0.0	0.0
**EXTRACELLULAR_REGION**	442	0.42336974	21.205.728	0.0	0.0	0.0
RECEPTOR_COMPLEX	54	0.5357445	20.056.446	0.0	3,29E+03	0.005
EXTERNAL_SIDE_OF_PLASMA_MEMBRANE	16	0.6711314	19.241.213	0.0	0.0025958682	0.042
CELL_SUBSTRATE_ADHERENS_JUNCTION	16	0.6496239	18.559.018	0.001776199	0.0063270414	0.108
INTEGRIN_COMPLEX	19	0.6167504	18.339.581	0.0036697248	0.00681179	0.137
CELL_SURFACE	78	0.45862836	18.414.276	0.0	0.006845611	0.127
BASEMENT_MEMBRANE	37	0.5143726	18.115.678	0.0015797789	0.008396592	0.18
EXTRACELLULAR_SPACE	242	0.3780886	1.800.035	0.0	0.008646189	0.199
CELL_MATRIX_JUNCTION	18	0.6064776	17.628.424	0.0068728523	0.011516997	0.276
BASAL_LAMINA	21	0.5803754	17.538.316	0.0051107327	0.011721258	0.296
LYSOSOME	61	0.4241982	16.789.434	0.0045045046	0.022236206	0.496
LYTIC_VACUOLE	61	0.4241982	1.646.706	0.009345794	0.028496666	0.602
INSOLUBLE_FRACTION	15	0.5764318	15.954.666	0.036842104	0.04042416	0.76

miRNA NAME	SIZE	ES	NES	NOM p-val	FDR q-val	FWER p-val

0 significat terms

Transcription Factor NAME	SIZE	ES	NES	NOM p-val	FDR q-val	FWER p-val
**V$COREBINDINGFACTOR_Q6**	262	0.48850337	230.569	0.0	0.0	0.0
**V$IRF_Q6**	234	0.4769561	22.442.153	0.0	0.0	0.0
**GGGNNTTTCC_V$NFKB_Q6_01**	132	0.5063444	22.416.205	0.0	0.0	0.0
**V$AML_Q6**	255	0.4606734	22.082.074	0.0	0.0	0.0
**RACCACAR_V$AML_Q6**	252	0.46126232	21.880.171	0.0	0.0	0.0
V$PU1_Q6	223	0.43330595	20.114.594	0.0	2,50E+02	0.004
V$AML1_01	258	0.42228472	20.127.084	0.0	2,67E+02	0.004
V$IRF1_01	241	0.43089455	20.651.941	0.0	2,80E+02	0.002
V$ICSBP_Q6	239	0.3950631	18.552.918	0.0	7,70E+02	0.02
V$IRF7_01	242	0.41226885	19.669.973	0.0	2,10E+03	0.004
V$IRF2_01	121	0.45187533	19.686.435	0.0	2,22E+03	0.004
V$SRF_Q5_01	214	0.42506447	198.127	0.0	2,35E+03	0.004
V$ISRE_01	241	0.43427518	2.054.772	0.0	2,45E+03	0.002
V$STAT5B_01	237	0.40976092	19.420.007	0.0	2,50E+03	0.005
V$IRF1_Q6	244	0.40736574	19.374.042	0.0	2,85E+03	0.006
RGAGGAARY_V$PU1_Q6	485	0.40069374	20.171.177	0.0	2,86E+03	0.004
V$ELF1_Q6	232	0.43160143	20.192.966	0.0	3,08E+03	0.004
STTTCRNTTT_V$IRF_Q6	181	0.46137062	2.105.103	0.0	3,27E+03	0.002
V$PEA3_Q6	250	0.42927888	20.459.585	0.0	3,32E+03	0.003
CCAWWNAAGG_V$SRF_Q4	87	0.48887655	2.026.946	0.0	3,33E+03	0.004
V$AML1_Q6	258	0.42228472	2.031.548	0.0	3,63E+03	0.004
V$ETS2_B	268	0.42377424	20.354.815	0.0	4,00E+03	0.004
V$ETS_Q4	244	0.40257472	18.943.301	0.0	4,08E+03	0.009
V$SRF_Q6	238	0.40048125	18.933.803	0.0	4,33E+03	0.01
V$SRF_C	209	0.40379184	18.773.036	0.0	4,80E+03	0.012
V$RP58_01	198	0.40750626	18.821.038	0.0	5,00E+03	0.012
V$SRF_01	50	0.4943002	18.354.716	0.0016207455	0.0010741383	0.03
V$STAT6_02	250	0.3854962	18.377.388	0.0	0.0010763664	0.029
V$ETS1_B	245	0.38225293	18.271.482	0.0	0.0011417845	0.033
V$NERF_Q2	241	0.3873262	18.244.433	0.0	0.0012382369	0.037
YNTTTNNNANGCARM_UNKNOWN	68	0.4616272	18.125.323	0.0014880953	0.0013616006	0.041
V$STAT4_01	254	0.37773255	17.969.328	0.0	0.0014679665	0.05
V$POU6F1_01	230	0.3812876	18.012.224	0.0	0.0014966326	0.048
V$NFAT_Q6	237	0.3831277	17.970.105	0.0	0.001511142	0.05
V$GATA1_04	237	0.38192433	18.015.342	0.0	0.0015434024	0.048
V$STAT5A_01	241	0.3772046	17.859.259	0.0	0.0017645261	0.061
AAANWWTGC_UNKNOWN	189	0.3815109	17.762.077	0.0	0.0019376534	0.069
V$FOXD3_01	192	0.37677848	17.589.036	0.0	0.002395692	0.087
V$MEF2_02	218	0.37846833	17.572.612	0.0	0.0024365056	0.09
V$NFKB_Q6	252	0.36677605	17.508.775	0.0	0.0025747663	0.097
V$PAX5_02	16	0.60831505	174.533	0.008417509	0.0026107866	0.101
V$OCT1_05	244	0.36836475	17.397.289	0.0	0.002744796	0.109
TTANWNANTGGM_UNKNOWN	58	0.4535573	17.373.544	0.0	0.0027768798	0.112
V$NFKB_Q6_01	228	0.3686697	173.372	0.0	0.0028304947	0.116
V$NFKAPPAB_01	247	0.36057448	17.191.975	0.0	0.0033310675	0.138
V$GATA1_03	239	0.35877156	17.035.255	0.0	0.0036036351	0.165
V$OCT1_04	225	0.36345565	17.006.227	0.0	0.0036224804	0.173
V$PAX4_04	209	0.36475325	17.085.959	0.0	0.003632023	0.153
V$MEF2_03	230	0.36643317	17.058.127	0.0	0.0036353867	0.163
V$E47_02	240	0.3602911	170.161	0.0	0.0036537193	0.171
V$AR_01	148	0.38234442	17.065.939	0.0027322404	0.0036685762	0.161
V$CEBP_01	260	0.35995808	17.066.467	0.0	0.0037257855	0.16
CATTGTYY_V$SOX9_B1	347	0.34580055	16.937.331	0.0	0.0038630913	0.187
V$NKX61_01	227	0.36069572	16.901.692	0.0	0.0038701175	0.195
V$NKX62_Q2	235	0.35737434	16.924.862	0.0	0.003904449	0.193
V$LMO2COM_02	236	0.3542479	1.693.913	0.0	0.0039359797	0.187
V$CDC5_01	239	0.3569457	16.848.186	0.0012804097	0.0039445222	0.21
V$GATA_Q6	191	0.36370614	16.867.437	0.0	0.0039606867	0.207

AAAYWAACM_V$HFH4_01	244	0.35434332	16.877.363	0.0	0.003977453	0.204
V$OCT1_B	256	0.35216755	16.806.433	0.0	0.0040637	0.22
V$PAX_Q6	245	0.35130575	16.730.616	0.0012468828	0.0044777202	0.241
V$TATA_C	276	0.34642512	16.692.663	0.0	0.004512477	0.251
YTAATTAA_V$LHX3_01	180	0.36541253	16.707.512	0.0	0.004519758	0.247
GATAAGR_V$GATA_C	284	0.34525746	1.663.709	0.0	0.004551074	0.264
V$FREAC4_01	145	0.3737893	16.672.404	0.0	0.0045839217	0.257
V$OSF2_Q6	252	0.3517182	16.639.223	0.0	0.004621091	0.264
V$NFKAPPAB65_01	231	0.35412675	16.591.272	0.0012804097	0.0048007173	0.281
V$SRF_Q4	218	0.35117003	16.534.657	0.0012953368	0.0049574	0.302
V$FOXO4_02	248	0.35004145	16.541.332	0.0	0.0049847793	0.299
V$NFAT_Q4_01	259	0.34656936	16.562.954	0.0	0.00499694	0.293
V$GFI1_01	257	0.35029227	16.547.412	0.0	0.0050277645	0.298
WWTAAGGC_UNKNOWN	138	0.3675782	16.411.972	0.0	0.0058420524	0.346
V$HMEF2_Q6	133	0.37044823	16.381.397	0.0	0.0059565944	0.354
V$MAF_Q6	249	0.34600997	16.345.627	0.0	0.006095601	0.363
YKACATTT_UNKNOWN	270	0.33732066	1.626.892	0.0	0.0066354335	0.389
V$MEF2_01	138	0.36592227	16.215.928	0.0013679891	0.007014514	0.411
V$S8_01	240	0.33933654	16.156.965	0.0	0.0075025107	0.435
V$CEBPB_01	251	0.34043127	16.120.723	0.0	0.007746899	0.45
V$STAT5A_03	250	0.34028077	16.109.282	0.0	0.007751229	0.454
V$RSRFC4_01	234	0.3422124	16.083.604	0.0	0.007857302	0.465
YTAAYNGCT_UNKNOWN	146	0.360145	16.054.528	0.0	0.0079464195	0.472
V$IK2_01	264	0.33252898	1.603.715	0.0	0.00803346	0.478
V$CREL_01	251	0.33516204	16.027.013	0.0	0.008081673	0.483
V$CEBP_Q2	222	0.33788043	15.988.058	0.0	0.008306633	0.505
V$NFKB_C	260	0.3366832	1.599.098	0.0	0.008367828	0.502
V$LHX3_01	218	0.33865768	15.998.063	0.0	0.0083711315	0.497
V$TAL1BETAITF2_01	245	0.33755043	15.912.497	0.0025445293	0.0089111915	0.528
V$OCT1_02	206	0.34167835	15.841.428	0.0	0.009438982	0.559
V$HP1SITEFACTOR_Q6	223	0.33425543	1.584.967	0.0	0.009477776	0.557
V$OCT_C	252	0.33330435	15.818.632	0.0	0.009545857	0.564
V$SOX9_B1	227	0.33440655	158.082	0.0013037809	0.009585912	0.572
TGATTTRY_V$GFI1_01	284	0.327154	15.778.502	0.0	0.009737893	0.587
RAAGNYNNCTTY_UNKNOWN	142	0.35579735	15.784.527	0.004316547	0.009777869	0.584
V$TCF11_01	245	0.33508983	15.759.493	0.0	0.009836049	0.597
V$FAC1_01	215	0.33563027	15.726.719	0.0	0.010122795	0.612
V$CIZ_01	229	0.33484593	15.631.437	0.0	0.011014787	0.641
V$GATA6_01	257	0.3276845	15.631.615	0.0	0.011129524	0.641
V$GATA1_05	273	0.3264761	15.584.705	0.0	0.011281988	0.663
V$GATA1_02	235	0.33010048	15.596.566	0.0012484394	0.011304299	0.656
GTGGGTGK_UNKNOWN	279	0.32425913	15.587.524	0.0	0.011374663	0.663
V$PITX2_Q2	240	0.32921234	15.564.591	0.0	0.011499333	0.669
YNGTTNNNATT_UNKNOWN	357	0.31749263	15.526.216	0.0011947431	0.011638857	0.691
V$SRY_02	243	0.32790488	15.530.952	0.0012738854	0.011654389	0.688
V$ELK1_01	261	0.32475325	15.534.827	0.0	0.011668082	0.686
V$EVI1_06	20	0.51822585	15.542.003	0.03208556	0.011674036	0.682
V$NCX_01	161	0.34690702	15.493.746	0.0027472528	0.012044145	0.708
V$PBX1_01	244	0.329364	15.449.549	0.0012738854	0.01230486	0.726
V$FOXJ2_02	227	0.32764244	1.545.634	0.0037783375	0.012354021	0.723
V$LBP1_Q6	210	0.33235344	15.413.846	0.0012987013	0.012712247	0.741
V$OCT1_Q5_01	260	0.3235466	15.401.173	0.001285347	0.012825283	0.745
V$EN1_01	107	0.3578884	15.337.445	0.008695652	0.012960961	0.771
V$MSX1_01	172	0.3361376	15.368.105	0.0013297872	0.013028108	0.757
V$PAX2_02	248	0.32215244	15.338.638	0.002541296	0.013073665	0.771
V$TFIIA_Q6	247	0.3221493	15.320.948	0.0	0.01310244	0.776
V$E47_01	243	0.32676	15.342.371	0.0	0.0131259365	0.769
YATTNATC_UNKNOWN	362	0.31513977	15.369.796	0.0	0.013127625	0.757
TTCYNRGAA_V$STAT5B_01	323	0.31713858	15.350.481	0.0	0.013180877	0.769

V$RSRFC4_Q2	206	0.32833123	15.298.693	0.001333333	0.013189072	0.785
V$MEF2_Q6_01	234	0.32244048	15.307.266	0.002551020	0.013196876	0.782
TGTYNNNNNRGCARM_UNKNOWN	83	0.3765325	15.289.018	0.00729927	0.013264856	0.789
V$CEBP_Q2_01	261	0.31818572	15.266.296	0.001285347	0.013404865	0.797
V$PAX8_01	36	0.43506065	15.252.254	0.019417476	0.013466294	0.805
V$OCT1_Q6	254	0.32098246	152.553	0.001261034	0.013485953	0.802
V$OCT1_03	220	0.3252212	1.526.935	0.001240694	0.013491073	0.796
V$STAT5A_04	204	0.3307845	15.205.677	0.003947368	0.01420596	0.825
V$EVI1_02	127	0.34931022	15.198.393	0.006887052	0.014295264	0.829
V$NKX25_02	256	0.31705487	15.076.215	0.0	0.016280472	0.862
V$OCT_Q6	252	0.31775954	15.052.627	0.003811944	0.016722074	0.87
V$CHX10_01	217	0.3222275	15.043.243	0.001264222	0.016820304	0.874
V$CEBPDELTA_Q6	233	0.31783402	1.502.136	0.001261034	0.016980415	0.875
V$PR_01	145	0.34042045	15.024.625	0.005494505	0.017048607	0.875
V$CRX_Q4	261	0.3109846	14.995.998	0.002421307	0.017211976	0.88
V$GATA3_01	233	0.31785774	15.001.531	0.008053691	0.017279934	0.88
V$HMGIY_Q6	241	0.31466615	14.968.712	0.001288659	0.017516391	0.893
V$POU3F2_02	253	0.3153112	14.972.692	0.0	0.017549725	0.891
CCCNNGGGAR_V$OLF1_01	309	0.3058142	14.888.239	0.0	0.019080954	0.915
V$HAND1E47_01	260	0.31153378	14.788.036	0.001216545	0.021313203	0.936
CTGYNNCTYTAA_UNKNOWN	81	0.36052763	14.776.874	0.018376723	0.021473812	0.939
V$ARP1_01	153	0.32839948	14.743.941	0.005361930	0.021987848	0.945
V$CDX2_Q5	242	0.30948654	14.747.376	0.002541296	0.022058964	0.945
V$GR_01	197	0.31544572	14.714.813	0.001293661	0.022062996	0.947
V$LFA1_Q6	234	0.31180397	1.471.044	0.008985879	0.022065133	0.95
V$HNF3_Q6	184	0.32062897	14.716.935	0.003984064	0.022167949	0.947
V$AREB6_04	243	0.30810732	14.717.249	0.001285347	0.022317111	0.947
V$HOXA4_Q2	258	0.30716243	14.717.886	0.006203474	0.022454122	0.947
V$HFH1_01	235	0.30953655	14.667.747	0.001275510	0.022900606	0.956
V$AR_02	38	0.4146533	14.570.969	0.0521327	0.025599891	0.971
GTTRYCATRR_UNKNOWN	160	0.32129985	14.564.313	0.01076716	0.02568053	0.974
RTTTNNNNYTGGM_UNKNOWN	148	0.32854775	14.557.806	0.006906077	0.02571287	0.974
YATGNWAAT_V$OCT_C	348	0.29602835	14.468.756	0.004761905	0.027442675	0.984
V$MYB_Q6	245	0.30483508	14.470.034	0.005063291	0.027588893	0.984
V$EVI1_05	165	0.32140112	1.447.187	0.009408602	0.027724521	0.984
V$TEF_Q6	245	0.30533335	1.447.447	0.00622665	0.027820831	0.984
V$TAL1BETAE47_01	239	0.3090433	14.475.251	0.003754693	0.027986642	0.984
V$CEBP_Q3	245	0.30443236	14.416.555	0.006265664	0.028918272	0.989
V$AMEF2_Q6	247	0.3030337	14.362.023	0.003797468	0.030524353	0.992
V$AP4_Q6	217	0.30613825	14.365.815	0.00498132	0.030577144	0.992
RGAANNTTC_V$HSF1_01	427	0.28765267	14.349.712	0.002272727	0.030709734	0.992
V$AP1_Q6_01	257	0.29951185	14.332.192	0.002551020	0.031181816	0.993
V$TEL2_Q6	228	0.30429047	14.313.083	0.002560819	0.03175072	0.993
V$FREAC7_01	185	0.3130104	14.295.421	0.010403121	0.03218856	0.993
V$SREBP1_02	86	0.3438137	14.252.584	0.026239067	0.033645112	0.993
V$HNF3ALPHA_Q6	199	0.30345637	14.205.829	0.005154639	0.03525981	0.994
V$CP2_01	254	0.29752567	14.175.178	0.003764115	0.03633073	0.996
V$HNF3B_01	210	0.30072752	14.161.615	0.007915568	0.036696926	0.997
V$FOXO4_01	228	0.29569986	14.111.674	0.009114583	0.03826942	0.998
V$CEBPB_02	246	0.29865044	14.112.448	0.008706467	0.038469244	0.998
V$IK3_01	218	0.30013955	14.092.258	0.009102731	0.038846064	0.999
V$FOX_Q2	206	0.30259466	14.066.373	0.010430247	0.03963326	0.999
CTAWWWATA_V$RSRFC4_Q2	348	0.28586072	14.066.448	0.0	0.039867774	0.999
V$CEBPGAMMA_Q6	245	0.29652533	14.045.807	0.00795756	0.04035338	0.999
V$PTF1BETA_Q6	230	0.2974706	14.031.034	0.005319149	0.040388532	0.999
V$OCT1_07	154	0.31147477	14.034.898	0.013888889	0.04040673	0.999
TGGNNNNNNKCCAR_UNKNOWN	402	0.2837754	14.038.454	0.001183432	0.04050541	0.999
CAGNWMCNNNGAC_UNKNOWN	79	0.34779036	14.004.929	0.034833092	0.04090436	0.999
V$HEN1_02	189	0.30315733	1.400.587	0.017615177	0.041075215	0.999

V$AP4_Q5	258	0.2969809	13.988.718	0.007623888	0.04109306	0.999
V$ALX4_01	16	0.4855166	13.971.324	0.10928962	0.041205708	0.999
V$TAL1ALPHAE47_01	240	0.2955443	13.988.926	0.009043927	0.04131952	0.999
V$GR_Q6	261	0.29160905	13.972.746	0.011392405	0.041361358	0.999
V$SREBP_Q3	249	0.2926546	1.397.339	0.0063211126	0.04157568	0.999
V$SMAD_Q6	241	0.29543325	1.395.749	0.00754717	0.041653752	0.999
V$PAX6_01	96	0.3330057	13.934.876	0.030837005	0.042264625	0.999
V$OCT1_01	254	0.28964105	13.935.357	0.008883249	0.04248452	0.999
V$FOXJ2_01	178	0.30360642	13.913.486	0.0066313	0.043041687	0.999
V$CHOP_01	228	0.2940522	13.886.095	0.0116731515	0.044096623	0.999
RNTCANNRNNYNATTW_UNKNOWN	62	0.35935032	13.879.952	0.05801527	0.04414453	0.999
V$PR_Q2	254	0.29147303	1.380.971	0.0038860103	0.047463857	0.999
V$AP2REP_01	173	0.3027737	1.378.268	0.026595745	0.0484736	0.999
V$SMAD3_Q6	229	0.29173198	13.758.948	0.012903226	0.04950897	1.0

Oncogenic Signature NAME	SIZE	ES	NES	NOM p-val	FDR q-val	FWER p-val
**RPS14_DN.V1_UP**	188	0.6244226	28.577.313	0.0	0.0	0.0
**P53_DN.V1_DN**	189	0.5746401	26.792.438	0.0	0.0	0.0
**CAHOY_ASTROGLIAL**	98	0.5571839	23.306.417	0.0	0.0	0.0
**LEF1_UP.V1_UP**	191	0.49340647	2.276.399	0.0	0.0	0.0
**CSR_LATE_UP.V1_DN**	159	0.50003725	22.705.717	0.0	0.0	0.0
**STK33_SKM_UP**	269	0.46551394	22.278.657	0.0	0.0	0.0
**SNF5_DN.V1_UP**	170	0.47837073	22.088.706	0.0	0.0	0.0
**HOXA9_DN.V1_UP**	189	0.47336954	21.988.037	0.0	0.0	0.0
**VEGF_A_UP.V1_UP**	189	0.4731134	21.808.286	0.0	0.0	0.0
**KRAS.KIDNEY_UP.V1_UP**	142	0.47984126	21.408.238	0.0	0.0	0.0
**PTEN_DN.V1_UP**	182	0.4699401	21.404.803	0.0	0.0	0.0
**STK33_NOMO_UP**	276	0.44791278	21.402.876	0.0	0.0	0.0
**BRCA1_DN.V1_UP**	129	0.4834513	21.200.721	0.0	0.0	0.0
**IL2_UP.V1_DN**	187	0.45594642	21.033.483	0.0	0.0	0.0
**STK33_UP**	275	0.43852395	2.094.354	0.0	0.0	0.0
**PTEN_DN.V2_UP**	136	0.46707594	20.672.445	0.0	0.0	0.0
**JNK_DN.V1_UP**	182	0.4422201	20.214.975	0.0	0.0	0.0
TGFB_UP.V1_UP	182	0.39783335	1.824.934	0.0	5,12E+01	0.015
CYCLIN_D1_KE_.V1_DN	189	0.42602375	19.591.274	0.0	1,78E+02	0.003
KRAS.PROSTATE_UP.V1_UP	132	0.4217848	1.849.612	0.0	3,77E+02	0.01
MEL18_DN.V1_UP	139	0.42237368	18.588.673	0.0	4,03E+02	0.01
BMI1_DN.V1_UP	144	0.4167647	18.475.833	0.0	4,33E+02	0.012
MEL18_DN.V1_DN	144	0.40849307	18.275.557	0.0	5,27E+02	0.015
KRAS.600.LUNG.BREAST_UP.V1_DN	276	0.38043714	18.159.188	0.0	5,64E+02	0.016
BMI1_DN_MEL18_DN.V1_UP	142	0.40743244	17.990.454	0.0	6,36E+02	0.02
IL21_UP.V1_UP	181	0.39309645	18.078.092	0.0	6,40E+02	0.019
EGFR_UP.V1_UP	188	0.38757756	17.893.206	0.0	6,63E+02	0.022
ATF2_UP.V1_DN	182	0.43151346	19.710.097	0.0	1,30E+03	0.002
ATF2_S_UP.V1_DN	179	0.42511615	19.618.685	0.0	1,88E+03	0.003
JNK_DN.V1_DN	180	0.41955614	19.286.509	0.0	1,94E+03	0.004
P53_DN.V2_UP	147	0.4352445	19.350.691	0.0	2,02E+03	0.004
KRAS.LUNG_UP.V1_UP	136	0.43855676	19.375.767	0.0	2,11E+03	0.004
AKT_UP.V1_DN	182	0.42082828	19.380.515	0.0	2,21E+03	0.004
CORDENONSI_YAP_CONSERVED_SIGNATURE	57	0.48766166	18.740.348	0.0	3,60E+03	0.008
KRAS.600.LUNG.BREAST_UP.V1_UP	277	0.3913937	18.770.742	0.0	3,74E+03	0.008
KRAS.600_UP.V1_UP	272	0.38764903	18.623.291	0.0	3,77E+03	0.009
MYC_UP.V1_DN	155	0.4120133	18.500.696	0.0	3,89E+03	0.01
HINATA_NFKB_IMMU_INF	17	0.6503985	18.681.815	0.0	3,91E+03	0.009
E2F1_UP.V1_DN	183	0.39701557	18.036.417	0.0	6,53E+03	0.02
IL15_UP.V1_DN	177	0.3918064	17.953.295	0.0	6,80E+03	0.022
KRAS.300_UP.V1_UP	139	0.39975536	17.670.695	0.0	7,87E+03	0.027
ESC_V6.5_UP_EARLY.V1_DN	170	0.38698152	17.504.812	0.0	0.0010204508	0.036
KRAS.LUNG.BREAST_UP.V1_UP	139	0.39045715	17.413.665	0.0	0.0011520191	0.041
CAMP_UP.V1_DN	194	0.3694115	17.303.969	0.0	0.0012978093	0.05
BMI1_DN_MEL18_DN.V1_DN	141	0.38886586	17.305.303	0.0013661202	0.0013266496	0.05
PTEN_DN.V1_DN	174	0.3788313	1.731.242	0.0	0.0013568007	0.05
WNT_UP.V1_DN	167	0.376704	17.081.398	0.0	0.0014631253	0.057
KRAS.BREAST_UP.V1_UP	136	0.37676817	16.821.352	0.0	0.002139388	0.088
KRAS.KIDNEY_UP.V1_DN	133	0.3832057	16.828.027	0.0	0.0021607776	0.087
RELA_DN.V1_DN	133	0.38481006	16.727.258	0.0014104373	0.002390641	0.101
CTIP_DN.V1_UP	130	0.3830959	16.684.502	0.0	0.0024552722	0.105
KRAS.50_UP.V1_UP	47	0.45453492	16.640.067	0.006451613	0.0025397083	0.11
CAHOY_NEURONAL	96	0.39061064	16.443.694	0.0014409221	0.003599617	0.156
KRAS.BREAST_UP.V1_DN	138	0.371436	16.382.909	0.0	0.003700296	0.161
IL2_UP.V1_UP	182	0.35743392	16.334.194	0.0025806453	0.003921433	0.173
ALK_DN.V1_UP	137	0.36897683	16.278.025	0.0027777778	0.004158387	0.186
PKCA_DN.V1_DN	162	0.35885426	16.231.617	0.0013333333	0.0041930987	0.194
KRAS.600_UP.V1_DN	274	0.33621523	16.232.604	0.0	0.004266662	0.194
RAF_UP.V1_UP	191	0.35265896	16.207.664	0.0013531799	0.004279013	0.2
KRAS.AMP.LUNG_UP.V1_UP	132	0.36812115	1.615.216	0.0	0.004611169	0.219
IL15_UP.V1_UP	181	0.35397673	16.135.879	0.0	0.004611697	0.222
MTOR_UP.N4.V1_DN	174	0.35662848	16.038.215	0.0013315579	0.005169574	0.251

NRL_DN.V1_DN	129	0.3663135	15.970.125	0.001369863	0.005508545	0.267
JAK2_DN.V1_UP	178	0.34450835	15.770.127	0.0027137042	0.007180953	0.331
NOTCH_DN.V1_DN	179	0.34495336	15.719.821	0.0012804097	0.0074416376	0.345
NOTCH_DN.V1_UP	180	0.340849	15.583.539	0.0	0.008769618	0.393
KRAS.PROSTATE_UP.V1_DN	138	0.3553995	15.565.917	0.0013850415	0.008777835	0.398
ATM_DN.V1_DN	146	0.34508628	15.378.934	0.0013927576	0.010851515	0.473
PDGF_UP.V1_DN	127	0.35007742	15.308.726	0.003937008	0.011854242	0.504
RAPA_EARLY_UP.V1_UP	174	0.3337242	15.209.383	0.0040431265	0.012860721	0.54
KRAS.DF.V1_UP	191	0.32879487	1.521.207	0.0026143792	0.012977209	0.537
GCNP_SHH_UP_EARLY.V1_DN	165	0.3275416	15.026.357	0.004037685	0.015825126	0.618
CRX_DN.V1_UP	134	0.336731	14.898.912	0.0014164306	0.01831907	0.676
PRC2_EDD_UP.V1_DN	186	0.32241443	14.781.423	0.0025740026	0.020492993	0.719
KRAS.300_UP.V1_DN	136	0.3327831	146.581	0.01345895	0.022929762	0.763
PRC2_SUZ12_UP.V1_DN	177	0.32145515	14.636.998	0.008253095	0.022957155	0.77
LTE2_UP.V1_DN	192	0.31515417	1.454.994	0.0077419356	0.0251734	0.804
IL21_UP.V1_DN	176	0.31419784	14.442.749	0.013404826	0.028087826	0.852
CSR_EARLY_UP.V1_DN	135	0.32733256	14.395.486	0.023448275	0.029257586	0.867
P53_DN.V2_DN	144	0.31840214	14.275.559	0.02642559	0.03242916	0.903
SNF5_DN.V1_DN	158	0.31684825	14.258.301	0.016574586	0.03247352	0.91
KRAS.AMP.LUNG_UP.V1_DN	137	0.3203887	14.201.272	0.027173912	0.033893026	0.917
ALK_DN.V1_DN	136	0.31542206	14.036.307	0.023188407	0.039127663	0.948
TGFB_UP.V1_DN	186	0.30372775	1.404.578	0.012048192	0.03927089	0.947
PIGF_UP.V1_DN	188	0.30542582	13.979.436	0.015810277	0.040915024	0.955
RELA_DN.V1_UP	147	0.31047836	1.392.786	0.022315202	0.042754423	0.963
MEK_UP.V1_DN	189	0.30033076	13.905.263	0.018087855	0.043225154	0.963
KRAS.LUNG.BREAST_UP.V1_DN	138	0.31364024	13.874.161	0.017639078	0.04356143	0.969
MTOR_UP.V1_DN	181	0.30009377	1.388.298	0.026845638	0.043644115	0.967
AKT_UP_MTOR_DN.V1_DN	178	0.30087206	13.861.936	0.025920874	0.04365036	0.972
ATF2_S_UP.V1_UP	185	0.2976641	13.849.981	0.012295082	0.04368766	0.972
DCA_UP.V1_UP	178	0.30129763	1.372.371	0.017639078	0.048088655	0.983
ATM_DN.V1_UP	144	0.31144783	13.725.749	0.029745042	0.04857589	0.983

Immunological Signature NAME	SIZE	ES	NES	NOM p-val	FDR q-val	FWER p-va
GSE7218_UNSTIM_VS_ANTIGEN_STIM_THROUGH_IGG_BCELL_DN	164	0.7147537	32.440.228	0.0	0.0	0.0
GSE7509_UNSTIM_VS_IFNA_STIM_IMMATURE_DC_DN	168	0.6737467	30.798.836	0.0	0.0	0.0
GSE10325_CD4_TCELL_VS_BCELL_DN	186	0.63913745	29.670.217	0.0	0.0	0.0
GSE11057_PBMC_VS_MEM_CD4_TCELL_UP	192	0.6381964	2.943.033	0.0	0.0	0.0
GSE10325_LUPUS_CD4_TCELL_VS_LUPUS_BCELL_DN	188	0.6408385	29.402.115	0.0	0.0	0.0
GSE19888_ADENOSINE_A3R_INH_PRETREAT_AND_ACT_BY_A3R_VS_TCELL_MEMBRANES_ACT_MAST_CELL_UP	194	0.6310631	2.923.597	0.0	0.0	0.0
GSE19888_ADENOSINE_A3R_ACT_VS_TCELL_MEMBRANES_ACT_IN_MAST_CELL_UP	195	0.62647367	28.901.632	0.0	0.0	0.0
GSE10325_LUPUS_CD4_TCELL_VS_LUPUS_MYELOID_DN	198	0.6216003	2.884.046	0.0	0.0	0.0
GSE1460_INTRATHYMIC_T_PROGENITOR_VS_CD4_THYMOCYTE_DN	192	0.6219865	28.826.554	0.0	0.0	0.0
GSE3039_NKT_CELL_VS_ALPHAALPHA_CD8_TCELL_DN	194	0.62153625	28.603.885	0.0	0.0	0.0
GSE7218_IGM_VS_IGG_SIGNAL_THGOUGH_ANTIGEN_BCELL_DN	167	0.63035506	28.590.515	0.0	0.0	0.0
GSE29618_PDC_VS_MDC_DAY7_FLU_VACCINE_DN	196	0.6116016	2.831.308	0.0	0.0	0.0
GSE10325_LUPUS_CD4_TCELL_VS_LUPUS_BCELL_UP	191	0.6167437	28.278.859	0.0	0.0	0.0
GSE21670_UNTREATED_VS_IL6_TREATED_STAT3_KO_CD4_TCELL_UP	195	0.60750586	28.253.653	0.0	0.0	0.0
GSE24634_IL4_VS_CTRL_TREATED_NAIVE_CD4_TCELL_DAY3_DN	195	0.6044646	28.137.617	0.0	0.0	0.0
GSE40685_TREG_VS_FOXP3_KO_TREG_PRECURSOR_DN	192	0.6054523	28.068.964	0.0	0.0	0.0
GSE29618_PDC_VS_MDC_DN	197	0.60266966	28.048.773	0.0	0.0	0.0
GSE24634_TREG_VS_TCONV_POST_DAY10_IL4_CONVERSION_DN	198	0.6030286	2.794.308	0.0	0.0	0.0
GSE10325_CD4_TCELL_VS_BCELL_UP	192	0.60253614	27.873.075	0.0	0.0	0.0
GSE43863_DAY6_EFF_VS_DAY150_MEM_LY6C_INT_CXCR5POS_CD4_TCELL_DN	192	0.60267437	27.776.916	0.0	0.0	0.0
GSE37533_PPARG1_FOXP3_VS_PPARG2_FOXP3_TRANSDUCED_CD4_TCELL_PIOGLITAZONE_TREATED_DN	194	0.6030138	27.767.358	0.0	0.0	0.0
GSE10325_CD4_TCELL_VS_MYELOID_DN	192	0.59901124	27.722.561	0.0	0.0	0.0
GSE24634_TREG_VS_TCONV_POST_DAY5_IL4_CONVERSION_DN	196	0.5982204	27.631.793	0.0	0.0	0.0
GSE37533_PPARG1_FOXP3_VS_FOXP3_TRANSDUCED_CD4_TCELL_DN	196	0.5913073	27.539.651	0.0	0.0	0.0
GSE22886_NAIVE_CD8_TCELL_VS_MONOCYTE_DN	197	0.58943295	27.530.124	0.0	0.0	0.0
GSE24634_TEFF_VS_TCONV_DAY3_IN_CULTURE_DN	189	0.6000759	27.524.643	0.0	0.0	0.0
GSE24634_TREG_VS_TCONV_POST_DAY7_IL4_CONVERSION_DN	193	0.5949426	27.504.947	0.0	0.0	0.0
GSE7509_UNSTIM_VS_FCGRIIB_STIM_DC_DN	169	0.6110045	27.476.795	0.0	0.0	0.0
GSE29618_MONOCYTE_VS_PDC_UP	192	0.5880238	27.237.036	0.0	0.0	0.0
GSE42021_CD24HI_VS_CD24INT_TREG_THYMUS_DN	194	0.5873849	2.712.839	0.0	0.0	0.0
GSE21670_STAT3_KO_VS_WT_CD4_TCELL_TGFB_IL6_TREATED_DN	194	0.577195	26.852.486	0.0	0.0	0.0
GSE13485_CTRL_VS_DAY7_YF17D_VACCINE_PBMC_DN	193	0.57443804	2.683.801	0.0	0.0	0.0
GSE6259_33D1_POS_DC_VS_CD4_TCELL_DN	171	0.58910346	26.680.512	0.0	0.0	0.0
GSE39556_UNTREATED_VS_3H_POLYIC_INJ_MOUSE_CD8A_DC_UP	198	0.57354414	2.655.033	0.0	0.0	0.0
GSE37533_PPARG2_FOXP3_VS_FOXP3_TRANSDUCED_CD4_TCELL_DN	192	0.57450736	26.519.425	0.0	0.0	0.0
GSE42021_CD24HI_VS_CD24LOW_TCONV_THYMUS_DN	192	0.5721439	2.648.619	0.0	0.0	0.0
GSE22886_NAIVE_TCELL_VS_MONOCYTE_DN	197	0.57176703	26.421.485	0.0	0.0	0.0
GSE22886_NAIVE_CD4_TCELL_VS_MONOCYTE_DN	198	0.57293284	26.408.794	0.0	0.0	0.0
GSE30083_SP2_VS_SP4_THYMOCYTE_DN	194	0.5741297	26.396.964	0.0	0.0	0.0
GSE24634_TREG_VS_TCONV_POST_DAY3_IL4_CONVERSION_DN	194	0.5686355	26.377.287	0.0	0.0	0.0
GSE22886_NAIVE_CD8_TCELL_VS_MONOCYTE_UP	192	0.5667605	2.632.428	0.0	0.0	0.0
GSE1432_CTRL_VS_IFNG_24H_MICROGLIA_DN	194	0.5679589	26.298.933	0.0	0.0	0.0
GSE42021_TREG_PLN_VS_CD24INT_TREG_THYMUS_DN	197	0.5656652	26.287.413	0.0	0.0	0.0
GSE24634_TEFF_VS_TCONV_DAY7_IN_CULTURE_DN	193	0.57303476	2.624.501	0.0	0.0	0.0
GSE29618_BCELL_VS_MDC_DAY7_FLU_VACCINE_UP	186	0.57004595	26.189.754	0.0	0.0	0.0
GSE22140_GERMFREE_VS_SPF_MOUSE_CD4_TCELL_UP	197	0.5665168	26.178.432	0.0	0.0	0.0
GSE18791_CTRL_VS_NEWCASTLE_VIRUS_DC_8H_DN	186	0.5657469	26.055.024	0.0	0.0	0.0
GSE26495_NAIVE_VS_PD1LOW_CD8_TCELL_DN	195	0.5713049	25.996.506	0.0	0.0	0.0
GSE42021_TREG_VS_TCONV_PLN_UP	193	0.56326336	25.965.986	0.0	0.0	0.0
GSE29618_BCELL_VS_PDC_UP	185	0.56003845	25.955.148	0.0	0.0	0.0
GSE10325_BCELL_VS_MYELOID_DN	193	0.5612032	25.943.964	0.0	0.0	0.0
GSE19888_ADENOSINE_A3R_INH_VS_ACT_WITH_INHIBITOR_PRETREATMENT_IN_MAST_CELL_UP	189	0.563452	2.593.636	0.0	0.0	0.0
GSE2405_0H_VS_12H_A_PHAGOCYTOPHILUM_STIM_NEUTROPHIL_UP	196	0.55660796	25.821.662	0.0	0.0	0.0
GSE29618_MONOCYTE_VS_MDC_DAY7_FLU_VACCINE_UP	196	0.5591251	25.813.847	0.0	0.0	0.0
GSE10325_CD4_TCELL_VS_MYELOID_UP	190	0.55938494	2.580.364	0.0	0.0	0.0
GSE26495_NAIVE_VS_PD1HIGH_CD8_TCELL_DN	189	0.56226796	25.675.066	0.0	0.0	0.0
GSE2935_UV_INACTIVATED_VS_LIVE_SENDAI_VIRUS_INF_MACROPHAGE_DN	170	0.55978376	2.566.302	0.0	0.0	0.0
GSE19888_CTRL_VS_TCELL_MEMBRANES_ACT_MAST_CELL_PRETREAT_A3R_INH_DN	196	0.5557534	25.637.372	0.0	0.0	0.0
GSE40685_TREG_VS_FOXP3_KO_TREG_PRECURSOR_UP	189	0.5538603	256.216	0.0	0.0	0.0
GSE11057_CD4_EFF_MEM_VS_PBMC_DN	190	0.5568378	25.557.497	0.0	0.0	0.0
GSE18791_CTRL_VS_NEWCASTLE_VIRUS_DC_10H_DN	190	0.5528017	25.461.206	0.0	0.0	0.0
GSE29618_MONOCYTE_VS_PDC_DAY7_FLU_VACCINE_UP	198	0.5497834	25.460.868	0.0	0.0	0.0
GSE40274_CTRL_VS_XBP1_TRANSDUCED_ACTIVATED_CD4_TCELL_UP	164	0.5668613	2.545.888	0.0	0.0	0.0
GSE17974_IL4_AND_ANTI_IL12_VS_UNTREATED_72H_ACT_CD4_TCELL_DN	184	0.5495284	25.457.964	0.0	0.0	0.0
GSE16450_IMMATURE_VS_MATURE_NEURON_CELL_LINE_UP	173	0.5552355	25.450.575	0.0	0.0	0.0
GSE29618_BCELL_VS_MDC_UP	183	0.55712897	2.544.604	0.0	0.0	0.0
GSE22886_DAY0_VS_DAY1_MONOCYTE_IN_CULTURE_UP	198	0.5430751	25.414.982	0.0	0.0	0.0
GSE22935_WT_VS_MYD88_KO_MACROPHAGE_UP	196	0.5507969	25.410.016	0.0	0.0	0.0
GSE3039_ALPHAALPHA_VS_ALPHABETA_CD8_TCELL_DN	193	0.54679906	2.540.947	0.0	0.0	0.0
GSE29618_MONOCYTE_VS_MDC_UP	197	0.54700696	25.372.934	0.0	0.0	0.0
GSE22886_DAY0_VS_DAY7_MONOCYTE_IN_CULTURE_UP	192	0.54683983	25.268.176	0.0	0.0	0.0
GSE41978_ID2_KO_VS_BIM_KO_KLRG1_LOW_EFFECTOR_CD8_TCELL_UP	195	0.5444491	25.228.276	0.0	0.0	0.0
GSE10325_LUPUS_BCELL_VS_LUPUS_MYELOID_DN	198	0.54140544	25.204.027	0.0	0.0	0.0
GSE43863_NAIVE_VS_MEMORY_TH1_CD4_TCELL_D150_LCMV_UP	165	0.5574333	25.197.978	0.0	0.0	0.0
GSE22886_CTRL_VS_LPS_24H_DC_DN	197	0.54140556	25.057.335	0.0	0.0	0.0
GSE5589_LPS_AND_IL10_VS_LPS_AND_IL6_STIM_IL6_KO_MACROPHAGE_45MIN_UP	195	0.5371887	25.056.481	0.0	0.0	0.0
GSE13484_UNSTIM_VS_YF17D_VACCINE_STIM_PBMC_DN	193	0.5420988	25.042.574	0.0	0.0	0.0
GSE18791_UNSTIM_VS_NEWCATSLE_VIRUS_DC_6H_DN	182	0.5470558	25.041.692	0.0	0.0	0.0
GSE42021_TREG_PLN_VS_TREG_PRECURSORS_THYMUS_DN	195	0.535888	2.499.702	0.0	0.0	0.0
GSE15767_MED_VS_SCS_MAC_LN_UP	196	0.5420458	24.986.916	0.0	0.0	0.0
GSE34156_UNTREATED_VS_6H_TLR1_TLR2_LIGAND_TREATED_MONOCYTE_UP	191	0.5456084	24.968.505	0.0	0.0	0.0
GSE22886_NAIVE_CD4_TCELL_VS_MONOCYTE_UP	190	0.5333611	24.962.695	0.0	0.0	0.0
GSE13485_CTRL_VS_DAY3_YF17D_VACCINE_PBMC_DN	187	0.5401889	24.924.695	0.0	0.0	0.0
GSE34156_TLR1_TLR2_LIGAND_VS_NOD2_AND_TLR1_TLR2_LIGAND_24H_TREATED_MONOCYTE_UP	192	0.53579164	24.892.778	0.0	0.0	0.0
GSE34156_NOD2_LIGAND_VS_TLR1_TLR2_LIGAND_6H_TREATED_MONOCYTE_DN	190	0.5413439	2.489.227	0.0	0.0	0.0
GSE18791_UNSTIM_VS_NEWCATSLE_VIRUS_DC_10H_DN	183	0.5426382	2.484.994	0.0	0.0	0.0
GSE3565_CTRL_VS_LPS_INJECTED_SPLENOCYTES_UP	168	0.5446187	24.846.754	0.0	0.0	0.0
GSE3565_CTRL_VS_LPS_INJECTED_DUSP1_KO_SPLENOCYTES_UP	173	0.54407334	2.481.495	0.0	0.0	0.0

GSE41867_MEMORY_VS_EXHAUSTED_CD8_TCELL_DAY30_LCMV_UP	191	0.5449262	24.805.136	0.0	0.0	0.0
GSE29618_BCELL_VS_MONOCYTE_DN	197	0.52908695	24.700.904	0.0	0.0	0.0
GSE3039_CD4_TCELL_VS_ALPHABETA_CD8_TCELL_UP	192	0.53557295	24.690.037	0.0	0.0	0.0
GSE1460_DP_VS_CD4_THYMOCYTE_DN	194	0.5346789	2.468.467	0.0	0.0	0.0
GSE3565_DUSP1_VS_WT_SPLENOCYTES_UP	160	0.5429443	246.577	0.0	0.0	0.0
GSE20715_0H_VS_24H_OZONE_LUNG_UP	193	0.5318066	2.463.912	0.0	0.0	0.0
GSE3982_MEMORY_CD4_TCELL_VS_BCELL_UP	188	0.5366534	24.626.667	0.0	0.0	0.0
GSE16450_CTRL_VS_IFNA_6H_STIM_IMMATURE_NEURON_CELL_LINE_DN	181	0.53417367	24.610.457	0.0	0.0	0.0
GSE21360_PRIMARY_VS_QUATERNARY_MEMORY_CD8_TCELL_UP	172	0.5408381	24.529.521	0.0	0.0	0.0
GSE3982_MEMORY_CD4_TCELL_VS_BCELL_DN	189	0.52734876	2.452.329	0.0	0.0	0.0
GSE29618_BCELL_VS_PDC_DAY7_FLU_VACCINE_UP	184	0.5305333	24.500.794	0.0	0.0	0.0
GSE30083_SP3_VS_SP4_THYMOCYTE_DN	194	0.52880925	2.449.545	0.0	0.0	0.0
GSE29618_BCELL_VS_MONOCYTE_UP	184	0.5348974	24.477.165	0.0	0.0	0.0
GSE21546_WT_VS_SAP1A_KO_DP_THYMOCYTES_UP	187	0.5358729	24.476.926	0.0	0.0	0.0
GSE14000_UNSTIM_VS_4H_LPS_DC_DN	190	0.53012717	2.441.794	0.0	0.0	0.0
GSE22886_CD4_TCELL_VS_BCELL_NAIVE_UP	195	0.52538353	24.391.832	0.0	0.0	0.0
GSE17301_ACD3_ACD28_VS_ACD3_ACD28_AND_IFNA5_STIM_CD8_TCELL_DN	196	0.52574366	243.715	0.0	0.0	0.0
GSE17974_0H_VS_24H_IN_VITRO_ACT_CD4_TCELL_UP	172	0.5362164	2.435.692	0.0	0.0	0.0
GSE8835_CD4_VS_CD8_TCELL_CLL_PATIENT_UP	192	0.5266086	24.296.594	0.0	0.0	0.0
GSE45739_UNSTIM_VS_ACD3_ACD28_STIM_NRAS_KO_CD4_TCELL_DN	185	0.5251911	2.422.996	0.0	0.0	0.0
GSE29618_BCELL_VS_MDC_DAY7_FLU_VACCINE_DN	192	0.52276295	24.212.935	0.0	0.0	0.0
GSE22196_HEALTHY_VS_OBESE_MOUSE_SKIN_GAMMADELTA_TCELL_UP	193	0.52068007	24.209.986	0.0	0.0	0.0
GSE11057_CD4_CENT_MEM_VS_PBMC_DN	188	0.52793384	242.026	0.0	0.0	0.0
GSE18791_CTRL_VS_NEWCASTLE_VIRUS_DC_16H_DN	180	0.5307334	2.417.805	0.0	0.0	0.0
GSE22886_NAIVE_BCELL_VS_BLOOD_PLASMA_CELL_UP	197	0.5195552	24.138.713	0.0	0.0	0.0
GSE36826_WT_VS_IL1R_KO_SKIN_STAPH_AUREUS_INF_DN	193	0.5158629	2.413.138	0.0	0.0	0.0
GSE20715_0H_VS_48H_OZONE_TLR4_KO_LUNG_UP	197	0.5192772	24.127.083	0.0	0.0	0.0
GSE23568_ID3_KO_VS_WT_CD8_TCELL_DN	196	0.5208354	24.120.326	0.0	0.0	0.0
GSE26488_CTRL_VS_PEPTIDE_INJECTION_OT2_THYMOCYTE_DN	178	0.5243641	24.103.425	0.0	0.0	0.0
GSE11864_CSF1_PAM3CYS_VS_CSF1_IFNG_PAM3CYS_IN_MAC_DN	181	0.5268524	24.102.478	0.0	0.0	0.0
GSE10325_LUPUS_CD4_TCELL_VS_LUPUS_MYELOID_UP	192	0.5217242	2.409.914	0.0	0.0	0.0
GSE9988_ANTI_TREM1_VS_VEHICLE_TREATED_MONOCYTES_DN	193	0.52187294	24.082.897	0.0	0.0	0.0
GSE11057_NAIVE_VS_EFF_MEMORY_CD4_TCELL_DN	188	0.51780784	2.407.181	0.0	0.0	0.0
GSE22140_GERMFREE_VS_SPF_ARTHRITIC_MOUSE_CD4_TCELL_DN	196	0.51549435	24.058.292	0.0	0.0	0.0
KAECH_NAIVE_VS_DAY15_EFF_CD8_TCELL_DN	194	0.5150343	24.044.988	0.0	0.0	0.0
GSE6259_CD4_TCELL_VS_CD8_TCELL_DN	166	0.530565	24.025.576	0.0	0.0	0.0
GSE36078_WT_VS_IL1R_KO_LUNG_DC_AFTER_AD5_INF_UP	196	0.52202934	24.020.965	0.0	0.0	0.0
GSE21774_CD62L_POS_CD56_BRIGHT_VS_CD62L_NEG_CD56_DIM_NK_CELL_UP	195	0.51890755	23.972.538	0.0	0.0	0.0
GSE21360_PRIMARY_VS_TERTIARY_MEMORY_CD8_TCELL_DN	198	0.51821107	23.962.333	0.0	0.0	0.0
GSE21546_UNSTIM_VS_ANTI_CD3_STIM_ELK1_KO_DP_THYMOCYTES_UP	169	0.5210479	2.394.978	0.0	0.0	0.0
GSE42724_NAIVE_BCELL_VS_PLASMABLAST_UP	186	0.52282333	23.942.482	0.0	0.0	0.0
GSE18791_CTRL_VS_NEWCASTLE_VIRUS_DC_6H_DN	192	0.5185075	23.931.754	0.0	0.0	0.0
GSE5679_RARA_AGONIST_AM580_VS_AM580_AND_ROSIGLITAZONE_TREATED_DC_DN	196	0.51615185	23.921.146	0.0	0.0	0.0
GSE22886_DC_VS_MONOCYTE_DN	194	0.5175825	2.387.968	0.0	0.0	0.0
GSE39110_UNTREATED_VS_IL2_TREATED_CD8_TCELL_DAY6_POST_IMMUNIZATION_UP	196	0.5176619	23.797.839	0.0	0.0	0.0
GSE23505_UNTREATED_VS_4DAY_IL6_IL1_IL23_TREATED_CD4_TCELL_DN	197	0.51124257	23.744.228	0.0	0.0	0.0
GSE1432_CTRL_VS_IFNG_6H_MICROGLIA_DN	196	0.50639194	23.721.046	0.0	0.0	0.0
GSE29618_BCELL_VS_MONOCYTE_DAY7_FLU_VACCINE_UP	186	0.51709074	23.720.584	0.0	0.0	0.0
GSE22886_CD8_TCELL_VS_BCELL_NAIVE_DN	188	0.51162404	23.719.964	0.0	0.0	0.0
GSE24634_IL4_VS_CTRL_TREATED_NAIVE_CD4_TCELL_DAY5_UP	197	0.51192063	23.718.724	0.0	0.0	0.0
GSE21380_NON_TFH_VS_TFH_CD4_TCELL_UP	193	0.5130693	23.695.934	0.0	0.0	0.0
GSE12366_GC_VS_NAIVE_BCELL_DN	186	0.5128391	23.689.303	0.0	0.0	0.0
GSE7460_CD8_TCELL_VS_CD4_TCELL_ACT_UP	192	0.51255274	23.635.705	0.0	0.0	0.0
GSE360_HIGH_DOSE_B_MALAYI_VS_M_TUBERCULOSIS_DC_DN	196	0.50796264	23.629.944	0.0	0.0	0.0
GSE39556_CD8A_DC_VS_NK_CELL_DN	194	0.5072532	23.593.526	0.0	0.0	0.0
GSE10325_BCELL_VS_MYELOID_UP	186	0.51631826	23.576.076	0.0	0.0	0.0
GSE40274_FOXP3_VS_FOXP3_AND_PBX1_TRANSDUCED_ACTIVATED_CD4_TCELL_UP	191	0.5129057	23.566.327	0.0	0.0	0.0
GSE41087_WT_VS_FOXP3_MUT_ANTI_CD3_CD28_STIM_CD4_TCELL_UP	193	0.5051582	23.553.324	0.0	0.0	0.0
GSE32533_MIR17_KO_VS_MIR17_OVEREXPRESS_ACT_CD4_TCELL_UP	191	0.5106107	2.347.584	0.0	0.0	0.0
GSE43863_NAIVE_VS_MEMORY_TFH_CD4_TCELL_D150_LCMV_UP	187	0.5088649	23.453.214	0.0	0.0	0.0
GSE22601_IMMATURE_CD4_SINGLE_POSITIVE_VS_CD8_SINGLE_POSITIVE_THYMOCYTE_UP	195	0.50630224	23.434.055	0.0	0.0	0.0
GSE23568_CTRL_TRANSDUCED_VS_WT_CD8_TCELL_DN	195	0.50512874	2.337.306	0.0	0.0	0.0
GSE21379_WT_VS_SAP_KO_TFH_CD4_TCELL_DN	192	0.5005246	23.372.476	0.0	0.0	0.0
KAECH_NAIVE_VS_MEMORY_CD8_TCELL_DN	197	0.50326645	23.360.474	0.0	0.0	0.0
GSE13485_DAY1_VS_DAY7_YF17D_VACCINE_PBMC_DN	189	0.50815964	23.351.016	0.0	0.0	0.0
GSE34156_UNTREATED_VS_24H_NOD2_LIGAND_TREATED_MONOCYTE_DN	194	0.50553775	23.348.298	0.0	0.0	0.0
GSE16266_LPS_VS_HEATSHOCK_AND_LPS_STIM_MEF_UP	196	0.5081389	23.342.562	0.0	0.0	0.0
GSE8921_UNSTIM_VS_TLR1_2_STIM_MONOCYTE_24H_UP	194	0.50722796	23.331.776	0.0	0.0	0.0
GSE13485_PRE_VS_POST_YF17D_VACCINATION_PBMC_DN	189	0.50918525	23.325.198	0.0	0.0	0.0
GSE21033_CTRL_VS_POLYIC_STIM_DC_12H_UP	152	0.5207305	23.317.938	0.0	0.0	0.0
GSE24634_IL4_VS_CTRL_TREATED_NAIVE_CD4_TCELL_DAY5_DN	197	0.50606006	2.330.614	0.0	0.0	0.0
GSE40274_CTRL_VS_FOXP3_AND_LEF1_TRANSDUCED_ACTIVATED_CD4_TCELL_UP	163	0.518905	23.296.413	0.0	0.0	0.0
GSE32986_UNSTIM_VS_CURDLAN_HIGHDOSE_STIM_DC_DN	184	0.50469214	23.286.362	0.0	0.0	0.0
GSE15330_MEGAKARYOCYTE_ERYTHROID_VS_GRANULOCYTE_MONOCYTE_PROGENITOR_UP	173	0.51002616	2.328.037	0.0	0.0	0.0
GSE29618_BCELL_VS_MONOCYTE_DAY7_FLU_VACCINE_DN	196	0.5052305	23.265.924	0.0	0.0	0.0
GSE17974_IL4_AND_ANTI_IL12_VS_UNTREATED_48H_ACT_CD4_TCELL_DN	180	0.5102278	23.253.555	0.0	0.0	0.0
GSE24142_EARLY_THYMIC_PROGENITOR_VS_DN2_THYMOCYTE_UP	196	0.5040198	23.251.872	0.0	0.0	0.0
GSE360_CTRL_VS_M_TUBERCULOSIS_DC_DN	198	0.49923992	23.217.635	0.0	0.0	0.0
GSE19888_ADENOSINE_A3R_INH_VS_TCELL_MEMBRANES_ACT_MAST_CELL_UP	191	0.50392425	23.203.065	0.0	0.0	0.0
GSE29618_BCELL_VS_MDC_DN	197	0.50398034	23.202.865	0.0	0.0	0.0
GSE21360_NAIVE_VS_PRIMARY_MEMORY_CD8_TCELL_DN	177	0.5059375	23.200.145	0.0	0.0	0.0
GSE3982_DC_VS_TH1_UP	191	0.49904224	23.192.697	0.0	0.0	0.0
GSE16450_IMMATURE_VS_MATURE_NEURON_CELL_LINE_12H_IFNA_STIM_UP	195	0.5025294	23.180.916	0.0	0.0	0.0
GSE17974_CTRL_VS_ACT_IL4_AND_ANTI_IL12_24H_CD4_TCELL_UP	177	0.5082677	23.173.316	0.0	0.0	0.0
GSE25677_MPL_VS_R848_STIM_BCELL_DN	175	0.5105737	23.170.314	0.0	0.0	0.0
GSE3920_IFNA_VS_IFNB_TREATED_ENDOTHELIAL_CELL_DN	161	0.51750386	2.311.507	0.0	0.0	0.0
GSE14000_UNSTIM_VS_4H_LPS_DC_TRANSLATED_RNA_DN	186	0.5040545	23.104.236	0.0	0.0	0.0
GSE2706_UNSTIM_VS_2H_LPS_AND_R848_DC_DN	176	0.5130941	23.095.117	0.0	0.0	0.0
GSE9650_NAIVE_VS_MEMORY_CD8_TCELL_DN	197	0.49974573	23.072.371	0.0	0.0	0.0

GSE2405_S_AUREUS_VS_A_PHAGOCYTOPHILUM_NEUTROPHIL_UP	196	0.49588305	2.303.453	0.0	0.0	0.0
GSE30971_CTRL_VS_LPS_STIM_MACROPHAGE_WBP7_HET_2H_DN	185	0.49913993	23.024.397	0.0	0.0	0.0
GSE40666_UNTREATED_VS_IFNA_STIM_STAT1_KO_CD8_TCELL_90MIN_UP	167	0.50680345	23.017.728	0.0	0.0	0.0
GSE7548_NAIVE_VS_DAY7_PCC_IMMUNIZATION_CD4_TCELL_DN	197	0.49846464	23.016.777	0.0	0.0	0.0
GSE43863_TH1_VS_TFH_MEMORY_CD4_TCELL_DN	191	0.49902505	22.996.216	0.0	0.0	0.0
GSE6259_FLT3L_INDUCED_33D1_POS_DC_VS_CD4_TCELL_UP	170	0.5076762	2.299.235	0.0	0.0	0.0
GSE18791_CTRL_VS_NEWCASTLE_VIRUS_DC_18H_DN	175	0.50668174	2.298.723	0.0	0.0	0.0
GSE32986_CURDLAN_HIGHDOSE_VS_GMCSF_AND_CURDLAN_HIGHDOSE_STIM_DC_DN	197	0.49796677	22.980.063	0.0	0.0	0.0
GSE2770_TGFB_AND_IL4_VS_IL12_TREATED_ACT_CD4_TCELL_6H_UP	195	0.4945217	22.964.227	0.0	0.0	0.0
GSE5542_UNTREATED_VS_IFNG_TREATED_EPITHELIAL_CELLS_6H_DN	192	0.49432597	2.296.208	0.0	0.0	0.0
GSE15330_LYMPHOID_MULTIPOTENT_VS_MEGAKARYOCYTE_ERYTHROID_PROGENITOR_IKAROS_KO_DN	175	0.50064105	22.945.921	0.0	0.0	0.0
GSE25123_WT_VS_PPARG_KO_MACROPHAGE_UP	181	0.4981471	2.294.177	0.0	0.0	0.0
GSE3982_CTRL_VS_LPS_4H_MAC_DN	198	0.49659196	22.938.902	0.0	0.0	0.0
GSE6269_E_COLI_VS_STAPH_AUREUS_INF_PBMC_UP	192	0.4930095	22.936.387	0.0	0.0	0.0
GSE12845_PRE_GC_VS_DARKZONE_GC_TONSIL_BCELL_UP	191	0.49965116	22.915.442	0.0	0.0	0.0
GSE19198_1H_VS_24H_IL21_TREATED_TCELL_DN	188	0.49688432	22.913.964	0.0	0.0	0.0
GSE21033_CTRL_VS_POLYIC_STIM_DC_24H_UP	147	0.51478773	22.902.446	0.0	0.0	0.0
GSE30971_CTRL_VS_LPS_STIM_MACROPHAGE_WBP7_KO_4H_DN	179	0.5046524	22.877.643	0.0	0.0	0.0
GSE24142_EARLY_THYMIC_PROGENITOR_VS_DN2_THYMOCYTE_ADULT_UP	196	0.4941866	2.287.539	0.0	0.0	0.0
GSE41867_NAIVE_VS_DAY30_LCMV_CLONE13_EXHAUSTED_CD8_TCELL_UP	185	0.50172466	2.285.844	0.0	0.0	0.0
GSE18791_UNSTIM_VS_NEWCATSLE_VIRUS_DC_18H_DN	171	0.50105995	2.282.919	0.0	0.0	0.0
GOLDRATH_NAIVE_VS_MEMORY_CD8_TCELL_DN	197	0.49288306	22.809.432	0.0	0.0	0.0
GSE24634_TEFF_VS_TCONV_DAY10_IN_CULTURE_DN	198	0.4901694	2.280.326	0.0	0.0	0.0
GSE1432_1H_VS_24H_IFNG_MICROGLIA_DN	191	0.49203774	22.758.455	0.0	0.0	0.0
GSE16450_CTRL_VS_IFNA_6H_STIM_MATURE_NEURON_CELL_LINE_UP	191	0.48879382	2.272.272	0.0	0.0	0.0
GSE36826_WT_VS_IL1R_KO_SKIN_UP	196	0.494177	227.223	0.0	0.0	0.0
GSE5589_LPS_VS_LPS_AND_IL10_STIM_IL10_KO_MACROPHAGE_45MIN_DN	191	0.4939868	22.701.335	0.0	0.0	0.0
GSE41176_UNSTIM_VS_ANTI_IGM_STIM_BCELL_1H_DN	193	0.49421197	2.267.807	0.0	0.0	0.0
GSE14415_INDUCED_TREG_VS_FOXP3_KO_INDUCED_TREG_IL2_CULTURE_UP	166	0.50373995	22.653.673	0.0	0.0	0.0
GSE39556_UNTREATED_VS_3H_POLYIC_INJ_MOUSE_CD8A_DC_DN	196	0.4923873	22.648.652	0.0	0.0	0.0
GSE5542_UNTREATED_VS_IFNG_TREATED_EPITHELIAL_CELLS_24H_UP	192	0.4868411	2.262.151	0.0	0.0	0.0
GSE40274_CTRL_VS_FOXP3_TRANSDUCED_ACTIVATED_CD4_TCELL_DN	194	0.49133062	22.583.377	0.0	0.0	0.0
GSE22886_NAIVE_TCELL_VS_DC_UP	188	0.48349944	22.555.869	0.0	0.0	0.0
GSE19941_UNSTIM_VS_LPS_AND_IL10_STIM_IL10_KO_MACROPHAGE_UP	192	0.48676735	22.501.357	0.0	0.0	0.0
GSE36888_STAT5_AB_KNOCKIN_VS_WT_TCELL_IL2_TREATED_2H_UP	192	0.48418158	2.244.524	0.0	0.0	0.0
GSE21063_WT_VS_NFATC1_KO_3H_ANTI_IGM_STIM_BCELL_DN	192	0.48412016	2.241.562	0.0	0.0	0.0
GSE13411_PLASMA_CELL_VS_MEMORY_BCELL_DN	189	0.4863343	22.412.586	0.0	0.0	0.0
GSE22886_NAIVE_TCELL_VS_MONOCYTE_UP	189	0.48731634	22.388.933	0.0	0.0	0.0
GSE18791_CTRL_VS_NEWCASTLE_VIRUS_DC_12H_DN	186	0.48492286	22.370.012	0.0	0.0	0.0
GSE22886_TCELL_VS_BCELL_NAIVE_DN	189	0.48569924	2.236.702	0.0	0.0	0.0
GSE45382_UNTREATED_VS_TGFB_TREATED_MACROPHAGES_UP	194	0.48240432	22.353.106	0.0	0.0	0.0
GSE32986_UNSTIM_VS_GMCSF_AND_CURDLAN_LOWDOSE_STIM_DC_DN	184	0.490677	22.341.955	0.0	0.0	0.0
GSE9988_ANTI_TREM1_VS_LOW_LPS_MONOCYTE_DN	184	0.4865736	22.334.375	0.0	0.0	0.0
GSE19888_ADENOSINE_A3R_INH_VS_ACT_IN_MAST_CELL_DN	191	0.4828981	22.306.871	0.0	0.0	0.0
GSE29617_DAY3_VS_DAY7_TIV_FLU_VACCINE_PBMC_2008_UP	173	0.4893639	22.304.456	0.0	0.0	0.0
GSE3982_CTRL_VS_LPS_48H_DC_DN	198	0.4806238	22.296.357	0.0	0.0	0.0
GSE30971_WBP7_HET_VS_KO_MACROPHAGE_2H_LPS_STIM_UP	185	0.48452875	22.260.232	0.0	0.0	0.0
GSE17974_IL4_AND_ANTI_IL12_VS_UNTREATED_12H_ACT_CD4_TCELL_DN	173	0.48827025	22.243.745	0.0	0.0	0.0
GSE18791_CTRL_VS_NEWCASTLE_VIRUS_DC_14H_DN	176	0.48826006	2.223.017	0.0	0.0	0.0
GSE14769_UNSTIM_VS_80MIN_LPS_BMDM_DN	190	0.47820958	22.223.103	0.0	0.0	0.0
GSE3982_DC_VS_CENT_MEMORY_CD4_TCELL_UP	197	0.47506878	22.218.435	0.0	0.0	0.0
GSE29164_UNTREATED_VS_CD8_TCELL_TREATED_MELANOMA_DAY7_UP	197	0.4800279	2.219.797	0.0	0.0	0.0
GSE4590_SMALL_VS_LARGE_PRE_BCELL_UP	170	0.48844194	22.196.152	0.0	0.0	0.0
GSE32164_ALTERNATIVELY_ACT_M2_VS_CMYC_INHIBITED_MACROPHAGE_UP	198	0.4835542	22.189.386	0.0	0.0	0.0
GSE21546_SAP1A_KO_VS_SAP1A_KO_AND_ELK1_KO_ANTI_CD3_STIM_DP_THYMOCYTES_DN	194	0.48071897	22.180.774	0.0	0.0	0.0
GSE16755_CTRL_VS_IFNA_TREATED_MAC_DN	188	0.48534697	22.171.037	0.0	0.0	0.0
GSE9988_LOW_LPS_VS_ANTI_TREM1_AND_LPS_MONOCYTE_UP	183	0.4887804	22.170.942	0.0	0.0	0.0
GSE13547_2H_VS_12_H_ANTI_IGM_STIM_ZFX_KO_BCELL_UP	159	0.488111	22.161.057	0.0	0.0	0.0
GSE17974_CTRL_VS_ACT_IL4_AND_ANTI_IL12_48H_CD4_TCELL_UP	173	0.48593467	22.159.512	0.0	0.0	0.0
GSE30962_PRIMARY_VS_SECONDARY_CHRONIC_LCMV_INF_CD8_TCELL_DN	190	0.47835016	22.120.795	0.0	0.0	0.0
GSE9988_LPS_VS_VEHICLE_TREATED_MONOCYTE_DN	190	0.484151	22.117.474	0.0	0.0	0.0
GSE3920_IFNA_VS_IFNG_TREATED_FIBROBLAST_UP	168	0.4807616	2.209.911	0.0	0.0	0.0
GSE17974_IL4_AND_ANTI_IL12_VS_UNTREATED_24H_ACT_CD4_TCELL_DN	170	0.48804948	22.057.142	0.0	0.0	0.0
GSE9988_ANTI_TREM1_AND_LPS_VS_VEHICLE_TREATED_MONOCYTES_DN	193	0.47671416	22.030.795	0.0	0.0	0.0
GSE22886_TCELL_VS_BCELL_NAIVE_UP	195	0.47741947	22.026.737	0.0	0.0	0.0
GSE369_SOCS3_KO_VS_WT_LIVER_POST_IL6_INJECTION_DN	192	0.47741178	220.075	0.0	0.0	0.0
GSE21063_WT_VS_NFATC1_KO_3H_ANTI_IGM_STIM_BCELL_UP	193	0.47213206	22.003.076	0.0	0.0	0.0
GSE22935_WT_VS_MYD88_KO_MACROPHAGE_48H_MBOVIS_BCG_STIM_DN	195	0.47407776	21.996.305	0.0	0.0	0.0
GSE4984_LPS_VS_VEHICLE_CTRL_TREATED_DC_DN	188	0.48022953	21.990.538	0.0	0.0	0.0
GSE24671_CTRL_VS_SENDAI_VIRUS_INFECTED_MOUSE_SPLENOCYTES_UP	155	0.496528	21.974.323	0.0	0.0	0.0
GSE7460_TCONV_VS_TREG_THYMUS_DN	195	0.4748322	21.970.947	0.0	0.0	0.0
GSE21063_WT_VS_NFATC1_KO_BCELL_UP	195	0.47189608	21.970.177	0.0	0.0	0.0
GSE39916_B_CELL_SPLEEN_VS_PLASMA_CELL_BONE_MARROW_DN	195	0.47380832	21.952.255	0.0	0.0	0.0
GSE13522_WT_VS_IFNAR_KO_SKING_T_CRUZI_Y_STRAIN_INF_UP	194	0.46854785	2.193.062	0.0	0.0	0.0
GSE42021_TCONV_PLN_VS_CD24HI_TCONV_THYMUS_UP	196	0.4730644	21.927.862	0.0	0.0	0.0
GSE24142_EARLY_THYMIC_PROGENITOR_VS_DN2_THYMOCYTE_FETAL_UP	195	0.47525197	21.906.304	0.0	0.0	0.0
GSE42724_MEMORY_VS_B1_BCELL_DN	190	0.47458255	21.883.478	0.0	0.0	0.0
GSE22886_NAIVE_BCELL_VS_NEUTROPHIL_DN	195	0.46569806	2.187.818	0.0	0.0	0.0
GSE25123_CTRL_VS_ROSIGLITAZONE_STIM_PPARG_KO_MACROPHAGE_UP	189	0.4744032	21.876.519	0.0	0.0	0.0
GSE22886_CD8_TCELL_VS_BCELL_NAIVE_UP	194	0.47470498	2.187.426	0.0	0.0	0.0
GSE360_DC_VS_MAC_B_MALAYI_HIGH_DOSE_DN	199	0.46837598	21.842.575	0.0	0.0	0.0
GSE22432_PDC_VS_TGFB1_TREATEDCOMMON_DC_PROGENITOR_DN	191	0.47015235	21.837.525	0.0	0.0	0.0
GSE13484_3H_UNSTIM_VS_YF17D_VACCINE_STIM_PBMC_DN	195	0.4751182	21.821.811	0.0	0.0	0.0
GSE19401_UNSTIM_VS_RETINOIC_ACID_AND_PAM2CSK4_STIM_FOLLICULAR_DC_DN	197	0.472734	21.810.045	0.0	0.0	0.0
GSE21360_TERTIARY_VS_QUATERNARY_MEMORY_CD8_TCELL_DN	171	0.48007688	21.800.985	0.0	0.0	0.0
GSE9988_LPS_VS_LPS_AND_ANTI_TREM1_MONOCYTE_UP	187	0.47249505	21.791.952	0.0	0.0	0.0
GSE34392_ST2_KO_VS_WT_DAY8_LCMV_EFFECTOR_CD8_TCELL_DN	195	0.46834886	21.780.863	0.0	0.0	0.0
GSE360_CTRL_VS_T_GONDII_DC_UP	198	0.4666887	2.175.678	0.0	0.0	0.0
GSE14699_NAIVE_VS_DELETIONAL_TOLERANCE_CD8_TCELL_DN	194	0.47326133	21.742.892	0.0	0.0	0.0

GSE1791_CTRL_VS_NEUROMEDINU_IN_T_CELL_LINE_0.8H_DN	170	0.47760224	2.172.813	0.0	0.0	0.0
GSE7460_CD8_TCELL_VS_TREG_ACT_UP	194	0.474277	21.710.436	0.0	0.0	0.0
GSE40274_CTRL_VS_LEF1_TRANSDUCED_ACTIVATED_CD4_TCELL_UP	167	0.4804063	21.704.316	0.0	0.0	0.0
GSE7219_UNSTIM_VS_LPS_AND_ANTI_CD40_STIM_NIK_NFKB2_KO_DC_UP	175	0.47986063	21.690.826	0.0	0.0	0.0
GSE22886_CD4_TCELL_VS_BCELL_NAIVE_DN	187	0.47396088	21.688.132	0.0	0.0	0.0
GSE7852_TREG_VS_TCONV_LN_UP	195	0.46426073	21.674.128	0.0	0.0	0.0
GSE23568_ID3_TRANSDUCED_VS_ID3_KO_CD8_TCELL_UP	196	0.46800712	2.166.265	0.0	0.0	0.0
GSE13485_DAY3_VS_DAY7_YF17D_VACCINE_PBMC_DN	182	0.4721704	21.660.464	0.0	0.0	0.0
GSE7219_WT_VS_NIK_NFKB2_KO_DC_DN	167	0.47743645	2.165.174	0.0	0.0	0.0
GSE21546_WT_VS_SAP1A_KO_AND_ELK1_KO_DP_THYMOCYTES_DN	192	0.4665538	2.164.322	0.0	0.0	0.0
GSE27670_BLIMP1_VS_LMP1_TRANSDUCED_GC_BCELL_UP	197	0.46826652	21.606.596	0.0	0.0	0.0
GSE2770_IL12_AND_TGFB_VS_IL4_TREATED_ACT_CD4_TCELL_48H_DN	170	0.4744711	2.159.924	0.0	0.0	0.0
GSE6259_FLT3L_INDUCED_DEC205_POS_DC_VS_CD8_TCELL_DN	162	0.47853047	2.159.235	0.0	0.0	0.0
GSE21670_UNTREATED_VS_TGFB_TREATED_STAT3_KO_CD4_TCELL_DN	193	0.46867046	21.588.647	0.0	0.0	0.0
GSE41867_DAY6_EFFECTOR_VS_DAY30_EXHAUSTED_CD8_TCELL_LCMV_CLONE13_UP	188	0.46818286	21.574.066	0.0	0.0	0.0
GSE33424_CD161_HIGH_VS_NEG_CD8_TCELL_UP	197	0.47114053	21.571.949	0.0	0.0	0.0
GSE42021_CD24INT_VS_CD24LOW_TCONV_THYMUS_UP	191	0.47078153	21.571.367	0.0	0.0	0.0
GSE28726_NAIVE_VS_ACTIVATED_CD4_TCELL_UP	195	0.4655389	2.156.519	0.0	0.0	0.0
GSE43863_LY6C_INT_CXCR5POS_VS_LY6C_LOW_CXCR5NEG_EFFECTOR_CD4_TCELL_UP	194	0.4650785	21.554.942	0.0	0.0	0.0
GSE4748_CYANOBACTERIUM_LPSLIKE_VS_LPS_AND_CYANOBACTERIUM_LPSLIKE_STIM_DC_3H_UP	181	0.47153753	2.153.971	0.0	0.0	0.0
GSE39556_CD8A_DC_VS_NK_CELL_MOUSE_3H_POST_POLYIC_INJ_DN	194	0.46534365	21.533.597	0.0	0.0	0.0
GSE26343_UNSTIM_VS_LPS_STIM_NFAT5_KO_MACROPHAGE_DN	196	0.4595451	21.529.872	0.0	0.0	0.0
GSE4984_UNTREATED_VS_GALECTIN1_TREATED_DC_DN	173	0.4712006	21.523.905	0.0	0.0	0.0
GSE1740_MCSF_VS_MCSF_AND_IFNG_DAY2_DERIVED_MACROPHAGE_UP	170	0.47362515	21.511.521	0.0	0.0	0.0
GSE25677_MPL_VS_R848_STIM_BCELL_UP	169	0.47443947	2.150.696	0.0	0.0	0.0
GSE33425_CD8_ALPHAALPHA_VS_ALPHABETA_CD161_HIGH_TCELL_UP	196	0.47060555	21.501.796	0.0	0.0	0.0
GSE21670_STAT3_KO_VS_WT_CD4_TCELL_UP	190	0.47018543	21.494.055	0.0	0.0	0.0
GSE22886_NAIVE_CD8_TCELL_VS_DC_UP	187	0.46787548	21.489.756	0.0	0.0	0.0
GSE40274_CTRL_VS_GATA1_TRANSDUCED_ACTIVATED_CD4_TCELL_UP	127	0.48816493	2.146.635	0.0	0.0	0.0
GSE41867_DAY15_EFFECTOR_VS_DAY30_EXHAUSTED_CD8_TCELL_LCMV_CLONE13_UP	188	0.47005567	21.465.504	0.0	0.0	0.0
GSE34515_CD16_NEG_MONOCYTE_VS_DC_UP	186	0.46577236	21.427.977	0.0	0.0	0.0
GSE32164_RESTING_DIFFERENTIATED_VS_ALTERNATIVELY_ACT_M2_MACROPHAGE_DN	198	0.4607665	2.142.743	0.0	0.0	0.0
GSE21360_NAIVE_VS_SECONDARY_MEMORY_CD8_TCELL_UP	176	0.46843982	21.420.734	0.0	0.0	0.0
GSE13522_CTRL_VS_T_CRUZI_Y_STRAIN_INF_SKIN_IFNAR_KO_UP	190	0.46208292	2.140.957	0.0	0.0	0.0
GSE2128_CTRL_VS_MIMETOPE_NEGATIVE_SELECTION_DP_THYMOCYTE_NOD_UP	190	0.46504503	21.395.655	0.0	0.0	0.0
GSE2770_IL12_AND_TGFB_ACT_VS_ACT_CD4_TCELL_6H_UP	188	0.4622751	2.137.239	0.0	0.0	0.0
GSE2706_UNSTIM_VS_2H_LPS_DC_DN	180	0.4687535	21.368.735	0.0	0.0	0.0
GSE30971_CTRL_VS_LPS_STIM_MACROPHAGE_WBP7_KO_2H_DN	185	0.46115106	21.337.783	0.0	0.0	0.0
GSE23502_BM_VS_COLON_TUMOR_MYELOID_DERIVED_SUPPRESSOR_CELL_UP	195	0.45848188	2.131.461	0.0	0.0	0.0
GSE8621_LPS_STIM_VS_LPS_PRIMED_AND_LPS_STIM_MACROPHAGE_DN	194	0.4562929	21.313.655	0.0	0.0	0.0
GSE13547_WT_VS_ZFX_KO_BCELL_UP	160	0.4696138	21.281.128	0.0	0.0	0.0
GSE24634_TEFF_VS_TCONV_DAY5_IN_CULTURE_DN	190	0.4628396	21.281.085	0.0	0.0	0.0
GSE5679_PPARG_LIGAND_ROSIGLITAZONE_VS_RARA_AGONIST_AM580_TREATED_DC_DN	193	0.46349156	21.280.928	0.0	0.0	0.0
GSE12366_GC_VS_MEMORY_BCELL_DN	189	0.4684872	212.673	0.0	0.0	0.0
GSE34156_UNTREATED_VS_6H_NOD2_AND_TLR1_TLR2_LIGAND_TREATED_MONOCYTE_DN	149	0.48209912	21.262.314	0.0	0.0	0.0
GSE18791_CTRL_VS_NEWCASTLE_VIRUS_DC_4H_DN	180	0.46049988	21.246.026	0.0	0.0	0.0
GSE40274_CTRL_VS_FOXP3_AND_GATA1_TRANSDUCED_ACTIVATED_CD4_TCELL_UP	137	0.48486158	21.243.644	0.0	0.0	0.0
GSE3337_4H_VS_16H_IFNG_IN_CD8POS_DC_DN	193	0.4632178	212.434	0.0	0.0	0.0
GSE2706_UNSTIM_VS_8H_R848_DC_DN	188	0.46095434	21.223.624	0.0	0.0	0.0
GSE30971_WBP7_HET_VS_KO_MACROPHAGE_4H_LPS_STIM_DN	184	0.4629978	21.220.315	0.0	0.0	0.0
GSE42021_CD24HI_VS_CD24INT_TCONV_THYMUS_DN	194	0.45834422	2.121.587	0.0	0.0	0.0
GSE14415_NATURAL_TREG_VS_FOXP3_KO_NATURAL_TREG_UP	144	0.4771732	21.215.653	0.0	0.0	0.0
GSE37532_TREG_VS_TCONV_PPARG_KO_CD4_TCELL_FROM_VISCERAL_ADIPOSE_TISSUE_DN	192	0.45666352	212.127	0.0	0.0	0.0
GSE42021_CD24HI_VS_CD24LOW_TREG_THYMUS_DN	191	0.46491325	21.200.888	0.0	0.0	0.0
GSE3982_BCELL_VS_CENT_MEMORY_CD4_TCELL_UP	189	0.46216774	21.185.474	0.0	0.0	0.0
GSE3982_NKCELL_VS_TH2_UP	186	0.46100783	2.117.391	0.0	0.0	0.0
GSE3982_BCELL_VS_TH2_UP	185	0.46348515	21.168.938	0.0	0.0	0.0
GSE7219_UNSTIM_VS_LPS_AND_ANTI_CD40_STIM_DC_DN	173	0.46062514	21.156.824	0.0	0.0	0.0
GSE37534_UNTREATED_VS_PIOGLITAZONE_TREATED_CD4_TCELL_PPARG1_AND_FOXP3_TRASDUCED_DN	195	0.461455	21.126.704	0.0	0.0	0.0
GSE34156_NOD2_LIGAND_VS_TLR1_TLR2_LIGAND_6H_TREATED_MONOCYTE_UP	194	0.45730186	21.124.814	0.0	0.0	0.0
GSE19198_CTRL_VS_IL21_TREATED_TCELL_6H_UP	195	0.4563297	21.117.492	0.0	0.0	0.0
GSE12392_WT_VS_IFNB_KO_CD8A_NEG_SPLEEN_DC_DN	192	0.46166265	21.110.554	0.0	0.0	0.0
GSE24142_EARLY_THYMIC_PROGENITOR_VS_DN3_THYMOCYTE_UP	197	0.45496547	21.107.428	0.0	0.0	0.0
GSE43863_TFH_VS_LY6C_LOW_CXCR5NEG_EFFECTOR_CD4_TCELL_UP	193	0.4582991	21.098.537	0.0	0.0	0.0
GSE2770_TGFB_AND_IL4_ACT_VS_ACT_CD4_TCELL_2H_DN	189	0.46565863	21.094.227	0.0	0.0	0.0
GSE22886_NAIVE_CD4_TCELL_VS_DC_UP	184	0.46095458	21.085.207	0.0	0.0	0.0
GSE20366_TREG_VS_NAIVE_CD4_TCELL_DN	192	0.45458943	21.059.744	0.0	0.0	0.0
GSE11057_PBMC_VS_MEM_CD4_TCELL_DN	186	0.45783532	2.105.186	0.0	0.0	0.0
GSE9988_ANTI_TREM1_VS_LPS_MONOCYTE_DN	184	0.45955306	2.104.928	0.0	0.0	0.0
GSE360_T_GONDII_VS_B_MALAYI_HIGH_DOSE_DC_UP	189	0.46017587	2.102.398	0.0	0.0	0.0
GSE36888_UNTREATED_VS_IL2_TREATED_TCELL_17H_UP	196	0.45376098	21.003.816	0.0	0.0	0.0
GSE36476_CTRL_VS_TSST_ACT_16H_MEMORY_CD4_TCELL_YOUNG_UP	191	0.45332247	20.999.799	0.0	0.0	0.0
GSE2405_HEAT_KILLED_LYSATE_VS_LIVE_A_PHAGOCYTOPHILUM_STIM_NEUTROPHIL_9H_DN	196	0.4543881	2.099.699	0.0	0.0	0.0
GSE9988_ANTI_TREM1_VS_CTRL_TREATED_MONOCYTES_DN	189	0.45436507	20.993.185	0.0	0.0	0.0
GSE14415_INDUCED_TREG_VS_FOXP3_KO_INDUCED_TREG_DN	178	0.4563992	20.975.883	0.0	0.0	0.0
GSE35825_UNTREATED_VS_IFNG_STIM_MACROPHAGE_UP	195	0.45036992	2.097.396	0.0	0.0	0.0
GSE3039_NKT_CELL_VS_B2_BCELL_UP	197	0.44614297	2.094.454	0.0	0.0	0.0
GSE45365_NK_CELL_VS_CD11B_DC_MCMV_INFECTION_DN	196	0.45014536	20.941.217	0.0	0.0	0.0
GSE37301_MULTIPOTENT_PROGENITOR_VS_CD4_TCELL_UP	166	0.46033624	20.938.733	0.0	0.0	0.0
GSE21546_ELK1_KO_VS_SAP1A_KO_AND_ELK1_KO_DP_THYMOCYTES_UP	178	0.4575959	20.936.275	0.0	0.0	0.0
GSE360_L_DONOVANI_VS_M_TUBERCULOSIS_DC_DN	194	0.4535694	20.925.353	0.0	0.0	0.0
GSE34205_RSV_VS_FLU_INF_INFANT_PBMC_DN	184	0.4593058	20.921.464	0.0	0.0	0.0
GSE5099_CLASSICAL_M1_VS_ALTERNATIVE_M2_MACROPHAGE_UP	185	0.4541009	20.917.306	0.0	0.0	0.0
GSE21774_CD62L_POS_CD56_DIM_VS_CD62L_NEG_CD56_DIM_NK_CELL_UP	196	0.45334068	2.091.185	0.0	0.0	0.0
GSE6674_UNSTIM_VS_CPG_STIM_BCELL_UP	137	0.47130883	20.907.512	0.0	0.0	0.0
GSE23568_ID3_TRANSDUCED_VS_ID3_KO_CD8_TCELL_DN	196	0.45340455	20.898.037	0.0	0.0	0.0
GSE9509_LPS_VS_LPS_AND_IL10_STIM_IL10_KO_MACROPHAGE_10MIN_UP	193	0.4511753	20.886.018	0.0	0.0	0.0
GSE19198_1H_VS_24H_IL21_TREATED_TCELL_UP	196	0.45656258	20.883.815	0.0	0.0	0.0
GSE39382_IL3_VS_IL3_IL33_TREATED_MAST_CELL_DN	191	0.450208	20.877.664	0.0	0.0	0.0

KAECH_DAY15_EFF_VS_MEMORY_CD8_TCELL_UP		194	0.44558233	20.877.185	0.0	0.0	0.0
GSE41867_NAIVE_VS_DAY6_LCMV_ARMSTRONG_EFFECTOR_CD8_TCELL_DN		193	0.45052204	20.876.858	0.0	0.0	0.0
GSE7852_TREG_VS_TCONV_THYMUS_UP		195	0.45390266	2.087.588	0.0	0.0	0.0
GSE13485_DAY7_VS_DAY21_YF17D_VACCINE_PBMC_UP		187	0.45222425	20.861.917	0.0	0.0	0.0
GSE21546_WT_VS_SAP1A_KO_AND_ELK1_KO_ANTI_CD3_STIM_DP_THYMOCYTES_UP		184	0.45588583	20.861.037	0.0	0.0	0.0
GSE5589_WT_VS_IL6_KO_LPS_AND_IL6_STIM_MACROPHAGE_45MIN_UP		190	0.45223016	20.853.097	0.0	0.0	0.0
GSE2405_0H_VS_3H_A_PHAGOCYTOPHILUM_STIM_NEUTROPHIL_UP		193	0.45317912	20.840.805	0.0	0.0	0.0
GSE32164_RESTING_DIFFERENTIATED_VS_CMYC_INHIBITED_MACROPHAGE_UP		199	0.4461875	20.840.354	0.0	0.0	0.0
GSE30083_SP1_VS_SP4_THYMOCYTE_DN		193	0.4518424	20.836.482	0.0	0.0	0.0
GSE23925_LIGHT_ZONE_VS_NAIVE_BCELL_UP		190	0.45111704	20.833.707	0.0	0.0	0.0
GSE37533_PPARG1_FOXP3_VS_FOXP3_TRANSDUCED_CD4_TCELL_PIOGLITAZONE_TREATED_UP		194	0.45320398	20.819.492	0.0	0.0	0.0
GSE37301_HEMATOPOIETIC_STEM_CELL_VS_CD4_TCELL_UP		170	0.4573183	20.818.224	0.0	0.0	0.0
GSE1460_INTRATHYMIC_T_PROGENITOR_VS_DP_THYMOCYTE_DN		189	0.4497712	2.081.552	0.0	0.0	0.0
GSE24026_PD1_LIGATION_VS_CTRL_IN_ACT_TCELL_LINE_UP		194	0.45674065	20.811.007	0.0	0.0	0.0
GSE14415_NATURAL_TREG_VS_TCONV_UP		147	0.46249485	2.077.071	0.0	0.0	0.0
GSE2405_HEAT_KILLED_LYSATE_VS_LIVE_A_PHAGOCYTOPHILUM_STIM_NEUTROPHIL_24H_UP		194	0.44795072	2.076.366	0.0	0.0	0.0
GSE17721_0.5H_VS_24H_CPG_BMDM_DN		197	0.44298446	2.075.196	0.0	0.0	0.0
GSE45739_UNSTIM_VS_ACD3_ACD28_STIM_WT_CD4_TCELL_UP		189	0.44829944	20.737.329	0.0	0.0	0.0
GSE11961_MEMORY_BCELL_DAY7_VS_GERMINAL_CENTER_BCELL_DAY7_DN		194	0.45054126	20.729.072	0.0	0.0	0.0
GSE11057_NAIVE_VS_CENT_MEMORY_CD4_TCELL_DN		190	0.4471146	207.162	0.0	0.0	0.0
GSE7219_UNSTIM_VS_LPS_AND_ANTI_CD40_STIM_NIK_NFKB2_KO_DC_DN		187	0.45405355	2.071.588	0.0	0.0	0.0
GSE42088_2H_VS_24H_LEISHMANIA_INF_DC_UP		194	0.44933408	20.713.453	0.0	0.0	0.0
GSE22432_UNTREATED_VS_TGFB1_TREATED_COMMON_DC_PROGENITOR_DN		191	0.44961032	20.710.695	0.0	0.0	0.0
GSE3982_MAST_CELL_VS_NKCELL_DN		194	0.44724602	20.709.798	0.0	0.0	0.0
GSE43863_DAY6_EFF_VS_DAY150_MEM_TH1_CD4_TCELL_DN		193	0.44404984	2.067.623	0.0	0.0	0.0
GSE22886_NAIVE_BCELL_VS_DC_UP		191	0.4480627	2.067.546	0.0	0.0	0.0
GSE30083_SP2_VS_SP3_THYMOCYTE_DN		195	0.45243165	20.671.806	0.0	0.0	0.0
GSE1791_CTRL_VS_NEUROMEDINU_IN_T_CELL_LINE_3H_UP		163	0.45560387	20.667.973	0.0	0.0	0.0
GSE45739_UNSTIM_VS_ACD3_ACD28_STIM_WT_CD4_TCELL_DN		195	0.44814634	20.661.094	0.0	0.0	0.0
GSE40274_HELIOS_VS_FOXP3_AND_HELIOS_TRANSDUCED_ACTIVATED_CD4_TCELL_UP		193	0.4444372	2.066.041	0.0	0.0	0.0
GSE21063_CTRL_VS_ANTI_IGM_STIM_BCELL_NFATC1_KO_16H_UP		192	0.44915915	20.647.511	0.0	0.0	0.0
GSE22886_IGG_IGA_MEMORY_BCELL_VS_BLOOD_PLASMA_CELL_UP		197	0.44836056	2.064.426	0.0	0.0	0.0
GSE1460_INTRATHYMIC_T_PROGENITOR_VS_NAIVE_CD4_TCELL_ADULT_BLOOD_DN		185	0.44893295	20.643.873	0.0	0.0	0.0
GSE21063_CTRL_VS_ANTI_IGM_STIM_BCELL_8H_UP		150	0.45818314	20.640.182	0.0	0.0	0.0
GSE6674_ANTI_IGM_VS_ANTI_IGM_AND_CPG_STIM_BCELL_UP		189	0.44788748	20.629.385	0.0	0.0	0.0
GSE7219_WT_VS_NIK_NFKB2_KO_LPS_AND_ANTI_CD40_STIM_DC_DN		194	0.44851354	20.628.855	0.0	0.0	0.0
GSE37301_PRO_BCELL_VS_CD4_TCELL_DN		157	0.45947337	2.062.446	0.0	0.0	0.0
GSE45365_NK_CELL_VS_CD11B_DC_DN		197	0.44472128	2.060.073	0.0	0.0	0.0
GSE37416_CTRL_VS_48H_F_TULARENSIS_LVS_NEUTROPHIL_UP		177	0.4483629	20.599.785	0.0	0.0	0.0
GSE43955_1H_VS_20H_ACT_CD4_TCELL_WITH_TGFB_IL6_DN		193	0.4473409	20.599.017	0.0	0.0	0.0
GSE25123_IL4_VS_IL4_AND_ROSIGLITAZONE_STIM_MACROPHAGE_DAY10_UP		195	0.44297323	20.598.228	0.0	0.0	0.0
GSE41867_DAY6_VS_DAY15_LCMV_ARMSTRONG_EFFECTOR_CD8_TCELL_UP		176	0.4528457	2.059.738	0.0	0.0	0.0
GSE43863_NAIVE_VS_MEMORY_LY6C_INT_CXCR5POS_CD4_TCELL_D150_LCMV_UP		188	0.44945306	2.056.053	0.0	0.0	0.0
GSE13946_CTRL_VS_DSS_COLITIS_GD_TCELL_FROM_COLON_UP		174	0.45600298	20.557.318	0.0	0.0	0.0
GSE7831_1H_VS_4H_INFLUENZA_STIM_PDC_DN		191	0.45013076	20.554.721	0.0	0.0	0.0
GSE17974_0H_VS_48H_IN_VITRO_ACT_CD4_TCELL_UP		176	0.44982123	20.547.733	0.0	0.0	0.0
GSE7852_TREG_VS_TCONV_DN		191	0.44402078	20.544.589	0.0	0.0	0.0
GSE34515_CD16_POS_MONOCYTE_VS_DC_DN		189	0.4496385	20.544.155	0.0	0.0	0.0
GSE5455_HEALTHY_VS_TUMOR_BEARING_MOUSE_SPLEEN_MONOCYTE_DN		193	0.44237763	20.543.885	0.0	0.0	0.0
GSE7219_WT_VS_NIK_NFKB2_KO_DC_UP		181	0.44770488	20.534.914	0.0	0.0	0.0
GSE2706_2H_VS_8H_LPS_STIM_DC_DN		185	0.4475334	20.528.634	0.0	0.0	0.0
GSE12392_WT_VS_IFNAR_KO_CD8A_NEG_SPLEEN_DC_UP		194	0.4465973	20.528.085	0.0	0.0	0.0
GSE17186_MEMORY_VS_CD21HIGH_TRANSITIONAL_BCELL_DN		194	0.4459979	2.052.343	0.0	0.0	0.0
GSE27291_0H_VS_7D_STIM_GAMMADELTA_TCELL_DN		180	0.44623002	20.519.323	0.0	0.0	0.0
GSE20715_0H_VS_48H_OZONE_LUNG_UP		195	0.44428232	2.051.924	0.0	0.0	0.0
GSE43863_DAY6_EFF_VS_DAY150_MEM_TH1_CD4_TCELL_UP		196	0.44080287	20.515.847	0.0	0.0	0.0
GSE29164_UNTREATED_VS_CD8_TCELL_TREATED_MELANOMA_DAY3_DN		195	0.43958235	20.512.714	0.0	0.0	0.0
GSE16266_CTRL_VS_LPS_STIM_MEF_UP		185	0.44489238	20.508.726	0.0	0.0	0.0
GSE37301_HEMATOPOIETIC_STEM_CELL_VS_RAG2_KO_NK_CELL_DN		177	0.4521925	20.507.765	0.0	0.0	0.0
GSE360_DC_VS_MAC_M_TUBERCULOSIS_UP		191	0.444108	20.504.224	0.0	0.0	0.0
GSE21063_CTRL_VS_ANTI_IGM_STIM_BCELL_3H_UP		171	0.44946775	20.501.254	0.0	0.0	0.0
GSE9650_NAIVE_VS_EFF_CD8_TCELL_DN		194	0.4439116	20.500.975	0.0	0.0	0.0
GSE40068_CXCR5NEG_BCL6NEG_CD4_TCELL_VS_CXCR5POS_BCL6NEG_TFH_DN		193	0.4424583	20.500.243	0.0	0.0	0.0
GSE22886_NAIVE_BCELL_VS_MONOCYTE_DN		197	0.4425655	20.486.388	0.0	0.0	0.0
GSE360_DC_VS_MAC_T_GONDII_UP		193	0.4425484	2.047.673	0.0	0.0	0.0
GSE11057_NAIVE_VS_MEMORY_CD4_TCELL_DN		191	0.4527416	20.455.735	0.0	0.0	0.0
GSE2706_UNSTIM_VS_2H_R848_DC_DN		182	0.44487685	20.448.515	0.0	0.0	0.0
GSE7509_DC_VS_MONOCYTE_WITH_FCGRIIB_STIM_DN		193	0.44363925	204.462	0.0	0.0	0.0
GSE41176_WT_VS_TAK1_KO_ANTI_IGM_STIM_BCELL_6H_DN		200	0.43797353	20.436.916	0.0	0.0	0.0
GSE360_L_DONOVANI_VS_M_TUBERCULOSIS_MAC_DN		193	0.4453784	20.435.236	0.0	0.0	0.0
GSE14415_INDUCED_TREG_VS_FAILED_INDUCED_TREG_DN		175	0.45013243	20.418.634	0.0	0.0	0.0
GSE42021_TREG_PLN_VS_CD24LO_TREG_THYMUS_DN		193	0.4418102	204.177	0.0	0.0	0.0
GSE14386_UNTREATED_VS_IFNA_TREATED_ACT_PBMC_MS_PATIENT_DN		148	0.45518538	20.409.095	0.0	0.0	0.0
GSE7460_TCONV_VS_TREG_LN_DN		198	0.44347525	20.407.822	0.0	0.0	0.0
GSE36527_CD69_NEG_VS_POS_TREG_CD62L_LOS_KLRG1_NEG_UP		195	0.4410229	20.397.542	0.0	0.0	0.0
GSE3720_UNSTIM_VS_LPS_STIM_VD1_GAMMADELTA_TCELL_DN		140	0.46358913	20.396.755	0.0	0.0	0.0
GSE13306_RA_VS_UNTREATED_MEM_CD4_TCELL_UP		189	0.4410889	2.039.373	0.0	0.0	0.0
GSE24210_CTRL_VS_IL35_TREATED_TCONV_CD4_TCELL_UP		192	0.44267505	20.386.112	0.0	0.0	0.0
GSE10325_CD4_TCELL_VS_LUPUS_CD4_TCELL_DN		194	0.43912363	2.037.416	0.0	0.0	0.0
GSE10325_MYELOID_VS_LUPUS_MYELOID_DN		195	0.44121033	20.369.072	0.0	0.0	0.0
GSE34156_UNTREATED_VS_6H_NOD2_LIGAND_TREATED_MONOCYTE_DN		167	0.45393956	20.356.464	0.0	0.0	0.0
GSE7219_UNSTIM_VS_LPS_AND_ANTI_CD40_STIM_DC_UP		171	0.44427952	20.351.448	0.0	0.0	0.0
GSE23502_BM_VS_COLON_TUMOR_MYELOID_DERIVED_SUPPRESSOR_CELL_DN		194	0.44461697	20.350.626	0.0	0.0	0.0
GSE15735_CTRL_VS_HDAC_INHIBITOR_TREATED_CD4_TCELL_2H_DN		194	0.43764085	20.337.384	0.0	0.0	0.0
GSE21360_NAIVE_VS_PRIMARY_MEMORY_CD8_TCELL_UP		167	0.44926286	20.334.094	0.0	0.0	0.0
GSE3982_MAST_CELL_VS_BCELL_DN		189	0.44140524	20.328.681	0.0	0.0	0.0
GSE20366_TREG_VS_NAIVE_CD4_TCELL_HOMEOSTATIC_CONVERSION_DN		191	0.43434587	20.323.982	0.0	0.0	0.0
GSE7348_LPS_VS_TOLERIZED_AND_LPS_STIM_MACROPHAGE_DN		168	0.44473553	2.030.799	0.0	0.0	0.0
GSE9988_LOW_LPS_VS_VEHICLE_TREATED_MONOCYTE_DN		189	0.4403302	20.299.182	0.0	0.0	0.0

**GSE3982_MAST_CELL_VS_DC_DN**	191	0.4399746	20.284.457	0.0	0.0	0.0
**GSE19401_NAIVE_VS_IMMUNIZED_MOUSE_PLN_FOLLICULAR_DC_DN**	195	0.4410898	20.280.874	0.0	0.0	0.0
**GSE24142_ADULT_VS_FETAL_EARLY_THYMIC_PROGENITOR_UP**	193	0.43773144	2.027.882	0.0	0.0	0.0
**GSE23568_CTRL_TRANSDUCED_VS_WT_CD8_TCELL_UP**	199	0.43693936	2.027.783	0.0	0.0	0.0
**GSE22886_IGM_MEMORY_BCELL_VS_BLOOD_PLASMA_CELL_UP**	188	0.43647307	20.254.493	0.0	0.0	0.0
**GSE2128_CTRL_VS_MIMETOPE_NEGATIVE_SELECTION_DP_THYMOCYTE_C57BL6_UP**	175	0.43829265	20.253.222	0.0	0.0	0.0
**GSE360_LOW_DOSE_B_MALAYI_VS_M_TUBERCULOSIS_DC_DN**	198	0.43607864	2.024.516	0.0	0.0	0.0
**GSE42021_CD24INT_VS_CD24LOW_TREG_THYMUS_DN**	191	0.43685392	2.023.531	0.0	0.0	0.0
**GSE30962_ACUTE_VS_CHRONIC_LCMV_PRIMARY_INF_CD8_TCELL_UP**	194	0.43921724	20.220.447	0.0	0.0	0.0
**GSE5589_LPS_VS_LPS_AND_IL10_STIM_MACROPHAGE_180MIN_UP**	191	0.438998	2.021.322	0.0	0.0	0.0
**GSE21360_NAIVE_VS_SECONDARY_MEMORY_CD8_TCELL_DN**	166	0.4478688	20.213.006	0.0	0.0	0.0
**GSE3039_B2_VS_B1_BCELL_UP**	197	0.43568772	20.160.732	0.0	0.0	0.0
**GSE3920_UNTREATED_VS_IFNA_TREATED_ENDOTHELIAL_CELL_UP**	170	0.44271624	20.139.244	0.0	0.0	0.0
**GSE13522_WT_VS_IFNAR_KO_SKIN_DN**	174	0.44584084	20.136.712	0.0	0.0	0.0
**GSE37605_FOXP3_FUSION_GFP_VS_IRES_GFP_TREG_C57BL6_DN**	171	0.44038007	20.121.055	0.0	0.0	0.0
**GSE32164_RESTING_DIFFERENTIATED_VS_CMYC_INHIBITED_MACROPHAGE_DN**	197	0.4322675	20.115.807	0.0	0.0	0.0
**GSE37532_WT_VS_PPARG_KO_VISCERAL_ADIPOSE_TISSUE_TCONV_UP**	191	0.43821415	20.101.616	0.0	0.0	0.0
**GSE22589_HEALTHY_VS_HIV_INFECTED_DC_DN**	193	0.4356473	20.080.664	0.0	0.0	0.0
GSE37301_CD4_TCELL_VS_GRANULOCYTE_MONOCYTE_PROGENITOR_DN	177	0.43473342	1.978.925	0.0	1,92E-01	0.001
GSE5589_WT_VS_IL10_KO_LPS_STIM_MACROPHAGE_180MIN_DN	149	0.42183182	18.923.421	0.0	3,15E-01	0.002
GSE3982_CENT_MEMORY_CD4_TCELL_VS_NKCELL_DN	196	0.40845618	18.899.952	0.0	4,72E-01	0.003
GSE17301_CTRL_VS_48H_ACD3_ACD28_STIM_CD8_TCELL_DN	196	0.4109751	18.835.075	0.0	6,19E-01	0.004
GSE45365_NK_CELL_VS_CD8_TCELL_MCMV_INFECTION_DN	195	0.40292332	18.661.779	0.0	7,44E-01	0.005
GSE46606_DAY1_VS_DAY3_CD40L_IL2_IL5_STIMULATED_BCELL_UP	167	0.40951663	18.672.168	0.0	7,47E-01	0.005
GSE2706_LPS_VS_R848_AND_LPS_8H_STIM_DC_UP	185	0.40310547	18.553.598	0.0	1,61E+00	0.011
GSE35825_UNTREATED_VS_IFNA_STIM_MACROPHAGE_DN	162	0.41851997	19.184.349	0.0	1,69E+00	0.001
GSE19941_UNSTIM_VS_LPS_STIM_IL10_KO_MACROPHAGE_UP	192	0.39954573	18.477.964	0.0	1,71E+00	0.012
GSE15733_BM_VS_SPLEEN_MEMORY_CD4_TCELL_UP	196	0.41559318	19.316.334	0.0	1,74E+00	0.001
GSE19825_NAIVE_VS_IL2RAHIGH_DAY3_EFF_CD8_TCELL_UP	188	0.42164892	19.346.857	0.0	1,75E+00	0.001
GSE37416_0H_VS_48H_F_TULARENSIS_LVS_NEUTROPHIL_UP	192	0.4199878	1.935.523	0.0	1,76E+00	0.001
GSE3982_BCELL_VS_TH1_UP	187	0.41485825	19.356.929	0.0	1,76E+00	0.001
GSE5542_UNTREATED_VS_IFNA_TREATED_EPITHELIAL_CELLS_24H_UP	194	0.42210215	19.436.531	0.0	1,78E+00	0.001
GSE19198_CTRL_VS_IL21_TREATED_TCELL_6H_DN	189	0.42894372	19.667.516	0.0	1,87E+00	0.001
GSE14000_UNSTIM_VS_16H_LPS_DC_DN	190	0.42653394	19.680.612	0.0	1,88E+00	0.001
GSE26030_TH1_VS_TH17_DAY15_POST_POLARIZATION_DN	195	0.4287299	19.765.271	0.0	1,91E+00	0.001
GSE40274_CTRL_VS_FOXP3_AND_IRF4_TRANSDUCED_ACTIVATED_CD4_TCELL_UP	149	0.44266513	19.811.913	0.0	1,96E+00	0.001
GSE38681_WT_VS_LYL1_KO_LYMPHOID_PRIMED_MULTIPOTENT_PROGENITOR_DN	196	0.4291428	19.894.865	0.0	1,99E+00	0.001
GSE29618_MONOCYTE_VS_PDC_DAY7_FLU_VACCINE_DN	195	0.3742444	17.298.957	0.0	2,76E+00	0.216
GSE12366_NAIVE_VS_MEMORY_BCELL_DN	185	0.41266313	18.956.839	0.0	3,17E+00	0.002
GSE10240_IL22_VS_IL22_AND_IL17_STIM_PRIMARY_BRONCHIAL_EPITHELIAL_CELLS_DN	191	0.41246393	18.957.907	0.0	3,18E+00	0.002
GSE29618_PDC_VS_MDC_UP	193	0.40557536	18.958.138	0.0	3,19E+00	0.002
GSE46606_IRF4HIGH_VS_WT_CD40L_IL2_IL5_DAY3_STIMULATED_BCELL_DN	192	0.41576797	18.998.034	0.0	3,22E+00	0.002
GSE3982_NEUTROPHIL_VS_CENT_MEMORY_CD4_TCELL_UP	196	0.40852186	19.011.192	0.0	3,23E+00	0.002
GSE2706_R848_VS_R848_AND_LPS_2H_STIM_DC_DN	174	0.41600257	19.012.545	0.0	3,24E+00	0.002
GSE2706_2H_VS_8H_R848_STIM_DC_DN	181	0.41663915	1.903.317	0.0	3,24E+00	0.002
GSE13411_NAIVE_BCELL_VS_PLASMA_CELL_UP	183	0.41764247	19.114.596	0.0	3,29E+00	0.002
GSE41867_NAIVE_VS_DAY6_LCMV_EFFECTOR_CD8_TCELL_DN	192	0.41423753	19.121.825	0.0	3,31E+00	0.002
GSE41867_NAIVE_VS_DAY6_LCMV_EFFECTOR_CD8_TCELL_UP	190	0.41640675	19.122.186	0.0	3,31E+00	0.002
GSE37605_FOXP3_FUSION_GFP_VS_IRES_GFP_TREG_NOD_DN	166	0.41700998	19.124.554	0.0	3,32E+00	0.002
GSE28783_ANTI_MIR33_VS_UNTREATED_ATHEROSCLEROSIS_MACROPHAGE_DN	193	0.4166548	1.917.121	0.0	3,35E+00	0.002
GSE37416_12H_VS_24H_F_TULARENSIS_LVS_NEUTROPHIL_UP	188	0.39404458	18.261.119	0.0	3,61E+00	0.027
GSE2405_0H_VS_1.5H_A_PHAGOCYTOPHILUM_STIM_NEUTROPHIL_UP	192	0.4094202	18.891.547	0.0	4,70E+00	0.003
GSE22601_IMMATURE_CD4_SINGLE_POSITIVE_VS_CD8_SINGLE_POSITIVE_THYMOCYTE_DN	193	0.40385696	18.898.504	0.0	4,71E+00	0.003
GSE38304_MYC_NEG_VS_POS_GC_BCELL_DN	197	0.38708577	18.013.325	0.0	5,77E+00	0.042
GSE25087_TREG_VS_TCONV_ADULT_UP	187	0.41192222	18.782.989	0.0	6,09E+00	0.004
GSE24634_IL4_VS_CTRL_TREATED_NAIVE_CD4_TCELL_DAY10_UP	194	0.40781584	18.787.631	0.0	6,11E+00	0.004
GSE9960_HEALTHY_VS_GRAM_POS_SEPSIS_PBMC_DN	186	0.409392	18.795.303	0.0	6,14E+00	0.004
GSE39820_IL1B_IL6_VS_IL1B_IL6_IL23A_TREATED_CD4_TCELL_UP	189	0.40973893	18.821.831	0.0	6,17E+00	0.004
GSE45365_HEALTHY_VS_MCMV_INFECTION_CD8A_DC_IFNAR_KO_DN	196	0.40522677	18.832.173	0.0	6,18E+00	0.004
GSE27859_MACROPHAGE_VS_CD11C_INT_F480_INT_DC_UP	164	0.39356166	17.936.472	0.0	6,61E+00	0.05
GSE18893_TCONV_VS_TREG_24H_CULTURE_DN	195	0.39969933	18.651.206	0.0	7,40E+00	0.005
GSE11057_CD4_CENT_MEM_VS_PBMC_UP	190	0.40162906	18.657.885	0.0	7,42E+00	0.005
GSE30083_SP1_VS_SP3_THYMOCYTE_DN	192	0.404932	18.668.941	0.0	7,46E+00	0.005
GSE3982_DC_VS_CENT_MEMORY_CD4_TCELL_DN	195	0.40652278	18.691.642	0.0	7,49E+00	0.005
GSE45739_UNSTIM_VS_ACD3_ACD28_STIM_NRAS_KO_CD4_TCELL_UP	191	0.40400827	18.721.552	0.0	7,52E+00	0.005
GSE3691_CONVENTIONAL_VS_PLASMACYTOID_DC_SPLEEN_UP	196	0.40545902	18.725.011	0.0	7,53E+00	0.005
GSE17721_CTRL_VS_POLYIC_4H_BMDM_DN	193	0.40582362	18.735.211	0.0	7,54E+00	0.005
GSE339_CD4POS_VS_CD8POS_DC_UP	197	0.40519258	18.742.645	0.0	7,56E+00	0.005
GSE24142_EARLY_THYMIC_PROGENITOR_VS_DN3_THYMOCYTE_FETAL_UP	197	0.40613893	18.753.153	0.0	7,57E+00	0.005
GSE9316_IL6_KO_VS_IFNG_KO_INVIVO_EXPANDED_CD4_TCELL_UP	195	0.40355873	18.623.713	0.0	8,84E+00	0.006
GSE22589_SIV_VS_HIV_AND_SIV_INFECTED_DC_UP	194	0.40235302	18.644.093	0.0	8,85E+00	0.006
GSE27241_CTRL_VS_DIGOXIN_TREATED_CD4_TCELL_IN_TH17_POLARIZING_CONDITIONS_DN	188	0.40358838	18.610.165	0.0	1,03E+01	0.007
GSE5542_UNTREATED_VS_IFNA_AND_IFNG_TREATED_EPITHELIAL_CELLS_6H_DN	187	0.3996698	18.549.106	0.0	1,61E+01	0.011
GSE3982_DC_VS_EFF_MEMORY_CD4_TCELL_UP	191	0.41590732	19.189.295	0.0	1,69E+01	0.001
GSE3982_DC_VS_BCELL_UP	194	0.42042544	19.194.164	0.0	1,70E+01	0.001
GSE20715_0H_VS_24H_OZONE_TLR4_KO_LUNG_UP	192	0.4140944	19.197.625	0.0	1,70E+01	0.001
GSE14699_DELETIONAL_TOLERANCE_VS_ACTIVATED_CD8_TCELL_UP	176	0.41584125	19.203.142	0.0	1,70E+01	0.001
GSE13522_CTRL_VS_T_CRUZI_Y_STRAIN_INF_SKIN_IFNAR_KO_DN	193	0.41287553	19.210.421	0.0	1,70E+01	0.001
GSE36476_CTRL_VS_TSST_ACT_40H_MEMORY_CD4_TCELL_OLD_UP	188	0.41390187	19.224.367	0.0	1,71E+01	0.001
GSE40274_XBP1_VS_FOXP3_AND_XBP1_TRANSDUCED_ACTIVATED_CD4_TCELL_UP	197	0.4129054	19.234.641	0.0	1,71E+01	0.001
GSE17580_UNINFECTED_VS_S_MANSONI_INF_TEFF_DN	194	0.41730204	19.238.007	0.0	1,71E+01	0.001
GSE5455_EX_VIVO_VS_POST_24H_INCUBATION_MONOCYTES_FROM_TUMOR_BEARING_MOUSE_DN	196	0.41678992	19.240.035	0.0	1,72E+01	0.001
GSE22432_CDC_VS_COMMON_DC_PROGENITOR_UP	196	0.4137191	19.243.811	0.0	1,72E+01	0.001
GSE21670_TGFB_VS_IL6_TREATED_STAT3_KO_CD4_TCELL_DN	193	0.42334303	19.279.047	0.0	1,72E+01	0.001
GSE43863_TFH_VS_LY6C_LOW_CXCR5NEG_EFFECTOR_CD4_TCELL_DN	196	0.41495237	19.282.951	0.0	1,73E+01	0.001
GSE360_L_DONOVANI_VS_B_MALAYI_HIGH_DOSE_MAC_DN	197	0.41932404	19.294.554	0.0	1,73E+01	0.001
GSE17974_0H_VS_4H_IN_VITRO_ACT_CD4_TCELL_UP	177	0.41910824	19.298.744	0.0	1,73E+01	0.001
GSE21360_SECONDARY_VS_QUATERNARY_MEMORY_CD8_TCELL_UP	174	0.42661306	19.310.918	0.0	1,73E+01	0.001
GSE25146_UNSTIM_VS_HELIOBACTER_PYLORI_LPS_STIM_AGS_CELL_DN	160	0.38944635	17.559.851	0.0	1,73E+01	0.133

GSE14415_INDUCED_TREG_VS_FOXP3_KO_INDUCED_TREG_IL2_CULTURE_DN	122	0.44699293	19.312.632	0.0	1,74E+01	0.001
GSE23114_PERITONEAL_CAVITY_B1A_BCELL_VS_SPLEEN_BCELL_IN_SLE2C1_MOUSE_UP	196	0.41561565	19.320.871	0.0	1,74E+01	0.001
GSE18281_CORTICAL_VS_MEDULLARY_THYMOCYTE_UP	197	0.41914338	19.343.455	0.0	1,75E+01	0.001
GSE14000_4H_VS_16H_LPS_DC_TRANSLATED_RNA_UP	189	0.41751036	19.350.033	0.0	1,75E+01	0.001
GSE24574_NAIVE_VS_TCONV_CD4_TCELL_UP	195	0.41737273	19.360.598	0.0	1,76E+01	0.001
GSE40274_FOXP3_VS_FOXP3_AND_IRF4_TRANSDUCED_ACTIVATED_CD4_TCELL_DN	190	0.41905516	19.386.299	0.0	1,77E+01	0.001
GSE46606_UNSTIM_VS_CD40L_IL2_IL5_1DAY_STIMULATED_IRF4_KO_BCELL_UP	193	0.4251358	19.396.238	0.0	1,77E+01	0.001
GSE6875_TCONV_VS_FOXP3_KO_TREG_UP	192	0.41729772	19.396.989	0.0	1,77E+01	0.001
GSE10240_CTRL_VS_IL22_STIM_PRIMARY_BRONCHIAL_EPITHELIAL_CELLS_UP	192	0.41981164	19.402.821	0.0	1,78E+01	0.001
GSE37532_WT_VS_PPARG_KO_LN_TCONV_UP	191	0.42045024	19.417.205	0.0	1,78E+01	0.001
GSE7852_LN_VS_THYMUS_TREG_UP	190	0.42056668	19.431.235	0.0	1,78E+01	0.001
GSE4590_PRE_BCELL_VS_VPREB_POS_LARGE_PRE_BCELL_UP	150	0.4375535	19.438.677	0.0	1,79E+01	0.001
GSE1460_INTRATHYMIC_T_PROGENITOR_VS_THYMIC_STROMAL_CELL_DN	194	0.4180385	19.457.738	0.0	1,79E+01	0.001
GSE16385_ROSIGLITAZONE_IFNG_TNF_VS_IL4_STIM_MACROPHAGE_UP	192	0.42041895	19.468.559	0.0	1,79E+01	0.001
GSE23502_WT_VS_HDC_KO_MYELOID_DERIVED_SUPPRESSOR_CELL_BM_UP	196	0.41983184	1.948.181	0.0	1,80E+01	0.001
GSE26030_TH1_VS_TH17_DAY5_POST_POLARIZATION_UP	193	0.42370847	19.488.112	0.0	1,80E+01	0.001
GSE32901_NAIVE_VS_TH17_ENRICHED_CD4_TCELL_UP	147	0.43534115	19.512.625	0.0	1,80E+01	0.001
GSE6092_UNSTIM_VS_IFNG_STIM_ENDOTHELIAL_CELL_DN	194	0.42194173	19.516.658	0.0	1,81E+01	0.001
GSE7852_LN_VS_FAT_TCONV_DN	197	0.42335168	19.518.193	0.0	1,81E+01	0.001
GSE20727_ROS_INH_VS_ROS_INH_AND_DNFB_ALLERGEN_TREATED_DC_UP	190	0.42416826	19.522.028	0.0	1,81E+01	0.001
GSE18281_SUBCAPSULAR_VS_CENTRAL_CORTICAL_REGION_OF_THYMUS_DN	194	0.4193761	19.525.381	0.0	1,82E+01	0.001
GSE3400_UNTREATED_VS_IFNB_TREATED_MEF_DN	164	0.43088427	19.531.037	0.0	1,82E+01	0.001
GSE15735_2H_VS_12H_HDAC_INHIBITOR_TREATED_CD4_TCELL_UP	195	0.42666712	19.531.647	0.0	1,82E+01	0.001
GSE5679_CTRL_VS_PPARG_LIGAND_ROSIGLITAZONE_AND_RARA_AGONIST_AM580_TREATED_DC_DN	197	0.3787412	1.752.788	0.0	1,83E+01	0.142
GSE9601_NFKB_INHIBITOR_VS_PI3K_INHIBITOR_TREATED_HCMV_INF_MONOCYTE_DN	194	0.42215046	19.557.399	0.0	1,83E+01	0.001
GSE9316_IL6_KO_VS_IFNG_KO_INVIVO_EXPANDED_CD4_TCELL_DN	196	0.42017084	1.955.873	0.0	1,83E+01	0.001
GSE10325_LUPUS_BCELL_VS_LUPUS_MYELOID_UP	188	0.4031823	18.435.526	0.0	1,83E+01	0.013
GSE28783_ANTI_MIR33_VS_CTRL_ATHEROSCLEROSIS_MACROPHAGE_UP	196	0.4261015	19.579.564	0.0	1,83E+01	0.001
GSE24574_BCL6_HIGH_TFH_VS_TCONV_CD4_TCELL_DN	190	0.42631465	19.591.476	0.0	1,84E+01	0.001
GSE19198_1H_VS_6H_IL21_TREATED_TCELL_UP	157	0.43962258	19.601.657	0.0	1,84E+01	0.001
GSE22886_IGG_IGA_MEMORY_BCELL_VS_BM_PLASMA_CELL_UP	197	0.4292276	19.603.268	0.0	1,85E+01	0.001
GSE3982_DC_VS_TH2_UP	191	0.4237434	196.038	0.0	1,85E+01	0.001
GSE17721_LPS_VS_POLYIC_24H_BMDM_DN	194	0.42467314	19.604.222	0.0	1,85E+01	0.001
GSE27786_LIN_NEG_VS_NKCELL_DN	190	0.42513114	19.613.541	0.0	1,86E+01	0.001
GSE22140_HEALTHY_VS_ARTHRITIC_GERMFREE_MOUSE_CD4_TCELL_DN	197	0.42166626	19.619.862	0.0	1,86E+01	0.001
GSE41867_DAY6_EFFECTOR_VS_DAY30_MEMORY_CD8_TCELL_LCMV_ARMSTRONG_UP	191	0.4269997	1.963.507	0.0	1,86E+01	0.001
GSE36476_CTRL_VS_TSST_ACT_16H_MEMORY_CD4_TCELL_OLD_UP	188	0.42720014	19.649.776	0.0	1,87E+01	0.001
GSE40666_NAIVE_VS_EFFECTOR_CD8_TCELL_WITH_IFNA_STIM_90MIN_UP	193	0.4256708	19.668.424	0.0	1,87E+01	0.001
GSE22140_GERMFREE_VS_SPF_MOUSE_CD4_TCELL_DN	198	0.42470995	1.969.581	0.0	1,88E+01	0.001
GSE36888_UNTREATED_VS_IL2_TREATED_TCELL_6H_UP	186	0.4289734	1.969.933	0.0	1,88E+01	0.001
GSE1740_UNSTIM_VS_IFNA_STIMULATED_MCSF_DERIVED_MACROPHAGE_DN	176	0.4304718	19.699.507	0.0	1,89E+01	0.001
GSE22140_HEALTHY_VS_ARTHRITIC_MOUSE_CD4_TCELL_UP	199	0.42709666	1.970.048	0.0	1,89E+01	0.001
GSE22601_DOUBLE_POSITIVE_VS_CD8_SINGLE_POSITIVE_THYMOCYTE_UP	195	0.4264542	19.744.307	0.0	1,89E+01	0.001
GSE29618_MONOCYTE_VS_MDC_DAY7_FLU_VACCINE_DN	193	0.428545	19.749.215	0.0	1,90E+01	0.001
GSE43863_LY6C_INT_CXCR5POS_VS_LY6C_LOW_CXCR5NEG_EFFECTOR_CD4_TCELL_DN	192	0.4274497	19.752.784	0.0	1,90E+01	0.001
GSE40666_UNTREATED_VS_IFNA_STIM_STAT1_KO_CD8_TCELL_90MIN_DN	187	0.42902535	19.753.462	0.0	1,91E+01	0.001
GSE17974_0H_VS_12H_IN_VITRO_ACT_CD4_TCELL_UP	177	0.43679902	19.776.126	0.0	1,91E+01	0.001
GSE42724_NAIVE_BCELL_VS_PLASMABLAST_DN	185	0.4303286	19.778.886	0.0	1,92E+01	0.001
GSE22886_NAIVE_VS_IGM_MEMORY_BCELL_DN	191	0.4239848	19.784.406	0.0	1,92E+01	0.001
GSE22886_NAIVE_VS_IGG_IGA_MEMORY_BCELL_DN	193	0.43109637	19.790.683	0.0	1,93E+01	0.001
GSE22229_UNTREATED_VS_IMMUNOSUPP_THERAPY_RENAL_TRANSPLANT_PATIENT_PBMC_DN	196	0.42346224	19.791.352	0.0	1,93E+01	0.001
GSE360_CTRL_VS_L_DONOVANI_DC_DN	194	0.42119852	19.793.603	0.0	1,94E+01	0.001
GSE8835_CD4_VS_CD8_TCELL_CLL_PATIENT_DN	187	0.42951068	19.798.174	0.0	1,94E+01	0.001
GSE37301_PRO_BCELL_VS_GRANULOCYTE_MONOCYTE_PROGENITOR_UP	138	0.44849095	19.801.126	0.0	1,94E+01	0.001
GSE17721_PAM3CSK4_VS_CPG_4H_BMDM_DN	197	0.42918032	1.980.795	0.0	1,95E+01	0.001
GSE25123_WT_VS_PPARG_KO_MACROPHAGE_IL4_STIM_DN	195	0.42601243	19.808.742	0.0	1,95E+01	0.001
GSE17974_CTRL_VS_ACT_IL4_AND_ANTI_IL12_4H_CD4_TCELL_UP	180	0.43158454	19.814.988	0.0	1,96E+01	0.001
GSE40274_CTRL_VS_FOXP3_AND_EOS_TRANSDUCED_ACTIVATED_CD4_TCELL_UP	159	0.43646246	19.822.725	0.0	1,96E+01	0.001
GSE22886_DAY1_VS_DAY7_MONOCYTE_IN_CULTURE_UP	197	0.42667702	19.824.138	0.0	1,97E+01	0.001
GSE19888_ADENOSINE_A3R_INH_VS_INH_PRETREAT_AND_ACT_WITH_TCELL_MEMBRANES_MAST_CELL_UP	191	0.42658666	19.844.786	0.0	1,97E+01	0.001
GSE24142_EARLY_THYMIC_PROGENITOR_VS_DN2_THYMOCYTE_DN	195	0.42801425	19.848.142	0.0	1,98E+01	0.001
GSE3039_CD4_TCELL_VS_B2_BCELL_UP	193	0.42749912	19.864.384	0.0	1,98E+01	0.001
GSE17974_CTRL_VS_ACT_IL4_AND_ANTI_IL12_12H_CD4_TCELL_UP	173	0.43456188	19.868.143	0.0	1,98E+01	0.001
GSE24142_EARLY_THYMIC_PROGENITOR_VS_DN3_THYMOCYTE_ADULT_UP	194	0.43168327	19.882.936	0.0	1,99E+01	0.001
GSE4535_BM_DERIVED_DC_VS_FOLLICULAR_DC_UP	190	0.43441835	19.903.942	0.0	2,00E+01	0.001
GSE22611_NOD2_TRANSDUCED_VS_CTRL_HEK293T_STIMULATED_WITH_MDP_2H_UP	172	0.4330478	19.909.571	0.0	2,00E+01	0.001
GSE40274_CTRL_VS_FOXP3_AND_XBP1_TRANSDUCED_ACTIVATED_CD4_TCELL_DN	151	0.4466829	19.914.526	0.0	2,00E+01	0.001
GSE33374_CD8_ALPHAALPHA_VS_ALPHABETA_CD161_HIGH_TCELL_DN	196	0.43414932	19.924.201	0.0	2,01E+01	0.001
GSE9988_ANTI_TREM1_VS_ANTI_TREM1_AND_LPS_MONOCYTE_DN	174	0.43958834	19.949.412	0.0	2,01E+01	0.001
GSE5542_UNTREATED_VS_IFNA_TREATED_EPITHELIAL_CELLS_6H_UP	183	0.436288	19.955.696	0.0	2,02E+01	0.001
GSE360_T_GONDII_VS_B_MALAYI_LOW_DOSE_DC_DN	197	0.43317193	19.961.293	0.0	2,02E+01	0.001
GSE16385_IFNG_TNF_VS_IL4_STIM_MACROPHAGE_UP	194	0.43271452	19.962.975	0.0	2,02E+01	0.001
GSE21927_SPLEEN_C57BL6_VS_4T1_TUMOR_BALBC_MONOCYTES_DN	194	0.43473426	19.965.426	0.0	2,03E+01	0.001
GSE2585_CD80_HIGH_VS_LOW_AIRE_KO_MTEC_UP	192	0.4301445	19.980.192	0.0	2,03E+01	0.001
GSE6259_FLT3L_INDUCED_VS_WT_SPLENIC_DC_33D1_POS_UP	160	0.44526765	19.984.959	0.0	2,04E+01	0.001
GSE360_T_GONDII_VS_M_TUBERCULOSIS_DC_DN	196	0.43391418	19.996.461	0.0	2,04E+01	0.001
GSE26290_CTRL_VS_AKT_INHIBITOR_TREATED_ANTI_CD3_AND_IL2_STIM_CD8_TCELL_DN	193	0.4330679	19.996.731	0.0	2,04E+01	0.001
GSE29618_MONOCYTE_VS_MDC_DN	194	0.43360317	19.998.255	0.0	2,05E+01	0.001
GSE21670_UNTREATED_VS_TGFB_TREATED_CD4_TCELL_DN	187	0.4342021	19.998.819	0.0	2,05E+01	0.001
GSE24634_IL4_VS_CTRL_TREATED_NAIVE_CD4_TCELL_DAY3_UP	192	0.43319455	20.003.796	0.0	2,06E+01	0.001
GSE17974_0H_VS_6H_IN_VITRO_ACT_CD4_TCELL_UP	171	0.44326702	20.018.487	0.0	2,06E+01	0.001
GSE17974_CTRL_VS_ACT_IL4_AND_ANTI_IL12_6H_CD4_TCELL_UP	177	0.43906164	20.019.774	0.0	2,07E+01	0.001
GSE19772_CTRL_VS_HCMV_INF_MONOCYTES_UP	197	0.4363654	20.031.533	0.0	2,07E+01	0.001
GSE4590_LARGE_PRE_BCELL_VS_VPREB_POS_LARGE_PRE_BCELL_DN	178	0.43552074	20.036.414	0.0	2,08E+01	0.001
GSE22886_NAIVE_BCELL_VS_BM_PLASMA_CELL_UP	196	0.43441498	2.004.741	0.0	2,08E+01	0.001
GSE33424_CD161_INT_VS_NEG_CD8_TCELL_UP	193	0.43612736	20.052.826	0.0	2,08E+01	0.001
GSE360_T_GONDII_VS_B_MALAYI_HIGH_DOSE_MAC_DN	195	0.43461108	2.006.294	0.0	2,09E+01	0.001
GSE21546_UNSTIM_VS_ANTI_CD3_STIM_SAP1A_KO_AND_ELK1_KO_DP_THYMOCYTES_UP	165	0.44095117	20.063.362	0.0	2,09E+01	0.001
GSE3982_MAST_CELL_VS_EFF_MEMORY_CD4_TCELL_DN	188	0.4326962	20.066.915	0.0	2,10E+01	0.001

GSE3982_BCELL_VS_NKCELL_DN	193	0.43407097	20.067.844	0.0	2,10E+01	0.001
GSE31082_DN_VS_CD8_SP_THYMOCYTE_DN	193	0.43733934	20.068.285	0.0	2,11E+01	0.001
GSE14000_UNSTIM_VS_16H_LPS_DC_TRANSLATED_RNA_DN	186	0.44277528	200.713	0.0	2,11E+01	0.001
GSE36392_EOSINOPHIL_VS_NEUTROPHIL_IL25_TREATED_LUNG_UP	198	0.42839748	2.007.389	0.0	2,12E+01	0.001
GSE21360_TERTIARY_VS_QUATERNARY_MEMORY_CD8_TCELL_UP	179	0.43793666	20.074.646	0.0	2,12E+01	0.001
GSE21379_TFH_VS_NON_TFH_CD4_TCELL_DN	190	0.37914854	17.381.697	0.0	2,36E+01	0.188
GSE21546_UNSTIM_VS_ANTI_CD3_STIM_DP_THYMOCYTES_UP	183	0.39730918	18.370.496	0.0	2,75E+01	0.02
GSE30971_WBP7_HET_VS_KO_MACROPHAGE_DN	187	0.37525806	17.242.371	0.0	3,10E+01	0.244
GSE24574_BCL6_HIGH_VS_LOW_TFH_CD4_TCELL_UP	195	0.41224134	18.918.245	0.0	3,14E+01	0.002
GSE14415_INDUCED_TREG_VS_TCONV_DN	150	0.42103663	18.920.145	0.0	3,14E+01	0.002
GSE40443_INDUCED_VS_TOTAL_TREG_DN	191	0.41037923	18.934.597	0.0	3,15E+01	0.002
GSE7768_OVA_ALONE_VS_OVA_WITH_LPS_IMMUNIZED_MOUSE_WHOLE_SPLEEN_6H_DN	164	0.4173323	18.936.988	0.0	3,16E+01	0.002
GSE21379_WT_VS_SAP_KO_TFH_CD4_TCELL_UP	193	0.41167	1.893.736	0.0	3,16E+01	0.002
GSE29618_BCELL_VS_PDC_DAY7_FLU_VACCINE_DN	193	0.40382645	18.954.093	0.0	3,17E+01	0.002
GSE46242_CTRL_VS_EGR2_DELETED_ANERGIC_TH1_CD4_TCELL_DN	186	0.40808058	18.967.127	0.0	3,19E+01	0.002
GSE33513_TCF7_KO_VS_HET_EARLY_THYMIC_PROGENITOR_UP	189	0.41174778	18.980.777	0.0	3,20E+01	0.002
GSE7348_UNSTIM_VS_LPS_STIM_MACROPHAGE_DN	165	0.42032197	18.989.569	0.0	3,20E+01	0.002
GSE42088_UNINF_VS_LEISHMANIA_INF_DC_24H_UP	196	0.41381747	1.899.646	0.0	3,21E+01	0.002
GSE39556_UNTREATED_VS_3H_POLYIC_INJ_MOUSE_NK_CELL_DN	195	0.41307387	18.997.946	0.0	3,21E+01	0.002
GSE33292_DN3_THYMOCYTE_VS_TCF1_KO_TCELL_LYMPHOMA_UP	197	0.41036072	19.005.971	0.0	3,22E+01	0.002
GSE16385_ROSIGLITAZONE_IL4_VS_IFNG_TNF_STIM_MACROPHAGE_UP	193	0.40915248	19.006.917	0.0	3,23E+01	0.002
GSE15735_CTRL_VS_HDAC_INHIBITOR_TREATED_CD4_TCELL_2H_UP	195	0.41569978	19.041.607	0.0	3,25E+01	0.002
GSE26912_TUMORICIDAL_VS_CTRL_MACROPHAGE_DN	194	0.4154815	1.904.889	0.0	3,25E+01	0.002
GSE22886_NAIVE_TCELL_VS_DC_DN	199	0.40745407	19.049.037	0.0	3,26E+01	0.002
GSE2770_IL12_AND_TGFB_VS_IL4_TREATED_ACT_CD4_TCELL_6H_DN	189	0.41429424	19.058.683	0.0	3,26E+01	0.002
GSE24671_CTRL_VS_BAKIMULC_INFECTED_MOUSE_SPLENOCYTES_DN	166	0.42131513	190.727	0.0	3,27E+01	0.002
GSE24634_IL4_VS_CTRL_TREATED_NAIVE_CD4_TCELL_DAY10_DN	197	0.4163309	19.079.162	0.0	3,28E+01	0.002
GSE9509_10MIN_VS_30MIN_LPS_STIM_IL10_KO_MACROPHAGE_DN	181	0.4186314	19.093.637	0.0	3,28E+01	0.002
GSE17974_0H_VS_2H_IN_VITRO_ACT_CD4_TCELL_UP	178	0.41686195	1.910.611	0.0	3,29E+01	0.002
GSE19198_6H_VS_24H_IL21_TREATED_TCELL_DN	191	0.41638184	19.117.069	0.0	3,30E+01	0.002
GSE7568_CTRL_VS_24H_TGFB_TREATED_MACROPHAGES_WITH_IL4_AND_DEXAMETHASONE_UP	190	0.41501027	19.119.345	0.0	3,30E+01	0.002
GSE13484_12H_UNSTIM_VS_YF17D_VACCINE_STIM_PBMC_DN	196	0.41275665	19.135.605	0.0	3,33E+01	0.002
GSE36476_CTRL_VS_TSST_ACT_72H_MEMORY_CD4_TCELL_YOUNG_UP	192	0.41495305	19.137.416	0.0	3,33E+01	0.002
GSE2585_CTEC_VS_MTEC_THYMUS_DN	192	0.40825024	19.138.612	0.0	3,34E+01	0.002
GSE15930_STIM_VS_STIM_AND_IL-12_48H_CD8_T_CELL_UP	197	0.41060805	19.142.357	0.0	3,34E+01	0.002
GSE37534_UNTREATED_VS_ROSIGLITAZONE_TREATED_CD4_TCELL_PPARG1_AND_FOXP3_TRASDUCED_DN	191	0.4154509	19.144.826	0.0	3,35E+01	0.002
GSE1112_HY_CD8AB_VS_HY_CD8AA_THYMOCYTE_RTOC_CULTURE_UP	177	0.4236087	19.177.016	0.0	3,36E+01	0.002
GSE32901_NAIVE_VS_TH17_NEG_CD4_TCELL_UP	167	0.40367022	18.283.478	0.0	3,36E+01	0.025
GSE3982_EOSINOPHIL_VS_MAST_CELL_UP	193	0.41391775	19.178.245	0.0	3,37E+01	0.002
GSE37301_COMMON_LYMPHOID_PROGENITOR_VS_RAG2_KO_NK_CELL_DN	190	0.39759445	18.250.988	0.0	3,60E+01	0.027
GSE28737_BCL6_HET_VS_BCL6_KO_MARGINAL_ZONE_BCELL_UP	194	0.39701778	18.255.174	0.0	3,61E+01	0.027
GSE18804_SPLEEN_MACROPHAGE_VS_COLON_TUMORAL_MACROPHAGE_UP	198	0.39719886	1.827.351	0.0	3,62E+01	0.027
GSE3982_DC_VS_EFF_MEMORY_CD4_TCELL_DN	189	0.39820513	18.212.359	0.0	3,70E+01	0.028
GSE9988_ANTI_TREM1_AND_LPS_VS_CTRL_TREATED_MONOCYTES_DN	189	0.38838002	1.821.534	0.0	3,71E+01	0.028
GSE40274_CTRL_VS_EOS_TRANSDUCED_ACTIVATED_CD4_TCELL_DN	142	0.4110758	18.194.631	0.0	3,95E+01	0.03
GSE30962_ACUTE_VS_CHRONIC_LCMV_SECONDARY_INF_CD8_TCELL_UP	196	0.39121118	18.159.212	0.0	4,07E+01	0.031
GSE17974_IL4_AND_ANTI_IL12_VS_UNTREATED_48H_ACT_CD4_TCELL_UP	173	0.39925566	1.817.881	0.0	4,08E+01	0.031
GSE3039_NKT_CELL_VS_B1_BCELL_UP	187	0.38758373	18.127.377	0.0	4,31E+01	0.033
GSE360_L_DONOVANI_VS_B_MALAYI_HIGH_DOSE_DC_UP	192	0.41242	18.891.457	0.0	4,70E+01	0.003
GSE46606_IRF4_KO_VS_WT_UNSTIM_BCELL_DN	193	0.4096584	18.902.235	0.0	4,73E+01	0.003
GSE13738_TCR_VS_BYSTANDER_ACTIVATED_CD4_TCELL_DN	184	0.3725237	16.984.469	0.0013404826	4,78E+01	0.364
GSE5503_MLN_DC_VS_SPLEEN_DC_ACTIVATED_ALLOGENIC_TCELL_UP	194	0.3931476	18.099.841	0.0	4,82E+01	0.036
GSE12003_MIR223_KO_VS_WT_BM_PROGENITOR_8D_CULTURE_UP	119	0.41251704	18.092.242	0.0	5,07E+01	0.038
GSE37605_TREG_VS_TCONV_C57BL6_FOXP3_FUSION_GFP_UP	127	0.4076433	18.083.084	0.0	5,17E+01	0.039
GSE9988_LOW_LPS_VS_VEHICLE_TREATED_MONOCYTE_UP	177	0.37140265	16.935.322	0.0	5,23E+01	0.4
GSE28737_WT_VS_BCL6_HET_FOLLICULAR_BCELL_DN	188	0.38906762	18.039.572	0.0	5,41E+01	0.04
GSE360_DC_VS_MAC_T_GONDII_DN	196	0.3876068	1.799.871	0.0	5,73E+01	0.042
GSE18281_SUBCAPSULAR_VS_PERIMEDULLARY_CORTICAL_REGION_OF_THYMUS_UP	195	0.391984	18.002.845	0.0	5,74E+01	0.042
GSE40273_XBP1_KO_VS_WT_TREG_DN	190	0.38913897	17.986.329	0.0	5,83E+01	0.043
GSE22886_NAIVE_BCELL_VS_MONOCYTE_UP	187	0.40698507	18.783.131	0.0	6,10E+01	0.004
GSE11961_GERMINAL_CENTER_BCELL_DAY7_VS_MEMORY_BCELL_DAY40_DN	195	0.40914166	18.790.833	0.0	6,12E+01	0.004
GSE15271_CXCR4_POS_VS_NEG_GC_BCELL_DN	165	0.41151655	18.790.942	0.0	6,13E+01	0.004
GSE40274_CTRL_VS_SATB1_TRANSDUCED_ACTIVATED_CD4_TCELL_UP	163	0.4141588	18.811.395	0.0	6,15E+01	0.004
GSE21774_CD62L_POS_CD56_DIM_VS_CD62L_NEG_CD56_DIM_NK_CELL_DN	194	0.41040397	18.818.407	0.0	6,16E+01	0.004
GSE2770_IL12_VS_TGFB_AND_IL12_TREATED_ACT_CD4_TCELL_48H_UP	194	0.40855718	18.853.736	0.0	6,20E+01	0.004
GSE40274_CTRL_VS_HELIOS_TRANSDUCED_ACTIVATED_CD4_TCELL_UP	155	0.424041	188.633	0.0	6,21E+01	0.004
GSE40274_CTRL_VS_FOXP3_AND_PBX1_TRANSDUCED_ACTIVATED_CD4_TCELL_UP	154	0.41971213	18.866.339	0.0	6,22E+01	0.004
GSE7831_UNSTIM_VS_INFLUENZA_STIM_PDC_4H_UP	196	0.41144916	18.867.564	0.0	6,23E+01	0.004
GSE32255_UNSTIM_VS_4H_LPS_STIM_DC_UP	175	0.41546306	18.879.768	0.0	6,24E+01	0.004
GSE16451_CTRL_VS_WEST_EQUINE_ENC_VIRUS_MATURE_NEURON_CELL_LINE_DN	195	0.40547007	18.882.251	0.0	6,25E+01	0.004
GSE2706_UNSTIM_VS_8H_LPS_DC_DN	190	0.40922752	1.888.957	0.0	6,26E+01	0.004
GSE32986_CURDLAN_LOWDOSE_VS_CURDLAN_HIGHDOSE_STIM_DC_UP	190	0.3871596	17.944.547	0.0	6,39E+01	0.048
GSE3720_LPS_VS_PMA_STIM_VD2_GAMMADELTA_TCELL_UP	131	0.40816855	17.942.477	0.0	6,51E+01	0.049
GSE11961_UNSTIM_VS_ANTI_IGM_AND_CD40_STIM_6H_FOLLICULAR_BCELL_DN	191	0.38868776	17.933.682	0.0	6,59E+01	0.05
GSE17580_TREG_VS_TEFF_S_MANSONI_INF_DN	191	0.3878832	17.935.368	0.0	6,60E+01	0.05
GSE17721_PAM3CSK4_VS_GADIQUIMOD_12H_BMDM_DN	197	0.3882853	17.935.563	0.0	6,61E+01	0.05
GSE1448_CTRL_VS_ANTI_VBETA5_DP_THYMOCYTE_DN	197	0.38957995	17.923.374	0.0	6,82E+01	0.052
GSE13306_TREG_VS_TCONV_SPLEEN_DN	191	0.3861509	17.911.904	0.0	6,92E+01	0.053
GSE4590_SMALL_VS_LARGE_PRE_BCELL_DN	153	0.39499208	17.892.321	0.0	7,00E+01	0.054
GSE16266_CTRL_VS_LPS_STIM_MEF_DN	194	0.38232198	17.892.641	0.0	7,01E+01	0.054
GSE37605_C57BL6_VS_NOD_FOXP3_IRES_GFP_TREG_DN	194	0.38903505	17.893.564	0.0	7,02E+01	0.054
GSE360_L_DONOVANI_VS_B_MALAYI_LOW_DOSE_DC_DN	199	0.385123	17.895.446	0.0	7,03E+01	0.054
GSE20366_TREG_VS_NAIVE_CD4_TCELL_DEC205_CONVERSION_UP	195	0.39123937	17.896.918	0.0	7,03E+01	0.054
GSE17974_IL4_AND_ANTI_IL12_VS_UNTREATED_24H_ACT_CD4_TCELL_UP	161	0.3709907	16.746.324	0.0	7,11E+01	0.515
GSE24142_DN2_VS_DN3_THYMOCYTE_UP	198	0.3830064	17.880.954	0.0	7,23E+01	0.055
GSE22935_UNSTIM_VS_24H_MBOVIS_BCG_STIM_MYD88_KO_MACROPHAGE_DN	188	0.3636487	16.739.278	0.0	7,25E+01	0.524
GSE17974_IL4_AND_ANTI_IL12_VS_UNTREATED_2H_ACT_CD4_TCELL_UP	173	0.40646192	1.865.627	0.0	7,41E+01	0.005
GSE360_CTRL_VS_T_GONDII_DC_DN	192	0.40473494	18.665.292	0.0	7,45E+01	0.005
GSE17974_0H_VS_72H_IN_VITRO_ACT_CD4_TCELL_UP	179	0.40452242	18.687.172	0.0	7,48E+01	0.005

GSE36476_CTRL_VS_TSST_ACT_40H_MEMORY_CD4_TCELL_YOUNG_UP	187	0.40660182	18.706.768	0.0	7,50E+01	0.005
GSE1740_UNSTIM_VS_IFNA_STIMULATED_MCSF_IFNG_DERIVED_MACROPHAGE_UP	171	0.40624344	18.740.413	0.0	7,55E+01	0.005
GSE2128_CTRL_VS_MIMETOPE_NEGATIVE_SELECTION_DP_THYMOCYTE_NOD_DN	177	0.41494626	18.755.798	0.0	7,58E+01	0.005
GSE21670_UNTREATED_VS_IL6_TREATED_CD4_TCELL_DN	196	0.40809125	18.773.755	0.0	7,60E+01	0.005
GSE29949_CD8_POS_DC_SPLEEN_VS_MONOCYTE_BONE_MARROW_UP	194	0.40703437	18.774.496	0.0	7,61E+01	0.005
GSE21380_NON_TFH_VS_TFH_CD4_TCELL_DN	196	0.389787	17.866.893	0.0	7,70E+01	0.058
GSE32986_UNSTIM_VS_GMCSF_STIM_DC_UP	184	0.39069617	1.786.816	0.0	7,71E+01	0.058
GSE5099_DAY3_VS_DAY7_MCSF_TREATED_MACROPHAGE_UP	183	0.38934878	17.868.837	0.0	7,72E+01	0.058
GSE3982_DC_VS_NKCELL_DN	188	0.38984242	17.849.835	0.0	8,53E+01	0.065
GSE37416_CTRL_VS_24H_F_TULARENSIS_LVS_NEUTROPHIL_UP	184	0.38616955	17.834.114	0.0	8,77E+01	0.067
GSE11057_EFF_MEM_VS_CENT_MEM_CD4_TCELL_UP	187	0.38499406	17.796.955	0.0	9,29E+01	0.072
GSE15624_3H_VS_6H_HALOFUGINONE_TREATED_CD4_TCELL_UP	162	0.39453462	17.797.183	0.0	9,30E+01	0.072
GSE12845_IGD_NEG_BLOOD_VS_DARKZONE_GC_TONSIL_BCELL_UP	189	0.38368878	17.805.605	0.0	9,31E+01	0.072
GSE22886_DAY0_VS_DAY1_MONOCYTE_IN_CULTURE_DN	194	0.36316678	16.590.568	0.0	9,41E+01	0.627
GSE37301_COMMON_LYMPHOID_PROGENITOR_VS_GRAN_MONO_PROGENITOR_DN	186	0.38797235	17.774.493	0.0	9,73E+01	0.076
GSE22886_NEUTROPHIL_VS_DC_UP	190	0.3628514	16.554.921	0.0	9,90E+01	0.648
GSE12845_IGD_POS_VS_NEG_BLOOD_BCELL_UP	189	0.38459408	17.752.513	0.0	1,09E+02	0.084
GSE24142_EARLY_THYMIC_PROGENITOR_VS_DN3_THYMOCYTE_DN	193	0.40331826	18.578.631	0.0	1,32E+02	0.009
GSE3982_EOSINOPHIL_VS_CENT_MEMORY_CD4_TCELL_UP	191	0.38658434	17.680.483	0.0	1,33E+02	0.104
GSE24292_WT_VS_PPARG_KO_MACROPHAGE_DN	194	0.38058335	17.639.289	0.0	1,50E+02	0.115
GSE22140_GERMFREE_VS_SPF_ARTHRITIC_MOUSE_CD4_TCELL_UP	195	0.38018793	17.618.979	0.0	1,53E+02	0.118
GSE12392_WT_VS_IFNB_KO_CD8A_POS_SPLEEN_DC_DN	193	0.38524133	17.619.381	0.0	1,53E+02	0.118
GSE7764_IL15_TREATED_VS_CTRL_NK_CELL_24H_DN	190	0.39976987	1.847.968	0.0	1,57E+02	0.011
GSE20715_WT_VS_TLR4_KO_48H_OZONE_LUNG_UP	198	0.40027538	18.480.451	0.0	1,58E+02	0.011
GSE3982_MAST_CELL_VS_BASOPHIL_DN	188	0.4007428	18.483.107	0.0	1,58E+02	0.011
GSE7460_FOXP3_MUT_VS_WT_ACT_TCONV_UP	191	0.4049793	18.485.389	0.0	1,58E+02	0.011
GSE7460_CTRL_VS_FOXP3_OVEREXPR_TCONV_1_DN	191	0.40238923	18.491.977	0.0	1,58E+02	0.011
GSE27241_CTRL_VS_DIGOXIN_TREATED_CD4_TCELL_IN_TH17_POLARIZING_CONDITIONS_UP	156	0.40801817	18.504.993	0.0	1,59E+02	0.011
GSE3039_ALPHAALPHA_CD8_TCELL_VS_B2_BCELL_UP	194	0.4000333	18.518.405	0.0	1,59E+02	0.011
GSE22589_HEALTHY_VS_SIV_INFECTED_DC_DN	193	0.401356	18.518.703	0.0	1,59E+02	0.011
GSE14769_UNSTIM_VS_60MIN_LPS_BMDM_DN	189	0.39931214	18.520.596	0.0	1,59E+02	0.011
GSE29618_PDC_VS_MDC_DAY7_FLU_VACCINE_UP	192	0.4058667	1.852.615	0.0	1,59E+02	0.011
GSE45365_CD8A_DC_VS_CD11B_DC_IFNAR_KO_MCMV_INFECTION_DN	158	0.41044626	18.532.572	0.0	1,60E+02	0.011
GSE6090_UNSTIM_VS_DC-SIGN_STIM_DC_DN	140	0.42218095	18.533.862	0.0	1,60E+02	0.011
GSE16385_ROSIGLITAZONE_IL4_VS_IFNG_TNF_STIM_MACROPHAGE_DN	189	0.40343094	18.536.248	0.0	1,60E+02	0.011
GSE3337_CTRL_VS_4H_IFNG_IN_CD8POS_DC_DN	197	0.40018466	185.392	0.0	1,60E+02	0.011
GSE12366_NAIVE_VS_MEMORY_BCELL_UP	174	0.4062176	18.540.922	0.0	1,61E+02	0.011
GSE21360_SECONDARY_VS_TERTIARY_MEMORY_CD8_TCELL_DN	157	0.41273162	18.552.028	0.0	1,61E+02	0.011
GSE24142_ADULT_VS_FETAL_DN2_THYMOCYTE_UP	195	0.40051857	18.562.088	0.0	1,62E+02	0.011
GSE22025_TGFB1_VS_TGFB1_AND_PROGESTERONE_TREATED_CD4_TCELL_UP	193	0.40164405	18.563.709	0.0	1,62E+02	0.011
GSE2770_IL4_ACT_VS_ACT_CD4_TCELL_48H_DN	192	0.37929553	17.587.489	0.0	1,62E+02	0.125
GSE8621_UNSTIM_VS_LPS_PRIMED_AND_LPS_STIM_MACROPHAGE_DN	190	0.40205935	18.461.432	0.0	1,71E+02	0.012
GSE10239_NAIVE_VS_MEMORY_CD8_TCELL_DN	190	0.40243614	18.464.946	0.0	1,71E+02	0.012
GSE19198_CTRL_VS_IL21_TREATED_TCELL_24H_DN	193	0.4032116	18.473.572	0.0	1,71E+02	0.012
GSE34006_WT_VS_A2AR_KO_TREG_DN	194	0.40027386	18.475.587	0.0	1,71E+02	0.012
GSE35543_IN_VIVO_NTREG_VS_IN_VITRO_ITREG_DN	161	0.38962898	17.565.705	0.0	1,72E+02	0.131
GSE36888_UNTREATED_VS_IL2_TREATED_STAT5_AB_KNOCKIN_TCELL_17H_UP	178	0.3965576	18.422.282	0.0	1,82E+02	0.013
GSE25085_FETAL_LIVER_VS_FETAL_BM_SP4_THYMIC_IMPLANT_DN	190	0.40377626	1.842.794	0.0	1,83E+02	0.013
GSE3982_MEMORY_CD4_TCELL_VS_TH2_UP	183	0.40525806	18.429.345	0.0	1,83E+02	0.013
GSE13522_CTRL_VS_T_CRUZI_Y_STRAIN_INF_SKIN_BALBC_MOUSE_DN	126	0.42453095	18.437.766	0.0	1,84E+02	0.013
GSE9988_ANTI_TREM1_VS_LPS_MONOCYTE_UP	188	0.40119192	18.437.893	0.0	1,84E+02	0.013
GSE2706_R848_VS_R848_AND_LPS_8H_STIM_DC_UP	171	0.4025076	18.443.271	0.0	1,84E+02	0.013
GSE5455_HEALTHY_VS_TUMOR_BEARING_MOUSE_SPLEEN_MONOCYTE_24H_INCUBATION_DN	192	0.4026593	18.451.711	0.0	1,84E+02	0.013
GSE360_HIGH_DOSE_B_MALAYI_VS_M_TUBERCULOSIS_MAC_DN	193	0.4014485	18.453.965	0.0	1,85E+02	0.013
GSE10856_CTRL_VS_TNFRSF6B_IN_MACROPHAGE_UP	178	0.38092718	17.496.129	0.0	1,90E+02	0.15
GSE39110_UNTREATED_VS_IL2_TREATED_CD8_TCELL_DAY6_POST_IMMUNIZATION_DN	197	0.37927148	17.490.473	0.0	1,93E+02	0.153
GSE34006_A2AR_KO_VS_A2AR_AGONIST_TREATED_TREG_UP	196	0.4024432	18.407.835	0.0	1,94E+02	0.014
GSE5589_IL6_KO_VS_IL10_KO_LPS_AND_IL10_STIM_MACROPHAGE_45MIN_UP	193	0.39814484	18.408.304	0.0	1,95E+02	0.014
GSE28726_ACT_CD4_TCELL_VS_ACT_VA24NEG_NKTCELL_DN	195	0.39860156	18.409.193	0.0	1,95E+02	0.014
GSE16522_MEMORY_VS_NAIVE_ANTI_CD3CD28_STIM_CD8_TCELL_DN	197	0.39899597	18.410.258	0.0	1,95E+02	0.014
GSE33425_CD161_INT_VS_NEG_CD8_TCELL_DN	196	0.398903	18.410.654	0.0	1,96E+02	0.014
GSE360_T_GONDII_VS_B_MALAYI_HIGH_DOSE_DC_DN	196	0.39940992	18.418.264	0.0	1,96E+02	0.014
GSE4748_CTRL_VS_CYANOBACTERIUM_LPSLIKE_STIM_DC_3H_UP	192	0.3956057	18.419.403	0.0	1,96E+02	0.014
GSE26343_WT_VS_NFAT5_KO_MACROPHAGE_DN	199	0.37638524	17.469.581	0.0	2,02E+02	0.159
GSE23502_BM_VS_COLON_TUMOR_HDC_KO_MYELOID_DERIVED_SUPPRESSOR_CELL_UP	193	0.37953714	17.415.439	0.0	2,16E+02	0.169
GSE10147_IL3_VS_IL3_AND_CPG_STIM_PDC_UP	137	0.3989976	17.403.111	0.0	2,28E+02	0.18
GSE29164_DAY3_VS_DAY7_UNTREATED_MELANOMA_UP	180	0.37744004	17.388.421	0.0	2,36E+02	0.188
GSE19923_WT_VS_HEB_KO_DP_THYMOCYTE_UP	196	0.37358236	17.363.695	0.0	2,47E+02	0.194
GSE3982_BCELL_VS_EFF_MEMORY_CD4_TCELL_DN	190	0.39851007	1.838.513	0.0	2,49E+02	0.018
GSE43863_DAY6_EFF_VS_DAY150_MEM_TFH_CD4_TCELL_DN	194	0.39978608	1.838.585	0.0	2,49E+02	0.018
GSE2706_UNSTIM_VS_8H_LPS_AND_R848_DC_DN	186	0.377867	17.353.985	0.0	2,49E+02	0.196
GSE43863_NAIVE_VS_TFH_CD4_EFF_TCELL_D6_LCMV_DN	156	0.40959492	18.388.261	0.0	2,49E+02	0.018
GSE36476_YOUNG_VS_OLD_DONOR_MEMORY_CD4_TCELL_72H_TSST_ACT_DN	187	0.37686792	1.734.995	0.0	2,51E+02	0.198
GSE32034_LY6C_HIGH_VS_LOW_MONOCYTE_UP	195	0.37685287	17.346.034	0.0	2,52E+02	0.199
GSE7568_IL4_TGFB_DEXAMETHASONE_VS_IL4_TGFB_TREATED_MACROPHAGE_DN	172	0.38006386	17.337.315	0.0	2,55E+02	0.201
GSE3982_EOSINOPHIL_VS_DC_UP	193	0.37563026	17.318.959	0.0	2,63E+02	0.209
GSE3982_EFF_MEMORY_VS_CENT_MEMORY_CD4_TCELL_UP	190	0.3744451	1.731.749	0.0	2,65E+02	0.209
GSE32423_IL7_VS_IL7_IL4_NAIVE_CD8_TCELL_UP	192	0.39983448	18.367.277	0.0	2,74E+02	0.02
GSE27241_CTRL_VS_DIGOXIN_TREATED_RORGT_KO_CD4_TCELL_IN_TH17_POLARIZING_CONDITIONS_DN	172	0.40570986	1.836.911	0.0	2,74E+02	0.02
GSE36392_TYPE_2_MYELOID_VS_NEUTROPHIL_IL25_TREATED_LUNG_UP	195	0.4002888	18.369.595	0.0	2,75E+02	0.02
GSE11961_FOLLICULAR_BCELL_VS_MARGINAL_ZONE_BCELL_UP	192	0.39691123	18.373.008	0.0013003901	2,75E+02	0.02
GSE22282_HYPOXIA_VS_NORMOXIA_MYELOID_DC_DN	185	0.3984	18.373.449	0.0	2,76E+02	0.02
GSE15330_HSC_VS_MEGAKARYOCYTE_ERYTHROID_PROGENITOR_UP	159	0.38138995	17.297.447	0.0026595744	2,76E+02	0.217
GSE40273_GATA1_KO_VS_WT_TREG_DN	194	0.39984292	18.380.053	0.0	2,76E+02	0.02
GSE22601_IMMATURE_CD4_SINGLE_POSITIVE_VS_DOUBLE_POSITIVE_THYMOCYTE_UP	170	0.38482794	17.286.311	0.0	2,81E+02	0.219
GSE45365_HEALTHY_VS_MCMV_INFECTION_CD8A_DC_DN	196	0.39657375	1.831.988	0.0	2,83E+02	0.021
GSE4984_UNTREATED_VS_GALECTIN1_TREATED_DC_UP	180	0.39927304	18.320.237	0.0	2,84E+02	0.021
GSE10422_WT_VS_BAFF_TRANSGENIC_LN_BCELL_DN	124	0.42059642	18.322.365	0.0	2,84E+02	0.021
GSE3982_BASOPHIL_VS_TH2_UP	189	0.39884087	18.323.574	0.0	2,84E+02	0.021

GSE36527_CD62L_HIGH_VS_CD62L_LOW_TREG_CD69_NEG_KLRG1_NEG_UP	196	0.39037532	1.833.184	0.0	2,85E+02	0.021
GSE15735_CTRL_VS_HDAC_INHIBITOR_TREATED_CD4_TCELL_12H_DN	193	0.39809832	18.333.462	0.0	2,85E+02	0.021
GSE35685_CD34POS_CD10NEG_CD62LPOS_VS_CD34POS_CD10POS_BONE_MARROW_DN	198	0.39660022	18.343.211	0.0	2,86E+02	0.021
GSE30083_SP2_VS_SP3_THYMOCYTE_UP	199	0.39646816	18.349.006	0.0	2,86E+02	0.021
GSE5455_HEALTHY_VS_TUMOR_BEARING_MOUSE_SPLEEN_MONOCYTE_UP	183	0.3997832	18.350.171	0.0	2,86E+02	0.021
GSE25088_WT_VS_STAT6_KO_MACROPHAGE_ROSIGLITAZONE_AND_IL4_STIM_DN	182	0.40356734	18.350.387	0.0	2,87E+02	0.021
GSE2706_R848_VS_LPS_8H_STIM_DC_DN	181	0.3987216	18.353.754	0.0	2,87E+02	0.021
GSE46606_DAY1_VS_DAY3_CD40L_IL2_IL5_STIMULATED_IRF4HIGH_BCELL_DN	191	0.3749251	17.263.432	0.0	2,94E+02	0.23
GSE40666_UNTREATED_VS_IFNA_STIM_EFFECTOR_CD8_TCELL_90MIN_UP	186	0.37802076	17.248.895	0.0	3,04E+02	0.238
GSE43955_1H_VS_10H_ACT_CD4_TCELL_WITH_TGFB_IL6_DN	194	0.39385408	18.313.569	0.0	3,10E+02	0.023
GSE46606_IRF4MID_VS_WT_CD40L_IL2_IL5_DAY1_STIMULATED_BCELL_DN	189	0.3726539	17.235.444	0.0	3,17E+02	0.251
GSE29949_MICROGLIA_BRAIN_VS_CD8_POS_DC_SPLEEN_UP	196	0.37302157	17.229.042	0.0013351135	3,21E+02	0.256
GSE19401_PAM2CSK4_VS_RETINOIC_ACID_AND_PAM2CSK4_STIM_FOLLICULAR_DC_DN	196	0.37014383	17.223.041	0.0	3,24E+02	0.258
GSE32255_UNSTIM_VS_4H_LPS_STIM_DC_DN	148	0.38395572	17.208.481	0.0013333333	3,34E+02	0.264
GSE10239_NAIVE_VS_KLRG1HIGH_EFF_CD8_TCELL_UP	193	0.37151504	17.195.024	0.0	3,37E+02	0.267
GSE3982_NEUTROPHIL_VS_TH2_UP	198	0.36623248	17.183.813	0.0	3,44E+02	0.271
GSE22886_IGM_MEMORY_BCELL_VS_BM_PLASMA_CELL_UP	190	0.39735243	18.275.188	0.0	3,49E+02	0.026
GSE26669_CTRL_VS_COSTIM_BLOCK_MLR_CD8_TCELL_DN	195	0.3712413	1.717.059	0.0	3,52E+02	0.277
GSE14415_ACT_TCONV_VS_ACT_NATURAL_TREG_DN	174	0.37078044	17.157.172	0.0	3,55E+02	0.282
GSE37301_HEMATOPOIETIC_STEM_CELL_VS_GRAN_MONO_PROGENITOR_DN	173	0.37577775	17.157.389	0.0	3,56E+02	0.282
GSE44732_UNSTIM_VS_IL27_STIM_IMATURE_DC_DN	188	0.3725212	17.152.276	0.0	3,58E+02	0.283
GSE43863_DAY6_EFF_VS_DAY150_MEM_LY6C_INT_CXCR5POS_CD4_TCELL_UP	193	0.39598468	18.230.399	0.0	3,58E+02	0.027
GSE3982_MAST_CELL_VS_NEUTROPHIL_DN	195	0.39224082	1.823.843	0.0	3,59E+02	0.027
GSE34205_HEALTHY_VS_RSV_INF_INFANT_PBMC_UP	182	0.3925662	1.824.826	0.0	3,59E+02	0.027
GSE37301_CD4_TCELL_VS_GRANULOCYTE_MONOCYTE_PROGENITOR_UP	160	0.406411	18.251.033	0.0	3,60E+02	0.027
GSE36078_WT_VS_IL1R_KO_LUNG_DC_DN	191	0.3969326	18.261.787	0.0	3,62E+02	0.027
GSE33425_CD161_HIGH_VS_INT_CD8_TCELL_UP	195	0.3979447	18.224.698	0.0	3,71E+02	0.028
GSE25085_FETAL_BM_VS_ADULT_BM_SP4_THYMIC_IMPLANT_UP	193	0.37026322	17.125.714	0.0	3,79E+02	0.296
GSE15330_WT_VS_IKAROS_KO_GRANULOCYTE_MONOCYTE_PROGENITOR_DN	192	0.39322877	18.206.401	0.0	3,96E+02	0.03
GSE26727_WT_VS_KLF2_KO_LPS_STIM_MACROPHAGE_UP	196	0.3670949	17.097.378	0.0	3,96E+02	0.305
GSE40274_FOXP3_VS_FOXP3_AND_IRF4_TRANSDUCED_ACTIVATED_CD4_TCELL_UP	195	0.3953724	18.153.955	0.0	4,05E+02	0.031
GSE30971_WBP7_HET_VS_KO_MACROPHAGE_2H_LPS_STIM_DN	188	0.3945163	18.155.166	0.0	4,06E+02	0.031
GSE21546_UNSTIM_VS_ANTI_CD3_STIM_SAP1A_KO_DP_THYMOCYTES_UP	183	0.39574814	18.156.042	0.0	4,06E+02	0.031
GSE37416_12H_VS_48H_F_TULARENSIS_LVS_NEUTROPHIL_UP	191	0.39425868	18.179.432	0.0	4,08E+02	0.031
GSE39820_CTRL_VS_IL1B_IL6_CD4_TCELL_DN	190	0.3694792	17.038.395	0.0	4,38E+02	0.337
GSE8921_UNSTIM_0H_VS_TLR1_2_STIM_MONOCYTE_6H_DN	193	0.3692605	17.030.466	0.0	4,42E+02	0.34
GSE36888_UNTREATED_VS_IL2_TREATED_STAT5_AB_KNOCKIN_TCELL_2H_DN	191	0.37114626	17.030.566	0.0	4,42E+02	0.34
GSE43863_TH1_VS_TFH_EFFECTOR_CD4_TCELL_DN	192	0.36524624	17.003.508	0.0	4,65E+02	0.356
GSE26343_WT_VS_NFAT5_KO_MACROPHAGE_LPS_STIM_DN	191	0.37070057	16.987.334	0.0	4,75E+02	0.363
GSE41176_UNSTIM_VS_ANTI_IGM_STIM_TAK1_KO_BCELL_24H_DN	194	0.3889973	18.099.629	0.0	4,82E+02	0.036
GSE43863_NAIVE_VS_TH1_EFF_CD4_TCELL_D6_LCMV_DN	157	0.37835312	16.954.633	0.0013422819	5,00E+02	0.382
GSE24726_WT_VS_E2-2_KO_PDC_DAY6_POST_DELETION_DN	197	0.36723983	1.694.978	0.0	5,04E+02	0.384
GSE3982_MAST_CELL_VS_CENT_MEMORY_CD4_TCELL_DN	187	0.3919017	18.084.694	0.0	5,06E+02	0.038
GSE37605_TREG_VS_TCONV_NOD_FOXP3_FUSION_GFP_UP	141	0.40751398	18.090.929	0.0	5,06E+02	0.038
GSE20715_WT_VS_TLR4_KO_LUNG_UP	196	0.37096664	16.943.752	0.0	5,15E+02	0.394
GSE17301_ACD3_ACD28_VS_ACD3_ACD28_AND_IFNA2_STIM_CD8_TCELL_DN	196	0.36731	16.942.495	0.0	5,16E+02	0.396
GSE7852_THYMUS_VS_FAT_TCONV_DN	194	0.38788766	18.084.189	0.0	5,18E+02	0.039
GSE32986_GMCSF_VS_GMCSF_AND_CURDLAN_LOWDOSE_STIM_DC_DN	179	0.37433827	16.936.569	0.0	5,22E+02	0.399
GSE12366_PLASMA_CELL_VS_NAIVE_BCELL_DN	190	0.36727616	16.937.693	0.0	5,22E+02	0.399
GSE23568_CTRL_VS_ID3_TRANSDUCED_CD8_TCELL_UP	195	0.39092872	18.075.949	0.0	5,29E+02	0.04
GSE2770_TGFB_AND_IL4_VS_IL12_TREATED_ACT_CD4_TCELL_2H_DN	183	0.39510712	1.806.719	0.0	5,42E+02	0.04
GSE6259_FLT3L_INDUCED_DEC205_POS_DC_VS_CD8_TCELL_UP	142	0.40516847	18.031.344	0.0	5,65E+02	0.042
GSE24634_NAIVE_CD4_TCELL_VS_DAY3_IL4_CONV_TREG_UP	185	0.39566475	18.031.883	0.0	5,66E+02	0.042
GSE9988_LPS_VS_CTRL_TREATED_MONOCYTE_UP	177	0.3667316	16.885.817	0.0	5,66E+02	0.427
GSE3039_CD4_TCELL_VS_B1_BCELL_DN	192	0.39132586	17.998.941	0.0	5,73E+02	0.042
GSE24574_BCL6_HIGH_TFH_VS_NAIVE_CD4_TCELL_UP	192	0.36576262	16.878.586	0.0	5,74E+02	0.431
GSE24634_IL4_VS_CTRL_TREATED_NAIVE_CD4_TCELL_DAY7_DN	191	0.367193	1.687.944	0.0	5,74E+02	0.431
GSE557_CIITA_KO_VS_I_AB_KO_DC_UP	193	0.39059284	18.003.126	0.0	5,75E+02	0.042
GSE17974_0H_VS_1H_IN_VITRO_ACT_CD4_TCELL_UP	171	0.3976436	1.800.888	0.0	5,76E+02	0.042
GSE7764_IL15_NK_CELL_24H_VS_SPLENOCYTE_DN	191	0.3696331	16.873.062	0.0	5,76E+02	0.433
GSE36888_UNTREATED_VS_IL2_TREATED_STAT5_AB_KNOCKIN_TCELL_6H_UP	183	0.39166173	18.011.893	0.0	5,76E+02	0.042
GSE30083_SP1_VS_SP2_THYMOCYTE_DN	196	0.390458	17.989.126	0.0	5,84E+02	0.043
GSE7509_DC_VS_MONOCYTE_UP	196	0.3871221	17.992.798	0.0	5,85E+02	0.043
GSE22313_HEALTHY_VS_SLE_MOUSE_CD4_TCELL_DN	193	0.38795966	17.971.448	0.0	5,91E+02	0.044
GSE28726_NAIVE_CD4_TCELL_VS_NAIVE_VA24NEG_NKTCELL_DN	194	0.38603568	179.732	0.0	5,92E+02	0.044
GSE19401_UNSTIM_VS_RETINOIC_ACID_STIM_FOLLICULAR_DC_DN	190	0.38820308	17.973.901	0.0	5,93E+02	0.044
GSE43955_1H_VS_60H_ACT_CD4_TCELL_UP	196	0.38976467	17.975.225	0.0	5,93E+02	0.044
GSE32423_MEMORY_VS_NAIVE_CD8_TCELL_IL7_UP	187	0.39321527	17.976.576	0.0	5,94E+02	0.044
GSE43955_TH0_VS_TGFB_IL6_TH17_ACT_CD4_TCELL_4H_DN	196	0.3894984	17.976.724	0.0	5,95E+02	0.044
GSE15330_HSC_VS_LYMPHOID_PRIMED_MULTIPOTENT_PROGENITOR_DN	177	0.39477935	17.963.971	0.0	6,03E+02	0.045
GSE6259_FLT3L_INDUCED_DEC205_POS_DC_VS_BCELL_DN	156	0.37552908	16.842.868	0.0	6,17E+02	0.46
GSE22886_NAIVE_BCELL_VS_DC_DN	195	0.36125636	16.837.094	0.0	6,19E+02	0.46
GSE33424_CD161_HIGH_VS_NEG_CD8_TCELL_DN	192	0.3643757	16.827.388	0.0	6,27E+02	0.468
GSE24634_NAIVE_CD4_TCELL_VS_DAY5_IL4_CONV_TREG_UP	189	0.36343858	16.823.387	0.0	6,32E+02	0.47
GSE3982_MAST_CELL_VS_TH1_DN	196	0.36368996	16.821.822	0.0	6,33E+02	0.47
GSE37301_CD4_TCELL_VS_RAG2_KO_NK_CELL_UP	142	0.40399885	17.950.983	0.0	6,40E+02	0.048
GSE40274_FOXP3_VS_FOXP3_AND_SATB1_TRANSDUCED_ACTIVATED_CD4_TCELL_UP	125	0.3840368	16.803.486	0.0013869626	6,48E+02	0.477
GSE25088_CTRL_VS_IL4_STIM_MACROPHAGE_UP	178	0.39518753	17.938.589	0.0	6,50E+02	0.049
GSE26669_CD4_VS_CD8_TCELL_IN_MLR_COSTIM_BLOCK_DN	195	0.3619961	1.679.203	0.0	6,58E+02	0.482
GSE28737_FOLLICULAR_VS_MARGINAL_ZONE_BCELL_DN	194	0.36331216	16.793.096	0.0	6,58E+02	0.482
GSE9509_LPS_VS_LPS_AND_IL10_STIM_IL10_KO_MACROPHAGE_20MIN_UP	174	0.38994464	17.928.946	0.0	6,71E+02	0.051
GSE28737_WT_VS_BCL6_KO_FOLLICULAR_BCELL_DN	195	0.36390668	16.777.568	0.0	6,80E+02	0.496
GSE21670_STAT3_KO_VS_WT_CD4_TCELL_IL6_TREATED_DN	191	0.39080045	17.916.682	0.0	6,80E+02	0.052
GSE6259_CD4_TCELL_VS_CD8_TCELL_UP	178	0.3952275	179.202	0.0	6,81E+02	0.052
GSE19923_E2A_KO_VS_HEB_AND_E2A_KO_DP_THYMOCYTE_DN	177	0.3670963	1.675.427	0.0	7,01E+02	0.512
GSE7596_AKT_TRANSD_VS_CTRL_CD4_TCONV_WITH_TGFB_DN	113	0.3891536	16.733.131	0.0014705883	7,32E+02	0.527
GSE2128_C57BL6_VS_NOD_THYMOCYTE_DN	190	0.36358413	16.732.413	0.0	7,34E+02	0.528
GSE21927_SPLEEN_C57BL6_VS_EL4_TUMOR_BALBC_MONOCYTES_UP	192	0.36236194	16.698.363	0.0	7,83E+02	0.557
GSE9509_LPS_VS_LPS_AND_IL10_STIM_IL10_KO_MACROPHAGE_30MIN_UP	182	0.36532775	16.696.919	0.0	7,84E+02	0.558

GSE27241_WT_VS_RORGT_KO_TH17_POLARIZED_CD4_TCELL_TREATED_WITH_DIGOXIN_DN	174	0.37121463	16.691.254	0.0013175231	7,92E+02	0.56
GSE11961_FOLLICULAR_BCELL_VS_GERMINAL_CENTER_BCELL_DAY40_UP	193	0.36154425	16.686.503	0.0	7,99E+02	0.562
GSE22886_NAIVE_CD8_TCELL_VS_MEMORY_TCELL_DN	198	0.38742843	17.861.239	0.0	8,05E+02	0.061
GSE4142_PLASMA_CELL_VS_MEMORY_BCELL_UP	193	0.36180848	1.667.345	0.0013089005	8,22E+02	0.575
GSE26488_WT_VS_VP16_TRANSGENIC_HDAC7_KO_DOUBLE_POSITIVE_THYMOCYTE_DN	161	0.3719252	16.666.412	0.0	8,30E+02	0.577
GSE32901_NAIVE_VS_TH1_CD4_TCELL_UP	123	0.41266978	17.829.053	0.0	8,75E+02	0.067
GSE3039_CD4_TCELL_VS_NKT_CELL_UP	189	0.35994253	16.630.973	0.0013422819	8,82E+02	0.601
GSE19825_CD24LOW_VS_IL2RA_HIGH_DAY3_EFF_CD8_TCELL_UP	188	0.3599255	16.626.036	0.0012970169	8,84E+02	0.601
GSE23321_EFFECTOR_MEMORY_VS_NAIVE_CD8_TCELL_UP	192	0.35995853	16.622.484	0.0	8,85E+02	0.602
GSE3982_MAC_VS_NEUTROPHIL_DN	192	0.36027828	16.622.884	0.0	8,86E+02	0.602
GSE17974_IL4_AND_ANTI_IL12_VS_UNTREATED_1H_ACT_CD4_TCELL_UP	160	0.39348406	17.825.304	0.0	8,86E+02	0.068
GSE20366_TREG_VS_TCONV_DN	195	0.3862004	17.812.343	0.0	9,22E+02	0.071
GSE34006_UNTREATED_VS_A2AR_AGONIST_TREATED_TREG_DN	194	0.38857505	17.805.609	0.0	9,32E+02	0.072
GSE3982_MAC_VS_BASOPHIL_DN	188	0.35876775	16.591.526	0.0	9,38E+02	0.627
GSE7852_TREG_VS_TCONV_LN_DN	192	0.38433272	17.793.238	0.0	9,40E+02	0.073
GSE25123_WT_VS_PPARG_KO_MACROPHAGE_ROSIGLITAZONE_STIM_DN	160	0.37109753	16.589.533	0.0	9,41E+02	0.627
GSE5589_UNSTIM_VS_180MIN_LPS_AND_IL10_STIM_MACROPHAGE_DN	193	0.35870972	16.583.353	0.0	9,49E+02	0.633
GSE18281_CORTEX_VS_MEDULLA_THYMUS_DN	172	0.3873669	17.785.736	0.0013869626	9,51E+02	0.074
GSE2405_0H_VS_3H_A_PHAGOCYTOPHILUM_STIM_NEUTROPHIL_DN	179	0.391827	17.779.711	0.0	9,62E+02	0.075
GSE3982_NEUTROPHIL_VS_BCELL_UP	196	0.35909602	16.574.414	0.0	9,63E+02	0.64
GSE2935_UV_INACTIVATED_VS_LIVE_SENDAI_VIRUS_INF_MACROPHAGE_UP	168	0.36530265	16.573.558	0.0013495276	9,64E+02	0.64
GSE40274_LEF1_VS_FOXP3_AND_LEF1_TRANSDUCED_ACTIVATED_CD4_TCELL_DN	193	0.3597376	16.569.114	0.0013020834	9,68E+02	0.641
GSE7764_NKCELL_VS_SPLENOCYTE_UP	195	0.3595696	16.558.994	0.0	9,88E+02	0.646
GSE35685_CD34POS_CD38NEG_VS_CD34POS_CD10POS_BONE_MARROW_DN	196	0.35745928	16.559.173	0.0	9,88E+02	0.646
GSE6259_DEC205_POS_DC_VS_CD8_TCELL_DN	153	0.37217718	16.554.321	0.0	9,90E+02	0.648
GSE19941_LPS_VS_LPS_AND_IL10_STIM_IL10_KO_NFKBP50_KO_MACROPHAGE_DN	195	0.3546907	16.555.965	0.0	9,90E+02	0.647
KAECH_NAIVE_VS_DAY8_EFF_CD8_TCELL_DN	197	0.35891783	16.553.026	0.0	9,94E+02	0.648
GSE40274_FOXP3_VS_FOXP3_AND_EOS_TRANSDUCED_ACTIVATED_CD4_TCELL_UP	192	0.38722393	1.776.697	0.0	1,01E+03	0.079
GSE3982_CENT_MEMORY_CD4_TCELL_VS_TH2_UP	191	0.40672722	18.611.623	0.0	1,03E+03	0.007
GSE32423_MEMORY_VS_NAIVE_CD8_TCELL_IL7_IL4_UP	192	0.38541603	17.756.807	0.0	1,07E+03	0.082
GSE22886_NAIVE_CD8_TCELL_VS_NEUTROPHIL_DN	186	0.3835567	17.725.544	0.0	1,20E+03	0.094
GSE28726_ACT_CD4_TCELL_VS_ACT_NKTCELL_DN	192	0.38477707	17.722.377	0.0	1,21E+03	0.095
GSE8835_HEALTHY_VS_CLL_CD4_TCELL_UP	191	0.38330737	17.717.803	0.0	1,22E+03	0.096
GSE7460_CTRL_VS_TGFB_TREATED_ACT_CD8_TCELL_DN	189	0.38486105	17.708.302	0.0	1,23E+03	0.097
GSE24210_RESTING_TREG_VS_TCONV_UP	196	0.3817066	17.696.393	0.0	1,28E+03	0.1
GSE22601_IMMATURE_CD4_SINGLE_POSITIVE_VS_DOUBLE_POSITIVE_THYMOCYTE_DN	190	0.38232017	1.769.042	0.0	1,28E+03	0.101
GSE20366_CD103_POS_VS_NEG_TREG_KLRG1NEG_DN	197	0.3802959	17.692.983	0.0	1,29E+03	0.101
GSE45365_NK_CELL_VS_BCELL_DN	134	0.40156317	17.692.988	0.0	1,29E+03	0.101
GSE369_PRE_VS_POST_IL6_INJECTION_SOCS3_KO_LIVER_DN	194	0.38244393	17.679.794	0.0	1,34E+03	0.105
GSE26890_CXCR1_NEG_VS_POS_EFFECTOR_CD8_TCELL_UP	193	0.3844405	17.658.869	0.0	1,44E+03	0.112
GSE9988_LPS_VS_CTRL_TREATED_MONOCYTE_DN	190	0.3823486	17.651.867	0.0	1,47E+03	0.113
GSE24142_EARLY_THYMIC_PROGENITOR_VS_DN2_THYMOCYTE_FETAL_DN	195	0.38151914	1.764.148	0.0	1,49E+03	0.115
GSE3337_CTRL_VS_16H_IFNG_IN_CD8POS_DC_UP	198	0.38058105	1.764.431	0.0	1,49E+03	0.115
GSE43955_TH0_VS_TGFB_IL6_TH17_ACT_CD4_TCELL_30H_UP	195	0.3807261	17.631.669	0.0	1,50E+03	0.116
GSE6269_STAPH_AUREUS_VS_STREP_PNEUMO_INF_PBMC_UP	151	0.38892746	17.634.727	0.0	1,51E+03	0.116
GSE36888_STAT5_AB_KNOCKIN_VS_WT_TCELL_IL2_TREATED_17H_DN	192	0.3813905	17.629.797	0.0	1,51E+03	0.116
GSE3982_EFF_MEMORY_CD4_TCELL_VS_NKCELL_DN	190	0.38422072	17.620.713	0.0	1,52E+03	0.117
GSE11864_CSF1_IFNG_VS_CSF1_PAM3CYS_IN_MAC_UP	193	0.38218483	17.620.822	0.0	1,52E+03	0.117
GSE26495_PD1HIGH_VS_PD1LOW_CD8_TCELL_DN	164	0.3903678	17.620.287	0.0	1,53E+03	0.118
GSE18893_TCONV_VS_TREG_2H_TNF_STIM_DN	182	0.382816	17.613.428	0.0	1,55E+03	0.119
GSE360_T_GONDII_VS_B_MALAYI_LOW_DOSE_DC_UP	191	0.37787437	17.614.722	0.0013003901	1,55E+03	0.119
GSE45365_NK_CELL_VS_BCELL_MCMV_INFECTION_DN	191	0.38149658	17.597.581	0.0	1,59E+03	0.122
GSE20727_CTRL_VS_H2O2_TREATED_DC_DN	180	0.38185203	17.599.833	0.0	1,59E+03	0.122
GSE41867_DAY6_EFFECTOR_VS_DAY30_MEMORY_CD8_TCELL_LCMV_ARMSTRONG_DN	191	0.38178578	17.582.556	0.0	1,65E+03	0.127
GSE44955_MCSF_VS_MCSF_AND_IL27_STIM_MACROPHAGE_UP	190	0.38192773	17.584.233	0.0	1,65E+03	0.127
GSE7831_UNSTIM_VS_CPG_STIM_PDC_1H_UP	197	0.3815038	1.757.589	0.0	1,69E+03	0.129
GSE19888_CTRL_VS_A3R_INHIBITOR_TREATED_MAST_CELL_DN	176	0.38461065	17.566.121	0.0	1,72E+03	0.131
GSE360_L_DONOVANI_VS_B_MALAYI_LOW_DOSE_DC_UP	194	0.37925127	17.564.205	0.0013245033	1,74E+03	0.133
GSE40274_GATA1_VS_FOXP3_AND_GATA1_TRANSDUCED_ACTIVATED_CD4_TCELL_UP	194	0.37878153	17.546.813	0.0	1,78E+03	0.137
GSE37534_GW1929_VS_PIOGLITAZONE_TREATED_CD4_TCELL_PPARG1_FOXP3_TRANSDUCED_UP	193	0.3763092	17.544.547	0.0	1,79E+03	0.138
GSE1460_DP_THYMOCYTE_VS_NAIVE_CD4_TCELL_CORD_BLOOD_DN	190	0.37871408	17.541.034	0.0	1,81E+03	0.14
GSE9988_LOW_LPS_VS_CTRL_TREATED_MONOCYTE_UP	180	0.3847432	17.542.979	0.0	1,81E+03	0.14
GSE4748_LPS_VS_LPS_AND_CYANOBACTERIUM_LPSLIKE_STIM_DC_3H_UP	179	0.38342446	17.527.719	0.0	1,82E+03	0.142
GSE3039_ALPHABETA_CD8_TCELL_VS_B2_BCELL_UP	192	0.38466376	17.521.118	0.0	1,84E+03	0.144
GSE29617_CTRL_VS_DAY7_TIV_FLU_VACCINE_PBMC_2008_UP	182	0.3867471	17.522.441	0.0	1,85E+03	0.144
GSE16385_UNTREATED_VS_12H_ROSIGLITAZONE_IFNG_TNF_TREATED_MACROPHAGE_UP	189	0.38051784	17.514.966	0.0	1,85E+03	0.145
GSE14769_UNSTIM_VS_120MIN_LPS_BMDM_DN	194	0.37803525	17.509.727	0.0	1,86E+03	0.146
GSE22025_UNTREATED_VS_PROGESTERONE_TREATED_CD4_TCELL_UP	190	0.38224912	1.749.349	0.0	1,91E+03	0.151
GSE28726_NAIVE_CD4_TCELL_VS_NAIVE_NKTCELL_DN	198	0.3754095	1.748.434	0.0	1,95E+03	0.155
GSE3982_MAST_CELL_VS_MAC_DN	190	0.37850857	17.481.426	0.0	1,99E+03	0.158
GSE14350_IL2RB_KO_VS_WT_TREG_DN	200	0.3763686	17.466.496	0.0	2,01E+03	0.159
GSE43955_TH0_VS_TGFB_IL6_TH17_ACT_CD4_TCELL_52H_DN	194	0.37693134	17.456.616	0.0	2,05E+03	0.161
GSE25123_IL4_VS_IL4_AND_ROSIGLITAZONE_STIM_PPARG_KO_MACROPHAGE_DAY10_DN	185	0.37614805	17.443.104	0.0	2,05E+03	0.161
GSE3720_LPS_VS_PMA_STIM_VD1_GAMMADELTA_TCELL_UP	137	0.39408886	17.431.884	0.0	2,12E+03	0.166
GSE44649_WT_VS_MIR155_KO_NAIVE_CD8_TCELL_DN	193	0.37931687	17.426.935	0.0	2,13E+03	0.167
GSE17721_PAM3CSK4_VS_GADIQUIMOD_4H_BMDM_DN	196	0.37952405	1.742.071	0.0	2,14E+03	0.168
GSE36009_UNSTIM_VS_LPS_STIM_DC_UP	197	0.37757722	17.411.623	0.0	2,20E+03	0.172
GSE20366_CD103_POS_VS_CD103_KLRG1_DP_TREG_DN	197	0.37853912	17.404.612	0.0	2,27E+03	0.179
GSE28737_BCL6_HET_VS_BCL6_KO_FOLLICULAR_BCELL_DN	194	0.37635157	1.740.231	0.0	2,28E+03	0.18
GSE360_LOW_DOSE_B_MALAYI_VS_M_TUBERCULOSIS_MAC_DN	194	0.37417942	17.387.471	0.0	2,36E+03	0.188
GSE26669_CTRL_VS_COSTIM_BLOCK_MLR_CD4_TCELL_DN	191	0.3748314	17.380.773	0.0	2,37E+03	0.189
GSE19198_6H_VS_24H_IL21_TREATED_TCELL_UP	188	0.37849265	1.737.627	0.0	2,40E+03	0.192
GSE29618_PRE_VS_DAY7_FLU_VACCINE_MDC_UP	190	0.37445074	17.361.977	0.0	2,48E+03	0.194
GSE25088_IL4_VS_IL4_AND_ROSIGLITAZONE_STIM_STAT6_KO_MACROPHAGE_DAY10_DN	190	0.3783663	17.355.566	0.0	2,49E+03	0.195
GSE17974_CTRL_VS_ACT_IL4_AND_ANTI_IL12_72H_CD4_TCELL_UP	183	0.37995666	17.355.734	0.0	2,49E+03	0.195
GSE15930_STIM_VS_STIM_AND_IFNAB_72H_CD8_T_CELL_UP	193	0.37312135	17.336.166	0.0	2,55E+03	0.201
GSE29618_MONOCYTE_VS_PDC_DN	191	0.37706748	17.337.023	0.0	2,55E+03	0.201
GSE12839_CTRL_VS_IL12_TREATED_PBMC_DN	112	0.4050868	17.329.537	0.001472754	2,59E+03	0.205
GSE22432_MULTIPOTENT_PROGENITOR_VS_CDC_UP	197	0.37599027	17.326.639	0.0	2,59E+03	0.206

GSE17721_12H_VS_24H_GARDIQUIMOD_BMDM_UP	197	0.37626135	17.320.517	0.0	2,63E+03	0.209
GSE39820_CTRL_VS_IL1B_IL6_IL23A_CD4_TCELL_DN	195	0.3702546	17.297.505	0.0	2,76E+03	0.217
GSE22886_UNSTIM_VS_IL15_STIM_NKCELL_UP	186	0.37553412	17.280.351	0.0	2,86E+03	0.223
GSE20366_EX_VIVO_VS_DEC205_CONVERSION_NAIVE_CD4_TCELL_UP	195	0.37738943	17.267.835	0.0	2,93E+03	0.23
GSE22886_NAIVE_CD8_TCELL_VS_NKCELL_DN	191	0.3778723	1.725.561	0.0	2,96E+03	0.231
GSE3982_NEUTROPHIL_VS_TH1_UP	192	0.37341008	1.724.609	0.0	3,06E+03	0.24
GSE3982_BASOPHIL_VS_TH1_UP	191	0.37571573	17.240.535	0.0	3,11E+03	0.245
GSE46242_TH1_VS_ANERGIC_TH1_CD4_TCELL_WITH_EGR2_DELETED_UP	189	0.37607518	17.240.415	0.0	3,12E+03	0.247
GSE3920_UNTREATED_VS_IFNG_TREATED_FIBROBLAST_DN	174	0.38023558	17.238.761	0.0	3,13E+03	0.248
GSE22886_NAIVE_BCELL_VS_NEUTROPHIL_UP	194	0.3743073	17.231.891	0.0	3,20E+03	0.254
GSE19401_NAIVE_VS_IMMUNIZED_MOUSE_PLN_FOLLICULAR_DC_UP	193	0.37129906	17.230.023	0.0	3,21E+03	0.255
GSE7764_NKCELL_VS_SPLENOCYTE_DN	197	0.37046194	17.224.498	0.0	3,24E+03	0.258
GSE1432_1H_VS_6H_IFNG_MICROGLIA_DN	196	0.3712212	17.207.916	0.0	3,35E+03	0.265
GSE42021_CD24INT_VS_CD24LOW_TCONV_THYMUS_DN	191	0.37051973	17.203.293	0.0	3,35E+03	0.266
GSE3337_CTRL_VS_16H_IFNG_IN_CD8POS_DC_DN	191	0.37392563	17.188.276	0.0	3,42E+03	0.269
GSE17721_12H_VS_24H_LPS_BMDM_UP	195	0.37613183	17.183.763	0.0	3,43E+03	0.271
GSE2706_R848_VS_LPS_2H_STIM_DC_DN	162	0.3775676	17.176.619	0.0	3,46E+03	0.272
GSE3982_EOSINOPHIL_VS_TH2_UP	188	0.37511456	17.173.927	0.0025974025	3,47E+03	0.273
GSE19941_LPS_VS_LPS_AND_IL10_STIM_IL10_KO_NFKBP50_KO_MACROPHAGE_UP	192	0.37318438	17.168.794	0.0	3,52E+03	0.278
GSE21927_GMCSF_IL6_VS_GMCSF_GCSF_TREATED_BONE_MARROW_UP	166	0.3825105	17.161.032	0.0013477089	3,55E+03	0.281
GSE32901_NAIVE_VS_TH1_CD4_TCELL_DN	162	0.37765437	17.150.065	0.0	3,58E+03	0.283
GSE22886_NAIVE_VS_IGG_IGA_MEMORY_BCELL_UP	189	0.3740779	17.140.766	0.0	3,65E+03	0.289
GSE39110_DAY3_VS_DAY6_POST_IMMUNIZATION_CD8_TCELL_UP	190	0.37359852	17.131.371	0.0	3,73E+03	0.293
GSE411_UNSTIM_VS_100MIN_IL6_STIM_MACROPHAGE_DN	192	0.37037137	17.130.378	0.0	3,73E+03	0.294
GSE21927_C26GM_VS_4T1_TUMOR_MONOCYTE_BALBC_DN	184	0.37351453	17.123.643	0.0	3,79E+03	0.296
GSE3039_CD4_TCELL_VS_B1_BCELL_UP	195	0.36998808	17.119.299	0.0	3,81E+03	0.296
GSE17974_IL4_AND_ANTI_IL12_VS_UNTREATED_72H_ACT_CD4_TCELL_UP	168	0.37536767	17.098.945	0.0	3,93E+03	0.305
GSE43863_DAY6_EFF_VS_DAY150_MEM_TFH_CD4_TCELL_UP	193	0.3697248	17.095.772	0.0	3,96E+03	0.305
GSE5589_LPS_VS_LPS_AND_IL6_STIM_IL6_KO_MACROPHAGE_45MIN_UP	198	0.3697184	17.094.654	0.0	3,96E+03	0.306
GSE360_L_MAJOR_VS_T_GONDII_MAC_UP	191	0.36586967	17.090.694	0.0	3,97E+03	0.307
GSE9988_LPS_VS_VEHICLE_TREATED_MONOCYTE_UP	178	0.37730658	17.088.509	0.0	3,99E+03	0.308
GSE2770_IL12_AND_TGFB_VS_IL4_TREATED_ACT_CD4_TCELL_48H_UP	191	0.37146464	17.087.203	0.0	4,00E+03	0.309
GSE37301_HEMATOPOIETIC_STEM_CELL_VS_RAG2_KO_NK_CELL_UP	169	0.37864596	17.083.174	0.0	4,02E+03	0.31
GSE36527_CD62L_HIGH_CD69_NEG_VS_CD62L_LOW_CD69_POS_TREG_KLRG1_NEG_DN	192	0.36905733	17.075.223	0.0	4,13E+03	0.317
GSE36392_TYPE_2_MYELOID_VS_EOSINOPHIL_IL25_TREATED_LUNG_DN	190	0.368225	17.074.853	0.0	4,13E+03	0.318
GSE7852_TREG_VS_TCONV_FAT_DN	194	0.37285885	17.073.762	0.0	4,14E+03	0.319
GSE25123_CTRL_VS_IL4_STIM_PPARG_KO_MACROPHAGE_DN	193	0.37160107	17.072.835	0.0	4,15E+03	0.321
GSE8921_3H_VS_24H_TLR1_2_STIM_MONOCYTE_UP	192	0.3698379	17.070.295	0.0	4,16E+03	0.321
GSE32533_WT_VS_MIR17_KO_ACT_CD4_TCELL_UP	195	0.36487025	17.062.007	0.0	4,24E+03	0.328
GSE17721_0.5H_VS_24H_CPG_BMDM_UP	196	0.3628996	17.051.774	0.0	4,31E+03	0.333
GSE32986_CURDLAN_HIGHDOSE_VS_GMCSF_AND_CURDLAN_HIGHDOSE_STIM_DC_UP	189	0.3736508	17.048.949	0.0	4,33E+03	0.334
GSE16385_IFNG_TNF_VS_UNSTIM_MACROPHAGE_ROSIGLITAZONE_TREATED_DN	191	0.36979237	17.049.177	0.0	4,33E+03	0.334
GSE22443_NAIVE_VS_ACT_AND_IL12_TREATED_CD8_TCELL_UP	195	0.37147924	17.043.614	0.0	4,38E+03	0.337
GSE32986_CURDLAN_LOWDOSE_VS_CURDLAN_HIGHDOSE_STIM_DC_DN	195	0.3678283	17.028.685	0.0	4,41E+03	0.34
GSE5589_IL6_KO_VS_IL10_KO_LPS_AND_IL10_STIM_MACROPHAGE_180MIN_UP	190	0.37209016	17.026.615	0.0	4,43E+03	0.341
GSE7460_TCONV_VS_TREG_LN_UP	190	0.36565343	16.974.735	0.0	4,84E+03	0.369
GSE13547_CTRL_VS_ANTI_IGM_STIM_ZFX_KO_BCELL_12H_DN	157	0.3757693	16.961.627	0.0	4,94E+03	0.377
GSE360_CTRL_VS_L_MAJOR_DC_DN	196	0.36411902	16.953.831	0.0	5,00E+03	0.382
GSE8835_HEALTHY_VS_CLL_CD8_TCELL_DN	190	0.369747	16.953.919	0.0013623978	5,01E+03	0.382
GSE7460_CTRL_VS_TGFB_TREATED_ACT_TREG_DN	191	0.36428186	16.950.862	0.0	5,02E+03	0.384
GSE369_PRE_VS_POST_IL6_INJECTION_IFNG_WT_LIVER_DN	192	0.36633968	16.936.423	0.0012771392	5,21E+03	0.399
GSE8868_SPLEEN_VS_INTESTINE_CD11B_POS_CD11C_NEG_DC_DN	194	0.36863518	16.936.674	0.0	5,22E+03	0.399
GSE14415_INDUCED_VS_NATURAL_TREG_UP	146	0.3790405	16.930.137	0.0	5,25E+03	0.4
GSE39820_IL1B_IL6_VS_IL1B_IL6_IL23A_TREATED_CD4_TCELL_DN	189	0.36647832	16.918.837	0.0	5,34E+03	0.407
GSE16697_CD4_TCELL_VS_TFH_CD4_TCELL_UP	193	0.36945468	16.911.722	0.0	5,40E+03	0.41
GSE1432_1H_VS_24H_IFNG_MICROGLIA_UP	199	0.36780855	16.911.767	0.0	5,40E+03	0.41
GSE22886_UNSTIM_VS_IL2_STIM_NKCELL_UP	189	0.36888736	16.912.304	0.0	5,41E+03	0.41
GSE25123_WT_VS_PPARG_KO_MACROPHAGE_ROSIGLITAZONE_STIM_UP	191	0.3660713	16.885.263	0.0	5,66E+03	0.427
GSE1791_CTRL_VS_NEUROMEDINU_IN_T_CELL_LINE_12H_DN	158	0.37123147	16.857.562	0.0	5,93E+03	0.443
GSE17301_IFNA2_VS_IFNA2_AND_ACD3_ACD28_STIM_CD8_TCELL_DN	191	0.36521432	16.855.377	0.0	5,99E+03	0.447
GSE3982_DC_VS_BCELL_DN	192	0.36618924	16.833.895	0.0013037809	6,19E+03	0.462
GSE28737_FOLLICULAR_VS_MARGINAL_ZONE_BCELL_BCL6_HET_UP	189	0.36677244	1.683.255	0.0	6,22E+03	0.462
GSE40274_CTRL_VS_FOXP3_AND_HELIOS_TRANSDUCED_ACTIVATED_CD4_TCELL_UP	196	0.36561635	16.827.887	0.0	6,26E+03	0.467
GSE3982_DC_VS_NEUTROPHIL_DN	192	0.36630738	16.825.722	0.0013037809	6,30E+03	0.468
GSE6259_FLT3L_INDUCED_33D1_POS_DC_VS_CD8_TCELL_DN	145	0.37703127	16.817.312	0.0	6,36E+03	0.471
GSE7831_CPG_VS_INFLUENZA_STIM_PDC_1H_DN	199	0.36035922	16.787.622	0.0	6,64E+03	0.485
GSE24210_IL35_TREATED_VS_RESTING_TREG_UP	189	0.3671733	16.783.172	0.0	6,75E+03	0.491
GSE32533_WT_VS_MIR17_OVEREXPRESS_ACT_CD4_TCELL_DN	196	0.3612914	16.766.486	0.0	6,92E+03	0.506
GSE46606_IRF4_KO_VS_WT_CD40L_IL2_IL5_1DAY_STIMULATED_BCELL_DN	184	0.36441195	16.763.787	0.0	6,94E+03	0.508
GSE26495_PD1HIGH_VS_PD1LOW_CD8_TCELL_UP	168	0.37199864	16.756.028	0.0	6,99E+03	0.511
GSE19941_UNSTIM_VS_LPS_AND_IL10_STIM_IL10_KO_NFKBP50_KO_MACROPHAGE_UP	163	0.37140957	1.675.622	0.0013550136	7,00E+03	0.511
GSE7460_WT_VS_FOXP3_HET_ACT_WITH_TGFB_TCONV_UP	190	0.3607413	16.742.563	0.0	7,19E+03	0.52
GSE7548_DAY7_VS_DAY28_PCC_IMMUNIZATION_CD4_TCELL_UP	195	0.36377698	16.730.425	0.0	7,38E+03	0.53
GSE15330_HSC_VS_MEGAKARYOCYTE_ERYTHROID_PROGENITOR_IKAROS_KO_DN	190	0.36011058	16.713.725	0.0	7,67E+03	0.546
GSE2770_UNTREATED_VS_TGFB_AND_IL4_TREATED_ACT_CD4_TCELL_48H_DN	196	0.3557535	16.710.024	0.0	7,73E+03	0.55
GSE2585_THYMIC_DC_VS_THYMIC_MACROPHAGE_DN	189	0.3634492	16.702.305	0.0	7,81E+03	0.555
GSE6259_FLT3L_INDUCED_VS_WT_SPLENIC_DC_33D1_POS_DN	154	0.37333992	16.661.339	0.0	8,35E+03	0.58
GSE22886_NAIVE_CD8_TCELL_VS_DC_DN	196	0.35849634	16.640.434	0.0	8,70E+03	0.595
GSE27786_LIN_NEG_VS_CD4_TCELL_DN	189	0.35914135	16.635.891	0.0	8,77E+03	0.598
GSE21774_CD62L_POS_CD56_BRIGHT_VS_CD62L_NEG_CD56_DIM_NK_CELL_DN	193	0.36038676	16.625.593	0.0	8,84E+03	0.602
GSE43955_TH0_VS_TGFB_IL6_TH17_ACT_CD4_TCELL_1H_UP	195	0.35904586	16.622.275	0.0	8,84E+03	0.602
GSE41176_UNSTIM_VS_ANTI_IGM_STIM_BCELL_1H_UP	196	0.3593062	16.626.258	0.0	8,85E+03	0.601
GSE32901_NAIVE_VS_TH17_NEG_CD4_TCELL_DN	121	0.38326457	16.615.615	0.0	9,03E+03	0.611
GSE43863_TFH_VS_LY6C_INT_CXCR5POS_EFFECTOR_CD4_TCELL_DN	196	0.35942644	16.592.513	0.0	9,39E+03	0.627
GSE37605_TREG_VS_TCONV_C57BL6_FOXP3_FUSION_GFP_DN	164	0.36659005	16.587.563	0.0	9,43E+03	0.629
GSE17974_IL4_AND_ANTI_IL12_VS_UNTREATED_4H_ACT_CD4_TCELL_DN	161	0.36737654	16.579.379	0.0	9,55E+03	0.637
GSE41176_UNSTIM_VS_ANTI_IGM_STIM_BCELL_24H_UP	189	0.3583447	16.564.248	0.0	9,73E+03	0.643
GSE7460_TREG_VS_TCONV_ACT_UP	191	0.38674608	17.758.027	0.0	1,04E+04	0.08
GSE3565_DUSP1_VS_WT_SPLENOCYTES_POST_LPS_INJECTION_DN	148	0.39793614	17.747.124	0.0	1,10E+04	0.085

GSE3039_ALPHAALPHA_CD8_TCELL_VS_B1_BCELL_UP	198	0.38357833	17.741.715	0.0	1,11E+04	0.086
GSE32533_MIR17_KO_VS_MIR17_OVEREXPRESS_ACT_CD4_TCELL_DN	187	0.3874007	17.729.868	0.0	1,17E+04	0.091
GSE23505_UNTREATED_VS_4DAY_IL6_IL1_IL23_TREATED_CD4_TCELL_UP	190	0.38547206	17.725.484	0.0	1,20E+04	0.094
GSE15330_HSC_VS_MEGAKARYOCYTE_ERYTHROID_PROGENITOR_IKAROS_KO_UP	192	0.35646927	1.654.659	0.0	0.001003823	0.654
GSE14769_UNSTIM_VS_40MIN_LPS_BMDM_DN	192	0.35781357	16.541.978	0.0	0.001012974	0.658
GSE9960_HEALTHY_VS_GRAM_NEG_SEPSIS_PBMC_DN	190	0.35687467	16.531.743	0.0	0.001028394	0.663
GSE23321_CENTRAL_MEMORY_VS_NAIVE_CD8_TCELL_DN	190	0.35493118	16.536.756	0.0	0.001028542	0.663
GSE23308_WT_VS_MINERALCORTICOID_REC_KO_MACROPHAGE_DN	193	0.360813	16.530.229	0.0	0.001029197	0.663
GSE37301_MULTIPOTENT_PROGENITOR_VS_COMMON_LYMPHOID_PROGENITOR_UP	163	0.3633002	1.653.192	0.001314060	0.001029350	0.663
GSE14415_NATURAL_TREG_VS_FOXP3_KO_NATURAL_TREG_DN	144	0.37585503	16.532.764	0.001358695	0.001029382	0.663
GSE2585_THYMIC_MACROPHAGE_VS_MTEC_DN	190	0.3600589	16.530.381	0.0	0.001030152	0.663
GSE13522_WT_VS_IFNAR_KO_SKIN_UP	192	0.359066	16.533.256	0.0	0.001030341	0.663
GSE7831_1H_VS_4H_CPG_STIM_PDC_DN	191	0.3563299	16.522.185	0.0	0.001043689	0.669
GSE17721_CPG_VS_GARDIQUIMOD_1H_BMDM_UP	194	0.35546952	16.522.582	0.0	0.001043746	0.668
GSE22935_WT_VS_MYD88_KO_MACROPHAGE_DN	192	0.35508808	16.520.034	0.0	0.001044550	0.669
GSE36891_UNSTIM_VS_POLYIC_TLR3_STIM_PERITONEAL_MACROPHAGE_DN	195	0.3573251	16.518.837	0.0	0.001046327	0.671
GSE4590_PRE_BCELL_VS_LARGE_PRE_BCELL_UP	153	0.37117246	16.508.355	0.001381215	0.001058960	0.676
GSE20727_CTRL_VS_H2O2_TREATED_DC_UP	185	0.3560616	16.507.686	0.001267427	0.001059800	0.676
GSE3982_MAC_VS_TH1_UP	189	0.35746798	16.505.764	0.0	0.001064257	0.68
GSE4142_NAIVE_VS_GC_BCELL_UP	192	0.35851654	16.501.002	0.0	0.001075898	0.683
GSE46242_CTRL_VS_EGR2_DELETED_ANERGIC_TH1_CD4_TCELL_UP	190	0.36073428	16.501.006	0.001302083	0.001076888	0.683
GSE21063_CTRL_VS_ANTI_IGM_STIM_BCELL_NFATC1_KO_3H_UP	196	0.36129618	16.496.714	0.0	0.001080335	0.684
GSE27241_WT_CTRL_VS_DIGOXIN_TREATED_RORGT_KO_CD4_TCELL_IN_TH17_POLARIZING_CONDITIONS_UP	169	0.36390206	16.493.697	0.001356852	0.001090190	0.687
GSE17301_CTRL_VS_48H_ACD3_ACD28_IFNA5_STIM_CD8_TCELL_UP	194	0.3584164	1.649.093	0.001310616	0.001090996	0.688
GSE19401_UNSTIM_VS_RETINOIC_ACID_STIM_FOLLICULAR_DC_UP	196	0.35831657	1.648.576	0.0	0.001101712	0.693
GSE32986_UNSTIM_VS_GMCSF_AND_CURDLAN_HIGHDOSE_STIM_DC_DN	176	0.36125848	1.647.868	0.0	0.001117800	0.704
GSE43955_TH0_VS_TGFB_IL6_TH17_ACT_CD4_TCELL_4H_UP	198	0.35353082	1.647.604	0.0	0.001122191	0.705
GSE17974_CTRL_VS_ACT_IL4_AND_ANTI_IL12_2H_CD4_TCELL_UP	181	0.35894418	16.456.703	0.001349527	0.001154477	0.717
GSE2770_TGFB_AND_IL4_ACT_VS_ACT_CD4_TCELL_48H_DN	187	0.35958636	16.455.805	0.0	0.001157009	0.719
GSE45365_NK_CELL_VS_BCELL_MCMV_INFECTION_UP	191	0.35443735	16.453.525	0.0	0.001159551	0.721
GSE6259_33D1_POS_DC_VS_CD8_TCELL_DN	124	0.37707087	16.444.343	0.001377410	0.001183475	0.73
GSE10240_IL22_VS_IL22_AND_IL17_STIM_PRIMARY_BRONCHIAL_EPITHELIAL_CELLS_UP	194	0.3537748	1.644.468	0.0	0.001184553	0.73
GSE37301_MULTIPOTENT_PROGENITOR_VS_LYMPHOID_PRIMED_MPP_DN	178	0.35797536	16.441.419	0.001302083	0.001187759	0.734
GSE6092_IFNG_VS_IFNG_AND_B_BURGDORFERI_INF_ENDOTHELIAL_CELL_DN	172	0.36488745	16.437.029	0.0	0.001195651	0.738
GSE7852_THYMUS_VS_FAT_TREG_DN	193	0.35633174	16.431.979	0.001317523	0.001201697	0.739
GSE1925_CTRL_VS_24H_IFNG_STIM_MACROPHAGE_UP	187	0.35868075	16.430.824	0.001295336	0.001203305	0.74
GSE36476_CTRL_VS_TSST_ACT_72H_MEMORY_CD4_TCELL_OLD_UP	190	0.35554767	16.422.814	0.0	0.001218308	0.746
GSE6269_FLU_VS_STAPH_AUREUS_INF_PBMC_UP	167	0.36394945	16.420.764	0.0	0.001223232	0.747
GSE2405_0H_VS_6H_A_PHAGOCYTOPHILUM_STIM_NEUTROPHIL_DN	172	0.36321324	16.421.145	0.0	0.001224339	0.747
GSE17974_CTRL_VS_ACT_IL4_AND_ANTI_IL12_0.5H_CD4_TCELL_DN	170	0.36208084	16.412.832	0.0	0.001237244	0.753
GSE3982_MAC_VS_NKCELL_DN	190	0.35559168	16.410.604	0.001328021	0.001240327	0.755
GSE33425_CD8_ALPHAALPHA_VS_ALPHABETA_CD161_HIGH_TCELL_DN	196	0.35243088	16.409.955	0.001298701	0.001240966	0.755
GSE22886_IGA_VS_IGM_MEMORY_BCELL_DN	192	0.3546334	16.410.867	0.0	0.001241447	0.755
GSE24634_TREG_VS_TCONV_POST_DAY10_IL4_CONVERSION_UP	196	0.35479486	16.401.119	0.0	0.001254892	0.765
GSE40666_WT_VS_STAT4_KO_CD8_TCELL_WITH_IFNA_STIM_90MIN_UP	195	0.35368407	16.398.723	0.0	0.001263504	0.768
GSE22886_NAIVE_CD4_TCELL_VS_NEUTROPHIL_DN	189	0.35420102	16.395.522	0.001360544	0.001268327	0.77
GSE3982_BCELL_VS_NKCELL_UP	190	0.3579518	16.395.997	0.001326259	0.001268594	0.77
GSE17301_CTRL_VS_48H_IFNA2_STIM_CD8_TCELL_DN	189	0.3577782	1.639.374	0.0	0.001271610	0.772
GSE17721_0.5H_VS_12H_CPG_BMDM_DN	195	0.35569975	16.391.428	0.0	0.001275511	0.774
GSE4590_LARGE_PRE_BCELL_VS_VPREB_POS_LARGE_PRE_BCELL_UP	168	0.35971698	1.639.193	0.001369863	0.001276654	0.774
GSE36891_POLYIC_TLR3_VS_PAM_TLR2_STIM_PERITONEAL_MACROPHAGE_UP	145	0.36793742	16.390.651	0.0	0.001277017	0.775
GSE19888_CTRL_VS_A3R_ACT_TREATED_MAST_CELL_PRETREATED_WITH_A3R_INH_UP	192	0.35669464	16.386.327	0.0	0.001289954	0.777
GSE21670_IL6_VS_TGFB_AND_IL6_TREATED_CD4_TCELL_UP	181	0.36031106	16.378.143	0.0	0.001303489	0.779
GSE29618_BCELL_VS_PDC_DN	194	0.3511899	163.787	0.0	0.001303772	0.779
GSE10273_HIGH_VS_LOW_IL7_TREATED_IRF4_8_NULL_PRE_BCELL_DN	195	0.35043606	16.364.027	0.0	0.001336592	0.786
GSE7460_CTRL_VS_TGFB_TREATED_ACT_FOXP3_MUT_TCONV_DN	193	0.35065144	16.362.865	0.0	0.001339790	0.786
GSE22935_24H_VS_48H_MBOVIS_BCG_STIM_MYD88_KO_MACROPHAGE_UP	193	0.35849312	16.355.622	0.0	0.001353471	0.789
GSE40277_EOS_AND_LEF1_TRANSDUCED_VS_GATA1_AND_SATB1_TRANSDUCED_CD4_TCELL_UP	196	0.35277304	16.342.924	0.0	0.001389998	0.803
GSE17721_LPS_VS_GARDIQUIMOD_2H_BMDM_UP	196	0.35368478	16.331.182	0.0	0.001422567	0.809
GSE7460_CTRL_VS_TGFB_TREATED_ACT_FOXP3_HET_TCONV_DN	192	0.35186574	1.633.146	0.001287001	0.001422953	0.809
GSE41867_DAY6_VS_DAY8_LCMV_CLONE13_EFFECTOR_CD8_TCELL_UP	188	0.35026187	16.328.982	0.001288659	0.001424801	0.811
GSE6269_HEALTHY_VS_FLU_INF_PBMC_DN	156	0.36336645	16.316.586	0.0	0.001456680	0.817
GSE46606_DAY1_VS_DAY3_CD40L_IL2_IL5_STIMULATED_BCELL_DN	183	0.35689887	16.314.851	0.0	0.001461482	0.817
GSE43955_TH0_VS_TGFB_IL6_IL23_TH17_ACT_CD4_TCELL_60H_DN	194	0.3531379	16.309.795	0.0	0.001475002	0.822
GSE24492_LYVE_NEG_VS_POS_MACROPHAGE_DN	191	0.35446405	16.296.802	0.0	0.001513772	0.831
GSE7460_CTRL_VS_TGFB_TREATED_ACT_TCONV_UP	195	0.3563169	16.291.643	0.0	0.001521984	0.831
GSE11961_GERMINAL_CENTER_BCELL_DAY7_VS_MEMORY_BCELL_DAY40_UP	192	0.35241818	16.289.363	0.0	0.001524121	0.832
GSE37301_MULTIPOTENT_PROGENITOR_VS_GRAN_MONO_PROGENITOR_DN	196	0.35030016	16.285.226	0.0	0.001532332	0.834
GSE17721_PAM3CSK4_VS_CPG_12H_BMDM_DN	195	0.35559288	16.283.711	0.001305483	0.001534435	0.834
GSE2770_IL4_ACT_VS_ACT_CD4_TCELL_6H_UP	177	0.35877174	16.283.009	0.0	0.001535684	0.834
GSE3982_BASOPHIL_VS_CENT_MEMORY_CD4_TCELL_UP	191	0.35338557	16.281.449	0.002680965	0.001537827	0.835
GSE22140_HEALTHY_VS_ARTHRITIC_GERMFREE_MOUSE_CD4_TCELL_UP	198	0.34901494	16.271.516	0.0	0.001573598	0.844
GSE36826_NORMAL_VS_STAPH_AUREUS_INF_SKIN_DN	195	0.34921303	16.268.238	0.0	0.001580859	0.845
GSE3720_LPS_VS_PMA_STIM_VD1_GAMMADELTA_TCELL_DN	170	0.35553506	16.267.344	0.0	0.001585519	0.847
GSE26559_TCF1_KO_VS_WT_LIN_NEG_CELL_UP	193	0.35572013	16.266.397	0.001291989	0.001586723	0.847
GSE40274_FOXP3_VS_FOXP3_AND_LEF1_TRANSDUCED_ACTIVATED_CD4_TCELL_UP	189	0.35329837	16.264.821	0.001270648	0.001588806	0.847
GSE36078_WT_VS_IL1R_KO_LUNG_DC_AFTER_AD5_INF_DN	195	0.35603327	16.259.509	0.0	0.001598057	0.85
GSE3720_UNSTIM_VS_LPS_STIM_VD1_GAMMADELTA_TCELL_UP	191	0.35022524	16.259.785	0.0	0.001598588	0.849
GSE33424_CD161_HIGH_VS_INT_CD8_TCELL_UP	197	0.35067347	1.625.447	0.0	0.001606403	0.852
GSE21927_SPLENIC_VS_TUMOR_MONOCYTES_FROM_C26GM_TUMOROUS_MICE_BALBC_DN	192	0.35093486	16.255.175	0.0	0.001606940	0.851
GSE32034_UNTREATED_VS_ROSIGLIZATONE_TREATED_LY6C_LOW_MONOCYTE_UP	163	0.3564429	16.249.472	0.0	0.001611863	0.853
GSE36392_EOSINOPHIL_VS_MAC_IL25_TREATED_LUNG_UP	193	0.3513381	16.245.375	0.0	0.001627610	0.857
GSE16450_IMMATURE_VS_MATURE_NEURON_CELL_LINE_6H_IFNA_STIM_UP	192	0.3510117	16.238.024	0.0	0.001652707	0.862
GSE20727_ROS_INH_VS_ROS_INH_AND_DNFB_ALLERGEN_TREATED_DC_DN	171	0.35785493	16.227.683	0.001383125	0.001682077	0.867
GSE26343_UNSTIM_VS_LPS_STIM_MACROPHAGE_DN	195	0.35288233	162.271	0.0	0.001687479	0.867
GSE9960_GRAM_NEG_VS_GRAM_POS_SEPSIS_PBMC_UP	185	0.35511458	16.223.967	0.0	0.001693733	0.869
GSE21063_WT_VS_NFATC1_KO_16H_ANTI_IGM_STIM_BCELL_DN	171	0.35785672	16.223.418	0.002717391	0.001695689	0.869
GSE10239_NAIVE_VS_DAY4.5_EFF_CD8_TCELL_UP	192	0.34991243	16.220.772	0.001326259	0.001700204	0.869
GSE40274_CTRL_VS_IRF4_TRANSDUCED_ACTIVATED_CD4_TCELL_UP	157	0.3578501	16.201.277	0.002642008	0.001757643	0.879

GSE26495_NAIVE_VS_PD1HIGH_CD8_TCELL_UP	172	0.3547902	16.198.618	0.001345895	0.0017604165	0.879
GSE35825_IFNA_VS_IFNG_STIM_MACROPHAGE_UP	154	0.36204886	16.196.504	0.0	0.0017691373	0.881
GSE17580_UNINFECTED_VS_S_MANSONI_INF_TREG_UP	196	0.3479172	16.194.106	0.0	0.0017752863	0.882
GSE411_UNSTIM_VS_100MIN_IL6_STIM_MACROPHAGE_UP	190	0.35046268	16.192.234	0.0	0.0017754597	0.882
GSE24574_BCL6_HIGH_TFH_VS_TFH_CD4_TCELL_DN	196	0.35006225	16.188.163	0.0	0.0017841398	0.885
GSE3982_EOSINOPHIL_VS_NEUTROPHIL_DN	191	0.3485842	1.618.701	0.0013550136	0.0017842962	0.885
GSE1925_CTRL_VS_IFNG_PRIMED_MACROPHAGE_3H_IFNG_STIM_DN	121	0.3732342	16.179.277	0.0	0.0018056332	0.887
GSE3982_NEUTROPHIL_VS_EFF_MEMORY_CD4_TCELL_UP	195	0.34878805	16.176.957	0.0	0.0018100231	0.887
GSE25123_CTRL_VS_IL4_STIM_MACROPHAGE_UP	189	0.3539607	16.173.124	0.0	0.0018236908	0.89
GSE27670_CTRL_VS_BLIMP1_TRANSDUCED_GC_BCELL_UP	193	0.35278708	16.171.435	0.0	0.0018289224	0.89
GSE15330_HSC_VS_LYMPHOID_PRIMED_MULTIPOTENT_PROGENITOR_UP	196	0.345721	1.616.142	0.0	0.0018737501	0.895
GSE360_T_GONDII_VS_M_TUBERCULOSIS_MAC_DN	192	0.35315332	16.152.806	0.0	0.0018966063	0.899
GSE7852_LN_VS_THYMUS_TCONV_UP	193	0.35093975	16.150.917	0.0013422819	0.0019009216	0.9
GSE39110_UNTREATED_VS_IL2_TREATED_CD8_TCELL_DAY3_POST_IMMUNIZATION_UP	193	0.35253635	16.147.166	0.0	0.0019093879	0.901
GSE10239_KLRG1INT_VS_KLRG1HIGH_EFF_CD8_TCELL_DN	191	0.35091335	16.144.522	0.0	0.0019111317	0.901
GSE45881_CXCR6HI_VS_CXCR1LO_COLONIC_LAMINA_PROPRIA_DN	196	0.34656873	16.142.142	0.0	0.0019120198	0.901
GSE2706_2H_VS_8H_R848_STIM_DC_UP	182	0.3571854	16.139.671	0.0	0.0019229649	0.903
GSE37533_UNTREATED_VS_PIOGLIZATONE_TREATED_CD4_TCELL_PPARG1_AND_FOXP3_TRASDUCED_DN	197	0.35061058	16.129.911	0.0	0.001954066	0.907
GSE5589_LPS_AND_IL10_VS_LPS_AND_IL6_STIM_MACROPHAGE_45MIN_DN	192	0.34713382	16.111.295	0.0012836971	0.0020094048	0.911
GSE17721_POLYIC_VS_CPG_8H_BMDM_UP	193	0.34734952	16.096.869	0.0	0.0020612485	0.918
GSE16385_ROSIGLITAZONE_IL4_VS_ROSIGLITAZONE_ALONE_STIM_MACROPHAGE_UP	187	0.3487724	16.085.727	0.0	0.0020953824	0.92
GSE19825_NAIVE_VS_DAY3_EFF_CD8_TCELL_UP	195	0.34952545	16.085.752	0.0013175231	0.0020971627	0.92
GSE16450_CTRL_VS_IFNA_6H_STIM_IMMATURE_NEURON_CELL_LINE_UP	191	0.34827378	16.081.786	0.0	0.0021078058	0.92
GSE42088_UNINF_VS_LEISHMANIA_INF_DC_8H_UP	197	0.35006207	16.078.002	0.0025575447	0.0021235566	0.926
GSE3920_UNTREATED_VS_IFNA_TREATED_FIBROBLAST_UP	164	0.35651258	16.072.294	0.0	0.002140159	0.926
GSE9650_NAIVE_VS_EXHAUSTED_CD8_TCELL_DN	191	0.3460081	16.069.622	0.0013262599	0.002152523	0.927
GSE43955_TGFB_IL6_VS_TGFB_IL6_IL23_TH17_ACT_CD4_TCELL_60H_DN	194	0.3479365	16.065.005	0.0	0.0021674675	0.928
GSE17301_IFNA2_VS_IFNA2_AND_ACD3_ACD28_STIM_CD8_TCELL_UP	197	0.3488795	16.061.252	0.001285347	0.0021789959	0.93
GSE27291_6H_VS_7D_STIM_GAMMADELTA_TCELL_DN	169	0.35483328	16.056.001	0.0013280213	0.0021985916	0.931
GSE13306_RA_VS_UNTREATED_TREG_DN	188	0.3512316	16.056.429	0.0	0.0021987823	0.931
GSE339_CD8POS_VS_CD4CD8DN_DC_DN	197	0.34633565	16.055.293	0.0	0.0022000456	0.931
GSE25123_ROSIGLITAZONE_VS_IL4_AND_ROSIGLITAZONE_STIM_PPARG_KO_MACROPHAGE_DAY10_DN	188	0.34788102	16.053.026	0.0	0.0022048287	0.931
GSE21063_CTRL_VS_ANTI_IGM_STIM_BCELL_NFATC1_KO_3H_DN	179	0.35016412	16.051.372	0.0	0.0022129677	0.932
GSE7509_DC_VS_MONOCYTE_DN	193	0.34619808	16.050.166	0.0	0.0022133817	0.932
GSE29949_MICROGLIA_VS_DC_BRAIN_DN	193	0.34989187	16.050.909	0.0013297872	0.0022135896	0.932
GSE22886_DC_VS_MONOCYTE_UP	195	0.34942615	16.047.834	0.0013245033	0.0022222574	0.934
GSE6681_DELETED_FOXP3_VS_WT_TREG_UP	188	0.34937862	16.041.328	0.0	0.0022476201	0.937
GSE44649_WT_VS_MIR155_KO_ACTIVATED_CD8_TCELL_UP	189	0.3477387	16.038	0.0013531799	0.0022556835	0.938
GSE13522_CTRL_VS_T_CRUZI_BRAZIL_STRAIN_INF_SKIN_DN	193	0.34561995	16.036.763	0.0	0.0022612521	0.939
GSE23505_IL6_IL1_IL23_VS_IL6_IL1_TGFB_TREATED_CD4_TCELL_UP	195	0.34500614	16.035.691	0.0	0.0022626473	0.939
GSE33424_CD161_HIGH_VS_INT_CD8_TCELL_DN	192	0.34482026	16.032.602	0.0	0.0022704105	0.941
GSE3920_UNTREATED_VS_IFNG_TREATED_ENDOTHELIAL_CELL_UP	148	0.35975635	16.033.025	0.0013661202	0.0022706601	0.94
GSE13738_RESTING_VS_BYSTANDER_ACTIVATED_CD4_TCELL_DN	189	0.35040408	16.025.746	0.0	0.002294494	0.943
GSE5589_UNSTIM_VS_45MIN_LPS_AND_IL6_STIM_MACROPHAGE_UP	192	0.3464629	16.026.098	0.0013623978	0.002294746	0.943
GSE9509_LPS_VS_LPS_AND_IL10_STIM_IL10_KO_MACROPHAGE_30MIN_DN	185	0.35161808	16.023.326	0.0012936611	0.0022964317	0.943
GSE13547_WT_VS_ZFX_KO_BCELL_ANTI_IGM_STIM_12H_DN	142	0.36096635	16.023.682	0.0	0.0022975188	0.943
GSE20727_H2O2_VS_ROS_INHIBITOR_TREATED_DC_UP	189	0.3481701	16.022.563	0.0013568521	0.0023010678	0.943
GSE6674_UNSTIM_VS_ANTI_IGM_STIM_BCELL_DN	121	0.37079442	16.013.771	0.004166667	0.0023376336	0.945
GSE7460_CTRL_VS_TGFB_TREATED_ACT_TCONV_DN	194	0.34491625	16.009.536	0.0025673942	0.0023495958	0.946
GSE5542_IFNG_VS_IFNA_TREATED_EPITHELIAL_CELLS_24H_DN	193	0.34974867	16.006.942	0.0012970169	0.0023533853	0.946
GSE43955_10H_VS_30H_ACT_CD4_TCELL_UP	195	0.34443077	16.006.509	0.0	0.0023538773	0.946
GSE25677_R848_VS_MPL_AND_R848_STIM_BCELL_DN	158	0.35200948	16.001.441	0.0	0.0023698735	0.946
GSE19888_CTRL_VS_TCELL_MEMBRANES_ACT_MAST_CELL_PRETREAT_A3R_INH_UP	194	0.3465387	16.000.339	0.0013386881	0.0023711696	0.946
GSE36476_YOUNG_VS_OLD_DONOR_MEMORY_CD4_TCELL_40H_TSST_ACT_UP	189	0.34918168	15.998.888	0.0	0.0023773555	0.948
GSE30153_LUPUS_VS_HEALTHY_DONOR_BCELL_UP	193	0.34516436	1.599.703	0.002617801	0.002379114	0.948
GSE5589_LPS_VS_LPS_AND_IL6_STIM_MACROPHAGE_45MIN_UP	196	0.34614512	15.997.669	0.0013089005	0.0023794624	0.948
GSE17186_NAIVE_VS_CD21HIGH_TRANSITIONAL_BCELL_UP	186	0.35144505	15.996.317	0.0	0.0023812042	0.948
GSE15930_NAIVE_VS_48H_IN_VITRO_STIM_CD8_TCELL_UP	194	0.35093868	15.988.144	0.0012870013	0.0024125052	0.949
GSE11961_FOLLICULAR_BCELL_VS_MEMORY_BCELL_DAY40_DN	195	0.3469959	15.984.579	0.0	0.002428352	0.95
GSE28737_FOLLICULAR_VS_MARGINAL_ZONE_BCELL_BCL6_HET_DN	191	0.34621057	15.974.807	0.0012722646	0.0024757811	0.954
GSE5679_PPARG_LIGAND_ROSIGLITAZONE_VS_ROSIGLITAZONE_AND_RARA_AGONIST_AM580_TREATED_DC_UP	192	0.34670967	15.972.878	0.0	0.0024797884	0.954
GSE20366_EX_VIVO_VS_HOMEOSTATIC_CONVERSION_NAIVE_CD4_TCELL_DN	192	0.3429698	15.973.163	0.0013262599	0.0024810142	0.954
GSE24142_DN2_VS_DN3_THYMOCYTE_DN	198	0.34502983	15.971.129	0.0	0.002485849	0.954
GSE28130_ACTIVATED_VS_INDUCEED_TREG_UP	187	0.35028222	15.969.357	0.0	0.0024902674	0.955
KAECH_DAY8_EFF_VS_DAY15_EFF_CD8_TCELL_DN	193	0.3476966	15.967.995	0.0014104373	0.0024946763	0.955
GSE17186_NAIVE_VS_CD21LOW_TRANSITIONAL_BCELL_DN	192	0.3495704	15.966.954	0.0	0.0024998947	0.956
GSE30971_CTRL_VS_LPS_STIM_MACROPHAGE_WBP7_KO_2H_UP	180	0.34831885	15.966.343	0.0	0.0025002712	0.956
GSE15624_CTRL_VS_3H_HALOFUGINONE_TREATED_CD4_TCELL_UP	153	0.35546744	15.964.634	0.004103967	0.0025038582	0.956
GSE18893_TCONV_VS_TREG_24H_CULTURE_UP	195	0.34269813	15.962.156	0.0	0.0025130957	0.958
GSE30971_CTRL_VS_LPS_STIM_MACROPHAGE_WBP7_HET_4H_DN	186	0.34873274	15.960.118	0.0	0.0025207128	0.958
GSE40274_FOXP3_VS_FOXP3_AND_PBX1_TRANSDUCED_ACTIVATED_CD4_TCELL_DN	196	0.3466612	15.959.238	0.0	0.0025234895	0.958
GSE29164_DAY3_VS_DAY7_CD8_TCELL_AND_IL12_TREATED_MELANOMA_DN	193	0.34602258	15.957.105	0.0012987013	0.0025302737	0.959
GSE27859_MACROPHAGE_VS_CD11C_INT_F480_INT_DC_DN	189	0.34402263	15.953.035	0.001312336	0.002546626	0.96
GSE15930_STIM_VS_STIM_AND_IFNAB_48H_CD8_T_CELL_UP	193	0.34381217	15.947.533	0.0	0.0025575624	0.961
GSE11057_NAIVE_VS_EFF_MEMORY_CD4_TCELL_UP	179	0.34815958	1.594.923	0.0013227513	0.0025577077	0.96
GSE17186_NAIVE_VS_CD21LOW_TRANSITIONAL_BCELL_CORD_BLOOD_UP	194	0.34222394	1.594.804	0.0026041667	0.002558837	0.961
GSE3982_BCELL_VS_EFF_MEMORY_CD4_TCELL_UP	194	0.34673136	1.594.971	0.0	0.0025589904	0.96
GSE39152_CD103_NEG_VS_POS_MEMORY_CD8_TCELL_UP	200	0.34380567	1.594.294	0.0	0.0025754997	0.963
GSE21670_UNTREATED_VS_TGFB_IL6_TREATED_STAT3_KO_CD4_TCELL_DN	191	0.34618032	15.942.452	0.0	0.0025766036	0.963
GSE36826_NORMAL_VS_STAPH_AUREUS_INF_IL1R_KO_SKIN_UP	194	0.34263337	15.935.928	0.0013157895	0.0026088294	0.965
GSE7460_FOXP3_MUT_VS_HET_ACT_TCONV_UP	194	0.34840375	15.931.174	0.0025839794	0.0026290645	0.967
GSE3982_DC_VS_MAC_LPS_STIM_DN	194	0.3427024	15.926.788	0.0	0.0026460304	0.97
GSE15624_CTRL_VS_6H_HALOFUGINONE_TREATED_CD4_TCELL_UP	124	0.36458236	1.592.005	0.004037685	0.0026708958	0.972
GSE19198_CTRL_VS_IL21_TREATED_TCELL_1H_DN	189	0.34541893	15.912.548	0.0	0.0026983966	0.973
GSE360_DC_VS_MAC_L_MAJOR_DN	194	0.34474364	1.591.326	0.0	0.0026989682	0.973
GSE29949_CD8_NEG_DC_SPLEEN_VS_DC_BRAIN_DN	195	0.34545377	1.590.268	0.0013280213	0.002737435	0.974
GSE22601_CD4_SINGLE_POSITIVE_VS_CD8_SINGLE_POSITIVE_THYMOCYTE_DN	197	0.3426183	1.589.756	0.0026631157	0.0027582173	0.974
GSE3982_MAC_VS_CENT_MEMORY_CD4_TCELL_UP	193	0.34044138	15.894.479	0.0	0.0027678742	0.974
GSE19198_CTRL_VS_IL21_TREATED_TCELL_24H_UP	196	0.3431408	15.890.611	0.0	0.0027815015	0.974

GSE3982_DC_VS_BASOPHIL_DN	191	0.34253913	15.887.362	0.0	0.002786656	0.975
GSE24081_CONTROLLER_VS_PROGRESSOR_HIV_SPECIFIC_CD8_TCELL_DN	194	0.3469444	15.886.465	0.0	0.002786786	0.976
GSE40274_FOXP3_VS_FOXP3_AND_HELIOS_TRANSDUCED_ACTIVATED_CD4_TCELL_UP	137	0.359893	1.588.852	0.0	0.002787189	0.974
GSE40666_WT_VS_STAT1_KO_CD8_TCELL_UP	195	0.34186572	15.887.688	0.002604166	0.002788106	0.975
GSE22229_RENAL_TRANSPLANT_VS_HEALTHY_PBMC_DN	188	0.34844574	15.883.076	0.0	0.002793482	0.977
GSE22886_UNSTIM_VS_STIM_MEMORY_TCELL_UP	188	0.34760356	1.588.522	0.0	0.002794013	0.976
GSE15930_STIM_VS_STIM_AND_IFNAB_48H_CD8_T_CELL_DN	196	0.34523344	15.883.431	0.001358695	0.002795715	0.977
GSE10239_MEMORY_VS_DAY4.5_EFF_CD8_TCELL_UP	191	0.34445694	15.870.929	0.001275510	0.002858829	0.98
GSE14699_NAIVE_VS_DELETIONAL_TOLERANCE_CD8_TCELL_UP	146	0.35617912	15.869.333	0.001396648	0.002862179	0.981
GSE15930_NAIVE_VS_24H_IN_VITRO_STIM_IL12_CD8_TCELL_UP	198	0.3434552	15.870.132	0.001300390	0.002863602	0.981
GSE29949_MICROGLIA_BRAIN_VS_MONOCYTE_BONE_MARROW_DN	193	0.34139407	15.869.561	0.0	0.002864460	0.981
GSE46606_UNSTIM_VS_CD40L_IL2_IL5_1DAY_STIMULATED_IRF4HIGH_SORTED_BCELL_DN	189	0.34557897	1.586.406	0.0	0.002885758	0.982
GSE39916_B_CELL_SPLEEN_VS_PLASMA_CELL_BONE_MARROW_UP	193	0.34072027	15.856.742	0.0	0.002915479	0.982
GSE3982_DC_VS_NKCELL_UP	195	0.34134042	15.851.426	0.0	0.002936647	0.982
GSE2770_UNTREATED_VS_TGFB_AND_IL4_TREATED_ACT_CD4_TCELL_4H_UP	191	0.34510297	15.851.563	0.001317523	0.002938979	0.982
GSE1460_INTRATHYMIC_T_PROGENITOR_VS_NAIVE_CD4_TCELL_CORD_BLOOD_DN	195	0.34158763	15.846.312	0.0	0.002956956	0.984
GSE32986_GMCSF_VS_GMCSF_AND_CURDLAN_HIGHDOSE_STIM_DC_UP	189	0.34450245	15.846.552	0.0	0.002957752	0.983
GSE4748_CTRL_VS_LPS_STIM_DC_3H_UP	195	0.3374475	15.843.765	0.001335113	0.002966317	0.984
GSE39152_SPLEEN_CD103_NEG_VS_BRAIN_CD103_POS_MEMORY_CD8_TCELL_UP	196	0.34423637	15.839.905	0.0	0.002987394	0.986
GSE46606_IRF4HIGH_VS_IRF4MID_CD40L_IL2_IL5_DAY1_STIMULATED_BCELL_DN	190	0.34651187	15.835.654	0.0	0.003006083	0.988
GSE17974_IL4_AND_ANTI_IL12_VS_UNTREATED_4H_ACT_CD4_TCELL_UP	176	0.34633934	15.831.066	0.0	0.003016913	0.989
GSE40666_WT_VS_STAT4_KO_CD8_TCELL_UP	164	0.353029	15.831.383	0.002695417	0.003017725	0.989
GSE21927_EL4_VS_MCA203_TUMOR_MONOCYTES_UP	157	0.35030708	15.826.476	0.0	0.003033198	0.989
GSE11961_FOLLICULAR_BCELL_VS_MEMORY_BCELL_DAY7_UP	193	0.3400685	15.819.852	0.0	0.003051818	0.989
GSE37532_WT_VS_PPARG_KO_LN_TREG_DN	116	0.36228654	15.818.926	0.0	0.00305562	0.989
GSE34217_MIR17_92_OVEREXPRESS_VS_WT_ACT_CD8_TCELL_UP	130	0.35801315	1.581.736	0.002758620	0.003064835	0.989
GSE43955_10H_VS_60H_ACT_CD4_TCELL_WITH_TGFB_IL6_DN	196	0.33946717	1.581.625	0.002621232	0.003067076	0.989
GSE10239_NAIVE_VS_KLRG1INT_EFF_CD8_TCELL_UP	191	0.34263897	15.814.158	0.001340482	0.003075530	0.989
GSE45365_NK_CELL_VS_CD8_TCELL_MCMV_INFECTION_UP	194	0.34114242	1.580.914	0.0	0.003100906	0.989
GSE40273_EOS_KO_VS_WT_TREG_DN	190	0.34431887	15.808.694	0.0	0.003103115	0.989
GSE36078_UNTREATED_VS_AD5_INF_MOUSE_LUNG_DC_UP	193	0.34519488	15.806.487	0.0	0.003113774	0.989
GSE1432_CTRL_VS_IFNG_24H_MICROGLIA_UP	197	0.3404425	15.799.831	0.0	0.003145901	0.99
GSE3920_IFNA_VS_IFNB_TREATED_ENDOTHELIAL_CELL_UP	164	0.35138398	15.800.406	0.0	0.003146802	0.99
GSE15930_STIM_VS_STIM_AND_IL-12_48H_CD8_T_CELL_DN	198	0.34524783	15.797.294	0.0	0.003148820	0.99
GSE24142_DN2_VS_DN3_THYMOCYTE_ADULT_UP	194	0.34335655	15.794.028	0.0	0.003162499	0.99
GSE15930_STIM_VS_STIM_AND_TRICHOSTATINA_48H_CD8_T_CELL_UP	195	0.34048384	15.790.774	0.002677376	0.003172346	0.99
GSE24142_EARLY_THYMIC_PROGENITOR_VS_DN2_THYMOCYTE_ADULT_DN	195	0.34627804	15.788.662	0.001349527	0.003179882	0.99
GSE28449_WT_VS_LRF_KO_GERMINAL_CENTER_BCELL_UP	194	0.33956656	15.786.114	0.0	0.003188925	0.99
GSE22886_CD8_VS_CD4_NAIVE_TCELL_DN	181	0.34873077	15.784.817	0.0	0.003194104	0.99
GSE33292_WT_VS_TCF1_KO_DN3_THYMOCYTE_UP	193	0.34102398	15.783.433	0.001312336	0.003200823	0.99
GSE360_CTRL_VS_L_DONOVANI_DC_UP	194	0.3419422	15.780.892	0.001295336	0.003220565	0.99
GSE17721_PAM3CSK4_VS_GADIQUIMOD_1H_BMDM_UP	194	0.34362662	15.779.784	0.0	0.003224215	0.99
GSE22501_PERIPHERAL_BLOOD_VS_CORD_BLOOD_TREG_DN	193	0.34016457	15.772.896	0.001340482	0.003266089	0.991
GSE5679_CTRL_VS_PPARG_LIGAND_ROSIGLITAZONE_AND_RARA_AGONIST_AM580_TREATED_DC_UP	196	0.3375948	15.771.693	0.002607561	0.003267129	0.991
GSE9239_CTRL_VS_TNF_INHIBITOR_TREATED_DC_UP	189	0.34388322	15.771.902	0.001302083	0.003268136	0.991
GSE16385_ROSIGLITAZONE_VS_UNTREATED_IFNG_TNF_STIM_MACROPHAGE_UP	191	0.34123605	15.765.676	0.001336898	0.003292076	0.992
GSE46242_TH1_VS_ANERGIC_TH1_CD4_TCELL_WITH_EGR2_DELETED_DN	181	0.34433472	15.760.536	0.002614379	0.003314738	0.992
GSE3982_MAC_VS_TH2_UP	193	0.342497	15.760.177	0.0	0.003315219	0.992
GSE29949_CD8_NEG_DC_SPLEEN_VS_MONOCYTE_BONE_MARROW_DN	192	0.3407196	15.758.944	0.001290322	0.003322518	0.992
GSE19923_WT_VS_HEB_AND_E2A_KO_DP_THYMOCYTE_DN	197	0.33956683	1.575.263	0.0	0.003352393	0.993
GSE360_HIGH_DOSE_B_MALAYI_VS_M_TUBERCULOSIS_MAC_UP	196	0.3400883	1.575.325	0.001335113	0.003354217	0.993
GSE27859_MACROPHAGE_VS_DC_DN	180	0.34396276	15.748.478	0.003916449	0.003374880	0.993
GSE2770_TGFB_AND_IL4_ACT_VS_ACT_CD4_TCELL_48H_UP	190	0.34329855	15.747.035	0.0	0.003382898	0.993
GSE23925_DARK_ZONE_VS_NAIVE_BCELL_DN	190	0.33927464	15.738.373	0.002567394	0.003430328	0.993
GSE17721_ALL_VS_24H_PAM3CSK4_BMDM_DN	195	0.34064102	15.734.439	0.0	0.003454201	0.994
GSE46606_IRF4_KO_VS_WT_CD40L_IL2_IL5_3DAY_STIMULATED_BCELL_DN	176	0.34277073	15.729.634	0.001342281	0.003475050	0.994
GSE40277_GATA1_AND_SATB1_TRANSDUCED_VS_CTRL_CD4_TCELL_UP	192	0.34534287	15.716.683	0.002635046	0.003546407	0.995
GSE17812_WT_VS_THPOK_KO_MEMORY_CD8_TCELL_UP	193	0.33990866	15.713.702	0.0	0.003564882	0.995
GSE29949_MICROGLIA_BRAIN_VS_CD8_NEG_DC_SPLEEN_UP	191	0.3413825	15.713.546	0.002656042	0.003565162	0.995
GSE27859_CD11C_INT_F480_HI_MACROPHAGE_VS_CD11C_ING_F480_INT_DC_DN	197	0.33921948	15.699.208	0.0	0.003648512	0.995
GSE5589_WT_VS_IL6_KO_LPS_AND_IL6_STIM_MACROPHAGE_45MIN_DN	196	0.33990857	15.695.602	0.002624672	0.003665275	0.995
GSE17974_IL4_AND_ANTI_IL12_VS_UNTREATED_6H_ACT_CD4_TCELL_DN	182	0.3455515	15.694.985	0.0	0.003669252	0.995
GSE27896_HDAC6_KO_VS_WT_TREG_UP	164	0.34509024	15.693.644	0.0	0.003674728	0.995
GSE6269_HEALTHY_VS_STREP_PNEUMO_INF_PBMC_DN	168	0.3438416	15.689.175	0.002621232	0.003697526	0.995
GSE21360_NAIVE_VS_QUATERNARY_MEMORY_CD8_TCELL_DN	194	0.3407894	15.686.959	0.002699055	0.003709769	0.995
GSE360_L_DONOVANI_VS_T_GONDII_DC_UP	195	0.34035155	15.686.349	0.0	0.003712187	0.995
GSE23321_CENTRAL_MEMORY_VS_NAIVE_CD8_TCELL_UP	195	0.33686668	15.685.987	0.002635046	0.003713136	0.995
GSE42724_NAIVE_VS_MEMORY_BCELL_UP	191	0.3413594	1.568.375	0.001315789	0.003721573	0.995
GSE19401_UNSTIM_VS_RETINOIC_ACID_AND_PAM2CSK4_STIM_FOLLICULAR_DC_UP	199	0.3386506	15.677.438	0.0	0.003763093	0.995
GSE12003_MIR223_KO_VS_WT_BM_PROGENITOR_4D_CULTURE_UP	192	0.339817	15.675.604	0.0	0.003769964	0.995
GSE17721_POLYIC_VS_GARDIQUIMOD_24H_BMDM_UP	191	0.33601508	15.672.468	0.0	0.003788112	0.995
GSE22342_CD11C_HIGH_VS_LOW_DECIDUAL_MACROPHAGES_DN	176	0.34451142	156.713	0.001312336	0.003788974	0.995
GSE40666_NAIVE_VS_EFFECTOR_CD8_TCELL_DN	191	0.33978423	15.665.144	0.0	0.003824961	0.996
GSE10325_BCELL_VS_LUPUS_BCELL_DN	194	0.3408614	15.658.257	0.0	0.003857252	0.996
GSE17580_TREG_VS_TEFF_DN	196	0.33829597	15.655.484	0.0	0.003864892	0.996
GSE2706_UNSTIM_VS_8H_R848_DC_UP	178	0.34192836	15.656.141	0.001377410	0.003866284	0.996
GSE36009_WT_VS_NLRP10_KO_DC_UP	193	0.33665067	156.559	0.001373626	0.003866328	0.996
GSE2585_CTEC_VS_THYMIC_MACROPHAGE_UP	191	0.33682868	15.644.792	0.001275510	0.003913456	0.996
GSE42724_MEMORY_BCELL_VS_PLASMABLAST_UP	192	0.33888823	15.644.804	0.001248439	0.003916412	0.996
GSE13306_RA_VS_UNTREATED_TCONV_DN	195	0.33679056	15.643.584	0.0	0.003916491	0.996
GSE13946_CTRL_VS_DSS_COLITIS_GD_TCELL_FROM_COLON_DN	168	0.3395698	15.645.474	0.002695417	0.003917883	0.996
GSE46242_CTRL_VS_EGR2_DELETED_TH1_CD4_TCELL_DN	176	0.34333068	15.642.713	0.001351351	0.003920204	0.996
GSE17580_UNINFECTED_VS_S_MANSONI_INF_TEFF_UP	198	0.33637893	15.637.189	0.002645502	0.00395138	0.996
GSE26030_UNSTIM_VS_RESTIM_TH1_DAY5_POST_POLARIZATION_DN	192	0.33946875	15.632.718	0.001335113	0.003981760	0.996
GSE24142_ADULT_VS_FETAL_DN3_THYMOCYTE_DN	195	0.33779904	15.630.193	0.0	0.003995842	0.997
GSE32034_UNTREATED_VS_ROSIGLIZATONE_TREATED_LY6C_LOW_MONOCYTE_DN	180	0.33717936	15.620.286	0.001302083	0.004054915	0.997
GSE41867_NAIVE_VS_DAY8_LCMV_EFFECTOR_CD8_TCELL_DN	194	0.33599475	15.620.484	0.002597402	0.004057219	0.997
GSE24142_ADULT_VS_FETAL_DN3_THYMOCYTE_UP	193	0.3329433	15.618.063	0.0	0.004068089	0.997
GSE22432_MULTIPOTENT_VS_COMMON_DC_PROGENITOR_DN	191	0.3401789	15.615.429	0.0	0.004076109	0.997

GSE360_L_DONOVANI_VS_L_MAJOR_MAC_DN	192	0.3352325	15.610.193	0.0	0.004121845	0.997
GSE36392_TYPE_2_MYELOID_VS_MAC_IL25_TREATED_LUNG_UP	190	0.33779487	15.607.305	0.001300390	0.004134106	0.997
GSE2770_UNTREATED_VS_TGFB_AND_IL4_TREATED_ACT_CD4_TCELL_48H_UP	151	0.34523672	15.607.319	0.001360544	0.004137200	0.997
GSE37301_HEMATOPOIETIC_STEM_CELL_VS_GRAN_MONO_PROGENITOR_UP	151	0.3480813	15.604.222	0.004054054	0.004155290	0.997
GSE22886_NAIVE_TCELL_VS_NKCELL_DN	190	0.33960545	15.603.274	0.001322751	0.004158049	0.997
GSE36078_WT_VS_IL1R_KO_LUNG_DC_UP	192	0.33692503	15.601.245	0.001356852	0.004168196	0.997
GSE6674_ANTI_IGM_VS_CPG_STIM_BCELL_UP	188	0.33808511	15.596.642	0.001321004	0.004188466	0.997
GSE22935_24H_VS_48H_MBOVIS_BCG_STIM_MACROPHAGE_UP	190	0.33869803	15.596.839	0.001342281	0.004191589	0.997
GSE26928_CENTR_MEMORY_VS_CXCR5_POS_CD4_TCELL_UP	174	0.34234992	15.593.101	0.001329787	0.004209552	0.997
GSE17974_CTRL_VS_ACT_IL4_AND_ANTI_IL12_0.5H_CD4_TCELL_UP	155	0.3457776	1.558.723	0.001375515	0.004239499	0.997
GSE21927_UNTREATED_VS_GMCSF_IL6_TREATED_BONE_MARROW_DN	167	0.3422662	15.574.414	0.005305039	0.004328803	0.998
GSE17301_IFNA2_VS_IFNA5_STIM_ACD3_ACD28_ACT_CD8_TCELL_UP	190	0.33643758	15.572.208	0.0	0.004338465	0.998
GSE8685_IL2_ACT_IL2_STARVED_VS_IL21_ACT_IL2_STARVED_CD4_TCELL_DN	137	0.34830588	15.572.623	0.0	0.004339501	0.998
GSE37605_FOXP3_FUSION_GFP_VS_IRES_GFP_TREG_C57BL6_UP	181	0.3386235	1.557.044	0.0	0.004349149	0.998
GSE25085_FETAL_BM_VS_ADULT_BM_SP4_THYMIC_IMPLANT_DN	190	0.34069088	15.567.172	0.002590673	0.004364167	0.998
GSE6259_FLT3L_INDUCED_DEC205_POS_DC_VS_BCELL_UP	146	0.3498688	15.555.258	0.002762431	0.004444048	0.998
GSE411_100MIN_VS_400MIN_IL6_STIM_SOCS3_KO_MACROPHAGE_DN	187	0.33765984	15.553.545	0.001319261	0.004453176	0.998
GSE29949_MICROGLIA_BRAIN_VS_CD8_POS_DC_SPLEEN_DN	194	0.33374465	15.548.772	0.001275510	0.004484721	0.998
GSE17974_IL4_AND_ANTI_IL12_VS_UNTREATED_6H_ACT_CD4_TCELL_UP	166	0.34622177	15.548.828	0.001347708	0.004488035	0.998
GSE13522_CTRL_VS_T_CRUZI_G_STRAIN_INF_SKIN_DN	127	0.35507566	15.548.882	0.001377410	0.004490624	0.998
GSE13411_NAIVE_VS_MEMORY_BCELL_UP	184	0.34177387	15.545.552	0.001371742	0.004497713	0.998
GSE2770_TGFB_AND_IL4_VS_IL4_TREATED_ACT_CD4_TCELL_2H_UP	189	0.33795372	15.545.808	0.0	0.004498137	0.998
GSE1460_CD4_THYMOCYTE_VS_NAIVE_CD4_TCELL_ADULT_BLOOD_DN	193	0.3338163	15.544.679	0.001317523	0.004498755	0.998
GSE39556_UNTREATED_VS_3H_POLYIC_INJ_MOUSE_NK_CELL_UP	197	0.33486044	1.553.814	0.0	0.004540433	0.998
GSE2585_CTEC_VS_THYMIC_DC_DN	195	0.33770105	15.526.891	0.0	0.004615566	0.998
GSE12845_NAIVE_VS_DARKZONE_GC_TONSIL_BCELL_UP	191	0.33509213	15.527.095	0.001338688	0.004616804	0.998
GSE17721_LPS_VS_POLYIC_2H_BMDM_UP	198	0.33740962	1.552.219	0.001290322	0.004644971	0.998
GSE32034_LY6C_HIGH_VS_LOW_ROSIGLIZATONE_TREATED_MONOCYTE_UP	197	0.33429846	15.522.408	0.0	0.004648383	0.998
GSE40441_NRP1_POS_INDUCED_TREG_VS_NRP1_NEG_NATURAL_TREG_UP	195	0.33289632	15.518.157	0.001329787	0.004672683	0.998
GSE17974_IL4_AND_ANTI_IL12_VS_UNTREATED_2H_ACT_CD4_TCELL_DN	164	0.3467326	15.516.125	0.002728513	0.004686623	0.998
GSE411_WT_VS_SOCS3_KO_MACROPHAGE_IL6_STIM_400MIN_UP	190	0.33836353	15.505.155	0.0	0.004766128	0.998
GSE17721_CTRL_VS_CPG_2H_BMDM_DN	192	0.33418867	15.498.888	0.001342281	0.004795271	0.999
GSE11057_CD4_EFF_MEM_VS_PBMC_UP	190	0.3357006	15.499.333	0.0	0.004796612	0.999
GSE6259_BCELL_VS_CD8_TCELL_UP	179	0.34208	15.497.301	0.003926701	0.00480255	0.999
GSE41176_UNSTIM_VS_ANTI_IGM_STIM_BCELL_3H_DN	190	0.33668894	1.549.365	0.001317523	0.004829939	0.999
GSE32423_MEMORY_VS_NAIVE_CD8_TCELL_UP	195	0.33967847	15.491.004	0.0	0.004845890	0.999
GSE3982_MAC_VS_NKCELL_UP	195	0.33284542	15.490.136	0.003994674	0.004848819	0.999
GSE7460_FOXP3_MUT_VS_WT_ACT_WITH_TGFB_TCONV_DN	194	0.338449	15.489.883	0.001340482	0.004851009	0.999
GSE21927_SPLEEN_MONOCYTE_VS_GMCSF_GCSF_BONE_MARROW_DN	185	0.3386532	15.486.584	0.004016064	0.004873276	0.999
GSE5589_LPS_VS_LPS_AND_IL10_STIM_IL6_KO_MACROPHAGE_45MIN_DN	193	0.33420518	15.485.072	0.002724795	0.004878359	0.999
GSE17721_ALL_VS_24H_PAM3CSK4_BMDM_UP	192	0.3334293	15.484.521	0.002607561	0.004881255	0.999
GSE360_L_MAJOR_VS_B_MALAYI_LOW_DOSE_DC_UP	197	0.33663023	154.833	0.002607561	0.004886293	0.999
GSE36888_UNTREATED_VS_IL2_TREATED_TCELL_2H_UP	183	0.3382634	15.483.111	0.0	0.004886334	0.999
GSE36888_UNTREATED_VS_IL2_TREATED_TCELL_17H_DN	184	0.3355729	15.482.072	0.004	0.004889189	0.999
GSE43955_TH0_VS_TGFB_IL6_IL23_TH17_ACT_CD4_TCELL_60H_UP	199	0.33528394	15.477.303	0.002652519	0.004914969	0.999
GSE2585_THYMIC_MACROPHAGE_VS_MTEC_UP	188	0.33590364	15.477.479	0.002600780	0.004918534	0.999
GSE11057_NAIVE_VS_CENT_MEMORY_CD4_TCELL_UP	168	0.34259868	15.475.891	0.002732240	0.004918537	0.999
GSE360_L_MAJOR_VS_M_TUBERCULOSIS_DC_DN	193	0.33521202	15.476.264	0.003942181	0.004919236	0.999
GSE7831_UNSTIM_VS_INFLUENZA_STIM_PDC_1H_UP	189	0.33387116	15.464.274	0.0	0.005017637	1.0
GSE16385_IFNG_TNF_VS_IL4_STIM_MACROPHAGE_ROSIGLITAZONE_TREATED_UP	169	0.34078264	154.618	0.004048583	0.005032535	1.0
GSE37301_COMMON_LYMPHOID_PROGENITOR_VS_RAG2_KO_NK_CELL_UP	190	0.33365145	15.458.487	0.001331557	0.00505024	1.0
GSE9988_ANTI_TREM1_VS_LOW_LPS_MONOCYTE_UP	186	0.3344644	15.456.505	0.001324503	0.005065138	1.0
GSE24574_BCL6_HIGH_TFH_VS_TFH_CD4_TCELL_UP	188	0.34063554	15.454.212	0.002688172	0.005080014	1.0
GSE26030_UNSTIM_VS_RESTIM_TH17_DAY5_POST_POLARIZATION_UP	173	0.33919656	15.449.128	0.004092769	0.005114673	1.0
GSE9988_LOW_LPS_VS_CTRL_TREATED_MONOCYTE_DN	191	0.3314942	1.543.967	0.0	0.005188192	1.0
GSE26030_TH1_VS_TH17_RESTIMULATED_DAY15_POST_POLARIZATION_UP	195	0.33329678	15.440.077	0.0	0.005188342	1.0
GSE23502_WT_VS_HDC_KO_MYELOID_DERIVED_SUPPRESSOR_CELL_BM_DN	192	0.33260673	15.429.358	0.001335113	0.005259577	1.0
GSE11961_MARGINAL_ZONE_BCELL_VS_MEMORY_BCELL_DAY40_UP	193	0.3293065	15.425.723	0.0	0.005281480	1.0
GSE2706_2H_VS_8H_R848_AND_LPS_STIM_DC_DN	187	0.33355403	15.426.052	0.001288659	0.005281753	1.0
GSE29164_DAY3_VS_DAY7_CD8_TCELL_TREATED_MELANOMA_UP	194	0.33132514	1.542.663	0.001340482	0.005282012	1.0
GSE8515_IL1_VS_IL6_4H_STIM_MAC_UP	196	0.3321444	15.421.966	0.001351351	0.005305984	1.0
GSE9960_GRAM_NEG_VS_GRAM_NEG_AND_POS_SEPSIS_PBMC_UP	166	0.3399509	15.420.256	0.002635046	0.005311105	1.0
GSE25088_ROSIGLITAZONE_VS_IL4_AND_ROSIGLITAZONE_STIM_STAT6_KO_MACROPHAGE_DAY10_UP	187	0.33376288	15.420.388	0.002699055	0.005313497	1.0
GSE6269_E_COLI_VS_STREP_PNEUMO_INF_PBMC_DN	160	0.34094056	15.414.656	0.0	0.005346783	1.0
GSE26343_WT_VS_NFAT5_KO_MACROPHAGE_LPS_STIM_UP	196	0.3306805	15.402.694	0.001272264	0.005431690	1.0
GSE40274_FOXP3_VS_FOXP3_AND_GATA1_TRANSDUCED_ACTIVATED_CD4_TCELL_DN	192	0.3352203	1.540.007	0.002594033	0.005448956	1.0
GSE24142_DN2_VS_DN3_THYMOCYTE_FETAL_UP	198	0.33371806	15.396.926	0.002666666	0.005482373	1.0
GSE37301_PRO_BCELL_VS_RAG2_KO_NK_CELL_DN	176	0.33953547	15.393.994	0.006622516	0.005505872	1.0
GSE4984_GALECTIN1_VS_VEHICLE_CTRL_TREATED_DC_UP	176	0.336706	15.393.682	0.0	0.005506157	1.0
GSE22886_IGA_VS_IGM_MEMORY_BCELL_UP	192	0.33446413	15.393.077	0.001319261	0.005507839	1.0
GSE3982_BASOPHIL_VS_NKCELL_UP	187	0.33174473	15.382.366	0.001326259	0.005601464	1.0
GSE11961_MEMORY_BCELL_DAY7_VS_GERMINAL_CENTER_BCELL_DAY7_UP	192	0.3325967	15.379.881	0.0	0.005616351	1.0
GSE43955_10H_VS_30H_ACT_CD4_TCELL_WITH_TGFB_IL6_DN	194	0.33308733	15.376.883	0.001287001	0.005646669	1.0
GSE26156_DOUBLE_POSITIVE_VS_CD4_SINGLE_POSITIVE_THYMOCYTE_UP	192	0.33534172	1.536.805	0.001308900	0.005730920	1.0
GSE20366_EX_VIVO_VS_DEC205_CONVERSION_NAIVE_CD4_TCELL_DN	185	0.33723602	1.536.761	0.002624672	0.005731045	1.0
GSE28408_LY6G_POS_VS_NEG_DC_UP	190	0.3345433	15.365.211	0.001307189	0.005751449	1.0
GSE26030_UNSTIM_VS_RESTIM_TH1_DAY15_POST_POLARIZATION_UP	197	0.33538538	15.362.422	0.002652519	0.005770463	1.0
GSE360_DC_VS_MAC_L_DONOVANI_DN	196	0.3305278	15.360.442	0.003901170	0.005776213	1.0
GSE17721_POLYIC_VS_GARDIQUIMOD_16H_BMDM_UP	193	0.3342007	15.360.938	0.002635046	0.005778193	1.0
GOLDRATH_NAIVE_VS_EFF_CD8_TCELL_DN	197	0.33273026	1.535.959	0.001321004	0.005779782	1.0
GSE29164_UNTREATED_VS_CD8_TCELL_AND_IL12_TREATED_MELANOMA_DAY7_DN	189	0.33350924	153.557	0.002621232	0.005813324	1.0
GSE9650_GP33_VS_GP276_LCMV_SPECIFIC_EXHAUSTED_CD8_TCELL_UP	198	0.33187518	15.354.196	0.0	0.005818985	1.0
GSE40274_FOXP3_VS_FOXP3_AND_XBP1_TRANSDUCED_ACTIVATED_CD4_TCELL_DN	150	0.34138522	15.345.036	0.004037685	0.005905888	1.0
GSE16385_UNTREATED_VS_12H_ROSIGLITAZONE_TREATED_MACROPHAGE_UP	158	0.34064463	15.339.702	0.001349527	0.005952455	1.0
GSE25088_IL4_VS_IL4_AND_ROSIGLITAZONE_STIM_MACROPHAGE_DAY10_UP	195	0.3315435	15.338.277	0.0	0.005959379	1.0
GSE46606_DAY1_VS_DAY3_CD40L_IL2_IL5_STIMULATED_IRF4MID_BCELL_UP	191	0.33574754	15.333.064	0.001290322	0.006002326	1.0
GSE21063_3H_VS_16H_ANTI_IGM_STIM_NFATC1_KOBCELL_UP	194	0.3320593	1.533.245	0.001328021	0.006004369	1.0
GSE360_HIGH_VS_LOW_DOSE_B_MALAYI_MAC_DN	197	0.3320058	15.329.262	0.002580645	0.006025847	1.0
GSE2128_C57BL6_VS_NOD_THYMOCYTE_MIMETOPE_NEGATIVE_SELECTION_DN	190	0.32948816	1.532.833	0.003968254	0.006029929	1.0

GSE5503_MLN_DC_VS_PLN_DC_ACTIVATED_ALLOGENIC_TCELL_DN		191	0.33113843	15.327.015	0.0	0.006035383	1.0
GSE4590_SMALL_VS_VPREB_POS_LARGE_PRE_BCELL_DN		151	0.3454603	15.323.462	0.002781641	0.006064394	1.0
GSE43955_1H_VS_42H_ACT_CD4_TCELL_UP		196	0.33153713	15.319.806	0.005167959	0.006087072	1.0
GSE3720_VD1_VS_VD2_GAMMADELTA_TCELL_WITH_LPS_STIM_DN		192	0.33350873	1.530.683	0.005464480	0.006196819	1.0
GSE18281_SUBCAPSULAR_CORTICAL_REGION_VS_WHOLE_MEDULLA_THYMUS_DN		186	0.33250502	15.298.755	0.003984064	0.006277991	1.0
GSE36078_UNTREATED_VS_AD5_T425A_HEXON_INF_MOUSE_LUNG_DC_DN		195	0.32949615	15.295.873	0.001280409	0.006301202	1.0
GSE3982_NEUTROPHIL_VS_NKCELL_UP		198	0.3299077	15.292.002	0.001322751	0.006336095	1.0
GSE7852_TREG_VS_TCONV_FAT_UP		195	0.32986426	15.287.898	0.0	0.006372346	1.0
GSE6566_STRONG_VS_WEAK_DC_STIMULATED_CD4_TCELL_DN		174	0.33646983	15.287.315	0.002680965	0.006374096	1.0
GSE2585_CTEC_VS_MTEC_THYMUS_UP		187	0.33522758	15.285.364	0.0	0.006393712	1.0
GSE32986_GMCSF_AND_CURDLAN_LOWDOSE_VS_GMCSF_AND_CURDLAN_HIGHDOSE_STIM_DC_DN		177	0.33403277	15.284.827	0.001336898	0.006394756	1.0
GSE2770_UNTREATED_VS_ACT_CD4_TCELL_2H_UP		191	0.3303455	1.528.275	0.003865979	0.006408177	1.0
GSE26030_UNSTIM_VS_RESTIM_TH1_DAY5_POST_POLARIZATION_UP		191	0.3363581	15.281.864	0.001282051	0.006417411	1.0
GSE32034_LY6C_HIGH_VS_LOW_ROSIGLIZATONE_TREATED_MONOCYTE_DN		197	0.33095017	15.274.333	0.001300390	0.006489776	1.0
GSE3982_NKCELL_VS_TH1_UP		183	0.33251163	15.273.271	0.002663115	0.006500305	1.0
GSE27859_CD11C_INT_F480_HI_MACROPHAGE_VS_CD11C_ING_F480_INT_DC_UP		172	0.33199883	1.527.269	0.002557544	0.006503327	1.0
GSE16385_ROSIGLITAZONE_IL4_VS_IL4_ALONE_STIM_MACROPHAGE_12H_UP		191	0.33276108	15.268.255	0.001273885	0.006541225	1.0
GSE8921_UNSTIM_0H_VS_TLR1_2_STIM_MONOCYTE_12H_DN		193	0.32880664	15.260.676	0.0	0.006605814	1.0
GSE43955_1H_VS_60H_ACT_CD4_TCELL_WITH_TGFB_IL6_UP		196	0.3290135	15.259.705	0.002534854	0.006612165	1.0
GSE28726_NAIVE_VS_ACTIVATED_VA24NEG_NKTCELL_DN		191	0.32738006	15.244.293	0.004137931	0.006763302	1.0
GSE8621_UNSTIM_VS_LPS_STIM_MACROPHAGE_DN		191	0.33095014	1.523.323	0.001288659	0.006861063	1.0
GSE37416_0H_VS_6H_F_TULARENSIS_LVS_NEUTROPHIL_UP		192	0.32707727	15.230.719	0.002628120	0.00689041	1.0
GSE21927_SPLEEN_VS_C26GM_TUMOR_MONOCYTE_BALBC_UP		157	0.33638126	15.226.166	0.003963012	0.006946942	1.0
GSE7831_UNSTIM_VS_CPG_STIM_PDC_4H_DN		195	0.33187833	15.224.795	0.001340482	0.006952352	1.0
GSE21927_SPLENIC_VS_TUMOR_MONOCYTES_FROM_C26GM_TUMOROUS_MICE_BALBC_UP		171	0.33033404	15.218.428	0.001369863	0.007020307	1.0
GSE24814_STAT5_KO_VS_WT_PRE_BCELL_UP		190	0.32990807	15.216.633	0.0	0.007034478	1.0
GSE1112_OT1_VS_HY_CD8AB_THYMOCYTE_RTOC_CULTURE_UP		191	0.3309805	15.215.281	0.0	0.007036892	1.0
GSE22432_UNTREATED_VS_TGFB1_TREATED_COMMON_DC_PROGENITOR_UP		190	0.3319356	15.215.306	0.002594033	0.007041742	1.0
GSE13173_UNTREATED_VS_IL12_TREATED_ACT_CD8_TCELL_UP		119	0.3540054	15.215.354	0.007112375	0.007045222	1.0
GSE22601_DOUBLE_NEGATIVE_VS_IMMATURE_CD4_SP_THYMOCYTE_DN		175	0.3334017	15.209.498	0.002677376	0.007102675	1.0
GSE7852_TREG_VS_TCONV_UP		193	0.33098507	15.208.429	0.001319261	0.007106588	1.0
GSE31082_DN_VS_CD4_SP_THYMOCYTE_DN		193	0.33065864	15.204.794	0.003870967	0.007148415	1.0
GSE40274_FOXP3_VS_FOXP3_AND_SATB1_TRANSDUCED_ACTIVATED_CD4_TCELL_DN		168	0.3369773	15.202.596	0.006963788	0.007163661	1.0
GSE5589_LPS_AND_IL10_VS_LPS_AND_IL6_STIM_IL10_KO_MACROPHAGE_45MIN_UP		194	0.33027083	15.202.628	0.003821656	0.007167235	1.0
GSE3982_BASOPHIL_VS_EFF_MEMORY_CD4_TCELL_UP		194	0.33165675	15.200.127	0.0	0.007189188	1.0
GSE21927_SPLEEN_VS_TUMOR_MONOCYTE_C57BL6_UP		165	0.33317092	1.519.917	0.001328021	0.007191690	1.0
GSE27786_CD4_VS_CD8_TCELL_UP		194	0.32530522	15.195.014	0.003886010	0.007238474	1.0
GSE32901_TH1_VS_TH17_ENRICHED_CD4_TCELL_UP		171	0.3337987	15.195.081	0.006858710	0.007242763	1.0
GSE40274_CTRL_VS_FOXP3_AND_SATB1_TRANSDUCED_ACTIVATED_CD4_TCELL_DN		144	0.34269956	1.519.059	0.004322767	0.007278068	1.0
GSE39820_CTRL_VS_IL1B_IL6_IL23A_CD4_TCELL_UP		198	0.32801804	1.518.715	0.001356852	0.007305462	1.0
GSE17974_0H_VS_0.5H_IN_VITRO_ACT_CD4_TCELL_UP		174	0.3302581	15.186.324	0.0	0.007308532	1.0
GSE19941_IL10_KO_VS_IL10_KO_AND_NFKBP50_KO_UNSTIM_MACROPHAGE_DN		196	0.32925013	15.185.442	0.002642008	0.007316984	1.0
GSE22886_NAIVE_TCELL_VS_NKCELL_UP		190	0.3316195	15.185.055	0.003921569	0.007317367	1.0
GSE15767_MED_VS_SCS_MAC_LN_DN		191	0.3277508	15.183.307	0.001298701	0.007329195	1.0
GSE37301_HEMATOPOIETIC_STEM_CELL_VS_PRO_BCELL_UP		189	0.32679486	15.181.739	0.0	0.007344352	1.0
GSE24671_CTRL_VS_BAKIMULC_INFECTED_MOUSE_SPLENOCYTES_UP		194	0.32932648	15.170.068	0.001360544	0.007473467	1.0
GSE25088_WT_VS_STAT6_KO_MACROPHAGE_ROSIGLITAZONE_STIM_DN		188	0.32749373	15.166.963	0.002567394	0.007502554	1.0
GSE36826_WT_VS_IL1R_KO_SKIN_DN		194	0.3285826	15.165.609	0.002645502	0.007513521	1.0
GSE13411_IGM_MEMORY_BCELL_VS_PLASMA_CELL_UP		187	0.32838023	15.164.515	0.005228758	0.007519839	1.0
GSE17721_POLYIC_VS_GARDIQUIMOD_8H_BMDM_UP		195	0.3289361	15.162.647	0.005115089	0.007528302	1.0
GSE2585_THYMIC_DC_VS_MTEC_DN		196	0.32548097	15.163.116	0.005340454	0.007529406	1.0
GSE11864_CSF1_VS_CSF1_PAM3CYS_IN_MAC_UP		184	0.32884187	15.151.182	0.001315789	0.007653446	1.0
GSE3982_EOSINOPHIL_VS_BCELL_UP		197	0.32480913	15.146.117	0.0	0.007706316	1.0
GSE2770_IL12_AND_TGFB_ACT_VS_ACT_CD4_TCELL_48H_UP		184	0.3337853	15.121.742	0.001338688	0.00795489	1.0
GSE25088_WT_VS_STAT6_KO_MACROPHAGE_IL4_STIM_UP		190	0.32783458	15.123.544	0.001319261	0.00795509	1.0
GSE12845_IGD_POS_BLOOD_VS_DARKZONE_GC_TONSIL_BCELL_UP		190	0.3270234	15.122.226	0.005277045	0.007955603	1.0
GSE37336_LY6C_POS_VS_NEG_NAIVE_CD4_TCELL_DN		152	0.33822948	15.123.084	0.002785515	0.007956385	1.0
GSE13306_TREG_VS_TCONV_LAMINA_PROPRIA_DN		186	0.33073264	15.122.241	0.002631579	0.007960983	1.0
GSE11961_MARGINAL_ZONE_BCELL_VS_GERMINAL_CENTER_BCELL_DAY7_DN		190	0.32822162	15.120.437	0.001326259	0.007964131	1.0
GSE25123_CTRL_VS_ROSIGLITAZONE_STIM_PPARG_KO_MACROPHAGE_DN		192	0.32533145	15.119.406	0.0	0.007975392	1.0
GSE19512_NAUTRAL_VS_INDUCED_TREG_UP		191	0.32934693	15.115.414	0.002659574	0.008017186	1.0
GSE19825_NAIVE_VS_IL2RALOW_DAY3_EFF_CD8_TCELL_UP		195	0.32706234	15.107.317	0.002673796	0.008119336	1.0
GSE14350_IL2RB_KO_VS_WT_TEFF_UP		192	0.32523462	15.105.108	0.002628120	0.008129621	1.0
GSE21546_WT_VS_SAP1A_KO_AND_ELK1_KO_ANTI_CD3_STIM_DP_THYMOCYTES_DN		190	0.3298339	15.105.559	0.002580645	0.008131784	1.0
GSE23502_WT_VS_HDC_KO_MYELOID_DERIVED_SUPPRESSOR_CELL_COLON_TUMOR_DN		189	0.32719794	15.102.885	0.002594033	0.008145885	1.0
GSE3982_BCELL_VS_BASOPHIL_DN		194	0.324594	15.102.236	0.005181347	0.008149648	1.0
GSE12484_HEALTHY_VS_PERIDONTITIS_NEUTROPHILS_UP		130	0.3429854	15.103.035	0.006887052	0.008150694	1.0
GSE17974_0H_VS_0.5H_IN_VITRO_ACT_CD4_TCELL_DN		166	0.33395988	15.100.186	0.004081632	0.008169332	1.0
GSE6269_E_COLI_VS_STAPH_AUREUS_INF_PBMC_DN		180	0.33033714	1.509.958	0.002677376	0.008171101	1.0
GSE1460_DP_THYMOCYTE_VS_NAIVE_CD4_TCELL_ADULT_BLOOD_DN		191	0.3266917	15.094.583	0.002638522	0.008221066	1.0
GSE3994_WT_VS_PAC1_KO_ACTIVATED_MAST_CELL_DN		196	0.32986227	15.092.952	0.001295336	0.008230725	1.0
GSE17721_CPG_VS_GARDIQUIMOD_16H_BMDM_UP		194	0.32730275	15.091.457	0.001338688	0.008239702	1.0
GSE32986_GMCSF_VS_GMCSF_AND_CURDLAN_HIGHDOSE_STIM_DC_DN		179	0.32892174	15.090.119	0.001336898	0.008249302	1.0
GSE21033_1H_VS_12H_POLYIC_STIM_DC_UP		170	0.3333673	15.086.823	0.009186352	0.008291845	1.0
GSE20366_TREG_VS_NAIVE_CD4_TCELL_DEC205_CONVERSION_DN		192	0.32328612	15.086.241	0.003921569	0.008293509	1.0
GSE27786_LSK_VS_CD4_TCELL_DN		190	0.3284649	15.082.158	0.002666666	0.008339274	1.0
GSE17721_PAM3CSK4_VS_GADIQUIMOD_2H_BMDM_UP		197	0.32497373	15.073.287	0.001288659	0.008442041	1.0
GSE36888_STAT5_AB_KNOCKIN_VS_WT_TCELL_IL2_TREATED_6H_UP		187	0.3243831	1.507.112	0.001412429	0.00846066	1.0
GSE21360_PRIMARY_VS_QUATERNARY_MEMORY_CD8_TCELL_DN		194	0.32682863	15.070.153	0.004070556	0.008468806	1.0
GSE37533_PPARG1_FOXP3_VS_PPARG2_FOXP3_TRANSDUCED_CD4_TCELL_DN		180	0.32702094	15.058.539	0.004081632	0.008612639	1.0
GSE46606_UNSTIM_VS_CD40L_IL2_IL5_3DAY_STIMULATED_IRF4MID_SORTED_BCELL_DN		191	0.32431734	15.052.742	0.003994674	0.008687410	1.0
GSE15324_NAIVE_VS_ACTIVATED_CD8_TCELL_UP		190	0.33129388	15.051.662	0.001319261	0.008699342	1.0
GSE40666_NAIVE_VS_EFFECTOR_CD8_TCELL_UP		194	0.32509965	15.033.286	0.007712082	0.008906282	1.0
GSE12366_PLASMA_CELL_VS_MEMORY_BCELL_DN		190	0.32518423	1.502.364	0.003870967	0.009040334	1.0
GSE26559_TCF1_KO_VS_WT_LIN_NEG_CELL_DN		185	0.3271581	15.016.934	0.002652519	0.009123757	1.0
GSE31082_CD4_VS_CD8_SP_THYMOCYTE_UP		191	0.32285917	15.015.975	0.00660502	0.009128189	1.0
GSE24210_TCONV_VS_TREG_UP		190	0.32324567	15.014.024	0.003968254	0.009142358	1.0
GSE20366_TREG_VS_TCONV_UP		195	0.32545125	15.008.674	0.001322751	0.009203507	1.0
GSE21546_UNSTIM_VS_ANTI_CD3_STIM_SAP1A_KO_AND_ELK1_KO_DP_THYMOCYTES_DN		154	0.33536533	14.999.701	0.008241759	0.009322562	1.0

GSE13229_MATURE_VS_INTMATURE_NKCELL_UP	187	0.32815784	14.997.151	0.001333333	0.009348276	1.0
GSE11961_GERMINAL_CENTER_BCELL_DAY7_VS_PLASMA_CELL_DAY7_UP	196	0.3228162	14.990.437	0.003952569	0.00943522	1.0
GSE21379_WT_VS_SAP_KO_CD4_TCELL_DN	192	0.32839438	14.987.606	0.006640106	0.009470576	1.0
GSE2405_0H_VS_24H_A_PHAGOCYTOPHILUM_STIM_NEUTROPHIL_DN	192	0.32754177	14.983.649	0.002673796	0.009531284	1.0
GSE15750_DAY6_VS_DAY10_TRAF6KO_EFF_CD8_TCELL_DN	186	0.3273603	14.982.451	0.004065040	0.009541222	1.0
GSE21670_IL6_VS_TGFB_AND_IL6_TREATED_STAT3_KO_CD4_TCELL_DN	191	0.32447776	14.980.295	0.002673796	0.009561548	1.0
GSE12845_IGD_POS_BLOOD_VS_NAIVE_TONSIL_BCELL_UP	186	0.32412407	14.974.233	0.002587322	0.009628084	1.0
GSE43863_TH1_VS_LY6C_INT_CXCR5POS_MEMORY_CD4_TCELL_DN	193	0.32465342	14.974.499	0.002677376	0.009631822	1.0
GSE27859_MACROPHAGE_VS_DC_UP	162	0.33082452	14.970.815	0.006811989	0.009675555	1.0
GSE37533_UNTREATED_VS_PIOGLIZATONE_TREATED_CD4_TCELL_PPARG1_AND_FOXP3_TRASDUCED_UP	196	0.32106107	14.970.176	0.003886010	0.009680195	1.0
GSE36888_STAT5_AB_KNOCKIN_VS_WT_TCELL_IL2_TREATED_17H_UP	196	0.32283816	14.964.696	0.002554278	0.009756672	1.0
GSE18281_MEDULLARY_THYMOCYTE_VS_WHOLE_MEDULLA_THYMUS_UP	181	0.32776716	14.963.561	0.002554278	0.009758117	1.0
GSE22601_IMMATURE_CD4_SINGLE_POSITIVE_VS_CD4_SINGLE_POSITIVE_THYMOCYTE_UP	195	0.32331166	14.964.051	0.003886010	0.009758705	1.0
GSE13522_WT_VS_IFNG_KO_SKIN_DN	120	0.349563	1.496.254	0.008571428	0.009764623	1.0
GSE9601_UNTREATED_VS_NFKB_INHIBITOR_TREATED_HCMV_INF_MONOCYTE_UP	157	0.33457536	14.961.715	0.004037685	0.009768586	1.0
GSE22025_UNTREATED_VS_TGFB1_TREATED_CD4_TCELL_DN	192	0.32201335	14.960.495	0.007822686	0.009786719	1.0
GSE7348_UNSTIM_VS_TOLERIZED_AND_LPS_STIM_MACROPHAGE_DN	156	0.3317648	14.953.946	0.006896551	0.009870587	1.0
GSE15659_TREG_VS_TCONV_DN	161	0.33168247	14.953.197	0.002624672	0.009876368	1.0
GSE13484_12H_VS_3H_YF17D_VACCINE_STIM_PBMC_DN	194	0.32308963	14.950.715	0.001329787	0.009901439	1.0
GSE44649_WT_VS_MIR155_KO_NAIVE_CD8_TCELL_UP	193	0.32532176	14.950.901	0.001364256	0.009904053	1.0
GSE25087_TREG_VS_TCONV_FETUS_UP	187	0.32542092	14.949.613	0.00405954	0.009909724	1.0
GSE17322_CD103_POS_VS_CD11B_HIGH_LUNG_DC_UP	188	0.32584843	14.936.364	0.006693440	0.010093325	1.0
GSE7768_OVA_ALONE_VS_OVA_WITH_MPL_IMMUNIZED_MOUSE_WHOLE_SPLEEN_6H_DN	160	0.33209756	14.935.194	0.004092769	0.010102189	1.0
GSE11864_UNTREATED_VS_CSF1_IN_MAC_UP	187	0.324375	14.933.677	0.006693440	0.010125105	1.0
GSE2826_WT_VS_BTK_KO_BCELL_DN	190	0.32371283	14.928.899	0.001321004	0.010186451	1.0
GSE6259_33D1_POS_VS_DEC205_POS_SPLENIC_DC_UP	174	0.33115932	14.919.347	0.003911343	0.010325922	1.0
GSE34205_HEALTHY_VS_FLU_INF_INFANT_PBMC_DN	190	0.32362488	14.917.339	0.005235602	0.010350561	1.0
GSE6259_DEC205_POS_DC_VS_BCELL_DN	164	0.33199468	14.915.239	0.005457026	0.010376478	1.0
GSE3982_MEMORY_CD4_TCELL_VS_TH1_UP	189	0.32373866	14.906.405	0.007905139	0.010492146	1.0
GSE1566_WT_VS_EZH2_KO_LN_TCELL_UP	171	0.32900763	14.906.607	0.005361930	0.010495757	1.0
GSE4748_CTRL_VS_LPS_AND_CYANOBACTERIUM_LPSLIKE_STIM_DC_3H_UP	191	0.32525453	1.490.432	0.001322751	0.010516020	1.0
GSE17721_CTRL_VS_GARDIQUIMOD_8H_BMDM_DN	193	0.32509306	14.902.346	0.003811944	0.010534076	1.0
GSE9650_EFFECTOR_VS_EXHAUSTED_CD8_TCELL_UP	194	0.32000208	14.896.404	0.00390625	0.010624883	1.0
GSE33292_DN3_THYMOCYTE_VS_TCF1_KO_TCELL_LYMPHOMA_DN	140	0.33618286	14.890.372	0.007002801	0.010702101	1.0
GSE360_DC_VS_MAC_L_DONOVANI_UP	194	0.32263646	14.889.935	0.001317523	0.01070348	1.0
GSE13547_WT_VS_ZFX_KO_BCELL_DN	171	0.32904118	14.885.553	0.002680965	0.010763443	1.0
GSE17721_12H_VS_24H_PAM3CSK4_BMDM_DN	194	0.32092604	14.883.426	0.004048583	0.01078199	1.0
GSE29949_CD8_NEG_DC_SPLEEN_VS_CD8_POS_DC_SPLEEN_UP	195	0.32489935	1.488.059	0.002607561	0.01082719	1.0
GSE40225_WT_VS_RIP_B7X_DIABETIC_MOUSE_PANCREATIC_CD8_TCELL_UP	183	0.326089	14.876.082	0.005361930	0.010901524	1.0
GSE14769_UNSTIM_VS_240MIN_LPS_BMDM_DN	197	0.32078668	14.872.421	0.001287001	0.010959357	1.0
GSE37532_WT_VS_PPARG_KO_LN_TCONV_DN	172	0.32670268	14.867.358	0.00947226	0.011037309	1.0
GSE37301_PRO_BCELL_VS_GRANULOCYTE_MONOCYTE_PROGENITOR_DN	171	0.3306629	1.486.469	0.001349527	0.011074587	1.0
GSE37416_0H_VS_12H_F_TULARENSIS_LVS_NEUTROPHIL_UP	193	0.32321376	14.861.379	0.006648936	0.011127009	1.0
GSE26030_UNSTIM_VS_RESTIM_TH1_DAY15_POST_POLARIZATION_DN	196	0.3234729	14.857.394	0.006426735	0.011174901	1.0
GSE25677_R848_VS_MPL_AND_R848_STIM_BCELL_UP	134	0.33685583	14.855.103	0.009735744	0.011211989	1.0
GSE16385_ROSIGLITAZONE_IL4_VS_IL4_ALONE_STIM_MACROPHAGE_12H_DN	198	0.3199848	14.854.053	0.005221932	0.011219285	1.0
GSE14386_UNTREATED_VS_IFNA_TREATED_ACT_PBMC_MS_PATIENT_UP	137	0.3384944	14.846.545	0.006906077	0.011337277	1.0
GSE360_L_DONOVANI_VS_T_GONDII_MAC_UP	190	0.32282522	14.838.611	0.011968086	0.011443654	1.0
GSE25088_IL4_VS_IL4_AND_ROSIGLITAZONE_STIM_STAT6_KO_MACROPHAGE_DAY10_UP	191	0.32384294	1.483.192	0.003875969	0.011531368	1.0
GSE3982_BCELL_VS_CENT_MEMORY_CD4_TCELL_DN	189	0.32003894	14.832.067	0.00390625	0.011538126	1.0
GSE37605_NOD_VS_C57BL6_IRES_GFP_TREG_UP	150	0.33094126	14.817.337	0.006747638	0.011760363	1.0
GSE17721_POLYIC_VS_PAM3CSK4_16H_BMDM_UP	194	0.31913367	14.809.024	0.006468305	0.011905286	1.0
GSE19941_LPS_VS_LPS_AND_IL10_STIM_IL10_KO_MACROPHAGE_DN	151	0.32618898	14.807.844	0.012261581	0.011909565	1.0
GSE18791_CTRL_VS_NEWCASTLE_VIRUS_DC_1H_DN	164	0.32374662	14.808.475	0.004	0.011909636	1.0
GSE5589_LPS_VS_LPS_AND_IL10_STIM_MACROPHAGE_45MIN_UP	194	0.3219652	14.804.517	0.002570694	0.011963593	1.0
GSE13484_UNSTIM_VS_3H_YF17D_VACCINE_STIM_PBMC_DN	191	0.3245027	14.803.342	0.007832898	0.011973562	1.0
GSE38304_MYC_NEG_VS_POS_GC_BCELL_UP	195	0.32021135	14.801.389	0.001307189	0.011999267	1.0
GSE3691_IFN_PRODUCING_KILLER_DC_VS_CONVENTIONAL_DC_SPLEEN_DN	192	0.3202257	14.791.361	0.005277045	0.012172973	1.0
GSE360_L_MAJOR_VS_B_MALAYI_HIGH_DOSE_MAC_UP	191	0.32223415	14.787.731	0.004005340	0.012228537	1.0
GSE22432_CONVENTIONAL_CDC_VS_PLASMACYTOID_PDC_DN	190	0.32147703	14.785.665	0.001298701	0.012253768	1.0
GSE22601_DOUBLE_NEGATIVE_VS_CD8_SINGLE_POSITIVE_THYMOCYTE_UP	174	0.32403257	14.785.888	0.006613756	0.012257168	1.0
GSE35825_UNTREATED_VS_IFNG_STIM_MACROPHAGE_DN	178	0.31775695	14.781.253	0.005326231	0.012319257	1.0
GSE11057_EFF_MEM_VS_CENT_MEM_CD4_TCELL_DN	165	0.3274616	14.778.862	0.005390835	0.012349627	1.0
GSE22611_NOD2_TRANSD_VS_CTRL_TRANSD_HEK293_MDP_STIM_2H_UP	187	0.32125854	14.770.633	0.003896104	0.012493228	1.0
GSE22611_UNSTIM_VS_6H_MDP_STIM_NOD2_TRANSDUCED_HEK293T_CELL_DN	192	0.31817824	14.767.863	0.005284016	0.012525893	1.0
GSE43955_10H_VS_60H_ACT_CD4_TCELL_DN	195	0.31985795	14.765.078	0.0	0.012572302	1.0
GSE11961_FOLLICULAR_BCELL_VS_GERMINAL_CENTER_BCELL_DAY7_DN	188	0.32240134	14.757.872	0.005312085	0.012697292	1.0
GSE3920_IFNA_VS_IFNG_TREATED_ENDOTHELIAL_CELL_DN	163	0.32725027	14.738.606	0.005449591	0.01305661	1.0
GSE3039_CD4_TCELL_VS_ALPHAALPHA_CD8_TCELL_UP	190	0.3175725	14.733.812	0.008985879	0.013119234	1.0
GSE25087_FETAL_VS_ADULT_TCONV_DN	174	0.3246837	14.734.237	0.012362638	0.013120032	1.0
GSE369_PRE_VS_POST_IL6_INJECTION_IFNG_KO_LIVER_UP	192	0.319572	14.724.083	0.001349527	0.013278458	1.0
GSE3565_DUSP1_VS_WT_SPLENOCYTES_DN	177	0.32265908	14.723.223	0.005398110	0.013289347	1.0
GSE24142_DN2_VS_DN3_THYMOCYTE_FETAL_DN	197	0.3214706	14.716.471	0.003989361	0.013422051	1.0
GSE37534_UNTREATED_VS_GW1929_TREATED_CD4_TCELL_PPARG1_AND_FOXP3_TRASDUCED_UP	199	0.3180618	14.713.649	0.004143646	0.013459571	1.0
GSE11057_NAIVE_VS_MEMORY_CD4_TCELL_UP	175	0.32286844	14.713.178	0.010738255	0.013460412	1.0
GSE1432_CTRL_VS_IFNG_1H_MICROGLIA_DN	187	0.32240927	14.700.954	0.006640106	0.013690798	1.0
GSE21033_3H_VS_24H_POLYIC_STIM_DC_UP	156	0.32643262	14.695.408	0.011688312	0.013783853	1.0
GSE46606_IRF4_KO_VS_WT_CD40L_IL2_IL5_1DAY_STIMULATED_BCELL_UP	184	0.31747085	14.689.068	0.00660502	0.013910879	1.0
GSE3720_LPS_VS_PMA_STIM_VD2_GAMMADELTA_TCELL_DN	149	0.33281124	14.687.651	0.012379643	0.01393065	1.0
GSE22886_NAIVE_TCELL_VS_NEUTROPHIL_DN	186	0.31762984	1.468.665	0.00397878	0.013938595	1.0
GSE25088_IL4_VS_IL4_AND_ROSIGLITAZONE_STIM_MACROPHAGE_DAY10_DN	193	0.3169552	14.678.416	0.010471204	0.01409183	1.0
GSE557_WT_VS_CIITA_KO_DC_DN	190	0.3201401	14.671.673	0.009222661	0.014230618	1.0
GSE14308_TH2_VS_TH17_DN	189	0.31858715	14.669.342	0.005312085	0.014263621	1.0
GSE32255_WT_VS_JMJD2D_KNOCKDOWN_4H_LPS_STIM_DC_UP	147	0.332602	1.466.792	0.013586956	0.014276911	1.0
GSE17186_NAIVE_VS_CD21LOW_TRANSITIONAL_BCELL_UP	194	0.3179711	14.664.757	0.009234829	0.01433585	1.0
GSE14415_ACT_TCONV_VS_ACT_NATURAL_TREG_UP	170	0.3226306	14.661.506	0.006729475	0.014376115	1.0
GSE23114_WT_VS_SLE2C1_MOUSE_SPLEEN_B1A_BCELL_DN	195	0.31771263	14.661.839	0.005154639	0.014380188	1.0
GSE43863_TFH_VS_LY6C_INT_CXCR5POS_MEMORY_CD4_TCELL_UP	193	0.32152084	14.662.061	0.003931848	0.014381144	1.0
GSE2770_IL12_VS_TGFB_AND_IL12_TREATED_ACT_CD4_TCELL_2H_DN	159	0.32363516	14.659.154	0.008230452	0.01441337	1.0

GSE22886_NAIVE_VS_IGM_MEMORY_BCELL_UP		189	0.31801566	14.656.845	0.005174644	0.014443157	1.0
GSE2770_UNTREATED_VS_TGFB_AND_IL4_TREATED_ACT_CD4_TCELL_2H_DN		192	0.31438282	14.655.693	0.007926024	0.014457458	1.0
GSE13306_TREG_VS_TCONV_LAMINA_PROPRIA_UP		196	0.31893268	14.650.015	0.006596306	0.014576291	1.0
GSE36826_NORMAL_VS_STAPH_AUREUS_INF_IL1R_KO_SKIN_DN		189	0.31806237	14.642.465	0.006693440	0.01472624	1.0
GSE41176_UNSTIM_VS_ANTI_IGM_STIM_TAK1_KO_BCELL_3H_DN		187	0.31776106	14.634.398	0.00405954	0.014891245	1.0
GSE29949_MICROGLIA_VS_DC_BRAIN_UP		196	0.31742042	14.631.674	0.013123359	0.014935952	1.0
GSE4142_GC_BCELL_VS_MEMORY_BCELL_UP		192	0.313429	14.626.043	0.003921569	0.01503889	1.0
GSE25677_MPL_VS_MPL_AND_R848_STIM_BCELL_UP		157	0.32484064	14.624.268	0.004178273	0.015068183	1.0
GSE36888_UNTREATED_VS_IL2_TREATED_STAT5_AB_KNOCKIN_TCELL_2H_UP		186	0.31790912	14.622.914	0.003891050	0.015085153	1.0
GSE19888_CTRL_VS_A3R_ACTIVATION_MAST_CELL_DN		188	0.31636292	1.460.788	0.009198423	0.015398611	1.0
GSE7568_IL4_VS_IL4_AND_DEXAMETHASONE_TREATED_MACROPHAGE_DN		143	0.32605323	14.604.679	0.016689846	0.015457496	1.0
GSE30962_PRIMARY_VS_SECONDARY_ACUTE_LCMV_INF_CD8_TCELL_DN		189	0.31799376	14.600.196	0.006675567	0.015537224	1.0
GSE43955_TGFB_IL6_VS_TGFB_IL6_IL23_TH17_ACT_CD4_TCELL_52H_DN		195	0.31594932	14.600.508	0.003952569	0.015542586	1.0
GSE40443_INDUCED_VS_TOTAL_TREG_UP		197	0.31400782	14.598.935	0.006561679	0.015553806	1.0
GSE3920_IFNA_VS_IFNG_TREATED_FIBROBLAST_DN		181	0.31719124	14.594.289	0.007782101	0.015647212	1.0
GSE9006_HEALTHY_VS_TYPE_1_DIABETES_PBMC_AT_DX_DN		194	0.31665164	14.581.565	0.008152174	0.015908785	1.0
GSE7852_TREG_VS_TCONV_THYMUS_DN		192	0.31521553	14.571.862	0.011734028	0.016112076	1.0
GSE28783_CTRL_ANTI_MIR_VS_UNTREATED_ATHEROSCLEROSIS_MACROPHAGE_DN		196	0.31450516	14.569.062	0.006684492	0.016168503	1.0
GSE17721_PAM3CSK4_VS_GADIQUIMOD_16H_BMDM_UP		196	0.31596413	14.567.219	0.005208333	0.016203072	1.0
GSE37532_VISCERAL_ADIPOSE_TISSUE_VS_LN_DERIVED_TCONV_CD4_TCELL_UP		118	0.33643335	14.556.967	0.016105417	0.016424617	1.0
GSE26030_UNSTIM_VS_RESTIM_TH17_DAY5_POST_POLARIZATION_DN		191	0.31528756	1.455.513	0.010403121	0.016457012	1.0
GSE33374_CD8_ALPHAALPHA_VS_ALPHABETA_CD161_HIGH_TCELL_UP		197	0.31543556	1.455.461	0.008	0.01645779	1.0
GSE27786_BCELL_VS_CD4_TCELL_DN		188	0.3162334	14.547.687	0.009210526	0.01659813	1.0
GSE18893_CTRL_VS_TNF_TREATED_TREG_24H_DN		187	0.31489557	1.452.396	0.00794702	0.017150065	1.0
GSE12392_IFNAR_KO_VS_IFNB_KO_CD8_NEG_SPLEEN_DC_DN		197	0.3154797	14.520.322	0.009043927	0.017223053	1.0
GSE42724_NAIVE_VS_B1_BCELL_UP		194	0.31294745	14.512.547	0.003921569	0.017398901	1.0
GSE40068_CXCR5POS_BCL6POS_TFH_VS_CXCR5NEG_BCL6NEG_CD4_TCELL_DN		195	0.31467113	1.450.965	0.012362638	0.017445302	1.0
GSE22033_UNTREATED_VS_ROSIGLITAZONE_TREATED_MEF_DN		188	0.31526282	14.509.802	0.011734028	0.017455412	1.0
GSE2770_UNTREATED_VS_IL12_TREATED_ACT_CD4_TCELL_48H_UP		197	0.31371263	14.506.284	0.007702182	0.017515615	1.0
GSE41176_WT_VS_TAK1_KO_ANTI_IGM_STIM_BCELL_1H_DN		189	0.31466952	1.450.343	0.005333333	0.017570123	1.0
GSE5589_UNSTIM_VS_180MIN_LPS_STIM_MACROPHAGE_DN		191	0.31498176	14.500.539	0.009210526	0.017628776	1.0
GSE3720_UNSTIM_VS_PMA_STIM_VD1_GAMMADELTA_TCELL_UP		170	0.31746024	14.496.285	0.012178619	0.017737504	1.0
GSE17721_CTRL_VS_LPS_2H_BMDM_DN		195	0.31402695	14.493.608	0.00390625	0.017802043	1.0
GSE23984_CTRL_VS_HYPOCALEMIC_VITAMIND_ANALOG_TCELL_DN		192	0.31575593	14.490.471	0.009655172	0.017867047	1.0
GSE21360_SECONDARY_VS_QUATERNARY_MEMORY_CD8_TCELL_DN		159	0.32248664	14.486.351	0.004081632	0.017945353	1.0
GSE22886_NAIVE_VS_MEMORY_TCELL_DN		199	0.31150982	14.484.942	0.005089058	0.01797475	1.0
GSE27670_CTRL_VS_LMP1_TRANSDUCED_GC_BCELL_UP		198	0.31284788	1.448.264	0.003931848	0.018014923	1.0
GSE6269_FLU_VS_STAPH_AUREUS_INF_PBMC_DN		174	0.31658384	14.480.102	0.012048192	0.018063461	1.0
GSE17721_LPS_VS_CPG_1H_BMDM_DN		196	0.31304845	14.476.209	0.005102040	0.018146073	1.0
GSE3565_CTRL_VS_LPS_INJECTED_DUSP1_KO_SPLENOCYTES_DN		168	0.31813484	14.475.186	0.009887005	0.018165067	1.0
GSE27291_0H_VS_7D_STIM_GAMMADELTA_TCELL_UP		171	0.31816584	14.458.482	0.005312085	0.01854291	1.0
GSE42088_UNINF_VS_LEISHMANIA_INF_DC_4H_DN		196	0.3125733	14.448.893	0.007731958	0.018770872	1.0
GSE29949_CD8_POS_DC_SPLEEN_VS_MONOCYTE_BONE_MARROW_DN		197	0.30991122	14.446.084	0.007874016	0.018827751	1.0
GSE41867_DAY8_VS_DAY15_LCMV_ARMSTRONG_EFFECTOR_CD8_TCELL_UP		151	0.3239435	14.444.237	0.014986376	0.018861163	1.0
GSE3982_EOSINOPHIL_VS_NKCELL_UP		189	0.31296188	14.444.375	0.003942181	0.018869026	1.0
GOLDRATH_EFF_VS_MEMORY_CD8_TCELL_DN		197	0.31191838	14.442.974	0.009198423	0.01888437	1.0
GSE24142_EARLY_THYMIC_PROGENITOR_VS_DN3_THYMOCYTE_FETAL_DN		197	0.31079072	1.444.036	0.0	0.018933283	1.0
GSE41867_DAY8_VS_DAY15_LCMV_CLONE13_EFFECTOR_CD8_TCELL_UP		191	0.31260002	14.435.288	0.008152174	0.019045567	1.0
GSE36891_UNSTIM_VS_POLYIC_TLR3_STIM_PERITONEAL_MACROPHAGE_UP		144	0.3255184	14.433.775	0.013661202	0.019055977	1.0
GSE27786_LSK_VS_CD8_TCELL_DN		192	0.31428024	14.434.143	0.006485084	0.019060327	1.0
GSE12198_CTRL_VS_HIGH_IL2_STIM_NK_CELL_DN		192	0.31152022	14.431.336	0.009222661	0.019126868	1.0
GSE19941_UNSTIM_VS_LPS_STIM_IL10_KO_MACROPHAGE_DN		192	0.31063142	14.423.496	0.009333333	0.019328838	1.0
GSE36476_YOUNG_VS_OLD_DONOR_MEMORY_CD4_TCELL_DN		184	0.3145424	14.420.137	0.008141112	0.019402305	1.0
GSE17580_TREG_VS_TEFF_S_MANSONI_INF_UP		197	0.30980915	14.413.564	0.003957784	0.019557297	1.0
GSE34156_TLR1_TLR2_LIGAND_VS_NOD2_AND_TLR1_TLR2_LIGAND_6H_TREATED_MONOCYTE_UP		192	0.3126811	14.410.455	0.004026845	0.01963238	1.0
GSE23505_IL6_IL1_VS_IL6_IL1_IL23_TREATED_CD4_TCELL_DN		196	0.31051016	14.407.239	0.003875969	0.019705541	1.0
GSE3982_EOSINOPHIL_VS_EFF_MEMORY_CD4_TCELL_UP		189	0.3155115	14.406.059	0.009536785	0.019721624	1.0
GSE21678_WT_VS_FOXO1_FOXO3_KO_TREG_UP		179	0.31467688	14.403.987	0.006666667	0.01976191	1.0
GSE10147_IL3_VS_IL3_AND_HIVP17_STIM_PDC_DN		138	0.32691833	14.402.843	0.008174387	0.019780288	1.0
GSE28783_ANTI_MIR33_VS_UNTREATED_ATHEROSCLEROSIS_MACROPHAGE_UP		193	0.3100461	14.389.873	0.006729475	0.020113604	1.0
GSE5142_HTERT_TRANSDUCED_VS_CTRL_CD8_TCELL_LATE_PASSAGE_CLONE_DN		192	0.31221592	14.387.056	0.002638522	0.020169653	1.0
GSE6092_IFNG_VS_IFNG_AND_B_BURGDORFERI_INF_ENDOTHELIAL_CELL_UP		173	0.31647393	14.384.648	0.005340454	0.020217359	1.0
GSE29164_CD8_TCELL_VS_CD8_TCELL_AND_IL12_TREATED_MELANOMA_DAY3_DN		196	0.31221166	14.376.979	0.009198423	0.02040198	1.0
GSE1925_CTRL_VS_3H_IFNG_STIM_IFNG_PRIMED_MACROPHAGE_DN		193	0.31047428	14.376.447	0.011568123	0.020404518	1.0
GSE17186_NAIVE_VS_CD21HIGH_TRANSITIONAL_BCELL_CORD_BLOOD_DN		191	0.30963925	1.437.705	0.005208333	0.020412453	1.0
GSE45365_CTRL_VS_MCMV_INFECTION_NK_CELL_UP		196	0.31231725	14.375.345	0.007894737	0.02042587	1.0
GSE29618_PRE_VS_DAY7_POST_TIV_FLU_VACCINE_MDC_UP		194	0.31028813	14.367.508	0.010526316	0.020629643	1.0
GSE6875_WT_VS_FOXP3_KO_TREG_UP		188	0.3110798	1.436.214	0.005263158	0.020774921	1.0
GSE6092_CTRL_VS_BORRELIA_BIRGDOFERI_INF_ENDOTHELIAL_CELL_DN		142	0.32462636	14.355.066	0.016830295	0.020972332	1.0
GSE13411_SWITCHED_MEMORY_BCELL_VS_PLASMA_CELL_DN		189	0.31144908	14.351.408	0.006657789	0.021038493	1.0
GSE17721_CTRL_VS_LPS_1H_BMDM_DN		194	0.31032366	1.435.184	0.006443299	0.021039927	1.0
GSE37532_TREG_VS_TCONV_CD4_TCELL_FROM_LN_DN		194	0.31323275	14.350.677	0.007672634	0.021043008	1.0
GSE45365_WT_VS_IFNAR_KO_CD8A_DC_DN		197	0.31304583	14.348.775	0.007894737	0.021082802	1.0
GSE40277_EOS_AND_LEF1_TRANSDUCED_VS_CTRL_CD4_TCELL_UP		196	0.3124586	14.339.191	0.015424165	0.021331342	1.0
GSE23321_CD8_STEM_CELL_MEMORY_VS_CENTRAL_MEMORY_CD8_TCELL_UP		193	0.31058496	14.336.786	0.005524862	0.021383846	1.0
GSE17721_LPS_VS_PAM3CSK4_24H_BMDM_DN		192	0.31040925	14.334.803	0.009222661	0.021415837	1.0
GSE40685_NAIVE_CD4_TCELL_VS_TREG_DN		188	0.30920848	14.335.268	0.002577319	0.021416293	1.0
GSE28737_WT_VS_BCL6_KO_FOLLICULAR_BCELL_UP		192	0.31104735	14.333.049	0.005277045	0.021443516	1.0
GSE17186_BLOOD_VS_CORD_BLOOD_CD21HIGH_TRANSITIONAL_BCELL_DN		191	0.31343052	14.330.373	0.008163265	0.021511678	1.0
GSE2770_IL12_VS_TGFB_AND_IL12_TREATED_ACT_CD4_TCELL_6H_UP		187	0.3127388	14.319.365	0.006684492	0.021793693	1.0
GSE19888_ADENOSINE_A3R_INH_PRETREAT_AND_ACT_BY_A3R_VS_A3R_INH_AND_TCELL_MEMBRANES_ACT_MAST_CELL		174	0.31275702	14.319.656	0.012228261	0.021795532	1.0
GSE9509_10MIN_VS_30MIN_LPS_STIM_IL10_KO_MACROPHAGE_UP		192	0.31158388	14.313.091	0.005242464	0.021956414	1.0
GSE6092_B_BURGDOFERI_VS_B_BURGDORFERI_AND_IFNG_STIM_ENDOTHELIAL_CELL_DN		175	0.3138617	14.308.484	0.00802139	0.022065084	1.0
GSE15330_HSC_VS_MEGAKARYOCYTE_ERYTHROID_PROGENITOR_DN		165	0.3127944	1.430.434	0.017663043	0.02216884	1.0
GSE5142_CTRL_VS_HTERT_TRANSDUCED_CD8_TCELL_EARLY_PASSAGE_CLONE_DN		193	0.3107672	14.302.331	0.007843138	0.022197975	1.0
GSE37301_HEMATOPOIETIC_STEM_CELL_VS_COMMON_LYMPHOID_PROGENITOR_UP		185	0.31165084	14.302.609	0.011968086	0.022206463	1.0
GSE20366_TREG_VS_NAIVE_CD4_TCELL_UP		194	0.308033	14.300.476	0.006535948	0.02221335	1.0
GSE360_L_DONOVANI_VS_B_MALAYI_HIGH_DOSE_DC_DN		198	0.3072391	14.301.314	0.014175258	0.022213455	1.0
GSE19941_UNSTIM_VS_LPS_STIM_IL10_KO_NFKBP50_KO_MACROPHAGE_DN		194	0.30715236	14.300.563	0.009198423	0.022225337	1.0

GSE19401_UNSTIM_VS_PAM2CSK4_STIM_FOLLICULAR_DC_DN	194	0.31170797	14.298.226	0.0064850845	0.022261424	1.0
GSE9037_CTRL_VS_LPS_4H_STIM_IRAK4_KO_BMDM_DN	194	0.30927417	14.296.488	0.0013071896	0.022282893	1.0
GSE3982_EFF_MEMORY_CD4_TCELL_VS_TH1_UP	187	0.30939445	14.296.764	0.011703511	0.02228675	1.0
GSE24972_MARGINAL_ZONE_BCELL_VS_FOLLICULAR_BCELL_DN	195	0.30541292	14.281.683	0.009126467	0.022715336	1.0
GSE22935_UNSTIM_VS_48H_MBOVIS_BCG_STIM_MYD88_KO_MACROPHAGE_DN	193	0.30935997	142.817	0.007968128	0.022728162	1.0
GSE360_L_MAJOR_VS_B_MALAYI_HIGH_DOSE_DC_UP	195	0.30751404	14.279.507	0.0077220076	0.02276361	1.0
GSE43863_TH1_VS_LY6C_INT_CXCR5POS_EFFECTOR_CD4_TCELL_DN	195	0.30760202	14.277.864	0.010471204	0.022782624	1.0
GSE15330_LYMPHOID_MULTIPOTENT_VS_MEGAKARYOCYTE_ERYTHROID_PROGENITOR_IKAROS_KO_UP	164	0.3174513	14.277.871	0.016172506	0.02279605	1.0
GSE6269_HEALTHY_VS_E_COLI_INF_PBMC_UP	167	0.31335297	14.263.777	0.006702413	0.02318848	1.0
GSE13484_UNSTIM_VS_12H_YF17D_VACCINE_STIM_PBMC_DN	196	0.30945554	14.263.378	0.01303781	0.023189312	1.0
GSE18804_BRAIN_VS_COLON_TUMORAL_MACROPHAGE_UP	182	0.31076548	14.261.688	0.009497965	0.023229009	1.0
GSE22919_RESTING_VS_IL2_IL12_IL15_STIM_NK_CELL_DN	189	0.3096493	14.260.164	0.014765101	0.023270946	1.0
GSE20484_MCSG_VS_CXCL4_MONOCYTE_DERIVED_MACROPHAGE_DN	189	0.30990186	14.259.117	0.011749348	0.023276076	1.0
GSE41867_NAIVE_VS_DAY15_LCMV_EFFECTOR_CD8_TCELL_DN	189	0.3094106	14.259.155	0.012953368	0.023289172	1.0
GSE21063_CTRL_VS_ANTI_IGM_STIM_BCELL_16H_DN	192	0.3083777	14.258.165	0.01511335	0.023291936	1.0
GSE3720_UNSTIM_VS_LPS_STIM_VD2_GAMMADELTA_TCELL_DN	143	0.31888542	1.425.342	0.01458886	0.02341623	1.0
GSE8921_UNSTIM_0H_VS_TLR1_2_STIM_MONOCYTE_24H_UP	188	0.31005523	1.425.286	0.0116731515	0.023419242	1.0
GSE5589_WT_VS_IL6_KO_LPS_AND_IL10_STIM_MACROPHAGE_180MIN_UP	98	0.34152472	14.252.179	0.03085554	0.023421124	1.0
GSE10240_IL17_VS_IL17_AND_IL22_STIM_PRIMARY_BRONCHIAL_EPITHELIAL_CELLS_UP	192	0.30836576	14.249.947	0.0053050397	0.023473158	1.0
GSE25087_FETAL_VS_ADULT_TREG_DN	178	0.31069043	14.244.559	0.018445322	0.023635741	1.0
GSE18203_CTRL_VS_INTRATUMORAL_CPG_INJ_MC38_TUMOR_UP	191	0.30786481	14.229.113	0.0091743115	0.02408366	1.0
GSE43955_TH0_VS_TGFB_IL6_TH17_ACT_CD4_TCELL_10H_DN	194	0.30649212	14.228.355	0.009358289	0.024090875	1.0
GSE7568_CTRL_VS_24H_TGFB_TREATED_MACROPHAGES_WITH_IL4_AND_DEXAMETHASONE_DN	195	0.30816182	14.224.854	0.011984021	0.024187788	1.0
GSE24081_CONTROLLER_VS_PROGRESSOR_HIV_SPECIFIC_CD8_TCELL_UP	189	0.30780497	14.212.884	0.009345794	0.024553774	1.0
GSE41978_WT_VS_BIM_KO_KLRG1_LOW_EFFECTOR_CD8_TCELL_UP	195	0.3061415	14.207.724	0.010638298	0.024721524	1.0
GSE40666_UNTREATED_VS_IFNA_STIM_STAT4_KO_EFFECTOR_CD8_TCELL_90MIN_DN	191	0.30570635	14.201.614	0.009210526	0.02491728	1.0
GSE39864_WT_VS_GATA3_KO_TREG_UP	198	0.30969104	14.201.027	0.010512483	0.024926307	1.0
GSE22886_TH1_VS_TH2_48H_ACT_UP	192	0.30951777	14.199.768	0.009150327	0.024938058	1.0
GSE27434_WT_VS_DNMT1_KO_TREG_DN	196	0.30644774	14.200.006	0.010568032	0.024944508	1.0
GSE16697_CD4_TCELL_VS_TFH_CD4_TCELL_DN	193	0.30888	14.197.327	0.0131233595	0.02499692	1.0
GSE17721_LPS_VS_POLYIC_16H_BMDM_DN	190	0.30710566	14.193.358	0.0066225166	0.02511522	1.0
GSE3982_MAC_VS_CENT_MEMORY_CD4_TCELL_DN	187	0.30679816	14.191.881	0.010256411	0.025148153	1.0
GSE17721_0.5H_VS_4H_CPG_BMDM_DN	194	0.30699536	1.418.831	0.01462766	0.025221925	1.0
GSE1448_CTRL_VS_ANTI_VALPHA2_DP_THYMOCYTE_DN	194	0.30563253	14.188.691	0.0025806453	0.025226831	1.0
GSE2770_UNTREATED_VS_ACT_CD4_TCELL_6H_UP	188	0.3087766	14.182.842	0.01171875	0.025378108	1.0
GSE18893_TCONV_VS_TREG_2H_CULTURE_DN	194	0.30973884	14.177.547	0.006459948	0.025542734	1.0
GSE36095_WT_VS_HDAC9_KO_TREG_DN	115	0.3329564	14.174.366	0.011267605	0.02564093	1.0
GSE9988_ANTI_TREM1_VS_ANTI_TREM1_AND_LPS_MONOCYTE_UP	190	0.30783683	14.172.819	0.006459948	0.025675701	1.0
GSE16385_UNTREATED_VS_12H_IL4_TREATED_MACROPHAGE_UP	193	0.3125385	14.170.607	0.010989011	0.025730358	1.0
GSE26488_WT_VS_HDAC7_DELTAP_TG_OT2_THYMOCYTE_WITH_PEPTIDE_INJECTION_UP	197	0.3043117	14.168.978	0.014304291	0.025759913	1.0
GSE37301_HEMATOPOIETIC_STEM_CELL_VS_CD4_TCELL_DN	129	0.32434654	14.167.355	0.019417476	0.02579682	1.0
GSE6259_33D1_POS_DC_VS_BCELL_DN	142	0.3203884	14.166.162	0.024623804	0.02582062	1.0
GSE22601_DOUBLE_NEGATIVE_VS_CD4_SINGLE_POSITIVE_THYMOCYTE_UP	193	0.30816886	1.416.294	0.010269577	0.025903046	1.0
GSE6259_33D1_POS_VS_DEC205_POS_FLT3L_INDUCED_SPLENIC_DC_UP	169	0.3099391	14.157.088	0.009615385	0.026079698	1.0
GSE3982_DC_VS_TH1_DN	196	0.3057646	14.154.379	0.005235602	0.026136972	1.0
GSE6674_UNSTIM_VS_PL2_3_STIM_BCELL_DN	165	0.3132304	14.154.383	0.01510989	0.026152037	1.0
GSE8685_IL2_STARVED_VS_IL2_ACT_IL2_STARVED_CD4_TCELL_UP	192	0.30441377	14.144.331	0.0066401064	0.026468145	1.0
GSE45739_NRAS_KO_VS_WT_UNSTIM_CD4_TCELL_DN	181	0.307298	1.414.231	0.013192612	0.026514776	1.0
GSE13229_IMM_VS_MATURE_NKCELL_DN	189	0.30920658	14.140.323	0.009222661	0.0265725	1.0
GSE2706_2H_VS_8H_LPS_STIM_DC_UP	191	0.3071875	14.137.491	0.012903226	0.026658673	1.0
GSE19198_1H_VS_6H_IL21_TREATED_TCELL_DN	197	0.30439615	141.359	0.017038008	0.026703976	1.0
GSE17721_LPS_VS_PAM3CSK4_12H_BMDM_UP	193	0.30513585	14.135.135	0.007989348	0.026716841	1.0
GSE3982_MAC_VS_EFF_MEMORY_CD4_TCELL_DN	186	0.30878115	14.132.631	0.015645372	0.026775658	1.0
GSE10240_CTRL_VS_IL17_STIM_PRIMARY_BRONCHIAL_EPITHELIAL_CELLS_DN	196	0.30837202	1.412.711	0.014120667	0.026934164	1.0
GSE17721_POLYIC_VS_GARDIQUIMOD_12H_BMDM_UP	189	0.30440032	14.127.506	0.014647137	0.026937168	1.0
GSE22025_UNTREATED_VS_TGFB1_AND_PROGESTERONE_TREATED_CD4_TCELL_UP	193	0.3045257	1.412.314	0.006684492	0.027071122	1.0
GSE19512_NAUTRAL_VS_INDUCED_TREG_DN	194	0.3042881	1.411.842	0.016064256	0.027214892	1.0
GSE37532_VISCERAL_ADIPOSE_TISSUE_VS_LN_DERIVED_TREG_CD4_TCELL_DN	146	0.31918058	1.411.876	0.016129032	0.027219737	1.0
GSE21927_HEALTHY_VS_TUMOROUS_BALBC_MOUSE_MONOCYTE_DN	159	0.3167279	14.110.608	0.020604396	0.027483856	1.0
GSE3565_CTRL_VS_LPS_INJECTED_SPLENOCYTES_DN	171	0.31117022	14.109.492	0.017931035	0.02749402	1.0
GSE27786_LSK_VS_NEUTROPHIL_DN	194	0.3042256	14.109.799	0.01193634	0.027499635	1.0
GSE6269_E_COLI_VS_STREP_PNEUMO_INF_PBMC_UP	157	0.31420526	14.108.335	0.020746889	0.02751824	1.0
GSE27786_CD8_TCELL_VS_NKTCELL_DN	191	0.30532935	14.106.919	0.0078125	0.027557608	1.0
GSE17721_LPS_VS_GARDIQUIMOD_0.5H_BMDM_UP	195	0.30530378	14.105.945	0.012064343	0.027574476	1.0
GSE13522_CTRL_VS_T_CRUZI_Y_STRAIN_INF_SKIN_IFNG_KO_DN	133	0.31918734	14.103.185	0.022630835	0.027651452	1.0
GSE32255_WT_UNSTIM_VS_JMJD2D_KNOCKDOWN_4H_LPS_STIM_DC_UP	168	0.30960447	14.097.614	0.023841059	0.027810728	1.0
GSE15930_NAIVE_VS_24H_IN_VITRO_STIM_CD8_TCELL_UP	198	0.30297002	14.095.908	0.0052015604	0.027866786	1.0
GSE9988_LPS_VS_LPS_AND_ANTI_TREM1_MONOCYTE_DN	179	0.3063509	1.409.396	0.013422819	0.027919319	1.0
GSE14699_NAIVE_VS_ACT_CD8_TCELL_DN	172	0.30785233	1.409.242	0.015625	0.027952835	1.0
GSE25677_MPL_VS_MPL_AND_R848_STIM_BCELL_DN	117	0.32691988	14.091.513	0.02467344	0.027970506	1.0
GSE3982_DC_VS_BASOPHIL_UP	190	0.30574757	14.083.503	0.011857707	0.028218744	1.0
GSE13411_NAIVE_VS_IGM_MEMORY_BCELL_UP	187	0.30950454	14.079.798	0.013020833	0.028341979	1.0
GSE3982_MAC_VS_BCELL_DN	189	0.30608693	14.077.159	0.018041236	0.02842875	1.0
GSE15930_NAIVE_VS_48H_IN_VITRO_STIM_IL12_CD8_TCELL_UP	194	0.30344933	14.072.989	0.019607844	0.028565634	1.0
GSE10147_IL3_AND_HIVP17_VS_IL3_AND_CPG_STIM_PDC_UP	147	0.3169051	1.406.809	0.015789473	0.028742637	1.0
GSE19941_IL10_KO_VS_IL10_KO_AND_NFKBP50_KO_LPS_AND_IL10_STIM_MACROPHAGE_UP	196	0.30566183	14.067.295	0.014360313	0.028752076	1.0
GSE17721_PAM3CSK4_VS_GADIQUIMOD_6H_BMDM_DN	192	0.30606	14.062.763	0.010403121	0.028881896	1.0
GSE43955_1H_VS_20H_ACT_CD4_TCELL_DN	193	0.3037789	14.060.081	0.013280213	0.028968703	1.0
GSE14000_UNSTIM_VS_16H_LPS_DC_TRANSLATED_RNA_UP	186	0.30516768	14.058.375	0.012987013	0.0290208	1.0
GSE22611_MUTANT_NOD2_TRANSDUCED_VS_CTRL_HEK293T_STIMULATED_WITH_MDP_6H_UP	185	0.30731755	14.053.937	0.018348623	0.029168155	1.0
GSE2128_C57BL6_VS_NOD_THYMOCYTE_MIMETOPE_NEGATIVE_SELECTION_UP	196	0.30400676	14.050.208	0.0091743115	0.029257571	1.0
GSE30971_CTRL_VS_LPS_STIM_MACROPHAGE_WBP7_KO_4H_UP	180	0.30628598	14.050.587	0.016	0.029259056	1.0
GSE7460_TREG_VS_TCONV_ACT_WITH_TGFB_UP	190	0.30545405	14.046.855	0.011538462	0.029365616	1.0
GSE13411_SWITCHED_MEMORY_BCELL_VS_PLASMA_CELL_UP	189	0.30194795	14.044.857	0.01458886	0.029423613	1.0
GSE13522_WT_VS_IFNG_KO_SKIN_UP	120	0.32449567	14.040.126	0.02312925	0.029568085	1.0
GSE7548_DAY7_VS_DAY28_PCC_IMMUNIZATION_CD4_TCELL_DN	194	0.30691236	14.037.702	0.009032258	0.029647432	1.0
GSE26928_NAIVE_VS_CENT_MEMORY_CD4_TCELL_DN	179	0.30417275	14.023.502	0.015665796	0.030176878	1.0
GSE37563_WT_VS_CTLA4_KO_CD4_TCELL_D4_POST_IMMUNIZATION_UP	156	0.3097802	14.019.848	0.013908206	0.030298542	1.0
GSE21380_TFH_VS_GERMINAL_CENTER_TFH_CD4_TCELL_UP	193	0.30465695	14.017.003	0.011764706	0.030390756	1.0

GSE33425_CD161_HIGH_VS_NEG_CD8_TCELL_UP	199	0.30333793	14.014.734	0.015228426	0.03045456	1.0
GSE15330_WT_VS_IKAROS_KO_HSC_UP	197	0.30254424	14.012.583	0.015936255	0.030502751	1.0
GSE1460_NAIVE_CD4_TCELL_ADULT_BLOOD_VS_THYMIC_STROMAL_CELL_DN	191	0.30626592	14.011.968	0.008010681	0.030509464	1.0
GSE43955_TH0_VS_TGFB_IL6_TH17_ACT_CD4_TCELL_42H_DN	198	0.30313787	14.012.666	0.005284016	0.030517096	1.0
GSE46606_IRF4HIGH_VS_IRF4MID_CD40L_IL2_IL5_DAY3_STIMULATED_BCELL_DN	193	0.30311337	14.011.085	0.018276762	0.030521117	1.0
GSE2197_CPG_DNA_VS_UNTREATED_IN_DC_UP	192	0.30219752	14.008.769	0.01660281	0.03056856	1.0
GSE17721_PAM3CSK4_VS_CPG_16H_BMDM_DN	193	0.29970732	14.009.217	0.010471204	0.03056971	1.0
GSE45739_NRAS_KO_VS_WT_ACD3_ACD28_STIM_CD4_TCELL_DN	185	0.30527693	14.007.962	0.018181818	0.030581811	1.0
GSE22196_HEALTHY_VS_OBESE_MOUSE_SKIN_GAMMADELTA_TCELL_DN	198	0.3026267	14.005.706	0.011795544	0.030645201	1.0
GSE22432_MULTIPOTENT_PROGENITOR_VS_PDC_UP	191	0.30336663	14.004.033	0.016949153	0.030686403	1.0
GSE15930_STIM_VS_STIM_AND_TRICHOSTATINA_24H_CD8_T_CELL_UP	196	0.30076635	14.000.285	0.014511873	0.030811308	1.0
GSE22935_UNSTIM_VS_48H_MBOVIS_BCG_STIM_MACROPHAGE_DN	197	0.30235273	13.999.319	0.006675567	0.030831054	1.0
GSE27786_LSK_VS_NKCELL_DN	190	0.30248415	13.998.158	0.015483871	0.030855067	1.0
GSE20366_EX_VIVO_VS_DEC205_CONVERSION_DN	189	0.30568138	1.399.708	0.018494055	0.03087468	1.0
GSE22886_NAIVE_CD8_TCELL_VS_MEMORY_TCELL_UP	185	0.3014954	13.991.797	0.021333333	0.031046169	1.0
GSE7460_TCONV_VS_TREG_THYMUS_UP	195	0.30231133	13.991.082	0.011749348	0.031053595	1.0
GSE6259_BCELL_VS_CD8_TCELL_DN	181	0.30734405	13.984.201	0.01193634	0.031281795	1.0
GSE6259_33D1_POS_VS_DEC205_POS_SPLENIC_DC_DN	188	0.30357307	13.984.435	0.012278308	0.031291	1.0
GSE11386_NAIVE_VS_MEMORY_BCELL_DN	154	0.3100867	13.983.117	0.014304291	0.03130273	1.0
GSE9316_CD4_TCELL_BALBC_VS_TH17_ENRI_CD4_TCELL_SKG_PMA_IONO_STIM_FR4NEG_DN	196	0.30358312	13.977.224	0.007905139	0.031512514	1.0
GSE360_CTRL_VS_L_MAJOR_DC_UP	194	0.2996801	13.975.383	0.01759134	0.031531114	1.0
GSE27670_CTRL_VS_LMP1_TRANSDUCED_GC_BCELL_DN	197	0.30306828	13.976.128	0.009433962	0.03154095	1.0
GSE40274_CTRL_VS_FOXP3_AND_SATB1_TRANSDUCED_ACTIVATED_CD4_TCELL_UP	158	0.30998263	139.754	0.024324324	0.031547528	1.0
GSE9650_EXHAUSTED_VS_MEMORY_CD8_TCELL_UP	193	0.3039311	13.973.653	0.006811989	0.03156817	1.0
GSE22033_WT_VS_PPARG_KO_MEF_UP	188	0.30698273	1.396.486	0.014435695	0.03189975	1.0
GSE17721_0.5H_VS_8H_LPS_BMDM_DN	193	0.30257905	13.964.301	0.012064343	0.0319006	1.0
GSE17721_LPS_VS_GARDIQUIMOD_12H_BMDM_UP	190	0.30183885	13.962.088	0.023746701	0.031978365	1.0
GSE5542_UNTREATED_VS_IFNG_TREATED_EPITHELIAL_CELLS_6H_UP	181	0.30362636	13.958.007	0.018276762	0.03210323	1.0
GSE19772_CTRL_VS_HCMV_INF_MONOCYTES_DN	167	0.31141052	13.958.482	0.015006822	0.032105707	1.0
GSE45881_CXCR6HI_VS_CXCR1LO_COLONIC_LAMINA_PROPRIA_UP	194	0.30214614	13.957.467	0.022457067	0.032107256	1.0
GSE41978_KLRG1_HIGH_VS_LOW_EFFECTOR_CD8_TCELL_UP	197	0.29949197	13.955.423	0.007915568	0.03217507	1.0
GSE9006_TYPE_1_DIABETES_AT_DX_VS_4MONTH_POST_DX_PBMC_UP	197	0.3016992	13.952.602	0.006459948	0.03227155	1.0
GSE17974_0H_VS_1H_IN_VITRO_ACT_CD4_TCELL_DN	179	0.30650088	13.948.938	0.009138381	0.032409344	1.0
GSE8835_CD4_VS_CD8_TCELL_DN	193	0.30352703	13.943.577	0.011857707	0.032619756	1.0
GSE13547_WT_VS_ZFX_KO_BCELL_ANTI_IGM_STIM_2H_DN	153	0.31148356	13.940.367	0.022849463	0.032738168	1.0
GSE43955_1H_VS_20H_ACT_CD4_TCELL_UP	196	0.29976717	13.939.008	0.009259259	0.0327863	1.0
GSE40274_FOXP3_VS_FOXP3_AND_XBP1_TRANSDUCED_ACTIVATED_CD4_TCELL_UP	146	0.313459	13.936.747	0.018918918	0.032847974	1.0
GSE42021_CD24HI_TREG_VS_CD24HI_TCONV_THYMUS_DN	197	0.29820916	13.935.503	0.010484928	0.03286229	1.0
GSE17301_CTRL_VS_48H_ACD3_ACD28_STIM_CD8_TCELL_UP	196	0.30175292	1.393.573	0.017038008	0.032872234	1.0
GSE30153_LUPUS_VS_HEALTHY_DONOR_BCELL_DN	194	0.29990926	13.929.667	0.015957447	0.0330909	1.0
GSE22025_PROGESTERONE_VS_TGFB1_AND_PROGESTERONE_TREATED_CD4_TCELL_DN	193	0.301568	13.929.156	0.012594459	0.03309114	1.0
GSE34515_CD16_NEG_VS_POS_MONOCYTE_UP	192	0.30251032	13.928.058	0.02254642	0.033116266	1.0
GSE9509_10MIN_VS_30MIN_LPS_AND_IL10_STIM_IL10_KO_MACROPHAGE_DN	154	0.3109244	13.926.125	0.02328767	0.03317434	1.0
GSE5589_LPS_VS_LPS_AND_IL6_STIM_IL6_KO_MACROPHAGE_45MIN_DN	193	0.30245167	13.924.414	0.020053476	0.03321939	1.0
GSE45365_HEALTHY_VS_MCMV_INFECTION_BCELL_DN	160	0.30773783	13.914.356	0.021828104	0.03364261	1.0
GSE17186_BLOOD_VS_CORD_BLOOD_CD21LOW_TRANSITIONAL_BCELL_UP	192	0.29949468	13.910.906	0.022818793	0.033768807	1.0
GSE20727_DNFB_ALLERGEN_VS_ROS_INH_AND_DNFB_ALLERGEN_TREATED_DC_UP	177	0.3044512	13.908.505	0.017567568	0.033852413	1.0
GSE45365_HEALTHY_VS_MCMV_INFECTION_CD8_TCELL_IFNAR_KO_DN	163	0.3060809	13.904.223	0.030261349	0.034028415	1.0
GSE29949_DC_BRAIN_VS_MONOCYTE_BONE_MARROW_DN	192	0.30179387	13.902.016	0.011464968	0.03411436	1.0
GSE11961_GERMINAL_CENTER_BCELL_DAY7_VS_PLASMA_CELL_DAY7_DN	191	0.3018624	13.896.238	0.018348623	0.03434734	1.0
GSE27786_BCELL_VS_NKCELL_DN	190	0.3015906	1.388.954	0.01310616	0.034599822	1.0
GSE24972_WT_VS_IRF8_KO_MARGINAL_ZONE_SPLEEN_BCELL_UP	193	0.3005962	13.886.476	0.01894452	0.034688435	1.0
GSE17721_LPS_VS_GARDIQUIMOD_1H_BMDM_UP	193	0.30011216	13.886.617	0.014511873	0.034702025	1.0
GSE7348_UNSTIM_VS_LPS_STIM_MACROPHAGE_UP	146	0.3132736	13.883.532	0.021216407	0.034753118	1.0
GSE7852_LN_VS_FAT_TREG_DN	194	0.30100784	13.883.873	0.01142132	0.034756497	1.0
GSE4142_NAIVE_BCELL_VS_PLASMA_CELL_UP	191	0.29978698	13.883.905	0.01591512	0.034774378	1.0
GSE27786_BCELL_VS_NEUTROPHIL_DN	197	0.30107972	13.879.592	0.023195876	0.034923	1.0
GSE3337_CTRL_VS_4H_IFNG_IN_CD8POS_DC_UP	195	0.2987035	13.878.407	0.01632653	0.034957636	1.0
GSE12198_CTRL_VS_HIGH_IL2_STIM_NK_CELL_UP	194	0.29821748	1.387.654	0.017925736	0.035008848	1.0
GSE7460_FOXP3_MUT_VS_WT_ACT_TCONV_DN	190	0.30215046	13.870.746	0.014360313	0.03522841	1.0
GSE37301_PRO_BCELL_VS_CD4_TCELL_UP	162	0.30630717	1.386.851	0.018741634	0.035260174	1.0
GSE46606_DAY1_VS_DAY3_CD40L_IL2_IL5_STIMULATED_IRF4MID_BCELL_DN	193	0.30164114	13.868.953	0.019788919	0.035263855	1.0
GSE29617_CTRL_VS_TIV_FLU_VACCINE_PBMC_2008_UP	170	0.30359077	13.869.146	0.024804177	0.03527288	1.0
GSE3982_CTRL_VS_PMA_STIM_EOSINOPHIL_DN	190	0.29743674	13.864.594	0.02008032	0.035396658	1.0
GSE29618_PRE_VS_DAY7_POST_LAIV_FLU_VACCINE_MDC_DN	185	0.30241024	13.862.114	0.02016129	0.03545184	1.0
GSE17721_CTRL_VS_CPG_4H_BMDM_DN	197	0.30018607	13.862.115	0.013071896	0.035471067	1.0
GSE17721_LPS_VS_GARDIQUIMOD_24H_BMDM_UP	191	0.30080652	13.859.261	0.012080537	0.035555996	1.0
GSE10211_UV_INACT_SENDAI_VS_LIVE_SENDAI_VIRUS_TRACHEAL_EPITHELIAL_CELLS_UP	141	0.31208023	13.851.575	0.039617486	0.035863	1.0
GSE46242_TH1_VS_ANERGIC_TH1_CD4_TCELL_UP	183	0.30207485	13.851.839	0.019788919	0.035870112	1.0
KAECH_DAY8_EFF_VS_MEMORY_CD8_TCELL_DN	196	0.3015577	13.846.703	0.014531043	0.036057852	1.0
GSE43955_TH0_VS_TGFB_IL6_IL23_TH17_ACT_CD4_TCELL_52H_DN	195	0.29880133	13.834.884	0.020215634	0.03649937	1.0
GSE29615_CTRL_VS_DAY7_LAIV_FLU_VACCINE_PBMC_UP	165	0.30608737	13.830.428	0.04199475	0.03666919	1.0
GSE22886_NAIVE_CD4_TCELL_VS_NKCELL_UP	186	0.30084628	13.827.124	0.01997337	0.036797192	1.0
GSE26928_NAIVE_VS_EFF_MEMORY_CD4_TCELL_DN	183	0.30155337	13.825.065	0.024804177	0.036883157	1.0
GSE27786_LIN_NEG_VS_MONO_MAC_DN	192	0.29965237	1.382.332	0.01591512	0.03694037	1.0
GSE1460_CD4_THYMOCYTE_VS_NAIVE_CD4_TCELL_ADULT_BLOOD_UP	190	0.3001223	13.821.805	0.023346303	0.036972255	1.0
GSE41176_UNSTIM_VS_ANTI_IGM_STIM_BCELL_6H_DN	192	0.2993787	13.822.228	0.022368422	0.03697304	1.0
GSE31082_DP_VS_CD4_SP_THYMOCYTE_DN	191	0.30024332	1.382.124	0.014416776	0.036975168	1.0
GSE3039_ALPHABETA_CD8_TCELL_VS_B1_BCELL_UP	194	0.2988525	13.813.928	0.010256411	0.03728918	1.0
GSE5503_PLN_DC_VS_SPLEEN_DC_ACTIVATED_ALLOGENIC_TCELL_DN	193	0.29761526	13.811.338	0.024739584	0.03738096	1.0
GSE2706_R848_VS_R848_AND_LPS_8H_STIM_DC_DN	177	0.30467477	13.809.826	0.022666667	0.037418593	1.0
GSE14308_TH2_VS_TH1_DN	194	0.29930878	1.380.853	0.018348623	0.03742859	1.0
GSE16522_ANTI_CD3CD28_STIM_VS_UNSTIM_MEMORY_CD8_TCELL_DN	189	0.29815513	13.808.551	0.029639175	0.037448704	1.0
GSE17721_POLYIC_VS_CPG_2H_BMDM_DN	193	0.29948157	13.802.493	0.014102564	0.037674893	1.0
GSE9988_ANTI_TREM1_VS_CTRL_TREATED_MONOCYTES_UP	184	0.30186456	1.379.753	0.026350461	0.03788143	1.0
GSE21033_1H_VS_24H_POLYIC_STIM_DC_DN	157	0.3052584	13.796.585	0.028767124	0.037898157	1.0
GSE9037_WT_VS_IRAK4_KO_LPS_4H_STIM_BMDM_DN	195	0.2978566	13.796.115	0.023746701	0.03790316	1.0
GSE17186_NAIVE_VS_CD21LOW_TRANSITIONAL_BCELL_CORD_BLOOD_DN	196	0.30090255	13.795.387	0.018041236	0.0379103	1.0
GSE37605_C57BL6_VS_NOD_FOXP3_FUSION_GFP_TREG_DN	182	0.30470642	13.789.531	0.021136064	0.038195487	1.0

GSE25123_CTRL_VS_IL4_STIM_MACROPHAGE_DN	191	0.29641646	13.784.895	0.019710906	0.038398392	1.0
GSE35543_IN_VITRO_ITREG_VS_CONVERTED_EX_ITREG_DN	191	0.2990061	13.781.711	0.015604681	0.038527627	1.0
GSE27786_BCELL_VS_NKTCELL_DN	192	0.29850122	13.778.136	0.022106633	0.038677253	1.0
GSE18804_SPLEEN_MACROPHAGE_VS_COLON_TUMORAL_MACROPHAGE_DN	191	0.2971961	13.777.632	0.020806242	0.0386824	1.0
GSE40685_NAIVE_CD4_TCELL_VS_FOXP3_KO_TREG_PRECURSOR_DN	183	0.3033283	13.776.336	0.014230272	0.038722288	1.0
GSE15215_CD2_POS_VS_NEG_PDC_UP	187	0.30040878	13.772.852	0.017925736	0.038873095	1.0
GSE369_PRE_VS_POST_IL6_INJECTION_IFNG_KO_LIVER_DN	193	0.30229935	1.376.672	0.019946808	0.039139476	1.0
GSE24574_BCL6_HIGH_TFH_VS_TCONV_CD4_TCELL_UP	193	0.2972523	13.762.447	0.02035278	0.039290898	1.0
GSE6269_FLU_VS_STREP_PNEUMO_INF_PBMC_UP	162	0.30304998	13.762.758	0.023841059	0.039298188	1.0
GSE2585_AIRE_KO_VS_WT_CD80_LOW_MTEC_DN	196	0.29646906	13.761.605	0.015345269	0.03931407	1.0
KAECH_DAY15_EFF_VS_MEMORY_CD8_TCELL_DN	197	0.29795587	1.375.828	0.012690355	0.039448306	1.0
GSE360_LOW_DOSE_B_MALAYI_VS_M_TUBERCULOSIS_DC_UP	198	0.2969694	13.754.417	0.012	0.039611917	1.0
GSE40274_LEF1_VS_FOXP3_AND_LEF1_TRANSDUCED_ACTIVATED_CD4_TCELL_UP	198	0.29836562	13.750.255	0.02116402	0.039780457	1.0
GSE360_CTRL_VS_B_MALAYI_HIGH_DOSE_DC_UP	189	0.29839003	13.746.976	0.019430052	0.03989759	1.0
GSE37301_LYMPHOID_PRIMED_MPP_VS_CD4_TCELL_UP	183	0.30153427	13.740.107	0.02075227	0.040192965	1.0
GSE15330_MEGAKARYOCYTE_ERYTHROID_PROGENITOR_VS_PRO_BCELL_UP	191	0.29790366	13.738.359	0.02312925	0.04024591	1.0
GSE17721_POLYIC_VS_GARDIQUIMOD_0.5H_BMDM_UP	191	0.30134916	13.733.777	0.010568032	0.040452376	1.0
GSE23321_CD8_STEM_CELL_MEMORY_VS_EFFECTOR_MEMORY_CD8_TCELL_UP	198	0.29550612	13.732.362	0.027704485	0.040488433	1.0
GSE20715_WT_VS_TLR4_KO_24H_OZONE_LUNG_UP	195	0.29591933	13.730.601	0.022457067	0.040562995	1.0
GSE29615_CTRL_VS_LAIV_FLU_VACCINE_PBMC_DN	145	0.30726495	13.727.944	0.03021978	0.04066683	1.0
GOLDRATH_NAIVE_VS_EFF_CD8_TCELL_UP	197	0.29714894	13.724.145	0.02356021	0.04082785	1.0
GSE24726_WT_VS_E2-2_KO_PDC_DN	195	0.29638678	13.720.838	0.016883116	0.040957667	1.0
GSE20715_WT_VS_TLR4_KO_LUNG_DN	189	0.29799202	1.372.087	0.028985508	0.040976208	1.0
GSE7460_CTRL_VS_TGFB_TREATED_ACT_FOXP3_MUT_TCONV_UP	195	0.29577404	13.719.624	0.019633507	0.040976293	1.0
GSE32034_UNTREATED_VS_ROSIGLIZATONE_TREATED_LY6C_HIGH_MONOCYTE_UP	193	0.29469004	13.720.003	0.018018018	0.040980782	1.0
GSE4142_NAIVE_VS_MEMORY_BCELL_UP	193	0.29839745	13.717.567	0.019283747	0.041057132	1.0
GSE39820_CTRL_VS_IL1B_IL6_CD4_TCELL_UP	194	0.29978022	13.716.956	0.017127799	0.04106558	1.0
GSE17721_CTRL_VS_LPS_12H_BMDM_DN	192	0.2966457	13.712.643	0.020408163	0.04126534	1.0
GSE7831_CPG_VS_INFLUENZA_STIM_PDC_1H_UP	197	0.29345274	13.705.268	0.019430052	0.041617043	1.0
GSE16385_MONOCYTE_VS_12H_IFNG_TNF_TREATED_MACROPHAGE_DN	190	0.2945404	13.701.835	0.015345269	0.04176228	1.0
GSE7460_TREG_VS_TCONV_ACT_DN	188	0.29882997	1.370.107	0.02008032	0.04178645	1.0
GSE13306_TREG_VS_TCONV_UP	190	0.2965498	13.694.437	0.01270648	0.042088658	1.0
GSE8835_HEALTHY_VS_CLL_CD4_TCELL_DN	188	0.29597872	13.693.922	0.020833334	0.042096518	1.0
GSE15330_LYMPHOID_MULTIPOTENT_VS_PRO_BCELL_DN	191	0.2973196	13.689.505	0.019710906	0.04228309	1.0
GSE18281_CORTICAL_VS_MEDULLARY_THYMOCYTE_DN	192	0.29950735	13.688.897	0.020942409	0.04229248	1.0
GSE17721_LPS_VS_PAM3CSK4_16H_BMDM_UP	192	0.2961489	13.683.437	0.028795812	0.042490724	1.0
GSE1432_6H_VS_24H_IFNG_MICROGLIA_UP	196	0.29720342	13.684.226	0.02356021	0.042499125	1.0
GSE360_L_MAJOR_VS_B_MALAYI_LOW_DOSE_MAC_UP	185	0.2975519	13.683.568	0.019505851	0.042506292	1.0
GSE19888_ADENOSINE_A3R_INH_VS_ACT_WITH_INHIBITOR_PRETREATMENT_IN_MAST_CELL_DN	195	0.29728845	13.680.478	0.02610966	0.042634334	1.0
GSE17721_PAM3CSK4_VS_CPG_2H_BMDM_DN	194	0.29508668	13.675.629	0.02642008	0.04286544	1.0
GSE339_EX_VIVO_VS_IN_CULTURE_CD8POS_DC_DN	197	0.29758832	13.672.578	0.028423773	0.042990573	1.0
GSE15735_2H_VS_12H_HDAC_INHIBITOR_TREATED_CD4_TCELL_DN	196	0.29236817	13.669.757	0.027954256	0.04311307	1.0
GSE3720_UNSTIM_VS_PMA_STIM_VD2_GAMMADELTA_TCELL_DN	193	0.2944881	13.667.598	0.014230272	0.043171924	1.0
GSE20715_WT_VS_TLR4_KO_6H_OZONE_LUNG_UP	193	0.29697207	1.365.826	0.010526316	0.043628164	1.0
GSE8685_IL15_ACT_IL2_STARVED_VS_IL21_ACT_IL2_STARVED_CD4_TCELL_UP	158	0.3034821	13.657.205	0.03155007	0.04366615	1.0
GSE30083_SP1_VS_SP2_THYMOCYTE_UP	192	0.296166	13.656.151	0.016949153	0.043701947	1.0
GSE2826_XID_VS_BTK_KO_BCELL_DN	197	0.2935133	13.653.218	0.016993465	0.043836575	1.0
GSE1925_CTRL_VS_IFNG_PRIMED_MACROPHAGE_24H_IFNG_STIM_UP	193	0.29492798	13.651.503	0.02389937	0.043900102	1.0
GSE15930_NAIVE_VS_24H_IN_VITRO_STIM_INFAB_CD8_TCELL_UP	198	0.2927151	13.644.428	0.014492754	0.044234473	1.0
GSE360_DC_VS_MAC_DN	190	0.2952416	13.642.157	0.025740026	0.044311117	1.0
GSE17721_LPS_VS_CPG_6H_BMDM_UP	195	0.2947706	13.642.222	0.01965924	0.044330135	1.0
GSE22935_UNSTIM_VS_48H_MBOVIS_BCG_STIM_MYD88_KO_MACROPHAGE_UP	191	0.29641715	13.636.396	0.016172506	0.044596463	1.0
GSE23505_UNTREATED_VS_4DAY_IL6_IL1_TGFB_TREATED_CD4_TCELL_UP	194	0.29426527	1.363.652	0.022193212	0.04461455	1.0
GSE40666_STAT1_KO_VS_STAT4_KO_CD8_TCELL_WITH_IFNA_STIM_90MIN_DN	161	0.3030732	13.634.765	0.026737968	0.04466042	1.0
GSE7831_UNSTIM_VS_CPG_STIM_PDC_4H_UP	197	0.29690447	13.630.649	0.022457067	0.044840116	1.0
GSE22601_DOUBLE_NEGATIVE_VS_DOUBLE_POSITIVE_THYMOCYTE_DN	197	0.2928188	1.362.997	0.017832648	0.04485316	1.0
GSE13887_ACT_CD4_VS_NO_TREATED_CD4_TCELL_DN	121	0.31214198	1.362.746	0.047210302	0.044964537	1.0
GSE39110_DAY3_VS_DAY6_POST_IMMUNIZATION_CD8_TCELL_WITH_IL2_TREATMENT_DN	190	0.29532298	13.625.615	0.02075227	0.045044526	1.0
GSE19772_CTRL_VS_HCMV_INF_MONOCYTES_AND_PI3K_INHIBITION_DN	199	0.29600957	13.624.957	0.022427442	0.045056395	1.0
GSE30971_2H_VS_4H_LPS_STIM_MACROPHAGE_WBP7_KO_DN	192	0.2976217	1.361.542	0.022339027	0.045541145	1.0
GSE11884_WT_VS_FURIN_KO_NAIVE_CD4_TCELL_UP	133	0.30879495	1.361.337	0.03561644	0.045626305	1.0
GSE17721_LPS_VS_CPG_12H_BMDM_DN	194	0.2917836	13.608.618	0.02425876	0.04585241	1.0
GSE14699_NAIVE_VS_ACT_CD8_TCELL_UP	191	0.2924113	13.607.004	0.022849463	0.045915425	1.0
GSE6269_STAPH_AUREUS_VS_STREP_PNEUMO_INF_PBMC_DN	169	0.29789004	13.603.591	0.02872777	0.046060443	1.0
GSE17721_LPS_VS_PAM3CSK4_4H_BMDM_UP	194	0.29530784	13.600.088	0.023622047	0.04623642	1.0
GSE5542_IFNG_VS_IFNA_TREATED_EPITHELIAL_CELLS_6H_DN	191	0.2929989	13.599.074	0.024707412	0.046270594	1.0
GSE10463_CD40L_AND_VA347_VS_CD40L_IN_DC_DN	163	0.30016515	13.595.817	0.032742154	0.046415742	1.0
GSE25088_CTRL_VS_IL4_AND_ROSIGLITAZONE_STIM_MACROPHAGE_UP	183	0.29223272	13.593.946	0.036	0.046478726	1.0
GSE17721_4_VS_24H_CPG_BMDM_UP	194	0.29576132	13.594.186	0.018893387	0.046491552	1.0
GSE3720_VD1_VS_VD2_GAMMADELTA_TCELL_DN	195	0.2917227	13.585.789	0.011734028	0.04687959	1.0
GSE22025_UNTREATED_VS_PROGESTERONE_TREATED_CD4_TCELL_DN	195	0.292446	13.585.998	0.014360313	0.046895616	1.0
GSE26488_WT_VS_VP16_TRANSGENIC_HDAC7_KO_DOUBLE_POSITIVE_THYMOCYTE_UP	193	0.29313213	13.583.627	0.027704485	0.046992507	1.0
GSE18791_UNSTIM_VS_NEWCATSLE_VIRUS_DC_2H_UP	157	0.30009425	13.567.935	0.03256445	0.047831904	1.0
GSE3920_UNTREATED_VS_IFNB_TREATED_ENDOTHELIAL_CELL_UP	190	0.29439682	1.355.694	0.023407022	0.04838813	1.0
GSE14415_FOXP3_KO_NATURAL_TREG_VS_TCONV_DN	167	0.30019534	13.557.236	0.03396739	0.048400383	1.0
GSE4748_CTRL_VS_LPS_AND_CYANOBACTERIUM_LPSLIKE_STIM_DC_3H_DN	200	0.29354929	13.553.518	0.01884253	0.04854711	1.0
GSE23308_CTRL_VS_CORTICOSTERONE_TREATED_MACROPHAGE_DN	195	0.29490003	1.355.178	0.017402945	0.04859346	1.0
GSE360_CTRL_VS_B_MALAYI_LOW_DOSE_DC_UP	193	0.2971614	1.355.207	0.022849463	0.048602745	1.0
GSE36888_UNTREATED_VS_IL2_TREATED_STAT5_AB_KNOCKIN_TCELL_17H_DN	197	0.29382417	13.549.703	0.029372497	0.048671726	1.0
GSE9946_MATURE_STIMULATORY_VS_PROSTAGLANDINE2_TREATED_MATURE_DC_DN	122	0.3130118	13.548.348	0.050946143	0.0487206	1.0
GSE10273_HIGH_IL7_VS_HIGH_IL7_AND_IRF4_IN_IRF4_8_NULL_PRE_BCELL_DN	194	0.2917976	13.544.092	0.022486772	0.048945397	1.0
GSE3203_WT_VS_IFNAR1_KO_INFLUENZA_INFECTED_LN_BCELL_UP	191	0.29203016	13.542.136	0.028947368	0.04904309	1.0
GSE32423_CTRL_VS_IL7_IL4_MEMORY_CD8_TCELL_UP	199	0.29041103	13.535.445	0.019946808	0.04938192	1.0
GSE17721_LPS_VS_PAM3CSK4_8H_BMDM_UP	194	0.29231793	13.533.559	0.022929937	0.049463052	1.0
GSE25088_CTRL_VS_IL4_STIM_MACROPHAGE_DN	188	0.2924724	13.530.157	0.023529412	0.04962391	1.0
GSE45365_NK_CELL_VS_CD8A_DC_UP	198	0.29220515	13.528.203	0.026246719	0.049719475	1.0
GSE40666_STAT1_KO_VS_STAT4_KO_CD8_TCELL_DN	157	0.30132398	1.352.674	0.036885247	0.049779207	1.0
