# Supplementary material for: Lymphocytic infiltration in stage II microsatellite stable colorectal tumors: A retrospective prognosis biomarker analysis
Source: PLoS Med. 2020 Sep 24;17(9):e1003292. doi: 10.1371/journal.pmed.1003292 (PMC7514069; doi:10.1371/journal.pmed.1003292)
Supplement: S2 Table — (DOCX) [file pmed.1003292.s003.docx]

**Supplementary Table 2.** Regression tables

**ICO CLX**

Tumor TCR abundance categorical

|  | hr | 95% CI | P-val |
| --- | --- | --- | --- |
| TIL2_high | 0.25 | (0.10-0.63) | 0.0030 |
| sexMale | 1.38 | (0.50-3.76) | 0.5338 |
| age | 0.99 | (0.95-1.04) | 0.7386 |
| siteRight | 0.85 | (0.34-2.10) | 0.7206 |
| stageIIB | 3.18 | (0.85-11.88) | 0.0846 |

Tumor TCR abundance continuous

|  | hr | 95% CI | P-val |
| --- | --- | --- | --- |
| tcr | 0.71 | (0.57-0.89) | 0.0029 |
| sexMale | 1.30 | (0.47-3.59) | 0.6120 |
| age | 0.99 | (0.95-1.04) | 0.7234 |
| siteRight | 0.76 | (0.31-1.91) | 0.5655 |
| stageII-B | 2.51 | (0.71-8.86) | 0.1535 |

Tumor clonality index categorical

|  | hr | 95% CI | P-val |
| --- | --- | --- | --- |
| CLON2_high | 2.32 | (0.90-5.97) | 0.0820 |
| sexMale | 1.49 | (0.54-4.14) | 0.4434 |
| age | 0.98 | (0.93-1.03) | 0.4318 |
| siteRight | 0.72 | (0.28-1.82) | 0.4838 |
| stageIIB | 2.63 | (0.73-9.55) | 0.1406 |

Tumor clonality index continuous

|  | hr | 95% CI | P-val |
| --- | --- | --- | --- |
| clon | 1.26 | (0.67-2.35) | 0.4732 |
| sexMale | 1.39 | (0.50-3.85) | 0.5224 |
| age | 0.98 | (0.94-1.03) | 0.5165 |
| siteRight | 0.78 | (0.31-1.95) | 0.5882 |
| stageII-B | 2.18 | (0.62-7.67) | 0.2223 |

Normal TCR abundance categorical

|  | hr | 95% CI | P-val |
| --- | --- | --- | --- |
| TIL_high | 0.57 | (0.20-1.60) | 0.2840 |
| sexMale | 1.29 | (0.47-3.56) | 0.6245 |
| age | 0.99 | (0.94-1.04) | 0.7112 |
| siteRight | 0.75 | (0.30-1.88) | 0.5415 |
| stageIIB | 1.89 | (0.55-6.47) | 0.3132 |

Normal clonality index categorical

|  | hr | 95% CI | P-val |
| --- | --- | --- | --- |
| CLON_n2_high | 1.46 | (0.52-4.08) | 0.4742 |
| sexMale | 1.35 | (0.48-3.79) | 0.5669 |
| age | 1.00 | (0.95-1.05) | 0.8639 |
| siteRight | 0.70 | (0.27-1.83) | 0.4661 |
| stageIIB | 1.38 | (0.30-6.29) | 0.6784 |

TIL estroma (pathologist)

|  | hr | 95% CI | P-val |
| --- | --- | --- | --- |
| TIL_EST2_high | 0.33 | (0.14-0.78) | 0.0117 |
| sexMale | 1.25 | (0.46-3.41) | 0.6692 |
| age | 0.99 | (0.95-1.04) | 0.6819 |
| siteRight | 0.84 | (0.34-2.10) | 0.7098 |
| stageIIB | 2.16 | (0.62-7.57) | 0.2283 |

TIL intraepithelial (pathologist)

|  | hr | 95% CI | P-val |
| --- | --- | --- | --- |
| TIL_intratum2_high | 0.33 | (0.04-2.64) | 0.2962 |
| sexMale | 1.25 | (0.45-3.46) | 0.6627 |
| age | 0.99 | (0.94-1.03) | 0.5669 |
| siteRight | 0.95 | (0.37-2.42) | 0.9101 |
| stageIIB | 1.96 | (0.57-6.74) | 0.2848 |

% ESTROMA (pathologist)

|  | hr | 95% CI | P-val |
| --- | --- | --- | --- |
| EST2_high | 3.30 | (0.91-11.95) | 0.0688 |
| sexMale | 1.22 | (0.44-3.36) | 0.7062 |
| age | 1.00 | (0.95-1.05) | 0.8982 |
| siteRight | 0.75 | (0.30-1.89) | 0.5469 |
| stageIIB | 2.35 | (0.68-8.16) | 0.1790 |

pathologist-immunoseq combination

|  | hr | 95% CI | P-val |
| --- | --- | --- | --- |
| comb2_stLymph-low, TCR-high | 0.38 | (0.10-1.42) | 0.1512 |
| comb3_stLymph-high, TCR-low | 0.42 | (0.12-1.53) | 0.1896 |
| comb4_stLymph-high, TCR-high | 0.11 | (0.03-0.36) | 0.0003 |
| sexMale | 1.26 | (0.45-3.54) | 0.6636 |
| age | 0.98 | (0.94-1.03) | 0.4848 |
| siteRight | 0.86 | (0.33-2.19) | 0.7449 |
| stageIIB | 3.17 | (0.83-12.05) | 0.0905 |

**ICO/FF**

TCR Abundance categorical

|  | hr | 95% CI | P-val |
| --- | --- | --- | --- |
| TIL2_high | 0.30 | (0.12-0.72) | 0.0074 |
| genderMale | 2.20 | (0.84-5.77) | 0.1087 |
| age | 1.04 | (0.99-1.10) | 0.0948 |
| stageII-B | 2.20 | (0.68-7.08) | 0.1859 |
| locationright | 0.17 | (0.05-0.57) | 0.0045 |

TCR Abundance continuous

|  | hr | 95% CI | P-val |
| --- | --- | --- | --- |
| tcr | 0.68 | (0.51-0.91) | 0.0103 |
| sexMale | 2.07 | (0.80-5.38) | 0.1346 |
| age | 1.04 | (0.99-1.09) | 0.1358 |
| siteRight | 0.14 | (0.04-0.51) | 0.0029 |
| stageII-B | 2.46 | (0.76-7.97) | 0.1347 |

Clonality index categorical

|  | hr | 95% CI | P-val |
| --- | --- | --- | --- |
| CLON2_high | 3.32 | (1.38-7.94) | 0.0072 |
| genderMale | 2.30 | (0.85-6.17) | 0.0993 |
| age | 1.05 | (0.99-1.10) | 0.0906 |
| stageII-B | 1.71 | (0.56-5.23) | 0.3486 |
| locationright | 0.18 | (0.05-0.64) | 0.0080 |

Clonality index continuous

|  | hr | 95% CI | P-val |
| --- | --- | --- | --- |
| clon | 1.56 | (0.87-2.80) | 0.1345 |
| sexMale | 2.06 | (0.78-5.46) | 0.1444 |
| age | 1.04 | (0.99-1.10) | 0.1218 |
| siteRight | 0.18 | (0.05-0.61) | 0.0061 |
| stageII-B | 1.94 | (0.63-6.00) | 0.2513 |

TCR abundance in treated

|  | hr | 95% CI | P-val |
| --- | --- | --- | --- |
| TIL2_high | 0.22 | (0.06-0.79) | 0.0200 |
| genderMale | 1.25 | (0.33-4.78) | 0.7440 |
| age | 1.06 | (0.96-1.16) | 0.2442 |
| stageII-B | 1.43 | (0.36-5.71) | 0.6094 |
| locationright | 0.09 | (0.01-0.72) | 0.0237 |

Abundance in non treated

|  | hr | 95% CI | P-val |
| --- | --- | --- | --- |
| TIL2_high | 0.34 | (0.09-1.27) | 0.1088 |
| genderMale | 3.23 | (0.77-13.57) | 0.1091 |
| age | 1.07 | (0.98-1.17) | 0.1300 |
| stageII-B | 4.08 | (0.44-37.96) | 0.2164 |
| locationright | 0.27 | (0.05-1.31) | 0.1033 |

**ICO/FFPE**

TCR abundance categorical

|  | hr | 95% CI | P-val |
| --- | --- | --- | --- |
| tcr_ca2_High | 0.41 | (0.18-0.93) | 0.0339 |
| sexMale | 1.92 | (0.75-4.90) | 0.1731 |
| age | 1.04 | (1.00-1.08) | 0.0830 |
| siteRight | 0.65 | (0.26-1.62) | 0.3522 |
| stageII-B | 1.26 | (0.42-3.82) | 0.6789 |
| msiMSS | 2.33 | (0.29-18.97) | 0.4281 |

TCR Abundance continuous

|  | 0.78 | 95% CI | P-val |
| --- | --- | --- | --- |
| tcr | 1.94 | (0.49-1.23) | 0.2880 |
| sexMale | 1.04 | (0.76-4.95) | 0.1678 |
| age | 0.65 | (1.00-1.09) | 0.0746 |
| siteRight | 1.11 | (0.26-1.63) | 0.3592 |
| stageII-B | 2.46 | (0.37-3.31) | 0.8569 |
| msiMSS | 0.78 | (0.30-20.03) | 0.3992 |

Clonality index categorical

|  | hr | 95% CI | P-val |
| --- | --- | --- | --- |
| clon_ca2_High | 0.71 | (0.30-1.68) | 0.4384 |
| sexMale | 1.95 | (0.76-5.01) | 0.1643 |
| age | 1.04 | (1.00-1.08) | 0.0677 |
| siteRight | 0.64 | (0.25-1.61) | 0.3399 |
| stageII-B | 0.96 | (0.33-2.83) | 0.9401 |
| msiMSS | 2.35 | (0.29-19.26) | 0.4265 |

Clonality index continuous

|  | hr | 95% CI | P-val |
| --- | --- | --- | --- |
| clon | 1.06 | (0.57-1.94) | 0.8621 |
| sexMale | 1.87 | (0.73-4.80) | 0.1922 |
| age | 1.04 | (0.99-1.08) | 0.0895 |
| siteRight | 0.67 | (0.26-1.67) | 0.3875 |
| stageII-B | 1.00 | (0.34-2.94) | 0.9997 |
| msiMSS | 2.49 | (0.30-20.36) | 0.3960 |

TCR abundance excluding MSI categorical

|  | hr | 95% CI | P-val |
| --- | --- | --- | --- |
| tcr_ca2_high | 0.40 | (0.16-0.98) | 0.0447 |
| sexMale | 1.65 | (0.59-4.58) | 0.3387 |
| age | 1.04 | (0.99-1.09) | 0.0847 |
| siteRight | 0.80 | (0.30-2.12) | 0.6534 |
| stageII-B | 1.69 | (0.55-5.25) | 0.3613 |

TCR abundance excluding MSI continuous

|  | hr | 95% CI | P-val |
| --- | --- | --- | --- |
| tcr | 0.97 | (0.92-1.03) | 0.2916 |
| sexMale | 1.64 | (0.59-4.57) | 0.3476 |
| age | 1.05 | (1.00-1.10) | 0.0668 |
| siteRight | 0.84 | (0.32-2.21) | 0.7233 |
| stageII-B | 1.53 | (0.50-4.72) | 0.4606 |

Clonality index excluding MSI categorical

|  | hr | 95% CI | P-val |
| --- | --- | --- | --- |
| clon_ca2_High | 0.74 | (0.28-1.98) | 0.5520 |
| sexMale | 1.70 | (0.61-4.75) | 0.3100 |
| age | 1.05 | (1.00-1.10) | 0.0696 |
| siteRight | 0.81 | (0.31-2.13) | 0.6694 |
| stageII-B | 1.32 | (0.43-4.04) | 0.6249 |

Clonality index excluding MSI continuous

|  | hr | 95% CI | P-val |
| --- | --- | --- | --- |
| clon | 1.03 | (0.97-1.09) | 0.3575 |
| sexMale | 1.57 | (0.56-4.41) | 0.3873 |
| age | 1.04 | (0.99-1.09) | 0.0939 |
| siteRight | 0.84 | (0.32-2.21) | 0.7259 |
| stageII-B | 1.48 | (0.48-4.54) | 0.4951 |

**MECC**

TCR abundance categorical

|  | hr | 95% CI | P-val |
| --- | --- | --- | --- |
| tcr_ca2_High | 0.56 | (0.31-1.00) | 0.0482 |
| sexMale | 0.88 | (0.51-1.50) | 0.6291 |
| age | 1.01 | (0.98-1.04) | 0.4577 |
| siteRight | 0.92 | (0.51-1.65) | 0.7744 |
| siteUnknown | 0.00 | (0.00-Inf) | 0.9958 |
| stageII-B | 6.96 | (3.08-15.71) | 0.0000 |
| mssMSI | 0.91 | (0.38-2.16) | 0.8228 |

TCR abundance continuous

|  | hr | 95% CI | P-val |
| --- | --- | --- | --- |
| tcr | 0.87 | (0.69-1.10) | 0.2461 |
| sexMale | 0.84 | (0.49-1.45) | 0.5360 |
| age | 1.01 | (0.98-1.04) | 0.5660 |
| siteRight | 0.92 | (0.51-1.65) | 0.7838 |
| siteUnknown | 0.00 | (0.00-Inf) | 0.9958 |
| stageII-B | 6.83 | (3.01-15.48) | 0.0000 |
| mssMSI | 0.86 | (0.36-2.06) | 0.7288 |

Clonality index categorical

|  | hr | 95% CI | P-val |
| --- | --- | --- | --- |
| clon_ca2_High | 2.45 | (1.39-4.32) | 0.0020 |
| sexMale | 0.88 | (0.50-1.56) | 0.6681 |
| age | 1.01 | (0.98-1.04) | 0.6009 |
| siteRight | 0.69 | (0.37-1.29) | 0.2472 |
| siteUnknown | 0.00 | (0.00-Inf) | 0.9958 |
| stageII-B | 6.79 | (2.82-16.33) | 0.0000 |
| mssMSI | 0.83 | (0.35-2.01) | 0.6851 |

Clonality index continuous

|  | hr | 95% CI | P-val |
| --- | --- | --- | --- |
| clon | 1.28 | (0.95-1.72) | 0.1076 |
| sexMale | 0.84 | (0.47-1.47) | 0.5359 |
| age | 1.01 | (0.98-1.04) | 0.6021 |
| siteRight | 0.76 | (0.41-1.43) | 0.3993 |
| siteUnknown | 0.00 | (0.00-Inf) | 0.9959 |
| stageII-B | 5.87 | (2.43-14.14) | 0.0001 |
| msiMSS | 0.92 | (0.38-2.21) | 0.8438 |

TCR abundance excluding MSI categorical

|  | hr | 95% CI | P-val |
| --- | --- | --- | --- |
| tcr_ca2_High | 0.52 | (0.28-0.95) | 0.0323 |
| sexMale | 0.85 | (0.48-1.49) | 0.5587 |
| age | 1.02 | (0.99-1.05) | 0.3089 |
| siteRight | 1.06 | (0.58-1.93) | 0.8446 |
| siteUnknown | 0.00 | (0.00-Inf) | 0.9959 |
| stageII-B | 7.58 | (3.18-18.08) | 0.0000 |

TCR abundance excluding MSI continuous

|  | hr | 95% CI | P-val |
| --- | --- | --- | --- |
| tcr | 0.98 | (0.94-1.03) | 0.4630 |
| sexMale | 0.81 | (0.46-1.43) | 0.4659 |
| age | 1.01 | (0.98-1.04) | 0.4253 |
| siteRight | 1.06 | (0.59-1.93) | 0.8428 |
| siteUnknown | 0.00 | (0.00-Inf) | 0.9960 |
| stageII-B | 7.43 | (3.08-17.92) | 0.0000 |

Clonality index excluding MSI categorical

|  | hr | 95% CI | P-val |
| --- | --- | --- | --- |
| clon_ca2_high | 2.58 | (1.41-4.71) | 0.0021 |
| sexMale | 0.87 | (0.48-1.57) | 0.6350 |
| age | 1.01 | (0.98-1.04) | 0.4837 |
| siteRight | 0.78 | (0.41-1.49) | 0.4470 |
| siteUnknown | 0.00 | (0.00-Inf) | 0.9959 |
| stageII-B | 7.31 | (2.82-18.94) | 0.0000 |

Clonality index excluding MSI continuous

|  | hr | 95% CI | P-val |
| --- | --- | --- | --- |
| clon | 1.02 | (1.00-1.03) | 0.0561 |
| sexMale | 0.80 | (0.44-1.45) | 0.4694 |
| age | 1.01 | (0.98-1.04) | 0.4341 |
| siteRight | 0.89 | (0.47-1.68) | 0.7198 |
| siteUnknown | 0.00 | (0.00-Inf) | 0.9960 |
| stageII-B | 6.84 | (2.65-17.63) | 0.0001 |

**all stage II MSS**

TCR abundance categorical

|  | hr | 95% CI | P-val |
| --- | --- | --- | --- |
| tcr_ca2_high | 0.39 | (0.26-0.57) | 0.0000 |
| sexMale | 1.15 | (0.78-1.69) | 0.4801 |
| age | 1.02 | (1.00-1.04) | 0.0268 |
| siteRight | 0.72 | (0.48-1.08) | 0.1096 |
| siteUnknown | 0.00 | (0.00-Inf) | 0.9943 |
| stageII-B | 2.85 | (1.65-4.93) | 0.0002 |

TCR abundance continuous

|  | hr | 95% CI | P-val |
| --- | --- | --- | --- |
| tcr_z | 0.85 | (0.78-0.93) | 0.0002 |
| sexMale | 1.10 | (0.75-1.61) | 0.6400 |
| age | 1.02 | (1.00-1.04) | 0.0464 |
| siteRight | 0.72 | (0.48-1.09) | 0.1196 |
| siteUnknown | 0.00 | (0.00-Inf) | 0.9943 |
| stageII-B | 2.69 | (1.56-4.64) | 0.0004 |

Clonality index categorical

|  | hr | 95% CI | P-val |
| --- | --- | --- | --- |
| clon_ca2_high | 2.13 | (1.44-3.15) | 0.0002 |
| sexMale | 1.16 | (0.78-1.71) | 0.4696 |
| age | 1.02 | (1.00-1.04) | 0.1088 |
| siteRight | 0.63 | (0.41-0.96) | 0.0333 |
| siteUnknown | 0.00 | (0.00-Inf) | 0.9944 |
| stageII-B | 2.46 | (1.41-4.31) | 0.0016 |

Clonality index continuous

|  | hr | 95% CI | P-val |
| --- | --- | --- | --- |
| clon_z | 1.16 | (1.03-1.31) | 0.0161 |
| sexMale | 1.11 | (0.75-1.65) | 0.5914 |
| age | 1.02 | (1.00-1.04) | 0.0968 |
| siteRight | 0.65 | (0.43-1.00) | 0.0492 |
| siteUnknown | 0.00 | (0.00-Inf) | 0.9946 |
| stageII-B | 2.36 | (1.35-4.12) | 0.0025 |

TCR abundance & clonality index categorical

|  | hr | 95% CI | P-val |
| --- | --- | --- | --- |
| comb2_TCR-low/CLON-high | 1.34 | (0.72-2.49) | 0.3548 |
| comb3_TCR-high/ClON-low | 0.38 | (0.22-0.65) | 0.0004 |
| comb4_TCR-high, CLON-high | 0.84 | (0.46-1.53) | 0.5695 |
| data$sexMale | 1.17 | (0.79-1.74) | 0.4287 |
| data$age | 1.02 | (1.00-1.04) | 0.0590 |
| data$stageII-B | 2.73 | (1.55-4.80) | 0.0005 |
| data$siteRight | 0.63 | (0.41-0.97) | 0.0359 |
| data$siteUnknown | 0.00 | (0.00-Inf) | 0.9944 |

**all stage II-B MSS**

TCR abundance categorical

|  | hr | 95% CI | P-val |
| --- | --- | --- | --- |
| tcr_ca2_High | 0.11 | (0.02-0.59) | 0.0102 |
| sexMale | 1.16 | (0.37-3.60) | 0.8028 |
| age | 1.14 | (1.04-1.24) | 0.0052 |
| siteRight | 0.57 | (0.13-2.40) | 0.4414 |

TCR abundance continuous

|  | hr | 95% CI | P-val |
| --- | --- | --- | --- |
| tcr | 0.87 | (0.76-1.01) | 0.0589 |
| sexMale | 0.67 | (0.23-1.92) | 0.4521 |
| age | 1.09 | (1.01-1.17) | 0.0208 |
| siteRight | 0.61 | (0.14-2.62) | 0.5013 |

Clonality index categorical

|  | hr | 95% CI | P-val |
| --- | --- | --- | --- |
| clon_ca2_High | 1.66 | (0.45-6.10) | 0.4433 |
| sexMale | 0.65 | (0.20-2.07) | 0.4655 |
| age | 1.08 | (1.00-1.16) | 0.0442 |
| siteRight | 0.23 | (0.04-1.24) | 0.0865 |

Clonality index continuous

|  | hr | 95% CI | P-val |
| --- | --- | --- | --- |
| clon | 1.06 | (0.96-1.18) | 0.2373 |
| sexMale | 0.67 | (0.21-2.19) | 0.5120 |
| age | 1.08 | (1.00-1.16) | 0.0443 |
| siteRight | 0.13 | (0.01-1.26) | 0.0783 |

combination

|  | hr | 95% CI | P-val |
| --- | --- | --- | --- |
| comb2_TCR-low/CLON-high | 0.00 | (0.00-Inf) | 0.9987 |
| comb3_TCR-high/CLON-low | 0.04 | (0.00-0.34) | 0.0038 |
| comb4_TCR-high/CLON-high | 0.26 | (0.04-1.74) | 0.1643 |
| data$sexMale | 2.49 | (0.52-12.02) | 0.2566 |
| data$age | 1.13 | (1.03-1.25) | 0.0108 |
| data$siteRight | 0.27 | (0.05-1.60) | 0.1500 |

**Combining TCR abundance and Clonality index**

Interaction categorical

|  | hr | 95% CI | P-val |
| --- | --- | --- | --- |
| tcr_ca2_high | 0.47 | (0.32-0.71) | 0.0003 |
| clon_ca2_high | 1.79 | (1.20-2.69) | 0.0046 |
| sexMale | 1.15 | (0.77-1.70) | 0.4991 |
| age | 1.02 | (1.00-1.04) | 0.0633 |
| siteRight | 0.66 | (0.43-1.00) | 0.0510 |
| siteUnknown | 0.00 | (0.00-Inf) | 0.9943 |
| stageII-B | 2.78 | (1.58-4.89) | 0.0004 |

Interaction continuous

|  | hr | 95% CI | P-val |
| --- | --- | --- | --- |
| tcr_z | 0.88 | (0.80-0.97) | 0.0111 |
| clon_z | 1.11 | (0.98-1.27) | 0.0996 |
| sexMale | 1.10 | (0.74-1.62) | 0.6509 |
| age | 1.02 | (1.00-1.04) | 0.0741 |
| siteRight | 0.67 | (0.44-1.03) | 0.0667 |
| siteUnknown | 0.00 | (0.00-Inf) | 0.9944 |
| stageII-B | 2.54 | (1.45-4.45) | 0.0011 |
