# Supplementary figures and images for: Lymphocytic infiltration in stage II microsatellite stable colorectal tumors: A retrospective prognosis biomarker analysis
Source: PLoS Med. 2020 Sep 24;17(9):e1003292. doi: 10.1371/journal.pmed.1003292 (PMC7514069; doi:10.1371/journal.pmed.1003292)

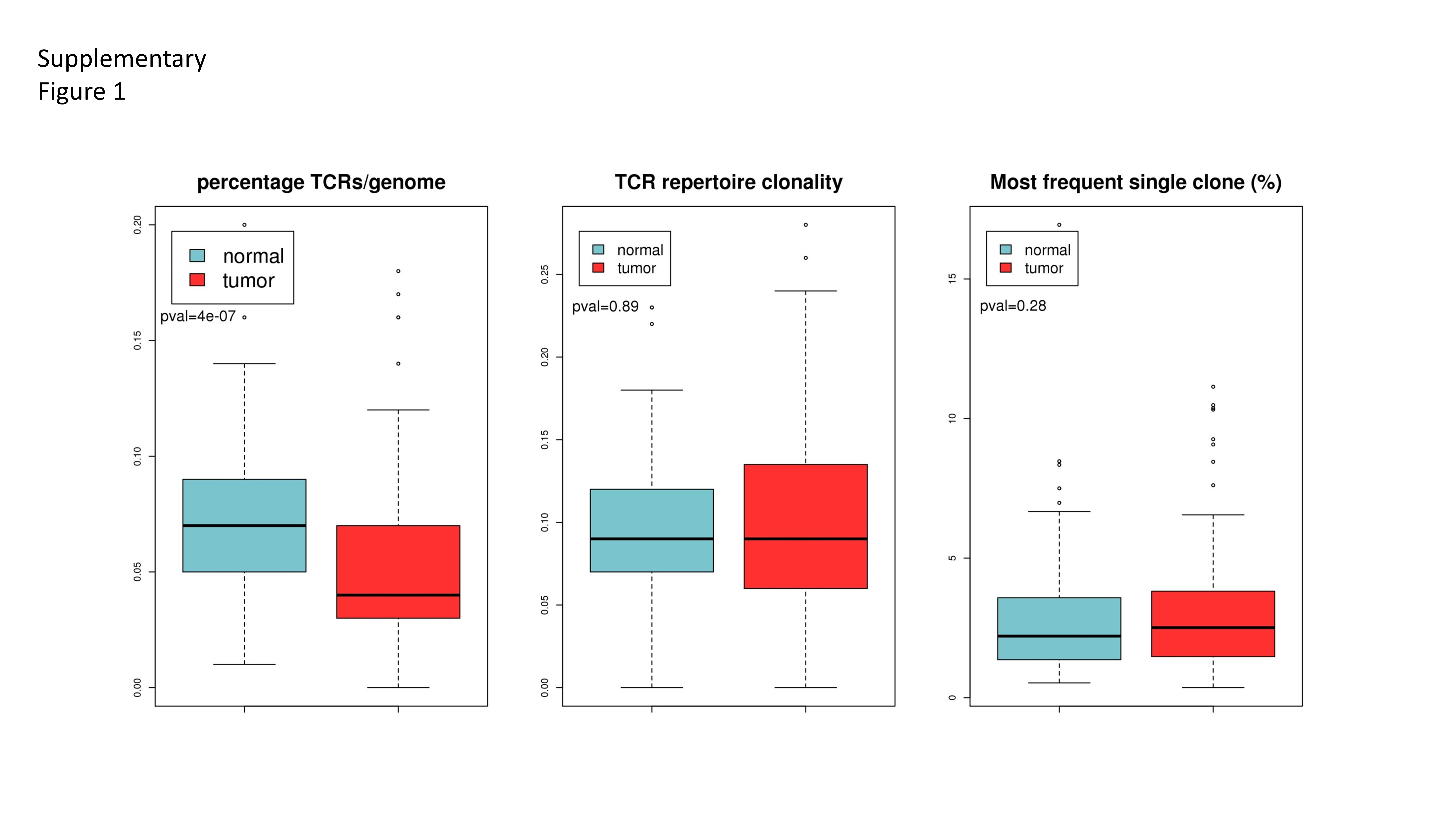

Supplement: S1 Fig — Boxplots showing differences between 96 adjacent normal and 96 tumor samples in: percentage of TCR (A). TCR repertoire clonality (B). Percentage of most frequent single clone (C); in the discovery ICO/CLX dataset. CLX, Colonomics study; ICO, Catalan Institute of Oncology; TCR, T-cell receptor. (TIF) [file pmed.1003292.s004.tif]

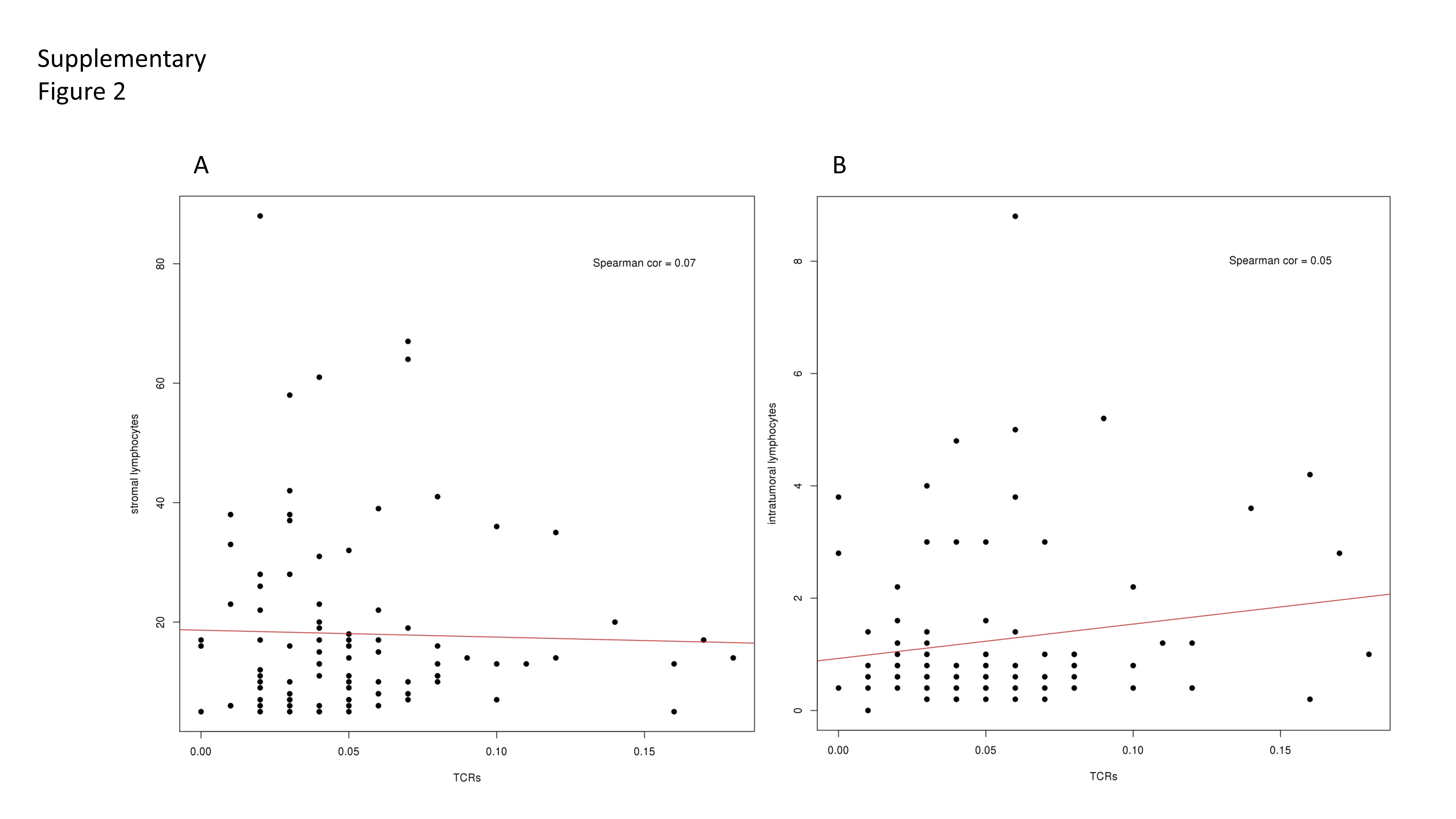

Supplement: S2 Fig — TCR, T-cell receptor; TIL, tumor-infiltrating lymphocyte. (TIF) [file pmed.1003292.s005.tif]

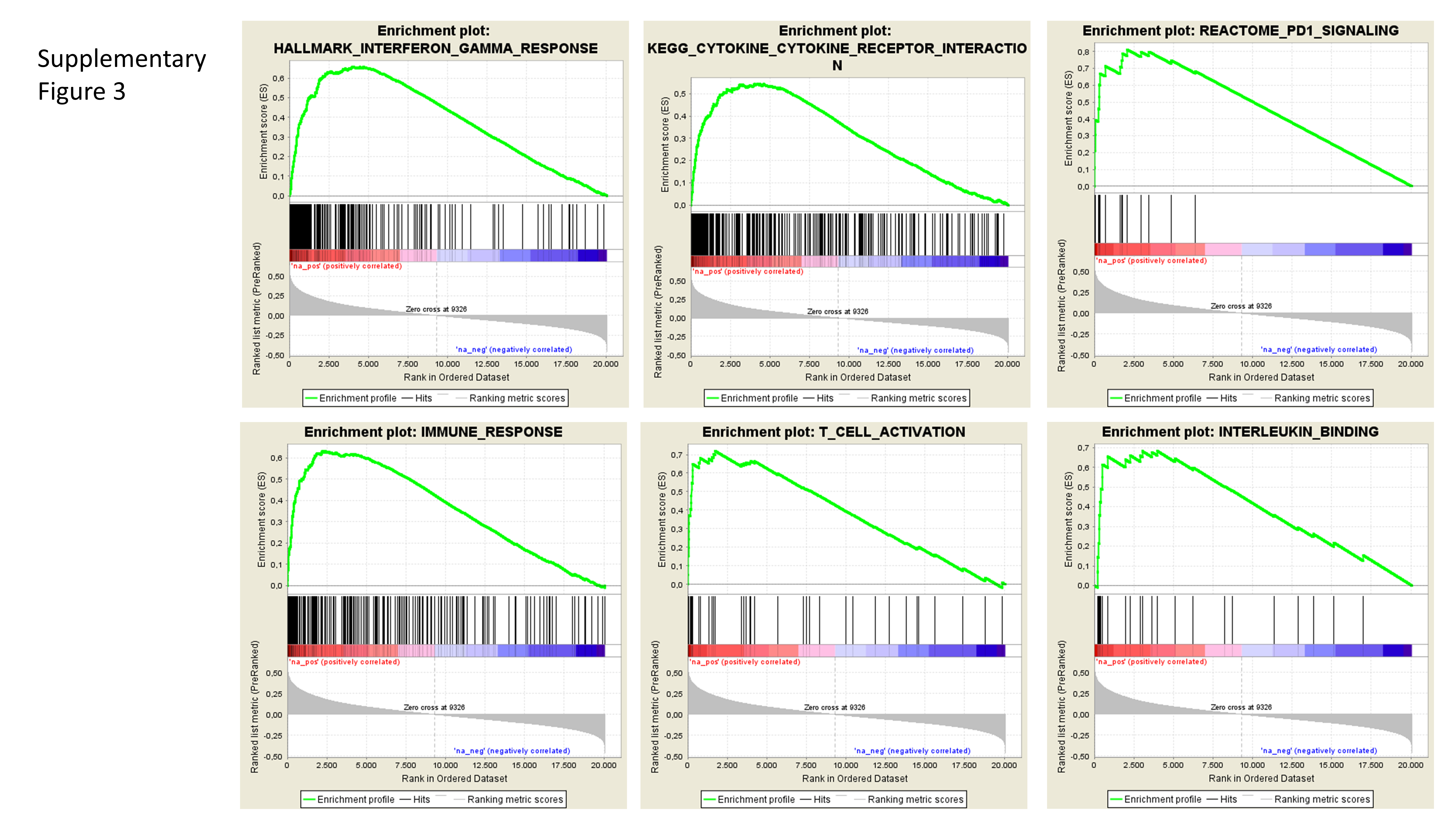

Supplement: S3 Fig — GSEA, Gene Set Enrichment Analysis; TCR, T-cell receptor. (TIF) [file pmed.1003292.s006.tif]

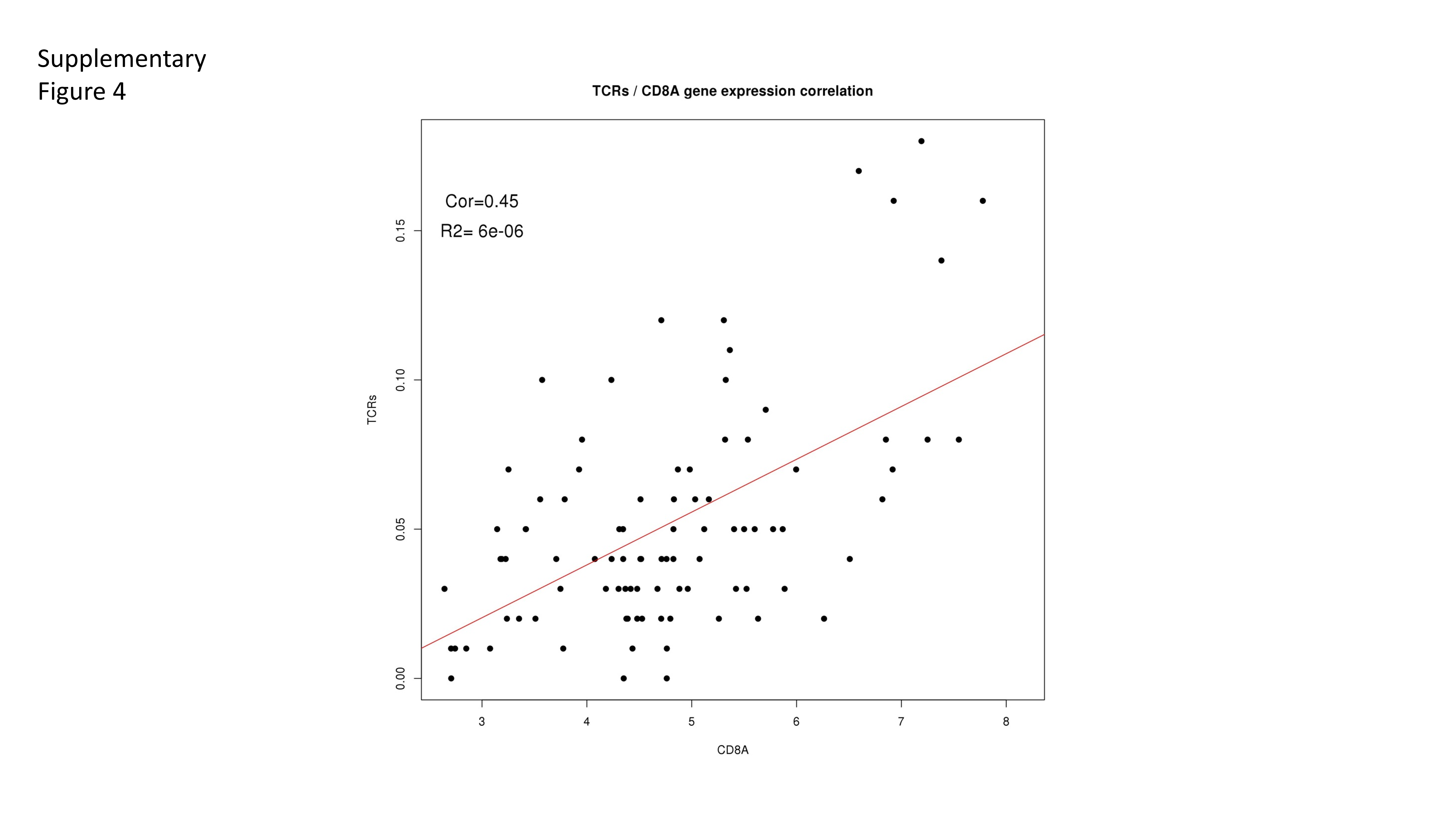

Supplement: S4 Fig — TCR, T-cell receptor. (TIF) [file pmed.1003292.s007.tif]

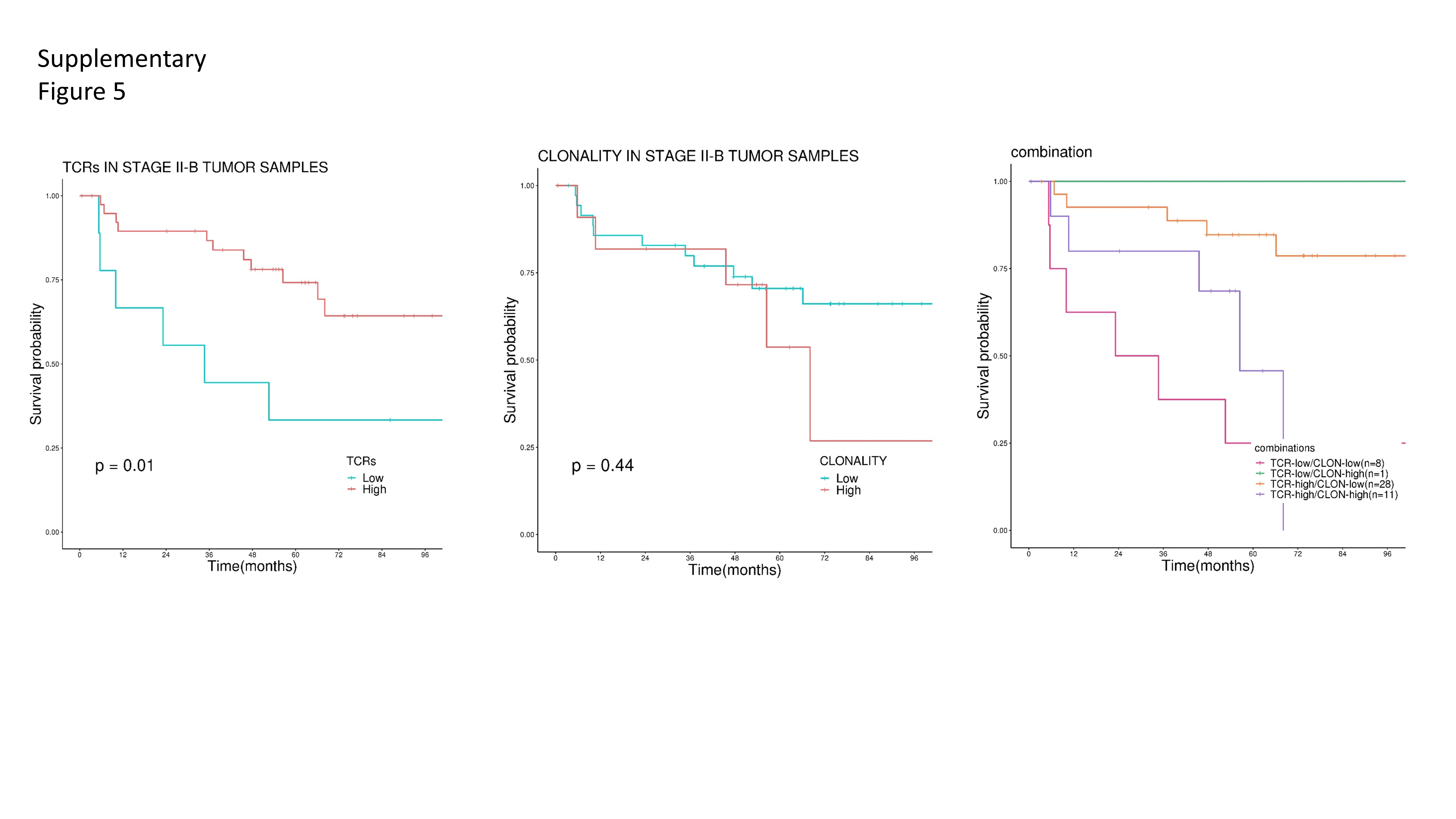

Supplement: S5 Fig — Kaplan–Meier curves dividing into high and low TCRs (A) and high and low clonality (B) categories in stage II-B tumors. C. Kaplan–Meier curve categorizing into 3 categories: high TCRs–high clonality, high TCRs–low clonality, low TCRs–high clonality, and low TCRs–low clonality. TCR, T-cell receptor. (TIF) [file pmed.1003292.s008.tif]
